# Mechanism-Inspired Ligand Design for Efficient Copper-Catalyzed C–N Coupling of Aryl and Heteroaryl Chlorides

**DOI:** 10.1021/jacs.5c20640

**Published:** 2026-02-16

**Authors:** Wei Zhao, Willi M. Amberg, Guodong Rao, Yuanzhe Xie, Christina N. Pierson, Serena M. Fantasia, Stephan M. Rummelt, Kurt Püntener, R. David Britt, John F. Hartwig

**Affiliations:** a Department of Chemistry, 1438 University of California, Berkeley, Berkeley, California 94720, United States; b Department of Chemistry, 8789 University of California, Davis, Davis, California 95616, United States; c Pharmaceutical Division, Synthetic Molecules Technical Development, Process Chemistry and Catalysis, F. Hoffmann-La Roche Ltd., Basel CH-4070, Switzerland

## Abstract

Cross-coupling reactions to form C–N bonds catalyzed by copper are becoming sustainable and cost-effective alternatives to those catalyzed by palladium. An array of ligand classes has been reported over the past two decades to create copper catalysts that couple aryl iodides and bromides. However, these systems typically require higher catalyst loadings than are required for palladium, and the couplings of aryl chlorides catalyzed by copper complexes require particularly high loadings, high temperatures, or both. We report a catalytic system designed to destabilize the bis-ligated copper­(II) oxalamide complexes that are the major species in reactions of aryl bromides catalyzed by complexes of oxalamide ligands. A sterically hindered oxalamide ligand in combination with Cu­(I) or Cu­(II) leads to the coupling of aryl and heteroaryl chlorides with a set of primary amines, as well as aqueous ammonia, under mild conditions at low catalyst loadings (0.03–1 mol %) with turnover numbers up to 2300. Mechanistic studies reveal that increased steric bulk on the amide causes a monoligated copper species with a Cu­(I) oxidation state to be the major copper complex in the reactions of aryl chlorides. Both Cu­(I) and Cu­(II) amine complexes containing this oxalamide have been isolated, structurally characterized, and observed in the catalytic systems by EPR and NMR spectroscopy. Initial kinetic studies indicate that oxidative addition to the amine complex is rate limiting, and the identity of the halide on the aryl halide (Cl vs Br) changes the oxidation state of the major copper species in solution.

## Graphical abstract

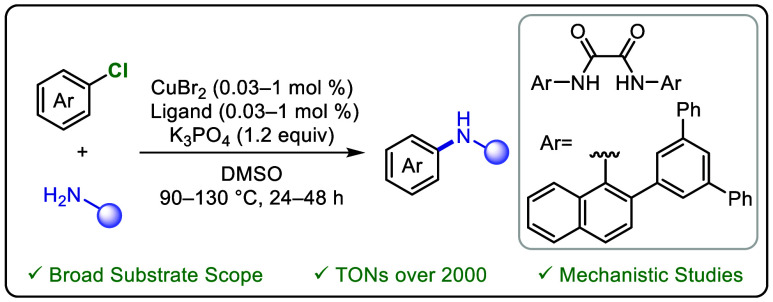

## Introduction

The coupling of amines with aryl halides has become one of the most widely used reactions for the synthesis of pharmaceuticals, agrochemicals, fine chemicals, polymers, and organic electronics.^1^ Following Ullmann’s seminal publication on copper-mediated C–N cross-coupling in 1903, significant effort has been devoted to develop copper catalysts.^2^ Most recently, catalysts based on copper have begun to rival the activity of those based on palladium in some cases.^3^

Advances in ligand design over the past two decades have led to systems with copper that couple aryl bromides and aryl iodides with a range of amines and other nitrogen nucleophiles (Figure 1).^4^ However, the coupling of the less reactive aryl chlorides, particularly at low catalyst loadings, remains rare.^5^ In 2015 Ma and co-workers reported an oxalamide-based system that couples aryl chlorides with amines in the presence of 5 mol % CuI and ligand **L1** at 120 °C, achieving turnover numbers (TONs) of up to 40 (Figure 1A).^6^ More recently, Buchwald, Liu and co-workers reported a diamine that forms a copper catalyst that couples aryl chlorides and primary amines at 70 °C in the presence of 5 mol % CuBr and 10 mol % ligand with TONs of up to 20 (Figure 1B).^7^ Clearly, significantly higher turnover numbers with similar turnover frequencies are desirable, particularly with ligands that are easily accessible.

**Figure 1:**
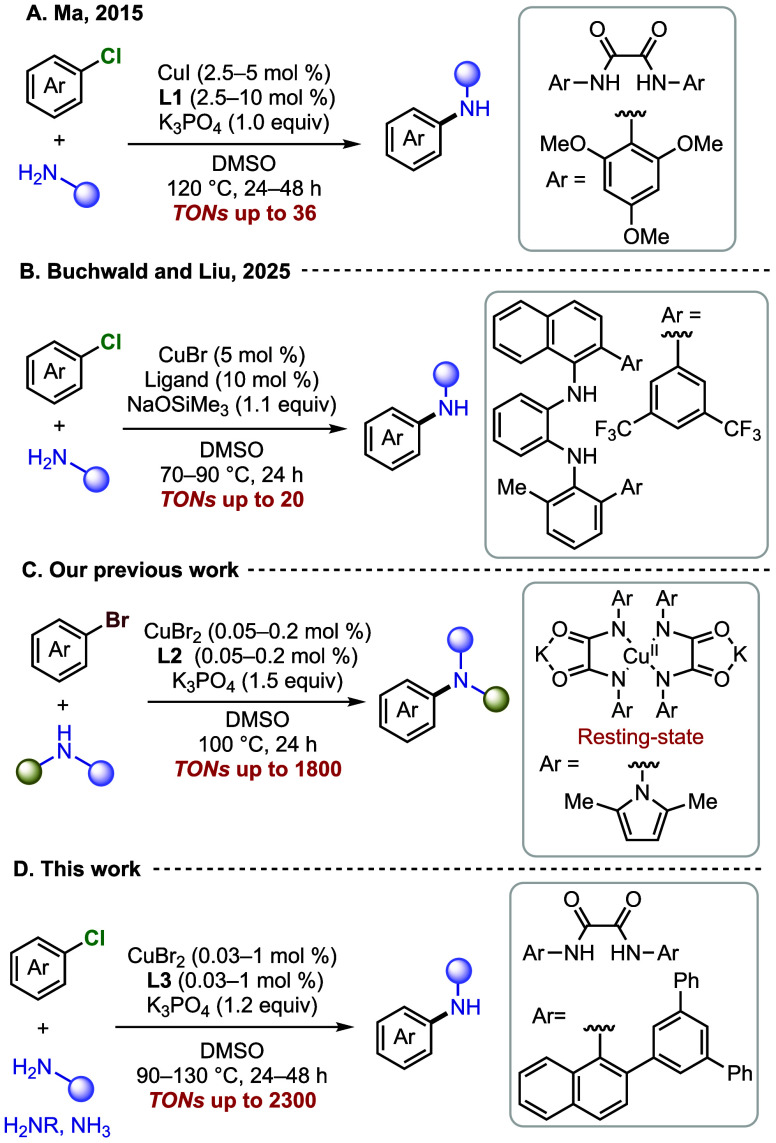
(A, B) Recent Cu-catalyzed couplings of aryl chlorides with amines. (C) Coupling of aryl bromides with high TONs. (D) This work.

We recently reported an oxalohydrazide-based ligand that forms a copper complex capable of catalyzing the coupling of aryl and heteroaryl bromides with amines, achieving TONs of up to 1800 (Figure 1C).^3b^ In that study, we showed that the major Cu complex in the catalytic reaction is a bis-ligated Cu­(II) complex and proposed that dissociation of a ligand is required to generate the active catalyst. We further demonstrated that a closely related bis-oxalamide Cu­(II) species catalyzes the coupling of O-nucleophiles with aryl bromides upon dissociation of one oxalamide ligand to generate the active catalyst.^8^ We reasoned that an increase in the steric bulk at the amide moiety of the oxalamide would disfavor formation of this bis-ligated Cu­(II) complex, thereby favoring the formation of a monoligated copper complex that could react with the aryl halide.

We report the combination of copper and a hindered oxalamide ligand (Figure 1D) that catalyzes the coupling of aryl and heteroaryl chlorides with amines at catalyst loadings as low as 0.03 mol %. We attribute the high efficiency of this system to the steric hindrance of the ligand that suppresses the formation of bis-ligated Cu­(II) species. Independent synthesis of amine-ligated Cu­(II) and Cu­(I) species, analysis of the catalyst composition by EPR and NMR spectroscopy, and kinetic studies show that the major species in the reactions of aryl chlorides is a Cu­(I) complex containing one oxalamide and one amine ligand, whereas the major copper complex in reactions with aryl bromides is a Cu­(II) species. Kinetic studies on the reactions of aryl chlorides show that oxidative addition of the aryl chloride, likely to the Cu­(I) amine complex, is rate determining.

## Results and Discussion

### Development of Reaction Conditions

We commenced our studies of the coupling of aryl chlorides with ligands designed to prevent the accumulation of stable, dianionic Cu­(II) complexes containing two X_2_-type ligands by investigating the coupling of *p*-methoxyphenyl chloride (**1a**) with *n*-hexylamine (**2a**) (Table 1). To evaluate our hypothesis that sterically hindered substituents at nitrogen would suppress the formation of such complexes and promote the C–N cross-coupling, we synthesized a set of oxalamides possessing *N*-aryl groups of varying size, including ligand **L1**, previously reported by Ma and co-workers for the coupling of aryl chlorides at high loadings (5 mol %).^6^ The coupling reaction with K_3_PO_4_ as base, CuBr_2_ (1 mol %) as precursor, and **L1** (1 mol %) as ligand at 90 °C afforded the coupled product in just 8% yield (Table 1, entry 2). The same reaction with our oxalohydrazide based ligand **L2** occurred in just 9% yield (entry 3). However, substantially higher yields were observed with ligands containing larger *N*-aryl groups. Ligand **L4** containing 2-phenylnaphthalene formed the product in 62% yield (entry 4), whereas the reaction with **L3** containing additional substituents in the 3,5-positions of the phenyl moiety furnished arylamine **3a** in a nearly quantitative 96% yield (entry 1).

**Table 1:**
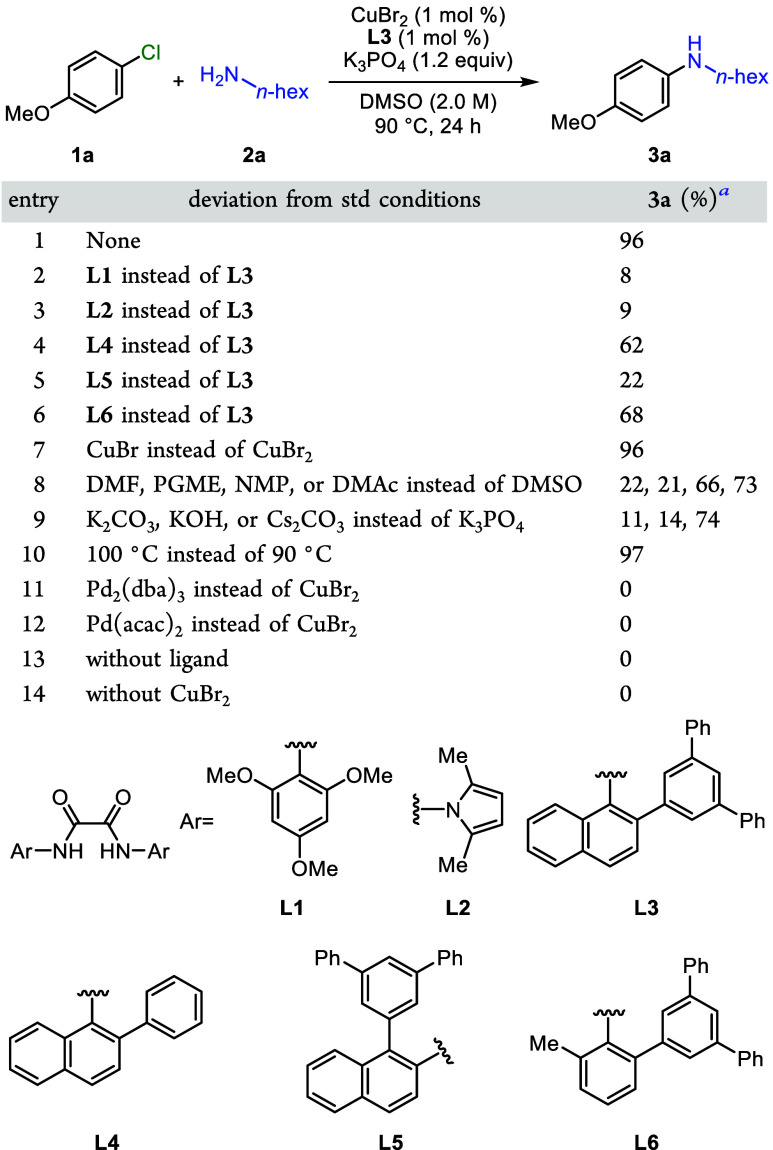
Effect of Varying Reaction Components and Conditions on the Reaction Yield

a Determined by ^1^H NMR spectroscopy with 1,3,5-trimethoxybenzene as internal standard.

**L3** was prepared in two steps and isolated in high yield without column chromatography. Suzuki-Miyaura coupling of 2-bromo-1-aminonaphthalene with commercially available 3,5-diphenyl-phenylboronic acid and subsequent reaction of the resulting amine with oxalyl chloride generated ligand **L3**.^4f^ We prepared 12.6 g of this ligand in 93% overall yield, as described in the Supporting Information. We note that high yields for C–N cross coupling were achieved with only a 1:1 ratio of ligand to Cu. This low ratio is important because the ligand, even one that is easily prepared like **L3**, is more costly than the copper precursor.

Further increasing the steric bulk around the Cu center did not lead to higher activity. For instance, the ligand containing the same 3,5-diphenyl-substituted arene at position 1 instead of position 2 of the naphthalene (**L5**) led to a catalyst that gave the product in only 22% yield (entry 5). Reducing the steric bulk on the backbone from naphthalen-2-amine to *o*-toluidine (**L6**) also led to a catalyst that reacted in lower yield (entry 6).

Conducting the cross-coupling reaction with CuBr instead of CuBr_2_ led to no change in yield (96%, entry 7). Reactions in DMF, PGME, NMP, or DMAc in place of DMSO occurred in lower 22%, 21%, 66%, or 73% yields, respectively (entry 8). Substituting K_3_PO_4_ with K_2_CO_3_, KOH, or Cs_2_CO_3_ also led to lower yields (entry 9). In the absence of ligand or Cu, or when a palladium precatalyst (Pd_2_(dba)_3_ or Pd­(acac)_2_) was used in place of copper, no reaction was observed; the starting material was unchanged (entries 11–14).

### Evaluation of Reaction Scope

With the optimized conditions in hand, we investigated the scope of the couplings of aryl chlorides (Figure 2). First, we conducted reactions of benzylamine with a series of aryl chlorides. Reactions of both electron-rich and electron-poor aryl chlorides formed the coupled product in excellent yield. The results in Figure 2 also show that the transformation is tolerant of a wide range of functionalities, including, but not limited to, aromatic nitriles, esters, amides, ketones, ethers, and nitroarenes; aryl amines **3b**–**l** were obtained in 87% to quantitative yield. A range of heteroaryl chlorides also reacted well under the established conditions. For example, 5-chlorobenzothiophene, 2-chloropyridine, and 3-chloropyridine derivatives formed the coupled product in excellent yields (**3n**–**3r** 91% to quant.). 7-Chloro- and 6-chloroquinoline also furnished the corresponding coupled products, **3s** and **3t**, in high quantitative and 97% yields, respectively. Etoricoxib (**1u**), a nonsteroidal anti-inflammatory drug and selective COX-2 inhibitor, is used to relieve pain and inflammation in conditions such as osteoarthritis, rheumatoid arthritis, and acute gout attacks.^9^ The compound contains an aryl chloride functionality, which was amenable to our reaction conditions, yielding **3u** in 77% yield and demonstrating the suitability of our method for late-stage functionalization.

**Figure 2:**
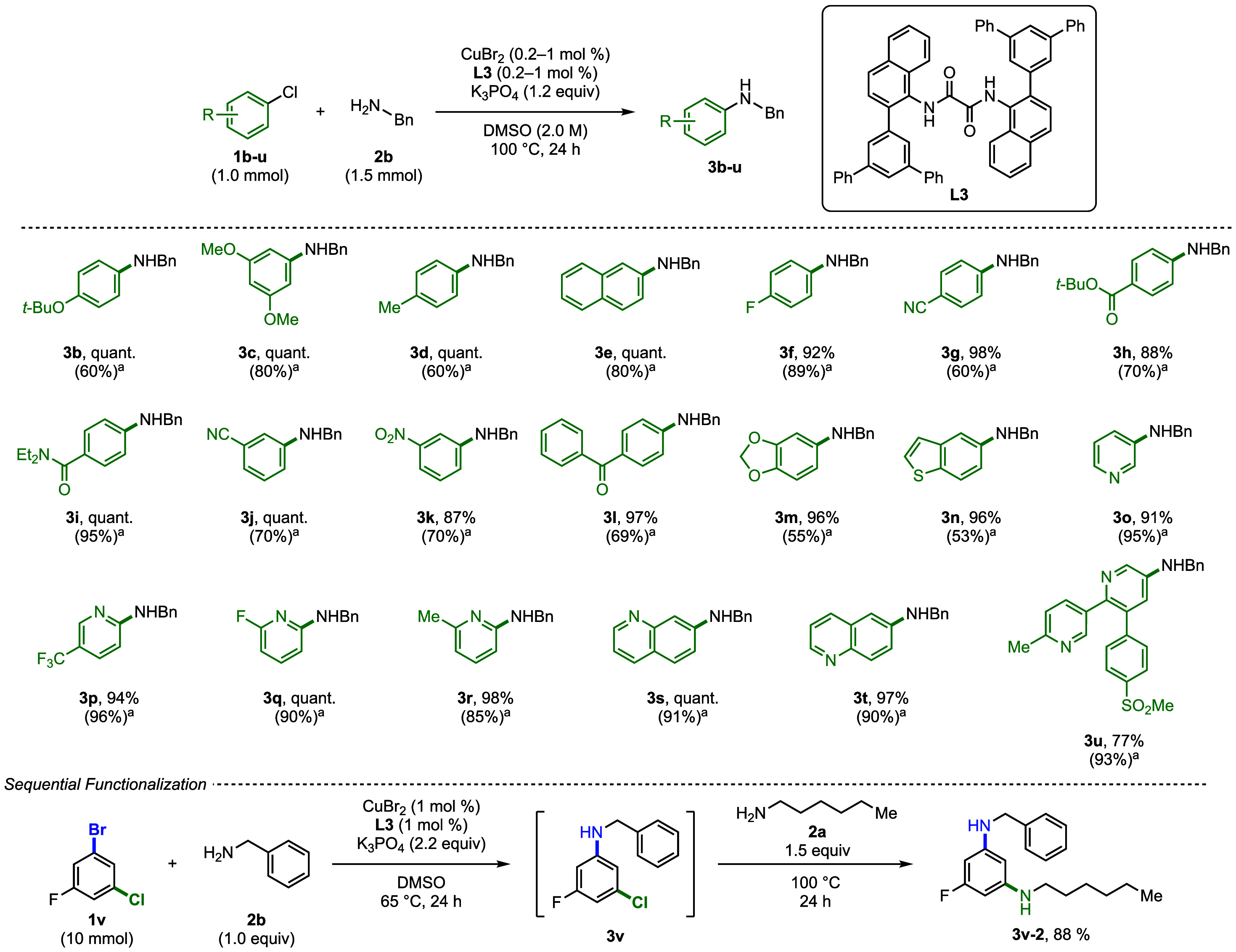
Cu-catalyzed C–N coupling of benzylamine with (hetero)­aryl chlorides. Reaction conditions: (hetero)­aryl chloride (1.0 mmol), amine (1.5 mmol), K_3_PO_4_ (1.2 mmol), CuBr_2_ (1.0 mol %), **L3** (1.0 mol %) in DMSO (0.5 mL) heated to 100 °C for 24 h. Reported yields are isolated. ^a^The reactions were performed with 0.2 mol % CuBr_2_ and 0.2 mol % **L3**. Yield obtained by ^1^H NMR spectroscopy with 1,3,5-trimethoxybenzene as an internal standard. Sequential functionalization of aryl dihalides, reaction conditions: **1v** (1.0 equiv), NH_2_Bn (1.0 equiv), K_3_PO_4_ (2.2 equiv), CuBr_2_ (1 mol %), **L3** (1 mol %) in DMSO (2 M) heated to 65 °C for 24 h. Then **2a** (1.5 equiv) was added and the reaction mixture was heated to 100 °C for 24 h.

To assess the potential of these reactions to occur with low loadings of Cu, we repeated the C–N couplings but with 0.2 mol % of CuBr_2_ and **L3** under otherwise identical reaction conditions. The reactions of electron-rich and electron-neutral arenes **1b**–**e** proceeded in good yield while maintaining high turnover numbers (300–400 TON). In addition, substrates **1f**–**1l** bearing an electron-withdrawing group, such as F, CN, CO_2_ *t*‑Bu, C­(O)­NEt_2_, NO_2_, or C­(O)­R, afforded the coupled product in comparable yield as observed with 1 mol % catalyst loading (60–95% yield, 300–475 TON). The yield for reactions of heterocycles **1m** and **1n** was lower (55% and 53%, respectively), but the reactions of pyridine and quinoline derivatives occurred in excellent yields with just 0.2 mol % copper and ligand (**3o**–**3u**, 85–96%, 425–480 TON).

To assess the degree to which the product might form from an S_N_Ar pathway or by trace impurities in ligand **L3**, we conducted control experiments with eight aryl chlorides. The reaction was conducted in the absence of copper but with 1 mol % of **L3** under otherwise identical reaction conditions with electron-rich, electron-poor, and heterocyclic aromatic halides (**1a**, **1c**, **1e**, **1f**, **1j**, **1k**, **1o**, and **1w**). Only trace amounts of product (<1% yield) were observed in the crude reaction mixture in all cases by ^1^H NMR spectroscopy.

The sequential functionalization of arenes bearing two reactive halides, one chloride and one bromide, was also investigated (Figure 2). The reaction of 1-bromo-3-chloro-5-fluorobenzene **1v** and benzylamine (1.0 equiv) was conducted with 1 mol % CuBr_2_ and 1 mol % **L3** at 65 °C. After 24 h 1-amino-3-chloro-5-fluorobenzene **3v** formed, and 1.5 equiv of *n*-hexylamine was added to the reaction mixture. After an additional 24 h, the diamine product was isolated in 88% overall yield.

The scope of amines that react under these conditions is shown in Figure 3. The reactions with 1 mol % of Cu and 1 mol % of **L3** at 100 °C consistently formed the coupled product in excellent yields with primary alkylamines. Heterocycles and functional groups on the amine, such as a thiophene (**5a**), furan (**5b**, **5l**), cyclopropane (**5h**), pyridine (**5i**), morpholine (**5j**), piperidine (**5k**), or enol ether (**5n**), formed the cross-coupled products in 83% to quantitative yields. The reaction of *p*-trifluoromethyl­chlorobenzene and 4-aminobutan-1-ol exclusively formed the C–N coupled product in 92% yield. The reaction of 4-(aminomethyl)­aniline gave the product from coupling at the alkylamine over the arylamine exclusively. These examples highlight the high selectivity of this catalyst system for the coupling of alkylamines and illustrate that the reaction tolerates the presence of unprotected anilines and alcohols.

**Figure 3:**
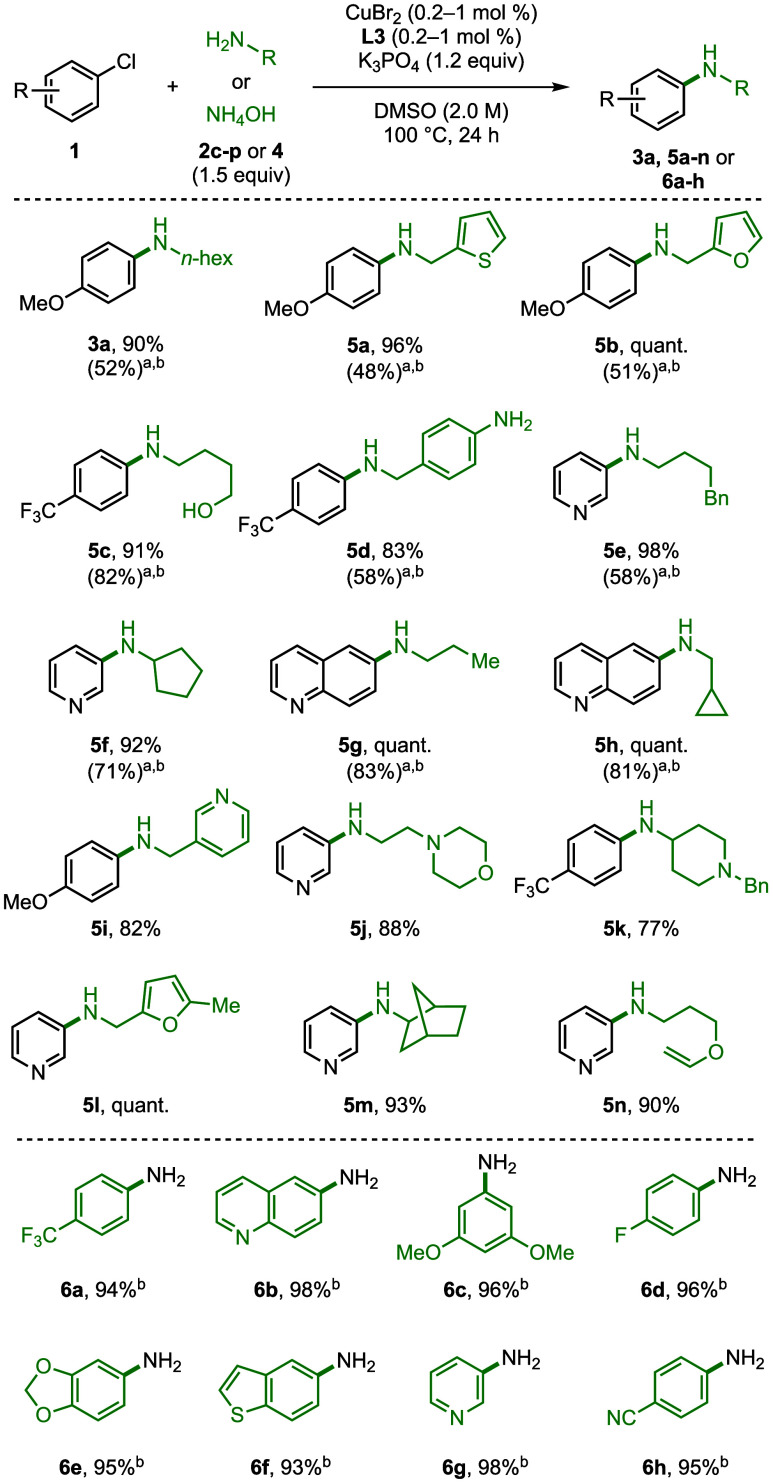
Investigation of the coupling of various primary amines with electron-rich, electron-neutral, and electron-poor (hetero)­aryl chlorides. Reaction conditions: (hetero)­aryl chloride (1.0 mmol), amine (1.5 mmol), K_3_PO_4_ (1.2 mmol), CuBr_2_ (1.0 mol %), **L3** (1.0 mol %) in DMSO (0.5 mL) heated to 100 °C for 24 h. ^a^The reactions were performed with 0.2 mol % CuBr_2_ and 0.2 mol % **L3** under otherwise identical reaction conditions. ^b^Yield obtained by ^1^H NMR spectroscopy with 1,3,5-mesitylene as the internal standard.

Again, reactions with just 0.2 mol % of CuBr_2_ and 0.2 mol % of **L3** were tested, and they occurred in good yields with a range of amines. Compounds **3a** and **5a**–**h** from coupling of electron-rich (**1a**), electron-poor (**1w**), or heterocycles (**1o**, **1t**) with amines **2a**, **2c**–**j** were all obtained in good to excellent yield (48–83%, 240–415 TON).

The coupling of ammonia with aryl chlorides is valuable to form synthetic intermediates and fine chemicals.^10^ Thus, we tested the coupling of aqueous ammonia with electron-rich and electron-poor aryl and heteroaryl chlorides (Figure 3, bottom). Anilines **6a**–**h** were obtained in 93–98% yield. No diarylamine product from coupling of the product aniline was observed.

### Evaluation of Turnover Numbers

Having demonstrated that the coupling of aryl chlorides occurs with a broad scope at low loadings of catalyst, we assessed the maximum turnovers for selected substrate combinations (Figure 4). The reaction of 1 mmol of 3-chloropyridine **1o** with 1.5 equiv of benzylamine, 1.0 equiv of K_3_PO_4_, 0.03–0.05 mol % of a pre-formed Cu­(I)/**L3**/amine complex (for details vide infra and Supporting Information) in DMSO at 130 °C for 48 h formed amine **5o** in 69% and 90% yield, respectively, corresponding to a turnover number of 2300–1800. The reaction of **1o** with 0.05 mol % of a pre-formed Cu­(I)/**L3**/amine complex on a 25 mmol scale formed the coupled product in 79% yield, corresponding to a TON of 1580. This value is noteworthy because the unhindered pyridine poisons or at least deactivates most coupling catalysts.^11^

**Figure 4:**
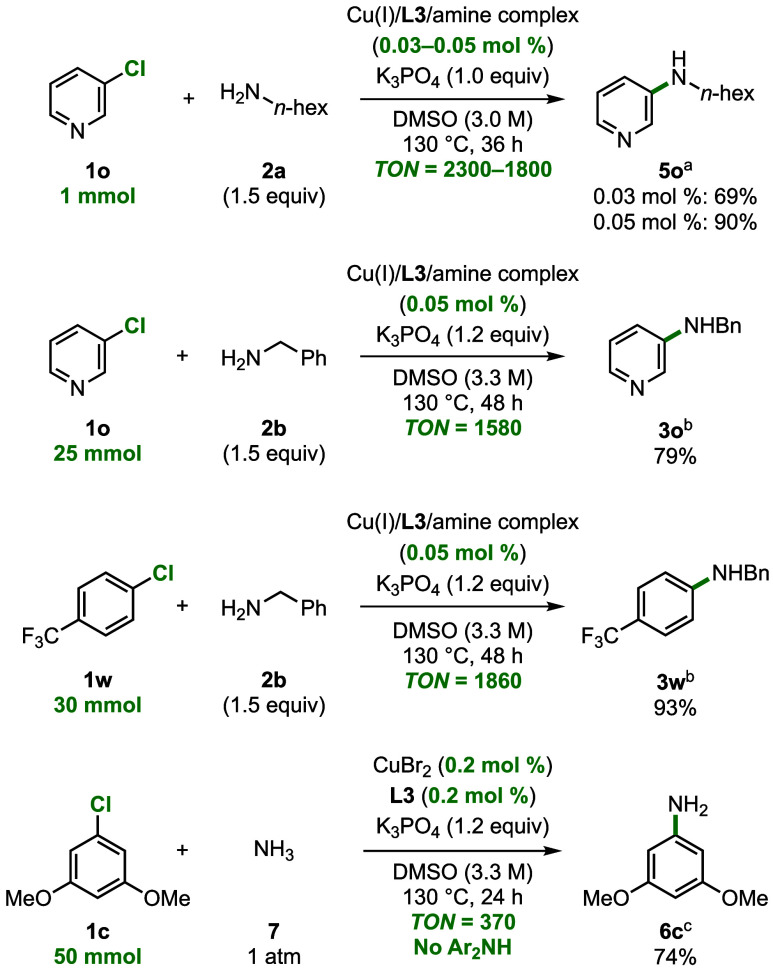
Investigation of reactions with low catalyst loading. ^a^(Hetero)­aryl chloride (1.0 equiv), amine (1.5 equiv), K_3_PO_4_ (1.0 equiv), pre-formed Cu­(I)/**L3**/amine complex (0.03–0.05 mol %) in DMSO (3 M) heated to 130 °C for 36 h. ^b^(Hetero)­aryl chloride (1.0 equiv), amine (1.5 equiv), K_3_PO_4_ (1.2 equiv), pre-formed Cu­(I)/**L3**/amine complex (0.05 mol %) in DMSO (3.3 M) heated to 130 °C for 48 h. ^c^ **1c** (1.0 equiv), NH_3_ (1 atm, via balloon), K_3_PO_4_ (1.2 equiv), CuBr_2_ (0.2 mol %), **L3** (0.2 mol %) in DMSO (3.3 M) heated to 130 °C for 24 h.

The coupling of 1-chloro-4-(trifluoromethyl)­benzene **1w** with **2b** on a 30 mmol scale produced **3w** in 93% yield, corresponding to a TON of 1860. These reactions on 25–30 mmol scale proceeded under reaction conditions (1.0–1.2 equiv of K_3_PO_4_, 3−3.3 M) and for reaction times (36–48 h) that are similar to those of reactions on the smaller 1 mmol scale.

To assess the amount of product that might form by an uncatalyzed S_N_Ar pathway, we conducted the C–N cross-coupling reaction of **1o** with *n*-hexylamine and benzylamine and of **1w** with benzylamine on a 1 mmol scale without Cu and **L3** under otherwise identical reaction conditions. Only minute quantities of product (1%, 3.5%, and 0.5%, respectively) were observed, confirming that the C–N coupled product results from reactions of our newly developed catalytic system.

The coupling of ammonia gas with electron-rich arene **1c** occurred with just 0.2 mol % CuBr_2_ and 0.2 mol % **L3** with a TON of 370. Again, no competing coupling of the aniline product to form the corresponding diarylamine was observed.

### Mechanistic Investigations

To understand the effect of the ligand, copper source, and halide on the reaction mechanism, we first determined the copper complexes in solution under various conditions. To do so, we obtained EPR spectra of reactions with labeled and unlabeled amine. The spectrum from the reaction of CuBr_2_ (5 mol %), **L3** (5 mol %), ^14^NH_2_Bn (1.50 equiv), and K_3_PO_4_ (1.20 equiv) in DMSO after 80 °C for 1 h is shown in Figure 5 (**EPR-1**). The same experiment was conducted with ^15^NH_2_Bn in place of ^14^NH_2_Bn (**EPR-2** in Figure 5, top). Examination of the hyperfine coupling pattern in the g_2_ region revealed differences between the ^14^NH_2_Bn and ^15^NH_2_Bn spectra, implying that the amine is coordinated to Cu­(II) in solution. This assignment was supported by spectral simulation, in which replacement of two ^14^N nuclei in **EPR-1** with two ^15^N nuclei reproduced the g_2_ features of **EPR-2** (for details see the Supporting Information). The EPR spectrum obtained for the combination of CuBr_2_, **L3** and K_3_PO_4_ with no amine is also shown in Figure 5 (**EPR-3**). Simulation of the observed hyperfine coupling pattern in this spectrum indicates that two ^14^N atoms are coordinated to the Cu­(II) center. This result implies that **L3** is too sterically congested to form a bis-ligated Cu­(II) complex like that observed previously, even in the absence of the amine. We suggest that the species observed in **EPR-3** containing two nitrogen atoms coordinated to copper is a dimeric complex or a monomer with coordinated DMSO. This species reacts with benzylamine to form the more stable Cu­(**L3**)­(NH_2_R)_2_ complex.

**Figure 5:**
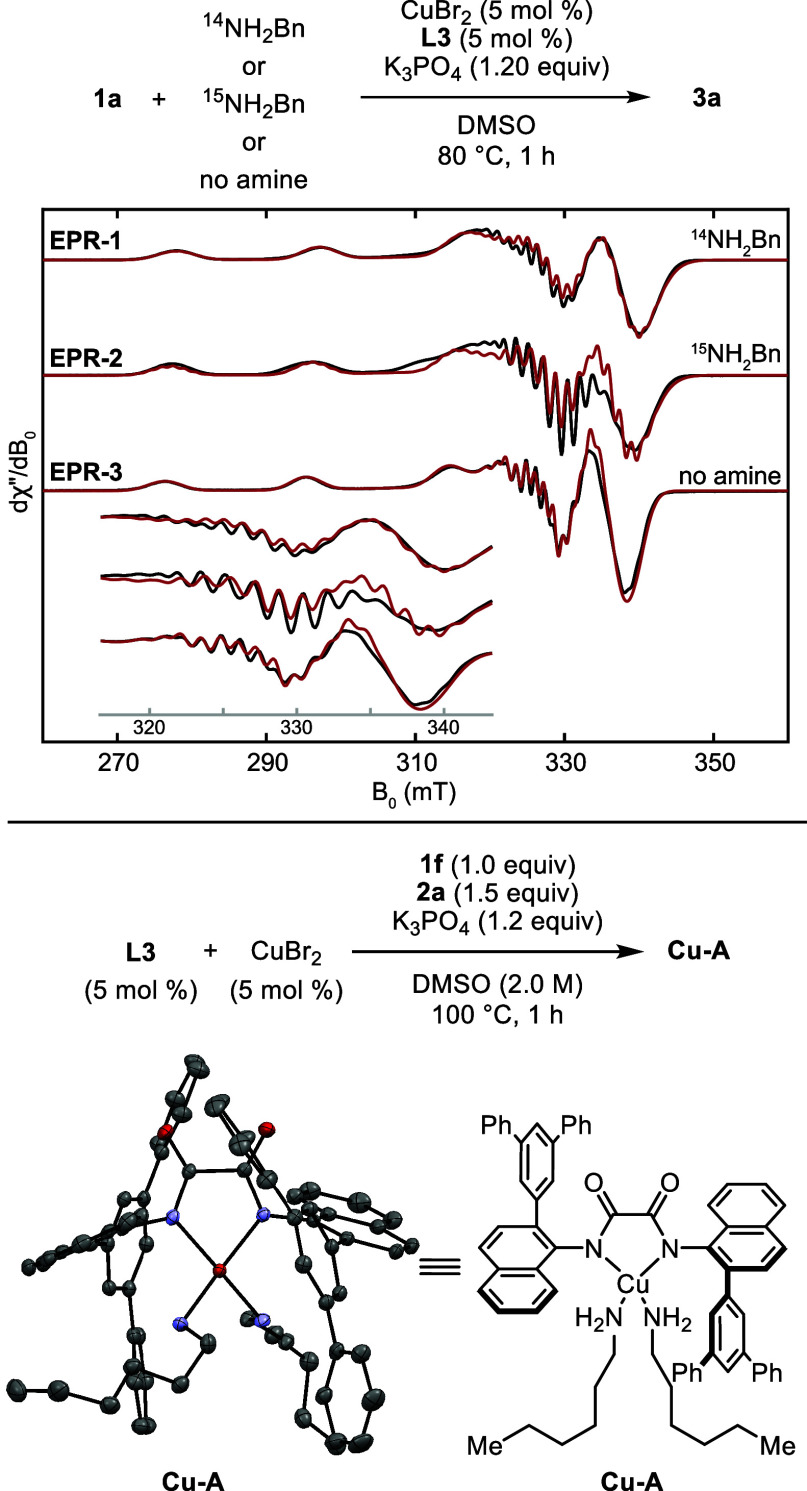
Analysis of copper complexes in the catalytic system. Top: EPR spectra of reaction mixtures containing ^14^N (**EPR-1**) or ^15^N (**EPR-2**) benzylamine or no amine (**EPR-3**). Black: experimental spectrum. Red: simulated spectrum. See Supporting Information for the simulation parameters. Bottom: synthesis and structure of complex **Cu-A**, showing 50% probability displacement ellipsoids (H atoms and DMSO omitted for clarity). CCDC deposition number for **Cu-A**: 2499046.

The **L3**-ligated Cu­(II) bis-amine complex was isolated by conducting the cross-coupling of *p*-F-chlorobenzene with *n*-hexylamine on a 2.5 mmol scale with 5 mol % CuBr_2_ and 5 mol % **L3** at 100 °C for 60 min. As noted below, this complex could not be prepared from CuBr_2_, ligand, base, and amine in the absence of haloarene because the amine and base reduce the Cu­(II) to Cu­(I). Rapid filtration to remove K_3_PO_4_ and allowing the resulting filtrate to stand for 12 h at room temperature yielded a red solid. Recrystallization from a mixture of DMSO and *n*-hexylamine and structural analysis of the resulting crystalline material by X-ray diffraction showed the complex to be **Cu-A**, in which the copper is coordinated by one oxalamide ligand (**L3**) and two *n*-hexylamines (Figure 5, bottom).

This structure matches the assignment deduced from the EPR data and is consistent with our design of an oxalamide ligand with sufficient steric bulk to favor complexes with a single oxalamide ligand. The reaction mixture from which complex **Cu-A** precipitated (Figure 5, bottom), was also analyzed by ^1^H NMR spectroscopy. The spectrum revealed trace amounts of material exhibiting well resolved aromatic signals that were distinct from those of aryl halide **1f**, arylamine, free ligand **L3**, or **Cu-A**. These signals suggested the presence of a diamagnetic Cu­(I) complex.

To determine the identity of the diamagnetic Cu­(I) species, we heated CuI (3 mol %), **L3** (3 mol %), K_3_PO_4_ (1.0 equiv), and *n*-hexylamine (1.0 equiv) in DMSO under a nitrogen atmosphere at 100 °C for 2 h. ^1^H NMR analysis of the resulting mixture revealed the same species that was observed in the filtrates from the synthesis of **Cu-A**. Heating CuI (3 mol %), **L3** (3 mol %), K_3_PO_4_ (1.0 equiv), and *n*-hexylamine (1.0 equiv) in DME under otherwise identical reaction conditions, followed by filtration of K_3_PO_4_ and slow evaporation of the solvent under nitrogen, gave deep red single crystals. Structural analysis by X-ray diffraction revealed the presence of the monoligated Cu­(I) dimer **Cu-B** shown in Figure 6A in which the two monomeric components are monoanionic and balanced by one potassium counterion.

**Figure 6:**
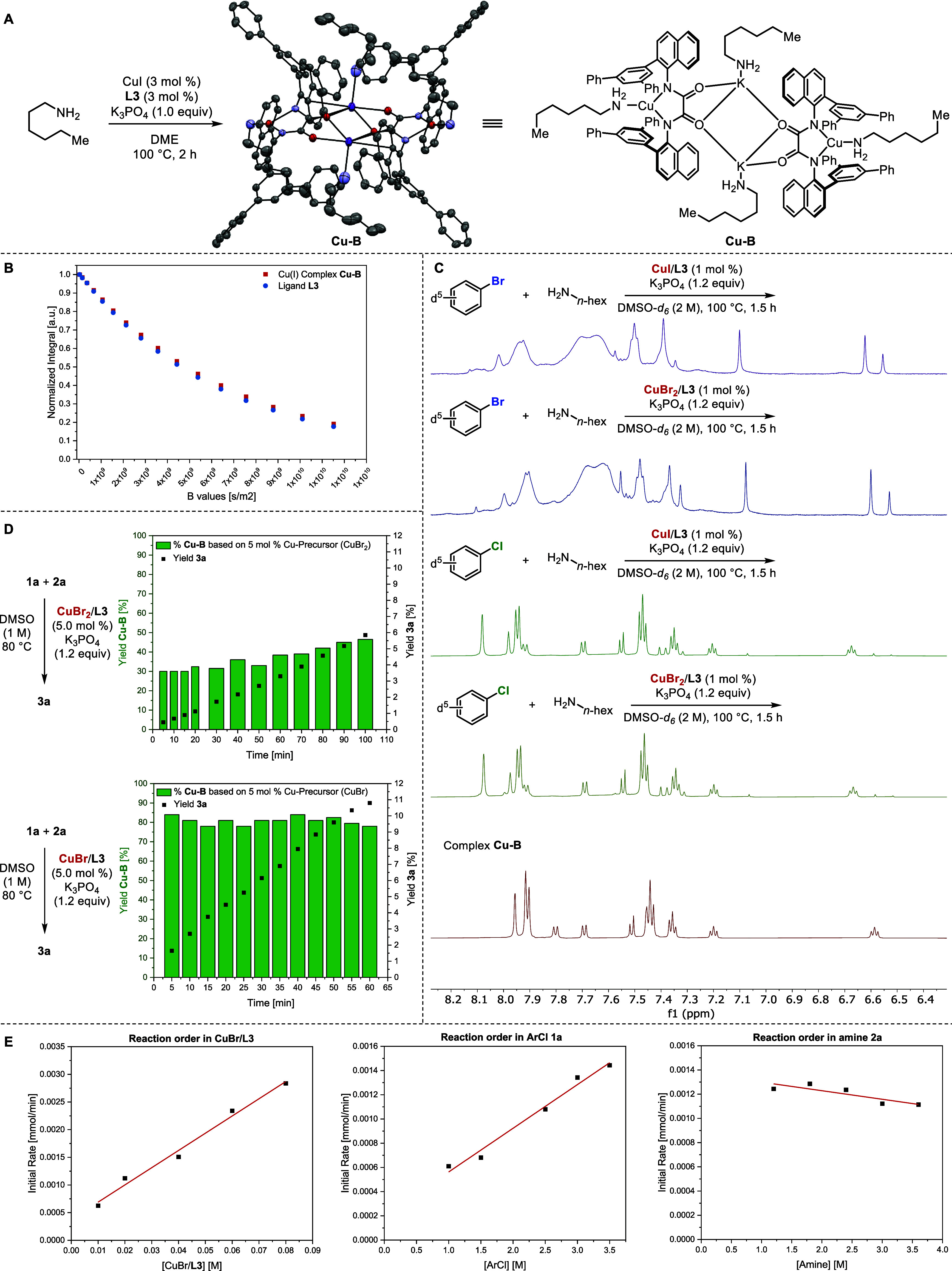
(A). Synthesis and X-ray structure of complex **Cu-B**, showing 50% probability displacement ellipsoids (H atoms are omitted for clarity). CCDC deposition number for **Cu-B**: 2503981. (B) DOSY traces for **L3** and complex B. (C) ^1^H NMR spectra of the unpurified reaction mixtures for the coupling of PhBr-*d*_5_ and PhCl-*d*_5_. (D) Assessment of the population of Cu­(I) catalyst by ^1^H NMR spectroscopy and initial rate of the coupling of **1a** with **2a** catalyzed by CuBr or CuBr_2_ with K_3_PO_4_ as the base in DMSO at 100 °C. (E) Order of the reactions in aryl chloride, amine, and copper for the coupling of **1a** with **2a** catalyzed by CuBr with K_3_PO_4_ as base in DMSO at 100 °C.

To determine whether **Cu-B** is dimeric or monomeric in solution, we conducted ^1^H diffusion ordered spectroscopy (DOSY) (Figure 6B, for details see the Supporting Information).^12^ The diffusion coefficient of this complex was 1.44 × 10^–10^ m^2^/s· For comparison, the diffusion coefficient of the free ligand **L3** was determined under identical experimental conditions and was found to be 1.54 × 10^–10^ m^2^/s. The similarity of the diffusion coefficients of the Cu complex to the free ligand implies that the hydrodynamic radius of the complex is similar to or slightly larger than that of the free ligand and is much smaller than that of the dimeric analogue.^13^ Thus, we conclude that complex **Cu-B** is predominantly a monomer in solution.

Our previous studies on the coupling of aryl bromides (e.g., *p*-fluorobromobenzene) with copper precursors and **L1** showed that the Cu­(I) complexes are rapidly oxidized to Cu­(II) species with concomitant formation of the dehalogenated arene (fluorobenzene).^3b^ To determine if the Cu­(I) complex of **L3** also would undergo oxidation with aryl halides to form Cu­(II), we conducted the amination of PhBr-*d*_5_ with *n*-hexylamine in the presence of 1 mol % **L3** and 1 mol % CuI or 1 mol % CuBr_2_ in DMSO-*d*_6_ at 100 °C (see the Supporting Information for experimental details). Under these conditions, no Cu­(I) complex was observed in the ^1^H NMR spectrum of the crude reaction mixture (Figure 6C). In contrast, the Cu­(I) species was clearly observed as the major Cu complex in the amination conducted with PhCl-*d*_5_ and CuI or CuBr_2_, under otherwise identical conditions (Figure 6C). These results imply that the weaker oxidizing potential of an aryl chloride versus an aryl bromide causes Cu­(I) to be present in the C−N coupling of PhCl-*d*_5_ with *n*-hexylamine. Due to the steric properties of **L3** disfavoring formation of a stable Cu­(II) complex and the weak oxidizing power of the aryl chloride, the Cu­(I) catalyst has a long lifetime for coupling of chloroarenes.

We presume that the high reactivity observed in the coupling of aryl chlorides arises from the formation of a stable Cu­(I) species that is reactive toward the oxidative addition of aryl chlorides. To assess this hypothesis, with the identity of the Cu­(I) and Cu­(II) species determined, we monitored reactions under standard conditions, which generate the Cu­(I) species and Cu­(II) species. Because the catalytic reactions are conducted with the heterogeneous base K_3_PO_4_, a separate reaction vial was assembled for each time point.

First, we ran the cross-coupling of chloroanisole **1a** with *n*-hexylamine **2a** with 5 mol % CuBr_2_ and 5 mol % **L3**. After 5 min at 80 °C, 30% of the Cu complexes in solution were Cu­(I) (Figure 6D, top). Over time, the amount of Cu­(I) gradually increased and was 44% after 100 min. In a second experiment, we ran the cross-coupling of **1a** with **2a** with CuBr as the source of copper. After 5 min, 82% of the Cu in solution remained Cu­(I), and the amount of Cu­(I) was constant between 78% and 82% over time, as determined by integration of signals corresponding to the Cu­(I) complex in the ^1^H NMR spectrum (Figure 6D, bottom). The amount of Cu­(II) in each reaction described in Figure 6C, which was conducted with 1 mol % of Cu catalyst, also was quantified by EPR spectroscopy. The amount of Cu­(II) in the reaction of PhBr-*d*_*5*_ was nearly 100% of all copper, while the amount of Cu­(II) in the reaction of PhCl-*d*_*5*_ was only 16–21% of the copper, matching the ratio of Cu­(I) to Cu­(II) measured by ^1^H NMR spectroscopy (for details see the Supporting Information).

With CuBr_2_ as the precursor, the initial rate was found to be 0.565 mM/min (Figure 6D). With Cu­(I) as the precursor, the initial rate was found to be 1.68 mM/min, or roughly 3 times greater than that with CuBr_2_. This difference in initial rate correlates with the roughly 2–3 times greater amount of Cu­(I) complex in solution with CuBr as precursor than with CuBr_2_ as precursor (78%–82% for CuBr precursor and 30–44% for CuBr_2_ precursor). Thus, we assert that the Cu­(I) is the major active species in this system for the coupling of chloroarenes, and that the reaction occurs by a cycle involving Cu­(I) and Cu­(III) species.

We propose that the amine serves as the reductant during the conversion of Cu­(II) to Cu­(I), forming trace amounts of imine in the process.^14^ Indeed, when a mixture of CuBr_2_ (10 mol %), **L3** (10 mol %), K_3_PO_4_, and *p*-fluorobenzylamine was heated in DMSO at 100 °C for 1.5 h, signals consistent with Cu­(I) were observed in the ^1^H NMR spectrum of the unpurified reaction mixture, while ^19^F NMR analysis indicated the presence of an imine (for details see Supporting Information). Further studies are needed to determine the path to reduction in reactions of ammonia, but Warren and co-workers have shown that a Cu­(I) dimer, bridged by a hydrazido dianion, can form from two Cu­(II) complexes of a parent amido (−NH_2_) ligand.^15^

Kinetic studies were conducted to assess the order in which the aryl halide and amine react in this catalytic cycle (Figure 6E). Initial rates were determined for the coupling of *p*-chloroanisole **1a** with *n*-hexylamine **2a**. Again, each measured time point was obtained from a separate reaction, due to the heterogeniety of the reaction. Our data showed that the reactions occur with a zero-order dependence in amine, first-order dependence in aryl chloride, and first-order dependence on the concentration of copper.

### Mechanistic Conclusions

Based on the independent synthesis of Cu complexes, EPR and NMR spectra of reaction mixtures and isolated complexes, ^1^H DOSY experiments on Cu­(I) complexes, and kinetic data, we propose that the coupling of aryl chlorides occurs by the mechanism shown in Figure 7. In this mechanism, Cu­(I) complex **Cu-C**, the generic monomeric form of **Cu-B**, which was isolated and fully characterized, undergoes oxidative addition to the aryl chloride to form the corresponding anionic Cu­(III) intermediate **Cu-D**, with an associated potassium cation. Following this oxidative addition, complex **Cu-D** likely undergoes deprotonation, possibly with concomitant dissociation of halide to avoid formation of a dianionic species, to generate a monoanionic Cu­(III) intermediate with an associated potassium cation **Cu-E**. Reductive elimination from this complex would form the product, and coordination of amine would regenerate the catalyst.

**Figure 7:**
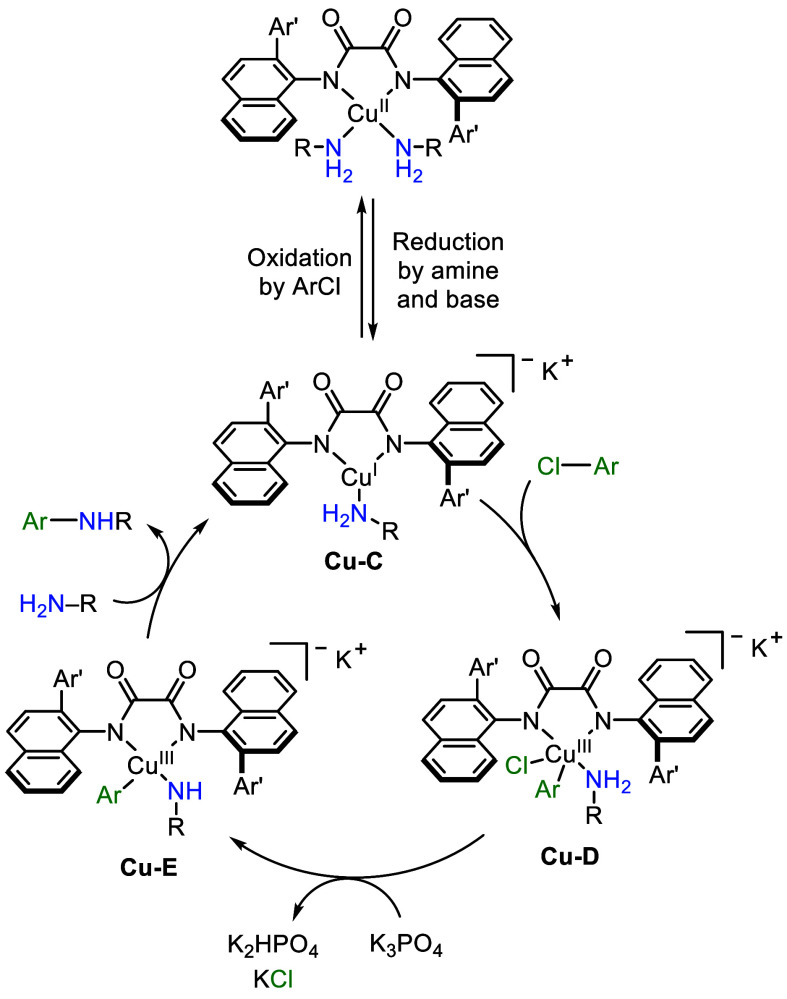
Proposed mechanism.

This mechanism contrasts the one we deduced from studies on the coupling of aryl bromides with Cu complexes of the less hindered **L1**.^3b^ The rapid oxidation of the Cu­(I) species with the aryl bromide to form Cu­(II) led us to propose that a cycle involving Cu­(II) and Cu­(III) occurs, and this observation is consistent with our observed effect of the halide on the identity of the Cu complex ligated by **L3**. Further studies on the reactions of complexes with systematically varied ligands and additional studies on reactions of aryl bromides will be conducted to determine which pathway occurs under the various conditions with different haloarenes, particularly with aryl halides that rapidly oxidize Cu­(I) to Cu­(II).

## Conclusion

Our mechanistic investigations of the identity of Cu complexes in cross-coupling reactions enabled the design of a ligand that stabilizes the catalytically active, monoligated Cu­(I) complex, thereby increasing the reactivity of the copper catalyst toward aryl chlorides and reaching turnover numbers of over 2000. Our catalytic system is broadly applicable, requiring merely 0.2 mol % loading for the coupling of primary amines and ammonia with electron-rich, electron-neutral, and electron-poor aryl chlorides, as well as heteroaryl chlorides. Key to success was the increased steric hindrance of the substituents on the amide moiety of the oxalamide ligand, favoring monoligated Cu­(II) and Cu­(I) species in the process. Mechanistic studies reveal the presence of Cu­(II) and Cu­(I) complexes in the catalytic reactions, with the latter being responsible for the coupling of aryl chlorides. We anticipate that the reactivity and insights provided in this work will encourage the application of Cu-based systems to coupling reactions that form C–N bonds, including those of typically less reactive aryl chlorides.

## Supplementary Material



## Notes

Experimental procedures and characterization data (PDF)
