# Supplementary material for: Mechanism-Inspired Ligand Design for Efficient Copper-Catalyzed C–N Coupling of Aryl and Heteroaryl Chlorides
Source: J Am Chem Soc. 2026 Feb 16;148(7):7505–14. doi: 10.1021/jacs.5c20640 (PMC12951465; doi:10.1021/jacs.5c20640)
Supplement: Supplementary file 1 [file ja5c20640_si_001.pdf]

Supporting Information

Mechanism-Inspired Ligand Design for Efficient Cu-Catalyzed C–N Coupling of (Hetero)aryl Chlorides

Wei Zhao,‡ Willi M. Amberg,‡ Guodong Rao, Yuanzhe Xie, Christina N. Pierson, Serena M. Fantasia, Stephan M. Rummelt, Kurt Püntener, R. David Britt and John F. Hartwig*

Table of Contents

1. General Remarks 2
2. Ligand Synthesis 5
3. Evaluation of the effect of reaction components and conditions on yield 14
4. EPR Spectroscopy Simulations 18
5. Quantification of Cu(I) Complex in Catalytic Reaction over Time 26
6. Characterization of Cu-Species in Catalytic Reaction via ^{1}H NMR and EPR Spectroscopy 31
7. Imine Formation from Cu(II) Reduction 36
8. Initial Rates for Coupling of 4-Chloroanisole with *n*-Hexylamine 37
9. General Procedures for C-N Cross Coupling of Aryl Chlorides with Amines 42
10. Substrate Scope 44
11. High Turnover Number Reaction 79
12. Sequential Amination of Aryl dihalides 82
13. Synthesis of Cu(I) and Cu(II) Complexes 84
14. ^{1}H, ^{13}C, and ^{19}F NMR Spectra 86
15. X-Ray Crystallographic Data 131
16. References 133

1. General Remarks

Procedure

Unless otherwise stated, all reactions were conducted in a nitrogen filled glove box. Reactions on 1 mmol scale were performed in 15x45 mm screw-thread vials (KIMBLE®, Clear Sample Vial, 4 mL, Catalog Nr. 60910-1) that were charged with a magnetic stirrer bar (PTFE, 12x4.5 mm, Fisherbrand™ Octagon Spinbar™, Catalog Nr. 14-513-57) and sealed with a screwcap (Qorpak™ Green Thermoset Cap with F217 and PTFE Liner, Catalog Nr. 272632) and electrical tape.

Chemicals

Unless otherwise stated, reagents and solvents were purchased from commercial suppliers (Acros, Sigma Aldrich, Strem, AmBeed) and used as received. The anhydrous K_3PO_4 used in optimization studies and for the substrate scope was finely ground and sieved to have a particle size <178 µm.

Thin-Layer Chromatography

Analytical thin layer chromatography (TLC) was performed on Merck Kieselgel 60 F254 fluorescent-treated silica and visualized via exposure to ultraviolet light (254 nm or 365 nm) or TLC stain (aqueous potassium permanganate solution followed by heating or aqueous ceric ammonium molybdate solution followed by heating).

Flash Column Chromatography

Flash column chromatography was performed using Teledyne Isco Combiflash® Rf system with RediSep GoldTM columns. Triethylamine-deactivated silica was prepared as follows: SiO_2 was equilibrated with a 5% solution of triethylamine in dichloromethane or hexane and poured into a chromatography column. The column was flushed several times with the eluent used for purification of the respective compound prior to loading.

Nuclear Magnetic Resonance Spectroscopy

All NMR spectra were recorded at the Pines Magnetic Resonance Center's Core NMR Facility (PMRC Core) in deuterated solvents at room temperature with a JEOL-400 (400 MHz, equipped with a ECZL400S console and a 5 mm HFX probe), Bruker NEO-500 (500 MHz, equipped with a Bruker Avance IV NEO console and a 5 mm 1H/BB iProbe), Bruker NEO-501 (equipped with a Bruker Avance IV NEO console and a 5 mm BBO Prodigy CryoProbe), or Bruker Avance 600 (600 MHz, equipped with a Bruker Avance III console and a 5 mm 1H/BB Prodigy CryoProbe). Chemical shifts are referenced to the solvent residual signal ($CDCl_3$, ^{1}H: δ = 7.26 ppm, ^{13}C: δ = 77.16 ppm or DMSO-d6, ^{1}H: δ = 2.50 ppm, ^{13}C: δ = 39.52 ppm) and reported in parts per million (ppm). The following abbreviations are used in reporting NMR data: s = singlet, d = doublet, t = triplet, q = quartet, br = broad, dd = doublet of doublets, m = multiplet, etc. Note: In some instances, the ^{13}C NMR spectrum contains overlapping aromatic resonances.

Diffusion-Ordered-Spectroscopy Nuclear Magnetic Resonance (DOSY-NMR)

DOSY spectra were recorded on a Bruker NEO-400 (400 MHz, equipped with a Bruker Avance IV NEO console and a diffusion probe, 17 T/m max. gradient strength, capable of measuring diffusion rates down to 10^{-13} to 10^{-14} m^2/s).

High-Resolution Mass Spectrometry

High-resolution mass spectrometry data was acquired at the Lawrence Berkeley National Laboratory Catalysis Laboratory on a PerkinElmer AxION 2 time-of-flight (TOF) electron spray ionization (ESI) mass spectrometer paired with a New Era Instruments NE-300 syringe pump.

X-Ray Crystallographic Analysis

X-ray crystallographic analysis was performed at the College of Chemistry X-Ray Crystallographic Facility (CHEXRAY, University of California, Berkeley). Measurements were collected on a Rigaku XtaLab P200 equipped with a MicroMax 007HF rotating anode and a Pilatus 200K hybrid pixel array detector. Data were

collected using Cu Kα radiation (λ = 1.5406 Å). Crystals were kept at100(2) K throughout the collection. Data collection was performed with CrysAlisPro.4 Data processing was done with CrysAlisPro and included a multi-scan absorption correction applied using the SCALE3 ABSPACK scaling algorithm within CrysAlisPro.

Electron Paramagnetic Resonance (EPR) Spectroscopy

Electron paramagnetic resonance (EPR) spectroscopy at 30 K was performed at the CalEPR center in the University of California, Davis. X-band (9.4 GHz) continuous wave spectra were recorded on a Bruker BioSpin EleXsys E500 spectrometer equipped with a super high Q resonator (ER4122SHQE), an ESR900 liquid helium cryostat with a temperature controller (Oxford Instrument ITC503), and a gas flow controller. All CW EPR spectra were recorded under slow-passage, nonsaturating conditions with the following spectrometer settings: temperature = 30 K, microwave power = 0.2 mW, conversion time = 60 ms, modulation amplitude = 0.8 mT, modulation frequency = 100 kHz. CW EPR spectra were simulated in Matlab R2025a using the EasySpin 6.0.10 toolbox.[1]

Electron paramagnetic resonance (EPR) spectroscopy at 77K was performed at the Lawrence Berkeley National Laboratory Catalysis Laboratory on a Bruker Magnettech ESR5000 Benchtop EPR spectrometer fitted with a quartz finger dewar for experiments at 77K. The software to record and evaluate the data is ESRStudio 1.90.0. Quantification of Cu(II) species was conducted with the instrument’s software ESRStudio 1.90.0.

2. Ligand Synthesis

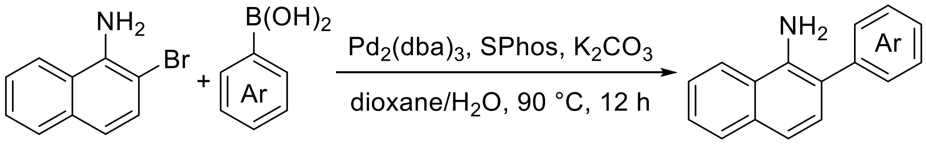

An oven-dried 500 mL Schlenk flask equipped with a Teflon-coated magnetic stir bar was charged with 2-bromonaphthalen-1-amine (9.04 g, 40.9 mmol, 1.00 equiv), aryl boronic acid (49.1 mmol, 1.20 equiv), $Pd_2(dba)_3$ (91.8 mg, 0.500 mol%), SPhos (336 mg, 2.00 mol%), and K_2CO_3 (11.3 g, 81.8 mmol, 2.00 equiv). After flushing with nitrogen for 3 min, dioxane (80 mL) and H_2O (20 mL) were added to the flask. The reaction mixture was tightly sealed, placed in an oil bath preheated to 90 °C, and stirred for 18 h. After 18 h, the reaction mixture was removed from the oil bath and allowed to cool to room temperature. At this point, the crude reaction mixture was diluted with EtOAc (100 mL) and H_2O (50 mL). The resulting suspension was filtered through a plug of Celite (ca. 2 cm), and the Celite plug was rinsed with additional EtOAc. The combined organic layers were dried over Na_2SO_4, and and the volatile materials evaporated under reduced pressure. The resulting crude product was recrystallized from hexane and dried in vacuo to yield the amine as a white solid.

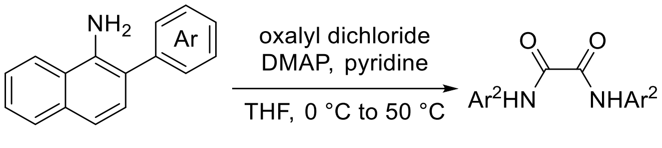

An oven-dried 250 mL Schlenk flask equipped with a Teflon-coated magnetic stir bar was charged with 2-arylnaphthalen-1-amine (40.0 mmol, 2.00 equiv) and 4-dimethylaminopyridine (DMAP, 2.00 mmol, 245 mg, 0.100 equiv). After evacuation and backfilling with nitrogen (3 cycles), THF (80 mL) and pyridine (200 mmol, 10.0 equiv) were added to the flask. The reaction mixture was cooled to 0 °C, and oxalyl chloride (2.54 g, 20.0 mmol, 1.00 equiv) was added dropwise. The reaction was then allowed to warm to room temperature. After stirring at room temperature for 2 h, the mixture was heated to 55 °C and maintained for 16 h. Upon cooling to room temperature, the volatile materials were evaporated under reduced pressure. Methanol (100 mL) was added to the residual solid, and the mixture was sonicated for

5 min. The resulting white suspension was filtered, and the white powder was further washed with methanol (20 mL × 2) and diethyl ether (30 mL × 3), then dried under vacuum to afford the corresponding ligand.

Compound L4:

***N*¹,*N*²-bis(2-phenylnaphthalen-1-yl)oxalamide**

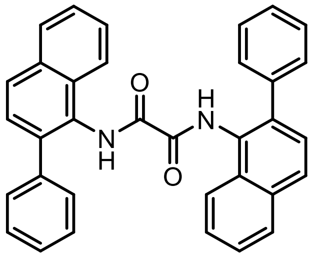

^{1}H NMR (500 MHz, DMSO-d$_6$) δ 10.68 (s, 2H), 8.09 – 7.98 (m, 4H), 7.79 – 7.72 (m, 2H), 7.66 – 7.57 (m, 6H), 7.53 – 7.44 (m, 10H).

^{13}C NMR (126 MHz, DMSO-d$_6$) δ 159.73, 139.33, 137.04, 132.97, 130.40, 129.61, 128.84, 128.19, 127.98, 127.87, 127.28, 126.96, 126.32, 123.48.

ESI-HR: calc'd for $C_{34}H_{24}N_2O_2Na^+$ ([M+Na]$^+$) 515.1731, found 515.1730, residual 0.19ppm

Compound L10:

***N*[1],*N*[2]-bis(2-(3,5-dimethylphenyl)naphthalen-1-yl)oxalamide**

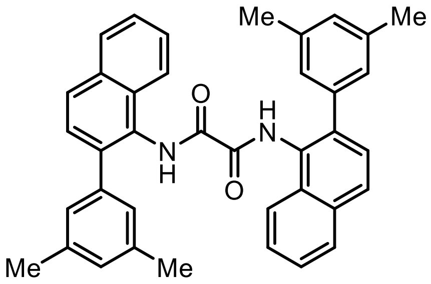

^{1}H NMR (500 MHz, DMSO-d$_6$) δ 10.70 (s, 1H), 8.07 – 7.96 (m, 2H), 7.74 – 7.66 (m, 1H), 7.64 – 7.53 (m, 3H), 7.16 (d, *J* = 1.6 Hz, 2H), 7.08 (s, 1H), 2.33 (s, 6H).

^{13}C NMR (126 MHz, DMSO-d$_6$) δ 159.94, 139.13, 137.13, 137.04, 132.92, 130.40, 129.57, 128.70, 128.04, 128.00, 127.76, 126.70, 126.19, 123.22, 21.01.

Compound L9:

***N*[1],*N*[2]-bis(2-(2,6-dimethylphenyl)naphthalen-1-yl)oxalamide**

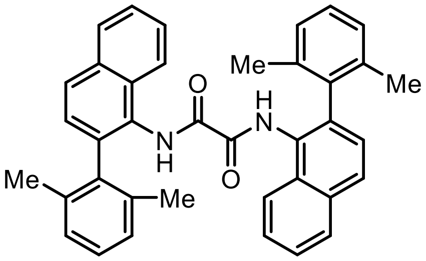

^{1}H NMR (500 MHz, DMSO-d$_6$) δ 10.13 (s, 2H), 8.06 – 7.93 (m, 4H), 7.62 (ddt, *J* = 20.0, 6.4, 3.4 Hz, 6H), 7.32 – 7.18 (m, 4H), 7.12 (d, *J* = 7.6 Hz, 4H), 1.94 (s, 12H).

^{13}C NMR (126 MHz, DMSO-d$_6$) δ 158.88, 138.11, 135.92, 135.88, 132.93, 130.29, 130.10, 127.89, 127.73, 127.18, 127.14, 126.40, 126.09, 123.96, 20.34.

ESI-HR: calc'd for $C_{38}H_{36}N_3O_2^+$ ($[M+NH_4]^+$) 566.2802, found 566.2798, residual 0.71ppm

Compound L11:

***N*[1],*N*[2]-bis(2-(3,5-di-tert-butylphenyl)naphthalen-1-yl)oxalamide**

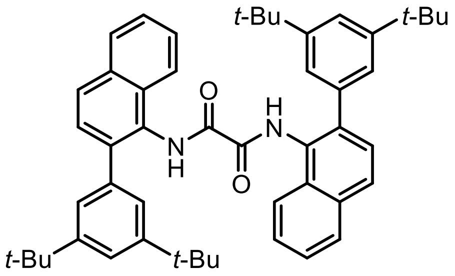

^{1}H NMR (500 MHz, DMSO-d$_6$) δ 10.67 (s, 2H), 8.02 (dd, *J* = 8.4, 3.3 Hz, 4H), 7.66 – 7.56 (m, 6H), 7.51 (ddd, *J* = 8.2, 6.8, 1.3 Hz, 2H), 7.45 (t, *J* = 1.8 Hz, 2H), 7.35 (d, *J* = 1.8 Hz, 4H), 1.31 (s, 36H).

^{13}C NMR (126 MHz, DMSO-d$_6$) δ 159.73, 149.92, 138.46, 138.12, 132.86, 130.38, 129.65, 128.37, 127.95, 127.73, 126.53, 126.08, 123.48, 123.23, 120.72, 34.61, 31.35.

ESI-HR: calc'd for $C_{50}H_{60}N_3O_2^+$ ([M+NH$_4$]$^+$) 566.2802, found 566.2798, residual 0.71ppm

Compound L6:

***N*¹,*N*²-bis(3-methyl-5'-phenyl-[1,1':3',1''-terphenyl]-2-yl)oxalamide**

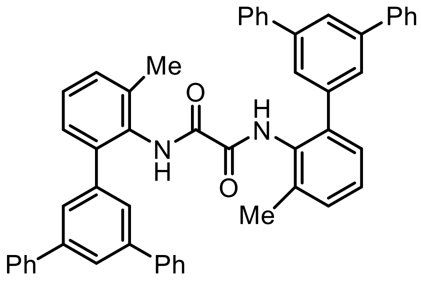

¹H NMR (500 MHz, DMSO-d6) δ 10.44 (s, 2H), 7.84 (t, *J* = 1.8 Hz, 2H), 7.82 – 7.73 (m, 8H), 7.61 (d, *J* = 1.7 Hz, 4H), 7.47 (dd, *J* = 8.3, 6.8 Hz, 8H), 7.43 – 7.35 (m, 6H), 7.31 (t, *J* = 7.5 Hz, 2H), 7.24 (dd, *J* = 7.6, 1.7 Hz, 2H), 1.85 (s, 6H).

¹³C NMR (126 MHz, DMSO-d6) δ 159.01, 140.65, 140.52, 140.04, 139.87, 136.29, 132.92, 129.56, 128.87, 127.88, 127.58, 127.53, 126.98, 126.15, 123.83, 17.61.

ESI-HR: calc'd for $C_{52}H_{40}N_2O_2Na^+$ ($[M+Na]^+$) 747.2982, found 747.3009, residual 3.61ppm

Synthesis of K_2-L3

Potassium oxalylbis((2-([1,1':3',1''-terphenyl]-5'-yl)naphthalen-1-yl)amide)

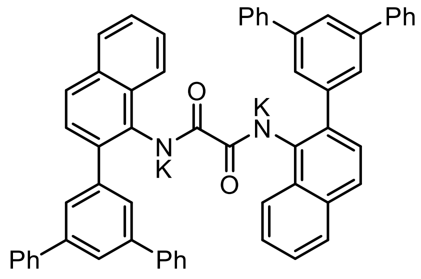

To a dry, 20-mL vial equipped with a stir bar was added **L3** (1.60 g, 2.00 mmol, 1.00 equiv) and potassium bis(trimethylsilyl)amide (KHMDS (95%), 798 mg, 4.00 mmol, 2.00 equiv). 15.0 mL of dry THF was added, and the reaction was stirred at room temperature for 24 h. After this time, the resulting white suspension was filtered through a fine porosity glass fritted funnel, and the filter cake was washed with dry pentane (30 mL*3). The collected solids were dried under high vacuum (50 mtorr) for 48 h to remove residual solvent, giving K_2-**L9** (1.57 g, 1.80 mmol, 90% yield) as a white solid.

^{1}H NMR (500 MHz, DMSO-d_6) δ 10.99 (s, 2H), 8.10 – 7.95 (m, 6H), 7.93 – 7.79 (m, 12H), 7.76 (d, *J* = 8.4 Hz, 2H), 7.48 (td, *J* = 7.9, 5.4 Hz, 12H), 7.44 – 7.34 (m, 4H), 7.13 (ddd, *J* = 8.4, 6.9, 1.3 Hz, 2H).

^{13}C NMR (126 MHz, DMSO-d_6) δ 159.86, 140.79, 140.59, 139.92, 137.00, 133.05, 130.28, 129.84, 128.92, 127.91, 127.65, 127.02, 126.66, 126.40, 126.20, 123.90, 123.08.

ESI-HR: calc'd for $C_{58}H_{40}N_2O_2Na^+$ ($[M+Na]^+$) 734.4680, found 734.4701, residual 2.86ppm

Synthesis of K_2-L12

Potassium oxalylbis((2-([1,1':3',1''-terphenyl]-5'-yl)naphthalen-1-yl)amide)

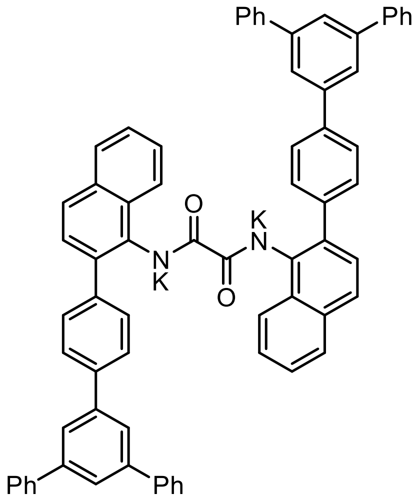

^{1}H NMR (500 MHz, DMSO-d_6) δ 8.31 – 8.27 (m, 2H), 8.05 – 7.99 (m, 4H), 7.91 – 7.82 (m, 10H), 7.81 – 7.70 (m, 12H), 7.55 – 7.48 (m, 10H), 7.46 – 7.40 (m, 4H), 7.31 (d, *J* = 8.4 Hz, 2H), 7.26 (dt, *J* = 5.9, 2.4 Hz, 2H).

^{13}C NMR (126 MHz, DMSO-d_6) δ 153.5, 142.4, 141.9, 141.5, 140.3, 136.7, 133.7, 130.1, 129.9, 128.9, 127.8, 127.6, 127.2, 127.1, 126.7, 126.4, 125.8, 124.5, 124.1, 124.0, 123.1, 117.5.

3. Evaluation of the effect of reaction components and conditions on yield

Example of procedure for small-scale reactions to evaluate reaction conditions

An oven-dried 4 mL glass vial was sequentially charged with ligand (10.0 µmol, 1.00 mol%), base (1.20 mmol, 1.20 equiv), 0.500 mL (10.0 µmol, 1.00 mol%) of a $CuBr_2$ stock solution in DMSO (or 0.500 mL, 10.0 µmol of other metal catalyst in DMSO), 1-chloro-4-methoxybenzene **1a** (123 µL, 143 mg, 1.00 mmol, 1.00 equiv), 1-hexylamine **2a** (198 µL, 152 mg, 1.50 mmol, 1.50 equiv) and a magnetic stir bar. The vial was capped, removed from the glovebox, placed in an aluminum heating block, heated to the indicated temperature (*vide infra*) and stirred for 24 h. After that time, 1,3,5-trimethoxybenzene (0.300 mmol, 33.6 mg) as an integration standard, dissolved in 1 mL DMSO, was added to the reaction mixture. An aliquot of the resulting solution was transferred into an NMR tube, diluted with $CDCl_3$, and the yield of the reaction was determined by ^{1}H NMR spectroscopy.

Table 1: Control Experiments

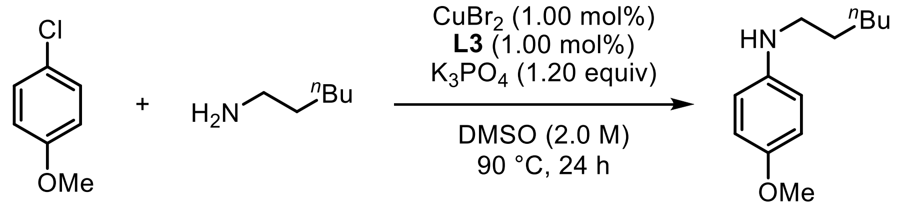

1a **2a** **3a**

Entry	Change	Product [%]
1	none	96
2	no $CuBr_2$	0
3	no **L3**	0
4	$Pd_2(dba)_3$ instead of $CuBr_2$	0
5	$Pd(acac)_2$ instead of $CuBr_2$	0

Table 2: Effect of Ligand

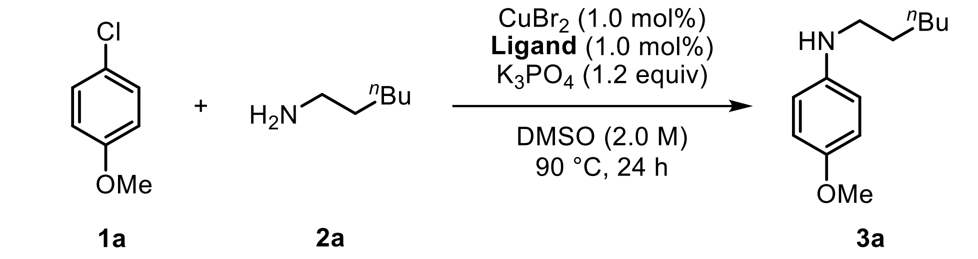

Entry	Ligand	Product [%]
1	**L1**	8
2	**L2**	9
3	**L3**	96
4	**L4**	62
5	**L5**	22
6	**L6**	68
7	**L7**	11
8	**L8**	7
9	**L9**	traces
10	**L10**	74
11	**L11**	95
12	**L12**	55

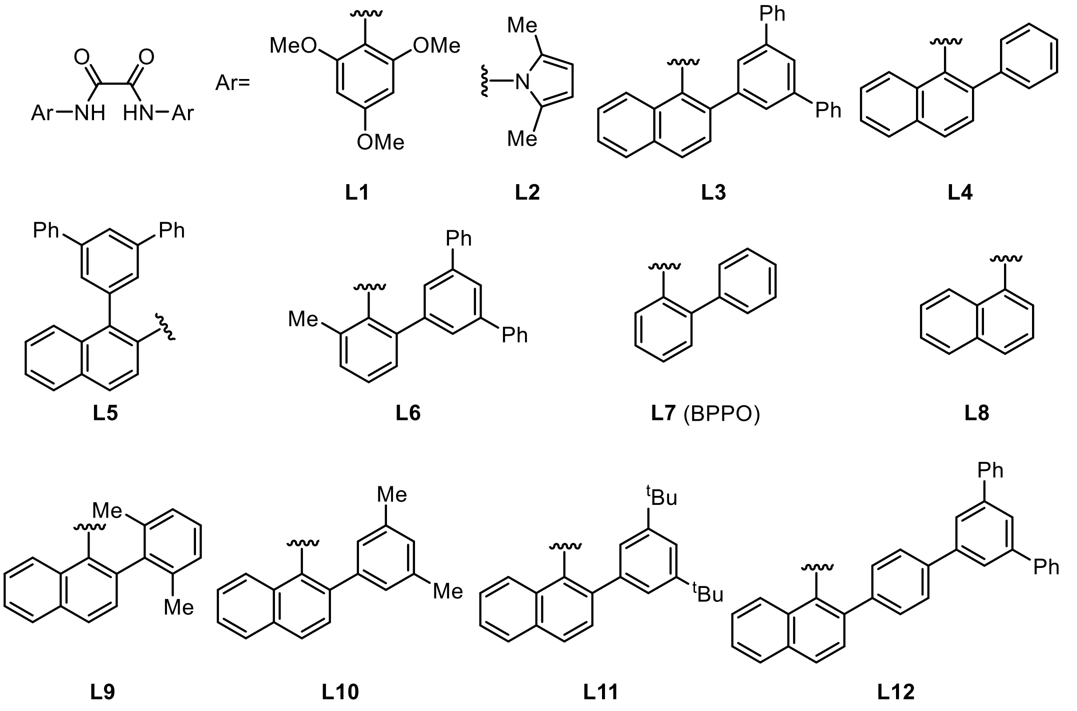

Table 3: Effect of Base

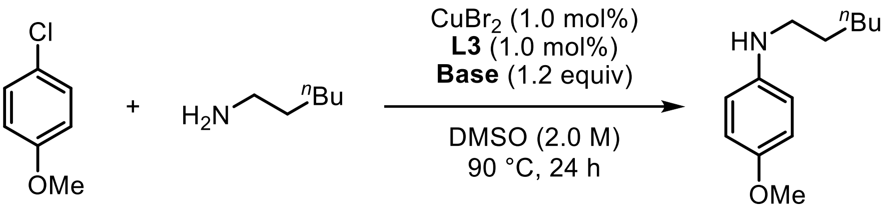

1a **2a** **3a**

Entry	Base	Product [%]
1	K_3PO_4	96
2	K_2CO_3	11
3	KOH	14
4	Cs_2CO_3	<5

Table 4: Effect of Solvent

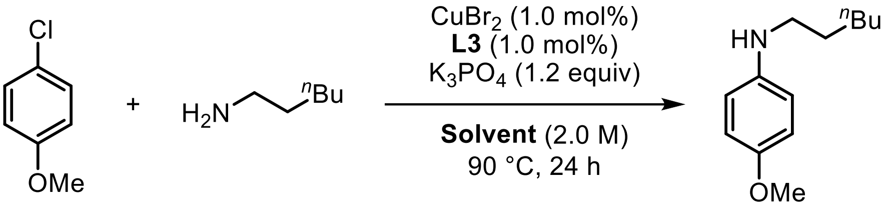

1a **2a** **3a**

Entry	Solvent	Product [%]
1	DMSO	96
2	DMF	22
3	PGME	21
4	NMP	66
5	DMA	73

Table 5: Effect of Temperature

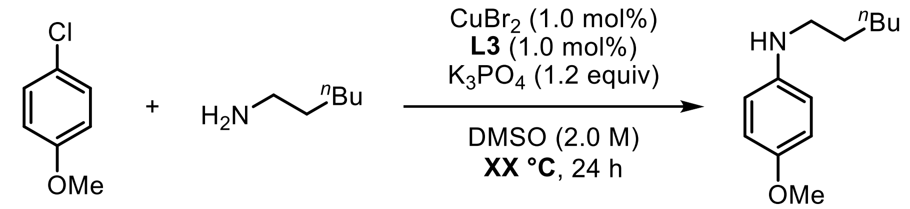

1a **2a** **3a**

Entry	Temperature	Product [%]
1	80 °C	51
2	90 °C	96
3	100 °C	97

Table 6: Effect of Cu Precatalyst

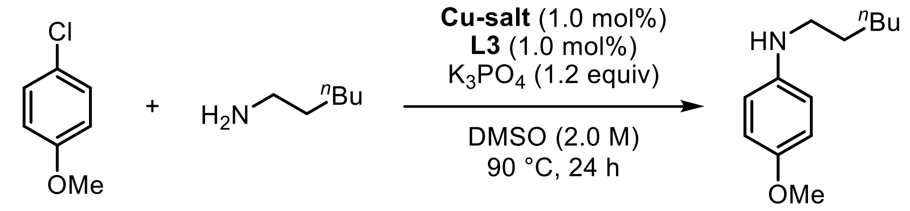

1a **2a** **3a**

Entry	Catalyst	Product [%]
1	CuBr	96
2	$CuBr_2$	96

4. EPR Spectroscopy Simulations

All three EPR spectra (**EPR-1**, **EPR-2**, and **EPR-3**) exhibit typical Cu^{2+} EPR signature with a d_{x2-y2} ground state. The simulation parameters are shown in Figure S1. Since **L3** is sterically congested, the Cu center in the Cu(**L3**)(NH_2R)$_2$ complex has a small out of plane distortion (see below for a DFT-optimized geometry), leading to a slightly rhombic *g*-tensor in **EPR-1** and **EPR-2**.

Simulation of the **EPR-2** trace indicates that there could be additional minor species in this sample, possibly introduced from minor impurities in the ^{15}N labeled substrate. The presence of these minor species should not affect our major conclusion because the ^{14}N/^{15}N isotopic response is clear and the majority of the spectral features are satisfactorily reproduced by simulation.”

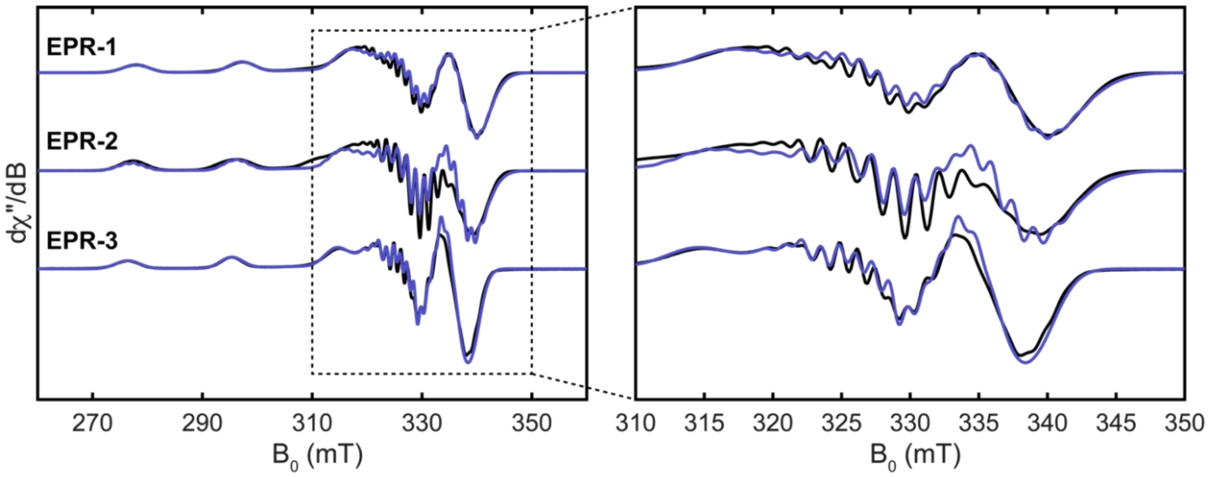

EPR-1:

g = [2.183, 2.050, 2.024];
Nucs = 'Cu, ^{14}N, ^{14}N, ^{14}N, ^{14}N';
A(^{63}Cu) = [575, 80, 30] MHz;
A($^{14}N1$) = [45, 42, 42] MHz;
A($^{14}N2$) = [45, 42, 42] MHz;
A($^{14}N3$) = [40, 35, 33] MHz;
A($^{14}N4$) = [40, 35, 33] MHz

EPR-2:

g = [2.183, 2.050, 2.024];
Nucs = 'Cu, ^{14}N, ^{14}N, ^{15}N, ^{15}N';
A(^{63}Cu) = [575, 80, 30] MHz;
A($^{14}N1$) = [45, 42, 42] MHz;
A($^{14}N2$) = [45, 42, 42] MHz;
A($^{15}N3$) = 1.406 × [40, 35, 33] MHz;
A($^{15}N4$) = 1.406 × [40, 35, 33] MHz

EPR-3:

g = [2.197, 2.042, 2.042];
Nucs = 'Cu, ^{14}N, ^{14}N';
A(^{63}Cu) = [572, 90, 30] MHz;
A($^{14}N1$) = [45, 40, 35] MHz;
A($^{14}N2$) = [45, 40, 35] MHz

Figure 1: Simulation parameters of the EPR spectra

Table 7: DFT-calculated EPR parameters for $CuL_3(NH_2Bn)_2$:

	DFT	Exp
***g*-tensor**	[2.12, 2.043, 2.024]	[2.183, 2.050, 2.024]
***A* (^{63}Cu) (MHz)**	[650, 89, 16]	[575, 80, 30]
***A* (^{14}N_amide) (MHz)**	[42, 29, 29]	[45, 42, 42]
***A* (^{14}N_amine) (MHz)**	[26, 17, 17]	[40, 35, 33]

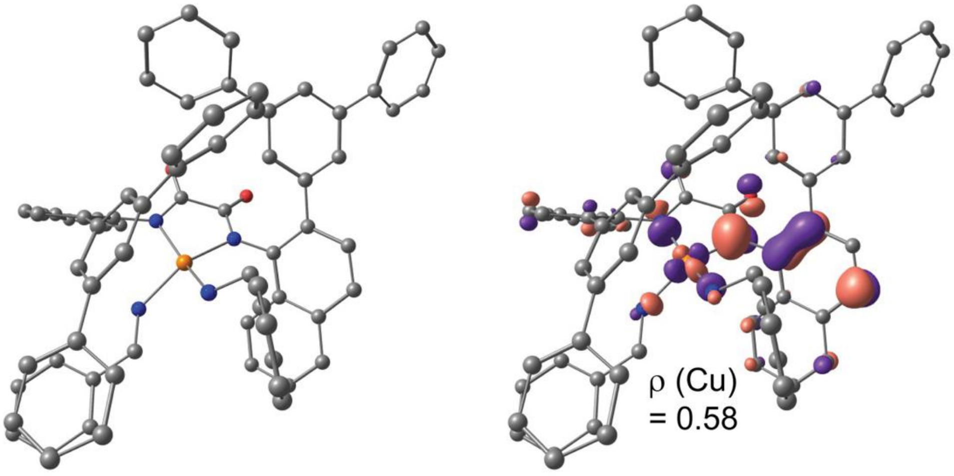

Figure 2. DFT-optimized geometry (left) and calculated SOMO (right) of $CuL_3(NH_2Bn)_2$. Isosurfaces indicate contour value of 0.05.

Computational details. Geometry optimization (GOPT) and calculation of EPR parameters were performed in ORCA 5.0.3.[2] The single-point energy and EPR parameters were calculated at DFT level using Unrestricted Kohn-Sham (UKS) formalism and employing the hybrid meta-GGA TPSSh wave functional along with the chain-of-sphere (RIJCOSX) approximation.[3] For GOPT, BP86 functional was used to accelerate the calculation.[4] Triple-zeta valence polarization def2-TZVP basis sets and the decontracted auxiliary basis sets def2/J coulomb-fitting were used for Cu, while the basis set of EPR-II was used for all light atoms (C, H, N, O).[3a, 5] The zero-order regular approximation (ZORA) was used to account for the scalar relativistic effects.[6] Tight SCF convergence were used throughout the calculation of all EPR parameters. The conductor-like polarizable continuum model (CPCM) was used to model the dielectric effects from the solvent DMSO (ε = 47) used for EPR samples. Dispersion correction was included in all calculations by using the D3BJ keyword in ORCA.[7]

Typical Input file for calculating EPR parameters:

```
! UKS TPSSH Zora EPR-II def2/J D3BJ decontractaux nofrozencore cpcm(DMSO)
! tightscf rijcosx slowconv

%basis
	NewGTO Cu "ZORA-def2-TZVP" end
end

%pal nprocs 8 end
%scf maxiter 500 end

%output
 PrintLevel=Normal
 Print[ P_Basis       ] 2
 Print[ P_MOs         ] 1
 Print[ P_Density     ] 1
 Print[ P_SpinDensity ] 1
end

* xyz 0 2
  C   -2.66602020148138    1.73862991920301   -0.69144961965329
  C   -2.47267980500765    1.19100470404496    0.74382598035475
  N   -3.38456430715484    0.91704245477764   -1.50330121055319
  O   -2.19883979067000    2.85353862580923   -0.98982697491215
  O   -1.67579113817757    1.74535964029592    1.51799247802536
  N   -3.23135340103000    0.09660061443254    1.00009833706070
  Cu  -4.20887798684858   -0.60180082603454   -0.58469698258755
  C   -3.40979529675077   -0.49490137230699    2.24703225086200
  C   -3.51966150142223    1.13882681595863   -2.87764374455980
  C   -3.27638280921027   -1.93926598891405    2.29782499999897
  C   -3.69949393766744   -2.66749484323019    3.46609684242364
  C   -4.14284550812181   -1.93274935807062    4.60468554551087
```

C	-4.17817857213890	-0.54851544316387	4.57048826226650
C	-3.84181041410474	0.20627804297092	3.40199779411048
C	-4.04582323958343	1.67982893315033	3.45704749010839
C	-4.78718038139059	1.03703938788544	-3.49874763700174
C	-4.90896964414297	1.26878946000675	-4.90220651060190
C	-3.81520096475567	1.59350692547102	-5.68635081088689
C	-2.50818512265845	1.61543257977544	-5.11598287717355
C	-2.34706761192763	1.34563903652311	-3.70731787192202
C	-6.00786603248022	0.55645827137626	-2.78372701044426
C	-6.58417207222977	-0.64401415780361	-3.25641328300611
C	-7.70368885115934	-1.22488217873929	-2.62550217071391
C	-8.32772018087799	-0.50401213543081	-1.58591653028335
C	-7.81381230178082	0.73092656220462	-1.13338552776793
C	-6.61462242479430	1.22705916739758	-1.69966441971327
C	-4.60773384090463	2.40584432421886	2.38459739351469
C	-4.87694717329714	3.78633990674872	2.49055713849673
C	-4.57512903705661	4.45120459622835	3.69810370667160
C	-3.99697655171947	3.76004710678701	4.78410810742175
C	-3.74097285033666	2.37829849199999	4.64592953202555
C	-1.35155360583927	1.85031843902493	-5.92067950839261
C	-0.07668293289954	1.77213643843469	-5.37723634214516
C	0.08629251283082	1.43618452785199	-4.00530262644497
C	-1.02176405257294	1.23056811396973	-3.19210820497515
C	-2.78090061996783	-2.67760348481044	1.18206667586884
C	-2.76988353907505	-4.06785800333958	1.18581551836364
C	-3.24293792913710	-4.78519672826168	2.31944940615265
C	-3.68218889383341	-4.09487259114387	3.44225497635644
N	-3.91241400179784	-1.91485845609403	-2.19489972033987
N	-5.73852882077235	-1.64281482041533	0.22565130164213
C	-6.45053238173369	-1.26023792148893	1.47223993029593
C	-7.63637149596761	-2.16932662120767	1.70814445575899
C	-4.64865697691646	-3.18518575763484	-2.26811261755175

C	-4.63302724405136	-3.90623014989081	-3.60219215227547
C	-8.95345559681408	-1.67160062659582	1.67189532188255
C	-10.05013560544455	-2.54404832499276	1.78364416634046
C	-9.83914197716932	-3.92534458606308	1.93545666099728
C	-8.52556577370349	-4.42893521363612	1.99392499817659
C	-7.43201742001589	-3.55580692869881	1.88512256180608
C	-5.10600666727220	-5.23455368839989	-3.65116213382170
C	-5.20702816572637	-5.91494052472937	-4.87257236032222
C	-4.83313850393981	-5.27686494251600	-6.07014320801228
C	-4.35268573527986	-3.95796962439613	-6.03032598338080
C	-4.25055364338170	-3.27775395638535	-4.80277433933894
C	-8.53408676819755	1.45858245545794	-0.05756413276215
C	-7.82657882168710	2.13433810856885	0.95900943880094
C	-8.49991867843133	2.73989709406998	2.02925953431883
C	-9.90330422204591	2.69923927572674	2.09273645789102
C	-10.62474670486940	2.05344435971989	1.07164774838470
C	-9.94800359766969	1.43632206541701	0.00844716124570
C	-8.14332158787238	-2.59941580122266	-2.97857110522911
C	-8.02751169384891	-3.09334346594059	-4.29852829499802
C	-8.38368734085516	-4.41552989826827	-4.60101082324884
C	-8.85276539648173	-5.27228185899893	-3.58957237599162
C	-8.96726597356543	-4.79502032636838	-2.27175001056411
C	-8.61897144100680	-3.47009453832752	-1.96773518347609
C	-3.66075540502728	4.47102565038695	6.04660826681452
C	-4.45412980213197	5.55073509286204	6.50369796658770
C	-4.14140740369774	6.22066435551199	7.69676232458904
C	-3.02796286356420	5.82656253547825	8.46028329805566
C	-2.23027458505090	4.75597944544958	8.01823856044981
C	-2.54301547083004	4.08594163605487	6.82513187344371
C	-5.48479036673802	4.52004106307654	1.34946030547684
C	-5.07239924656063	4.25835034458911	0.02276852261706
C	-5.70232458226839	4.88784556489720	-1.06220999023765

C	-6.75395886924062	5.79482612398133	-0.84147847965232
C	-7.15159497332293	6.08687841110770	0.47579965558788
C	-6.52049287361085	5.45905744232791	1.56093049421540
H	-4.47079588603286	-2.47782532196166	5.50296081478297
H	-4.54624188807495	-0.00281491453017	5.45200038745212
H	-5.91045545207104	1.20205681126341	-5.35404047591053
H	-3.93632223525263	1.80180117774358	-6.76047821995546
H	-6.10108661666113	-1.17471750599632	-4.09024530913845
H	-9.22154338163637	-0.92256863422426	-1.10279217723560
H	-6.18643767884579	2.17192382867482	-1.33770359180219
H	-4.86531488634737	1.87631522121259	1.45887476445710
H	-4.74786530768781	5.53511077118781	3.77176869489096
H	-3.30376919622873	1.82901059937654	5.49226396228425
H	-1.49386842628531	2.07482628806768	-6.98961526023474
H	0.80780010791170	1.94756712031872	-6.00885759094935
H	1.09835122037472	1.33228718817608	-3.58458504093072
H	-0.87522272972749	0.95646583837031	-2.13972137959633
H	-2.39588997705579	-2.12412334693254	0.31308817202984
H	-2.38448763602890	-4.61460662321263	0.31165266414226
H	-3.24383983710213	-5.88593888919868	2.31053231523704
H	-4.02876031694696	-4.63909520509435	4.33486830538490
H	-2.89647493361530	-2.08776447228195	-2.22648592087162
H	-4.10925728751747	-1.32758075601317	-3.01825548734843
H	-5.42277125191313	-2.62097709114980	0.31346033119751
H	-6.44147114780727	-1.65148895300737	-0.53259429560141
H	-5.73319117277557	-1.31878497436041	2.30996972270270
H	-6.76976555526339	-0.20659152818749	1.38243338056799
H	-4.26842876912702	-3.85119162053010	-1.46716968966782
H	-5.71321717037274	-2.98928562856241	-2.02501803406513
H	-9.12032625595046	-0.59240926570724	1.53858557515691
H	-11.07395615023445	-2.14118263269642	1.74289880786063
H	-10.69690648228501	-4.61111295898583	2.01263298340396

```
H  -8.35375273803091    -5.50843942453396    2.12536413501795
H  -6.40491947135307    -3.95563315032092    1.93377671944226
H  -5.41629733908292    -5.73131190782143   -2.71792022678072
H  -5.58616960842843    -6.94836274185599   -4.89264628059717
H  -4.91505191199716    -5.80834559157415   -7.03072642671774
H  -4.05266632109790    -3.44906909901568   -6.95941950487886
H  -3.86585926163059    -2.24595289478214   -4.79840328155019
H  -6.73153342234424     2.15921127346578    0.92526489655616
H  -7.91979479584140     3.23717042752527    2.82070488648352
H  -10.43452869119741    3.17229731624801    2.93281463982475
H  -11.72493758502121    2.02873251241727    1.10381446392756
H  -10.52211708124606    0.93667791895555   -0.78646315137639
H  -7.66457392056545    -2.43175207404565   -5.09928436655569
H  -8.28622569908064    -4.78184843536782   -5.63396575972756
H  -9.12119402293557    -6.31315279527205   -3.82673197792742
H  -9.32215583552916    -5.45975896824765   -1.46912296573037
H  -8.68620050390328    -3.11500282479042   -0.92757161021000
H  -5.34184833590030     5.85426705363578    5.92807655013557
H  -4.77782658477155     7.05229210050159    8.03721347028715
H  -2.78272201876314     6.35171861207976    9.39631715886177
H  -1.35118338824375     4.44376922160214    8.60326962165078
H  -1.89656852900201     3.26475657591663    6.47960668277218
H  -4.22330079421862     3.58629551552953   -0.16066894347477
H  -5.36209736591303     4.66934257249019   -2.08649100380671
H  -7.25565568392027     6.28101091919968   -1.69235946843381
H  -7.97052780410528     6.79949396251123    0.65981055675765
H  -6.86026429537078     5.66998043608009    2.58654211177614
*
%eprnmr gtensor true printlevel 3 end
%eprnmr

    nuclei = all Cu  aiso, adip
```

```
nuclei = all N   aiso, adip
end
```

5. Quantification of Cu(I) Complex in Catalytic Reaction over Time

General Remark

All reactions were assembled in a nitrogen filled glove box. Due to the heterogeneity of the reaction, each time point represents an individually assembled reaction. 1,3,5-trimethoxybenzene was added to each reaction to serve as ^{1}H NMR internal standard. All NMR samples were prepared in a nitrogen filled glove box.

Quantification of Cu(I) complex in the catalytic reaction with $CuBr_2$ as precursor

Twelve 4 mL scintillation vials were charged K_3PO_4 (256 mg, 1.20 mmol, 1.20 equiv) and a Teflon-coated stir bar.

Next, a stock solution was prepared which contained $CuBr_2$ and K_2-**L3** in DMSO-d_6. To a dry, 20 mL vial was added $CuBr_2$ (223 mg, 1.00 mmol), K_2-**L3** (873 mg, 1.00 mmol), dry DMSO-d_6 (10.0 mL), and a stir bar. The reaction was stirred at room temperature for 30 min, then filtered through a PTFE syringe filter (0.22 µm pore size) into a fresh 20 mL vial. The reaction vial was rinsed 3 times with a total of 10.0 mL DMSO-d_6 to give 1.00 mmol of Cu(II) catalyst in a total of 20.0 mL DMSO.

Each 4 mL scintillation vial which already contained K_3PO_4 and a Teflon-coated stir bar was sequentially charged with 1.00 mL of the Cu(II) catalyst stock solution (50.0 µmol, 5.00 mol%), *n*-hexylamine (198 µL, 152 mg, 1.50 mmol, 1.50 equiv) 1-chloro-4-methoxybenzene (123 µL, 143 mg, 1.00 mmol, 1.00 equiv) and 1,3,5-trimethoxybenzene (16.2 mg, 0.100 mmol,0.100 equiv). The vials were sealed with Teflon-lined caps, and the reactions were stirred at 80 °C for 5 min, 10 min, 15 min, 20 min, 30 min, 40 min, 50 min, 60 min, 70 min, 80 min, 90 min, 100 min. After these times, the vials were returned to the glove box. After cooling to room temperature, the reaction mixtures were filtered through a PTFE syringe filter (0.22 µm pore size) into an NMR tube. The NMR tubes were sealed tightly with parafilm before removing them from the glove box. The yields of **3a** and the amount of Cu(I) complex were subsequently determined by ^{1}H NMR spectroscopy.

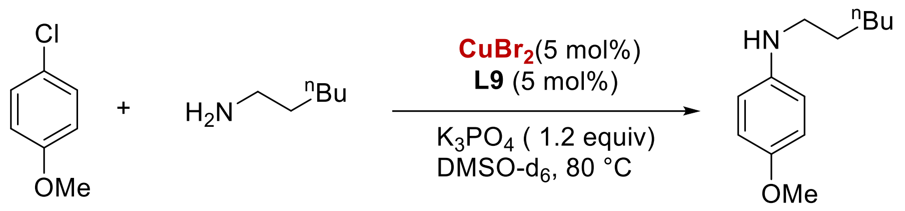

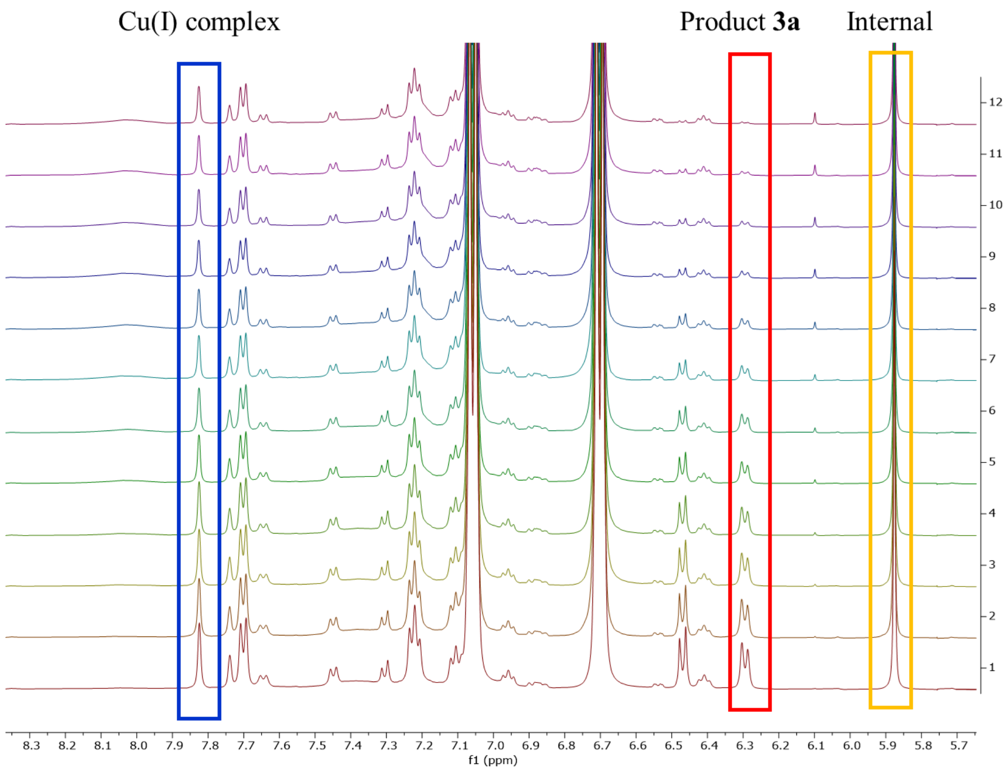

Figure 3: ^{1}H NMR spectra of each recorded time point with $CuBr_2$ as catalyst precursor. Signals corresponding to Cu(I) complex **Cu-B** (blue), product **3a** (red) and internal standard (yellow) are highlighted.

Table 8: Amount of Cu(I)-complex **Cu-B** and Product **3a** formed during the time course.

Entry	Time [min]	Cu(I)-complex [mM]	Product **3a** [mM]
1	5	15.00	4.50
2	10	15.00	6.75
3	15	15.00	9.00
4	20	16.20	11.25
5	30	15.75	17.25
6	40	18.00	21.75
7	50	16.50	27.00
8	60	19.20	33.00

9	70	19.50	39.00
10	80	21.00	45.75
11	90	22.50	51.75
12	100	23.25	58.50

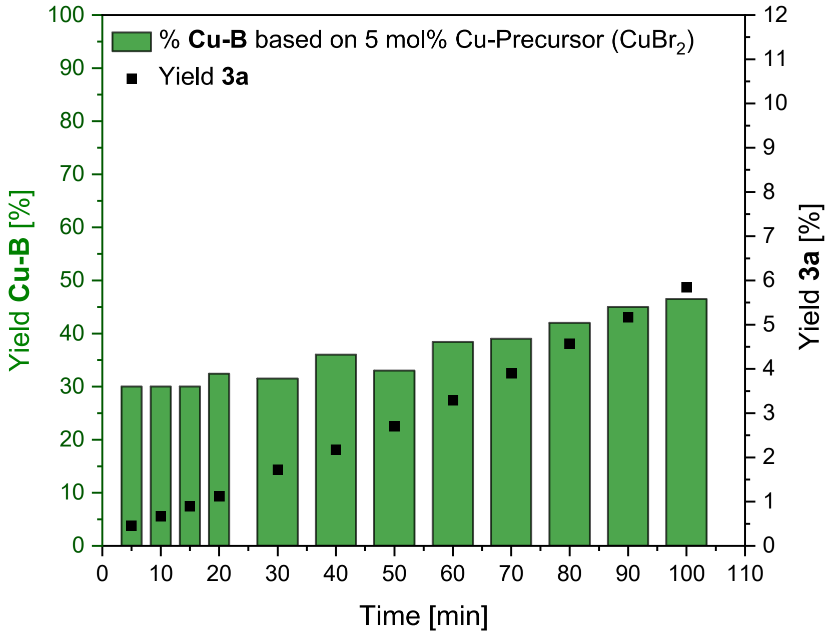

Figure 4: Graphical representation of the amount of **Cu-B** and **3a** over time with $CuBr_2$ as catalyst precursor.

Quantification of Cu(I) complex in the catalytic reaction with CuBr as precursor

Twelve 4 mL scintillation vials were charged K_3PO_4 (256 mg, 1.2 mmol, 1.2 equiv) and a Teflon-coated stir bar.

Next, a stock solution was prepared that contained CuBr and K_2-**L3** in DMSO-d_6. To a dry, 20 mL vial was added CuBr (143 mg, 1.00 mmol), K_2-**L3** (873 mg, 1.00 mmol), dry DMSO-d_6 (10.0 mL), and a stir bar. The reaction was stirred at room temperature for 30 min, then filtered through a PTFE syringe filter (0.22 µm pore size) into a fresh 20 mL vial. The reaction vial was rinsed 3 times with a total of 10.0 mL DMSO-d_6 to give 1.00 mmol of Cu(I) catalyst in a total of 20.0 mL DMSO.

Each 4 mL scintillation vial which already contained K_3PO_4 and a Teflon-coated stir bar was sequentially charged with 1.00 mL of the Cu(I) catalyst stock solution (50.0 µmol, 5.00 mol%), *n*-hexylamine (198 µL, 152 mg, 1.50 mmol, 1.50 equiv)

1-chloro-4-methoxybenzene (123 μL, 143 mg, 1.00 mmol, 1.00 equiv) and 1,3,5-trimethoxybenzene (16.2 mg, 0.100 mmol,0.100 equiv). The vials were sealed with Teflon-lined caps, and the reaction was stirred at 80 °C for 5 min, 10 min, 15 min, 20 min, 25 min, 30 min, 35 min, 40 min, 45 min, 50 min, 55 min, 60 min. After these times, the vials were returned to the glove box. After cooling to room temperature, the reaction mixture was filtered through a PTFE syringe filter (0.22 μm pore size) into an NMR tube. The NMR tubes were sealed tightly before removing it from the glove box. The yields of **3a** and the amount of Cu(I) complex were subsequently determined by ^{1}H NMR spectroscopy.

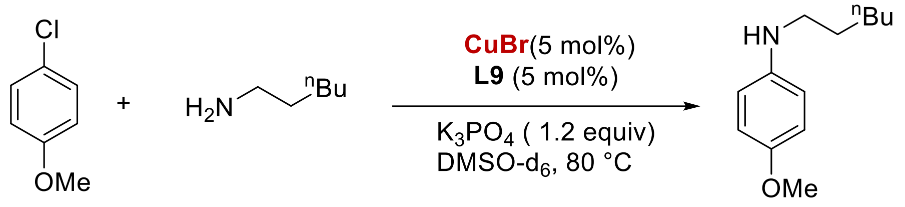

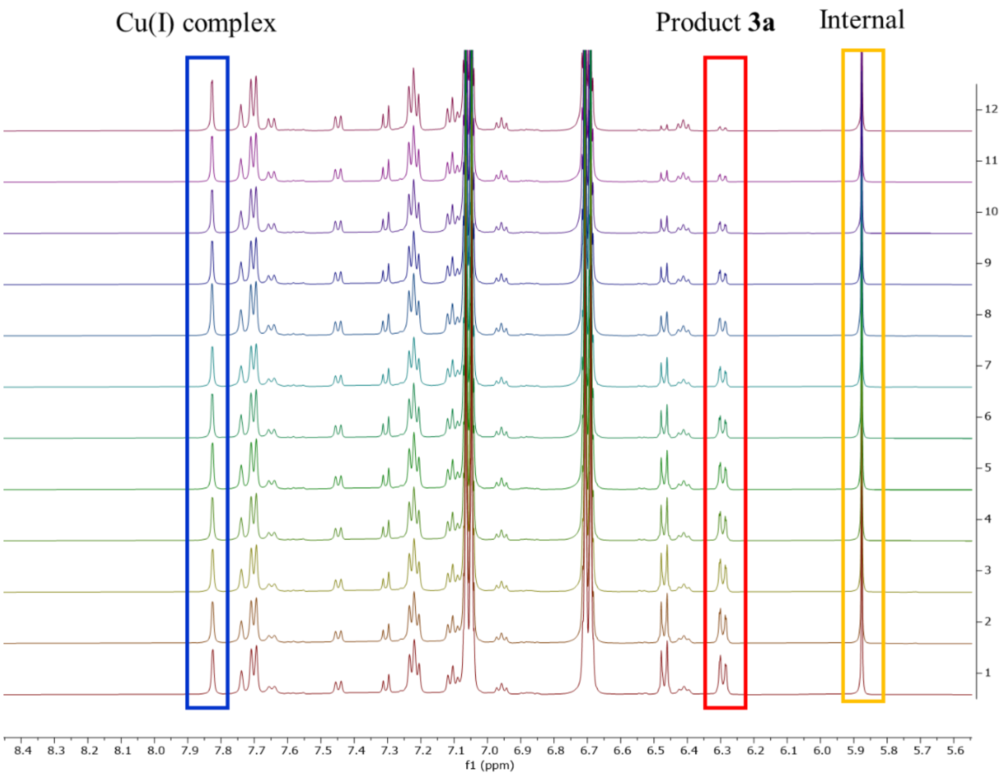

Figure 5: ^{1}H NMR spectra of each recorded time point with CuBr as catalyst precursor. Signals corresponding to Cu(I) complex **Cu-B** (blue), product **3a** (red) and internal standard (yellow) are highlighted.

Table 9: Amount of Cu(I)-complex **Cu-B** and Product **3a** formed during the time course.

Entry	Time [min]	Cu(I)-complex [mM]	Product **3a** [mM]
1	5	42.00	16.50
2	10	40.50	27.00
3	15	39.00	37.50
4	20	40.50	45.00
5	25	39.00	52.50
6	30	40.50	61.50
7	35	40.50	69.00
8	40	42.00	79.50
9	45	40.50	88.50
10	50	41.25	96.00
11	55	39.75	103.50
12	60	39.00	108.00

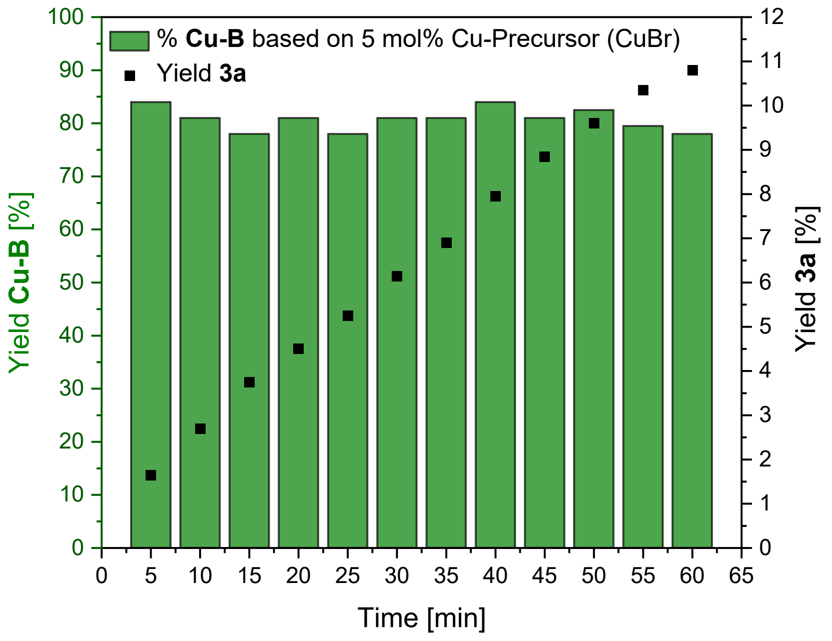

Figure 6: Graphical representation of the amount of **Cu-B** and **3a** over time with $CuBr_2$ as catalyst precursor.

6. Characterization of Cu-Species in Catalytic Reaction via ^{1}H NMR and EPR Spectroscopy

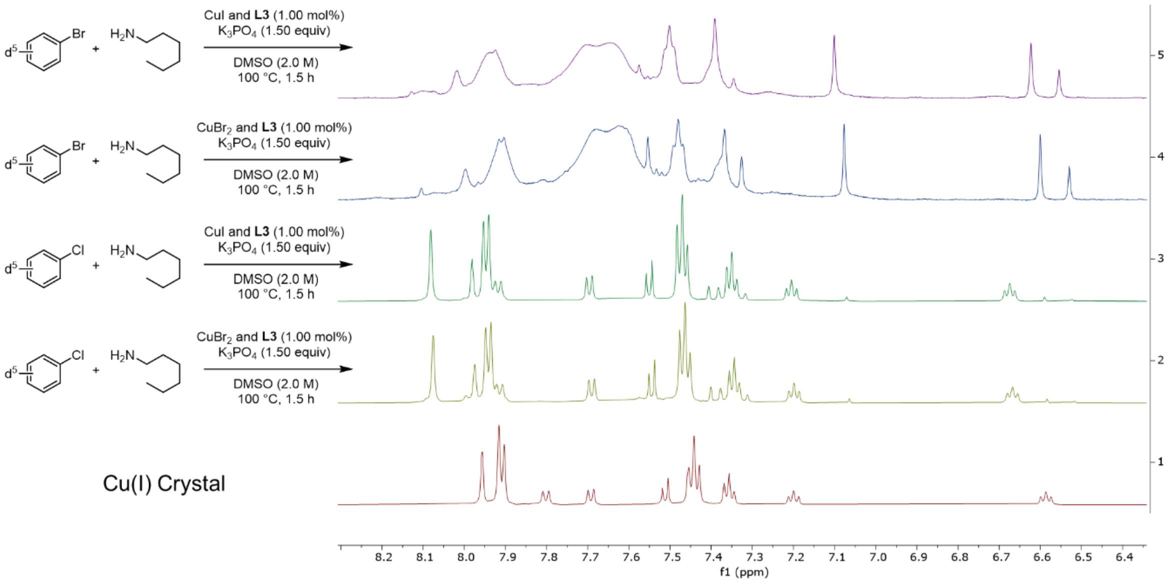

Figure 7: ^{1}H NMR spectra of the C-N coupling between d$_5$-PhBr or d$_5$-PhCl with *n*-hexylamine employing CuI or $CuBr_2$ as precatalyst. The 1H NMR spectrum of Cu(I) crystal **Cu-B** is overlayed as reference (bottom).

To determine the Cu-species during the coupling of aryl bromides vs. the coupling of aryl chlorides we conducted 4 separate reactions: The coupling of PhBr-d_5 with hexylamine in the presence of a CuI or $CuBr_2$ and the coupling PhCl-d_5 with hexylamine in the presence of a CuI or $CuBr_2$.

Procedure for the coupling of d$_5$-PhBr or d$_5$-PhCl with hexylamine

First a CuI and a $CuBr_2$ stock solution were prepared. For the CuI stock solution, a 4 mL vial was charged with CuI (9.5 mg, 50 µmol) and 1.00 mL d$_6$-DMSO. The mixture was stirred for 30 min at room temperature. For the $CuBr_2$ stock solution, a 4 mL vial was charged with $CuBr_2$ (11.2 mg, 50.0 µmol) and 1.00 mL d$_6$-DMSO. The mixture was stirred for 30 min at room temperature.

A 4 mL scintillation vial was sequentially charged with **L3** (8.0 mg, 10 µmol), K_3PO_4 (318 mg, 1.50 mmol, 1.50 equiv), 300 µL DMSO-d_6, *n*-hexylamine (198 µL, 152 mg, 1.50 mmol, 1.50 equiv) and PhBr-d_5 (105 µL, 162 mg, 1.00 mmol 1.00 equiv) or PhCl-

*d*5 (102 µL, 118 mg, 1.00 mmol, 1.00 equiv) and a stir bar. Then, 200 µL of the respective Cu-stock solution (10 µmol, 1.0 mol%) were added. The vials were sealed, taken out of the glove box, further sealed with electrical tape and heated to 100 °C for 1.5 h. After this time, the reactions were allowed to cool to room temperature, the electrical tape was removed, and the reactions were returned to the glove box. There, 350 µL DMSO-*d*6 were added to each vial, sealed, and vigorously shaken for 10 s. The reaction was allowed to stand for 5 min for the solid to settle. Then, 0.600 mL of the supernatant were transferred into an NMR tube, which was subsequently sealed with parafilm. ^{1}H NMR spectroscopy was recorded for each reaction, resulting in the spectra shown in Figure 7.

To verify that the species in the reaction of PhCl-*d*5 is the Cu complex **Cu-B**, we dissolved crystals of **Cu-B** in DMSO-*d*6, recorded a ^{1}H NMR spectrum, and overlayed the spectra. We noticed that the chemical shifts of the Cu(I) complex are slightly shifted from those of the species obtained from the coupling reaction of PhCl-*d*5 with *n*-hexylamine. Consequently, we added 7.1 mg of the **Cu-B** crystals to the NMR sample containing the $CuBr_2$ catalyzed reaction between PhCl-*d*5 and *n*-hexylamine. Spiking this reaction sample did not result in a new set of signals; instead the signals from the reaction shifted closer to signals of the pure **Cu-B** compound. This experiment provides strong evidence that $CuBr_2$ is reduced to **Cu-B** under reaction conditions.

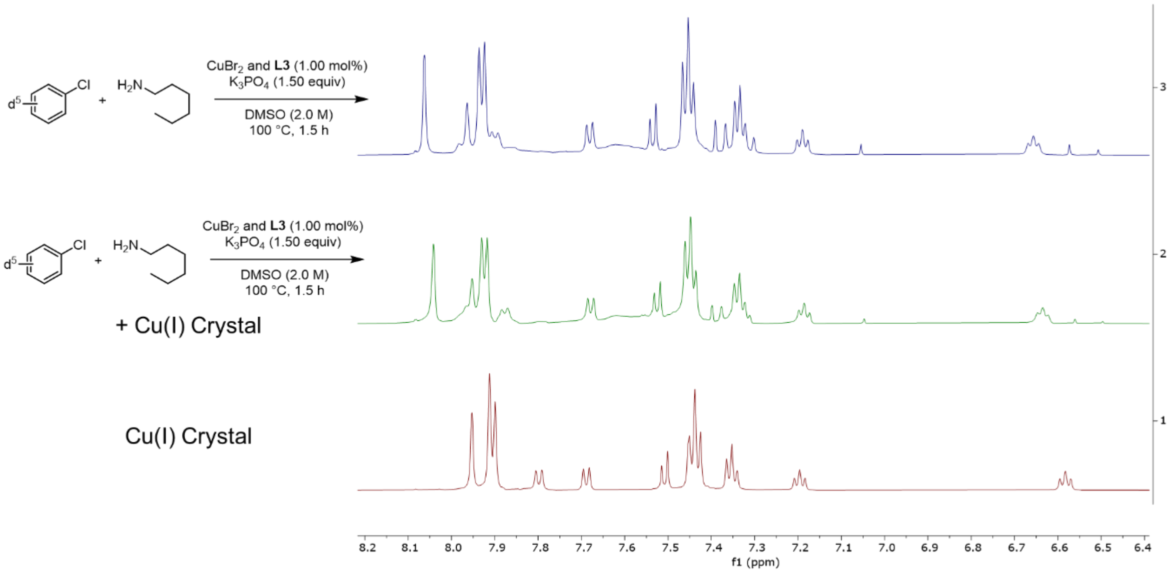

Figure 8: Coupling of d5-PhCl with *n*-hexylamine and $CuBr_2$ as precatalyst (top). Top NMR sample spiked with **Cu-B** (middle) and ^{1}H NMR spectrum of **Cu-B**.

In section 5 (vide supra) we demonstrated that the coupling reactions of the aryl chloride conducted with a Cu(I) precatalyst (e.g. CuBr) contain some amounts of Cu(II), presumably **Cu-A**. To further verify this finding, we conducted quantitative EPR spectroscopy on the four reactions presented in this section.

EPR sample preparation

Each reaction was assembled again according to the procedure described above for the coupling of PhBr-d_5 or PhCl-d_5 with hexylamine, except DMSO was used instead of DMSO-d_6. After the reactions, the 4 mL reaction vials were transferred into a nitrogen filled glove box. A total of 10.0 µmol of one or multiple Cu-species is present in each reaction. The total volume of each reaction includes the liquid reagents (*n*-hexylamine 198 µL, PhCl-d_5 101.6 µL or PhBr-d_5 105.3 µL) and the solvent (DMSO, 500 µL). Consequently, the Cu concentration in all four reactions is 12.0 mM. For quantitative EPR spectroscopy a Cu concentration below 5 mM should be used. Consequently, we transferred 200 µL of each reaction mixtures into a separate 4 mL scintillation vial and added 400 µL DMSO. This procedure results in 2.47 µmol Cu being dissolved in 600 µL solvent and a theoretical maximum Cu(II) concentration of 4.01 mM. 250 µL of this stock solution were transferred into an EPR tube and tightly sealed with parafilm.

The EPR spectra were recorded at 77 K. To determine the P1/2 value of each sample, a power sweep was conducted prior to Cu(II) quantification. The P1/2 value was > 7 mW which allowed us to measure the sample at 5 mW with a modulation of 0.6 mT. The sweep time was set to 60 s, the field spanned 200-450 mT, and the number of accumulations was set to 5. Quantification was conducted with the internal EPR software from Bruker. Area for baseline correction: 255-270 mT and 360-375 mT. Area for quantification: 270-360 mT. The spectra below show the spectral width from 255-375 mT

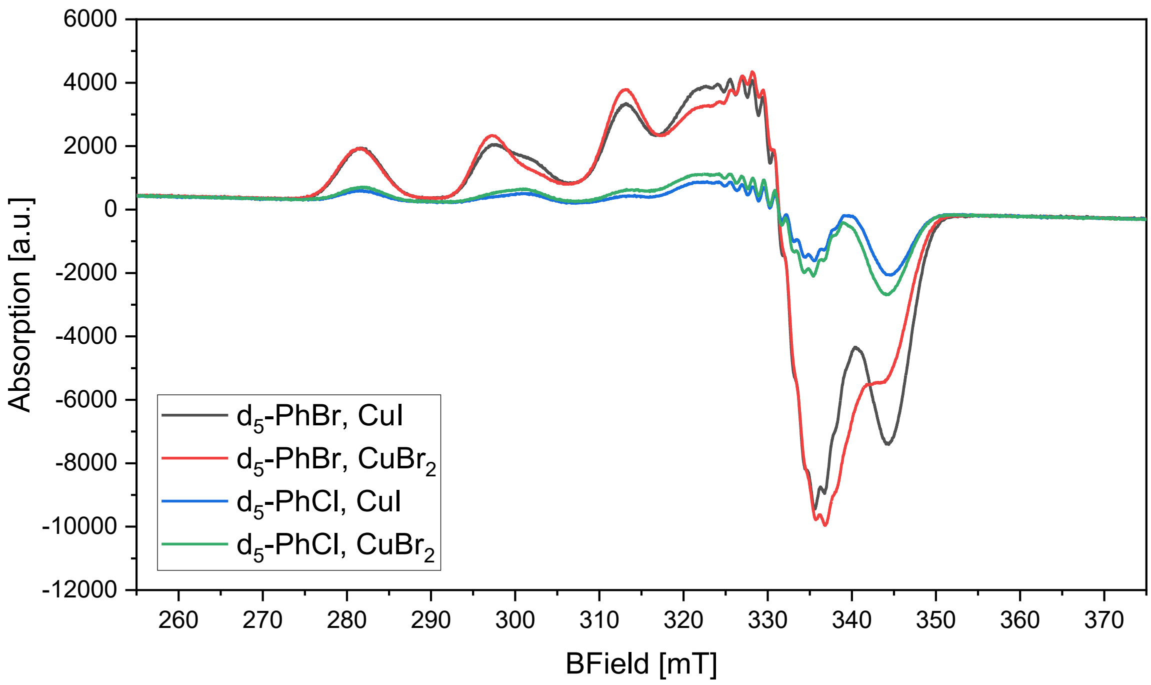

Figure 9: EPR spectra of the four coupling reactions

Table 10: EPR quantification of Cu(II) in the reaction. Note that the maximum theoretical concentration is 4.01 mM (*vide supra*).

**Experiment**	**EPR Quantification of Cu(II)**	**% of Cu(II) in the reaction**
PhBr-d_5, CuI	4.22 mM	105 %
PhBr-d_5, $CuBr_2$	3.63 mM	90.5%
PhCl-d_5, CuI	0.651 mM	16.2 %
PhCl-d_5, $CuBr_2$	0.852 mM	21.2 %

We conclude that the major Cu-species in the reactions with PhBr-d_5 contains a Cu(II) center, independent of the Cu precatalyst. However, in the reactions of PhCl-d_5, only ≈20% of the Cu in solution is Cu(II). The remaining species are likely Cu(I). This matches the Cu(I) quantification in section 5 in which 80% of the Cu in the reaction

corresponded to Cu(I) if CuBr is used as pre-catalyst. It is likely that with $CuBr_2$ as precatalyst, after enough time, the same level of Cu(I) will form.

7. Imine Formation from Cu(II) Reduction

To assess whether the amine in the reaction acts as reducing agent for Cu(II) to form the catalytically active Cu(I) species, we conducted the following experiment.

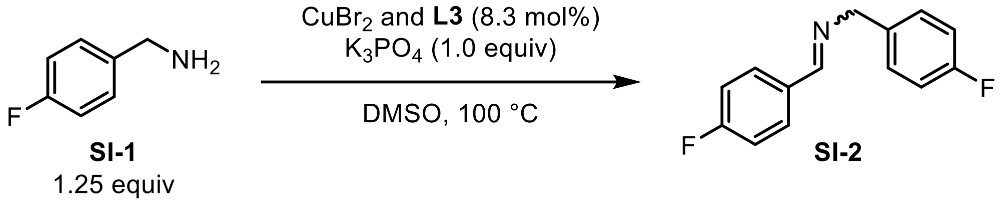

In a nitrogen filled glove box, a 4 mL vial was sequentially charged with 22.3 mg $CuBr_2$ (100 µmol, 8.30 mol%), 79.7 mg **L3** (100 µmol, 8.30 mol%), 255 mg K_3PO_4 (1.20 mmol, 1.00 equiv), 500 µL DMSO-d_6, 171 µL (4-fluorophenyl)methanamine **SI-1** (188 mg, 1.50 mmol, 1.25 equiv), and a stir bar. The vial was capped, removed from the glove box, sealed with electrical tape, stirred for 1 min at room temperature and 1.5 h at 100 °C. The vial was brought back into the glove box and 0.500 mL $CDCl_3$ were added. After vigorously shaking the vial, the K_3PO_4 was allowed to settle. After 5 min, 100 µL of the resulting reaction mixture were transferred into an NMR tube and 0.500 mL $CDCl_3$ were added. In the ^{19}F NMR spectrum imine **SI-2** was identified which matched the chemical shifts reported in the literature.[8]

8. Initial Rates for Coupling of 4-Chloroanisole with *n*-Hexylamine

General Remark

All reactions were assembled in a nitrogen filled glove box. The reaction order in aryl chloride, amine, and Cu-catalyst was determined on the basis of the C–N coupling between 4-chloroanisole and *n*-hexylamine. Due to the heterogeneity of the reaction, each time point represents an individually assembled reaction. 1,3,5-Mesitylene was added to each vial after the reaction to serve as NMR internal standard. NMR samples were prepared as follows: For each time point, the reaction vial was opened and 28 µL of 1,3,5-mesitylene was added, followed by 500 µL $CDCl_3$. The vial was resealed, vigorously shaken and the K3PO4 was allowed to settle. 100 µL of the resulting solution were transferred into an NMR tube, followed by 500 µL $CDCl_3$. Linear regression of product concentration against reaction time gave the initial reaction rate under each respective concentration of reagent (*vide infra*).

Preparation of Cu-B complex stock solution

A 20 mL scintillation vial was sequentially charged with 89.6 mg CuBr (0.625 mmol, 1.00 equiv), 498 mg **L3** (0.625 mmol, 1.00 equiv), 3.98 g K_3PO_4 (18.7 mmol, 30 equiv), 6.25 mL DMSO, 2.48 mL hexylamine (1.90 g, 18.7 mmol, 30 equiv), and a stir bar. The vial was capped, removed from the glove box, sealed with electrical tape and stirred at 100 °C for 1.5 h during which the solution turned orange. After this time the reaction was allowed to cool to room temperature, the electrical tape was removed and the vial was reintroduced into the glove box. The reaction was subsequently filtered via vacuum filtration. Note: The vacuum was applied gently to avoid evaporation of hexylamine an modification of the catalyst concentration. The filter cake was not washed and the filtrate was transferred into a fresh 20 mL vial. This **Cu-B** stock solution was used to determine the reaction order in 4-chloroansiole, hexylamine, and Cu/L3.

Note: To ensure the freshness of the stock solution, all initial rates for the reaction orders were obtained within 24 hours. It needs to be considered that excess hexylamine is in the stock solution. 69.8 µL of the prepared stock solution contains 5.00 µmol Cu/**L3** (corresponds to 1 mol% when the reaction is set up on 0.500 mmol scale) dissolved in 50.0 µL DMSO and 19.8 µL hexylamine.

Reaction Order in 4-chloroanisole

The initial rates were determined for reactions with five different aryl chloride concentrations: 1.00 M, 1.50 M, 2.50 M, 3.00 M, and 3.50 M. To do so, 20 scintillation vials (4 mL size, four per ArCl concentration) were charged with K_3PO_4 (127 mg, 0.750 mmol). Next, 200 µL DMSO were added to each reaction vial, followed by variable amounts of 4-chloroansiole (0.250 mmol, 0.375 mmol, 0.625 mmol, 0.750 mmol, and 0.875 mmol), 79.3 µL hexylamine, 69.8 µL of the **Cu-B** stock solution, and a stir bar. The reactions were removed from the glove box, sealed with electrical tape, stirred for 1 min at room temperature and subsequently stirred for 30, 60, 90, and 120 min. After this time, NMR samples were prepared of each reaction vial (*vide supra*) to determine the yield in the C–N cross-coupled product. The initial rate for each amount of 4-chloroanisole (linear regime of product formation) was plotted against the respective concentration., resulting in a 1st order for 4-chloroanisole.

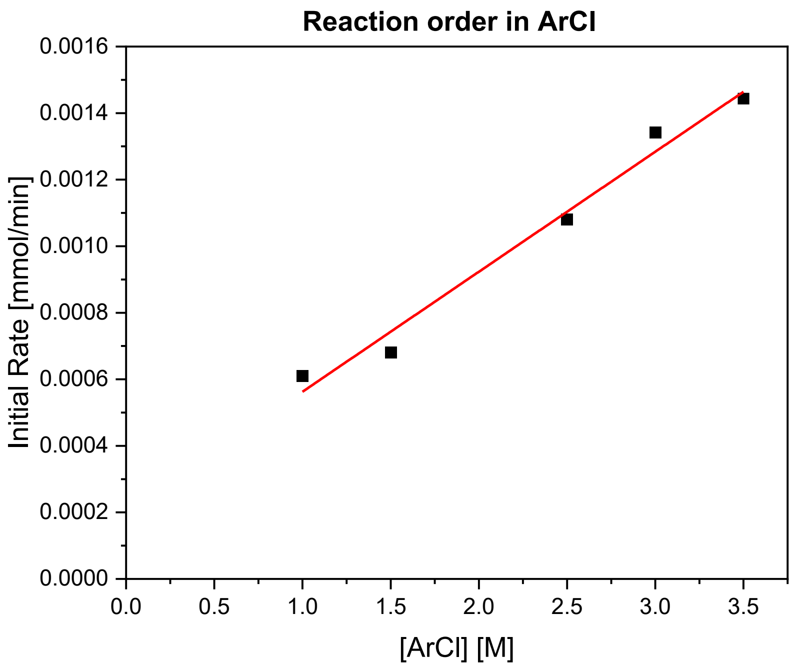

Figure 10: Reaction order in ArCl

Reaction order in *n*-hexylamine

The initial rates were determined for reactions with five different amine concentrations: 1.20 M, 1.80 M, 2.40 M, 3.00 M, and 3.60 M. To do so, 20 scintillation vials (4 mL size, four per *n*-hexylamine concentration) were charged with K_3PO_4 (127 mg, 0.750 mmol). Next, 200 µL DMSO were added to each reaction vial, followed by variable amounts of *n*-hexylamine (0.150 mmol, 0.300 mmol, 0.450 mmol, 0.600 mmol, and 0.750 mmol; note that the 69.8 µL of Cu-B stock contain additional 0.150 mmol *n*-hexylamine), 61.3 µL 4-chloroansiole (71.3 mg, 0.500 mmol), 69.8 µL of the **Cu-B** stock solution, and a stir bar. The reactions were removed from the glove box, sealed with electrical tape, stirred for 1 min at room temperature and subsequently stirred for 30, 60, 90, and 120 min. After this time, NMR samples were prepared of each reaction vial (*vide supra*) to determine the yield in the C–N cross-coupled product. The initial rate for each amount of *n*-hexylamine (linear regime of product formation) was plotted against the respective concentration., resulting in a 0th order for *n*-hexylamine.

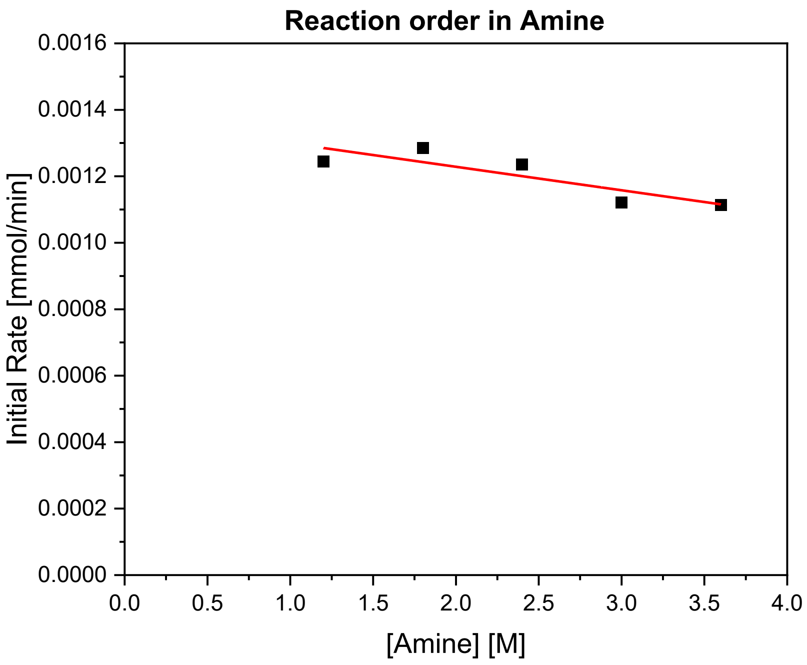

Figure 11: Reaction order in amine.

Reaction Order in CuBr/L3

The initial rates were determined for reactions with five different CuBr/**L3** concentrations: 10.0 mM, 20.0 mM, 40.0 mM, 60.0 mM, and 80.0 mM. To do so, 20 scintillation vials (4 mL size, four per CuBr/**L3** concentration) were charged with K_3PO_4 (127 mg, 0.750 mmol). Note that for each catalyst loading, increasing amounts of the **Cu-B** stock solution are added which leads to an increased amount in DMSO and hexylamine added via stock solution. Consequently, these amounts of DMSO and *n*-hexylamine must be adjusted for each set of reactions.

Next, variable amounts of DMSO (225 μL, 200 μL, 150 μL, 100 μL, and 50 μL) were added to each reaction vial, followed by variable amounts of *n*-hexylamine (0.675 mmol, 0.600 mmol, 0.450 mmol, 0.300 mmol; 0.150 mmol), 61.3 μL 4-chloroansiole (71.3 mg, 0.500 mmol), variable amounts of **Cu-B** stock solution (34.9 μL, 69.8 μL, 140 μL, 209 μL, and 279 μL), and a stir bar. The reactions were removed from the glove box, sealed with electrical tape, stirred for 1 min at room temperature and subsequently stirred for 30, 60, 90, and 120 min. After this time, NMR samples were prepared of each reaction vial (*vide supra*) to determine the yield in the C–N cross-coupled product. The initial rate for each amount of Cu/**L3** (linear regime

of product formation) was plotted against the respective concentration, resulting in a 1st order for Cu/**L3**.

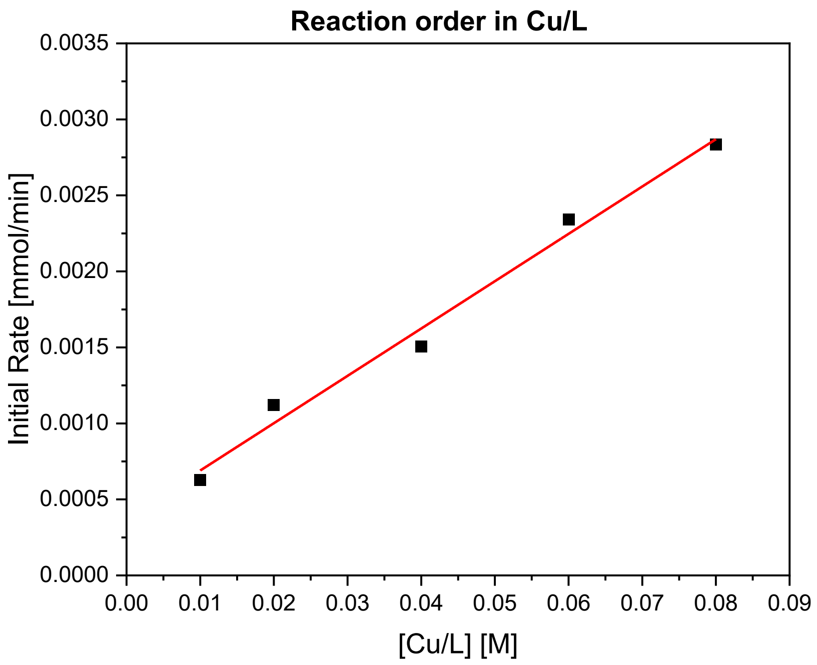

Figure 12: Reaction order in Cu/**L3**.

9. General Procedures for C-N Cross Coupling of Aryl Chlorides with Amines

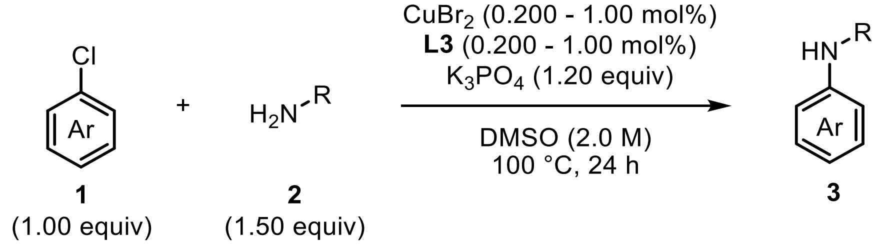

General procedure 1 (GP1): A 4 mL vial was sequentially charged with **L3** (8.0 mg, 10 μmol, 1.0 mol%), K_3PO_4 (1.20 mmol, 256 mg, 1.20 equiv), 0.500 mL of a $CuBr_2$ stock solution in DMSO (20.0 mM, 1.00 mol%), aryl chloride (1.00 mmol, 1.00 equiv), amine (1.50 mmol, 1.50 equiv) and a stir bar. The vial was capped, removed from the glovebox, placed in an aluminum heating block set at 100 °C, and stirred for 24 h. The reaction mixture was concentrated in vacuo and directly purified by silica gel column chromatography using EtOAc/hexane as the eluent to afford the pure product. Note: If the isolated yield is quant. the respective compound was isolated in 100% ± 5% yield.

General procedure 2 (GP2): Whenever the C–N coupling reaction could be conducted with 0.200 mol% $CuBr_2$ and **L3**, while maintaining appreciable yields (see Figure 2 and Figure 3 in the main manuscript), the following procedure was used:

A 4 mL vial was sequentially charged with K_3PO_4 (1.20 mmol, 256 mg, 1.2 equiv), 0.250 mL of a **L3** stock solution in DMSO (8.00 mM, 0.200 mol%), 0.250 mL of a $CuBr_2$ stock solution in DMSO (8.00 mM, 0.200 mol%), aryl chloride (1.00 mmol, 1.00 equiv), amine (1.50 mmol, 1.50 equiv) and a stir bar. The vial was capped, removed from the glovebox, placed in an aluminum heating block set at 100 °C, and stirred for 24 h. For substrates in Figure 2 of the main manuscript 1,3,5-trimethoxybenzene was used as internal standard, while for substrates represented in Figure 3 1,3,5-mesitylene was used as internal standard. NMR spectra were recorded in $CDCl_3$.

Note: Ligand **L3** forms a slurry in DMSO and the amount needed for the reaction should be extracted while the stock solution is stirring.

General procedure 3 (GP3): Whenever the practitioner aims to conduct the C–N coupling reaction with <0.200 mol% $CuBr_2$ and **L3**, while maintaining appreciable yields (see Figure 4 in the main manuscript), the following procedure was used unless otherwise stated:

First, a **Cu-B** stock solution is prepared to ensure that the active catalyst ends up in the reaction. The subsequent procedure is an example that can be scaled up. A 4 mL vial was charged with 22.9 mg CuI (120 µmol, 2.00 equiv) and 1.80 mL DMSO. This mixture was stirred for 30 min at which point the solution was homogenous. A separate 4 mL vial was sequentially charged with 47.8 mg **L3** (60.0 µmol, 1.00 equiv), 254 mg K_3PO_4 (1.20 mmol, 20.0 equiv), 900 µL of the CuI stock solution (60.0 µmol, 1.00 equiv), 159 µL *n*-hexylamine (121 mg, 1.20 mmol, 20.0 equiv) and a stir bar. The vial was removed from the glove box, sealed with electrical tape, stirred for 1 min at room temperature and then stirred at 100 °C for 1.5 h. After this time, the electrical tape was removed, and the vial was brought back into the glove box. 800 µL of the reaction mixture were filtered with a syringe filter. 600 µL of the filtrate were transferred into a 20 mL vial and 5.47 mL DMSO were added. 100 µL of the resulting diluted filtrate 0.200 µmol Cu/**L3**. While it is recommended to always prepare a fresh stock solution, the diluted filtrate can be stored for weeks in the glove box at room temperature while maintaining high activity.

For the catalytic reaction a 4 mL vial was sequentially charged with K_3PO_4 (1.20 mmol, 256 mg, 1.20 equiv), 0.150 mL of the Cu/**L3** stock solution in DMSO (0.300 µmol, 0.03 mol%), aryl chloride (1.00 mmol, 1.00 equiv), amine (1.50 mmol, 1.50 equiv), 0.183 mL DMSO, and a stir bar. The vial was capped, removed from the glovebox, placed in an aluminum heating block set to 130 °C, and stirred for 36 h. For substrate **5o** in Figure 4 of the main manuscript 1,3,5-mesitylene was used as internal standard. NMR spectra were recorded in $CDCl_3$.

10. Substrate Scope

Compound 3a:

***N*-hexyl-4-methoxyaniline**

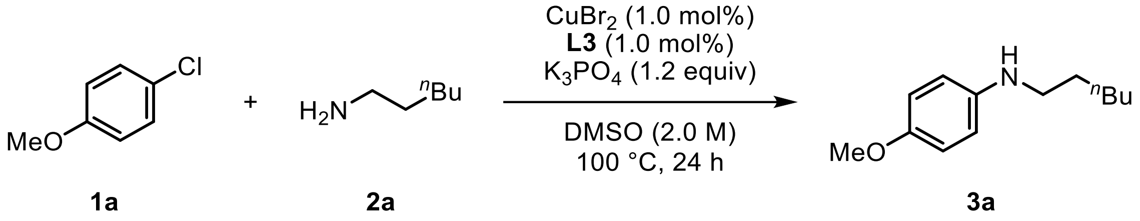

Aniline **3a** was prepared according to **GP1** from aryl chloride **1a** (122 µL, 143 mg, 1.00 mmol, 1.00 equiv) and amine **2a** (198 µL, 152 mg, 1.50 mmol, 1.50 equiv) in DMSO. The crude product was purified via flash column chromatography (SiO_2, EtOAc/hexanes) to give **3a** as a yellow oil.

Yield: 186 mg, 0.899 mmol, 90%

^{1}H NMR (500 MHz, $CDCl_3$): δ 6.78 (d, J = 8.8 Hz, 2H), 6.58 (d, J = 8.9 Hz, 2H), 3.75 (s, 3H), 3.06 (t, J = 7.2 Hz, 2H), 1.60 (p, J = 7.2 Hz, 2H), 1.45 – 1.35 (m, 2H), 1.35 – 1.29 (m, 4H), 0.93 – 0.87 (m, 3H).

^{13}C NMR (126 MHz, $CDCl_3$) δ 152.10, 143.03, 115.04, 114.15, 55.99, 45.18, 31.82, 29.81, 27.03, 22.77, 14.19.

Compound 3b:

***N*-benzyl-4-(tert-butoxy)aniline**

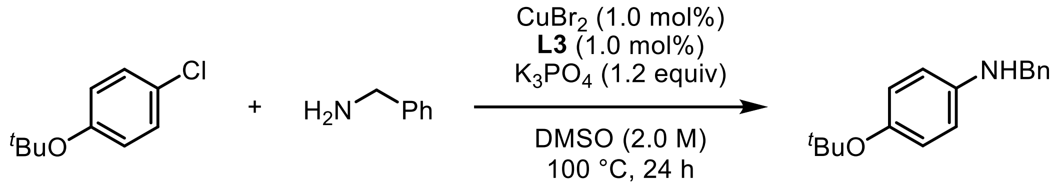

1b **2b** **3b**

Aniline **3c** was prepared according to **GP1** from aryl chloride **1b** (175 µL, 185 mg, 1.00 mmol, 1.00 equiv) and amine **2b** (164 µL, 161 mg, 1.50 mmol, 1.50 equiv) in DMSO. The crude product was purified via flash column chromatography (SiO_2, EtOAc/hexanes) to give **3b** as a yellow oil.

Yield: 265 mg, 1.04 mmol, quant.

^{1}H NMR (500 MHz, $CDCl_3$) δ 7.43 – 7.33 (m, 4H), 7.33 – 7.27 (m, 1H), 6.89 – 6.83 (m, 2H), 6.61 – 6.54 (m, 2H), 4.30 (s, 2H), 3.89 (s, 1H), 1.36 – 1.29 (m, 9H).

^{13}C NMR (101 MHz, $CDCl_3$) δ 146.66, 144.61, 139.67, 128.71, 127.78, 127.34, 125.52, 113.11, 77.80, 49.13, 28.83.

ESI-HR: calc'd for $C_{17}H_{22}NO^+$ ($[M+H]^+$) 256.1696, found 256.1707, residual 4.29 ppm.

Compound 3c:

***N*-benzyl-3,5-dimethoxyaniline**

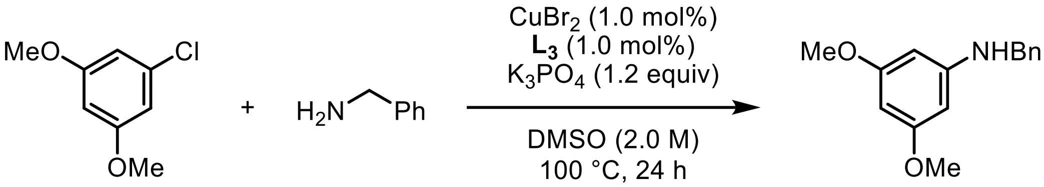

1c **2b** **3c**

Aniline **3c** was prepared according to **GP1** from aryl chloride **1c** (173 mg, 1.00 mmol, 1.00 equiv) and amine **2b** (164 µL, 161 mg, 1.50 mmol, 1.50 equiv) in DMSO. The crude product was purified via flash column chromatography (SiO_2, EtOAc/hexanes) to give **3c** as a yellow solid.

Yield: 244 mg, 1.00 mmol, quant.

^{1}H NMR (400 MHz, $CDCl_3$) δ 7.40 – 7.31 (m, 4H), 7.31 – 7.26 (m, 1H), 5.90 (t, *J* = 2.2 Hz, 1H), 5.84 (d, *J* = 2.2 Hz, 2H), 4.30 (s, 2H), 4.04 (s, 1H), 3.74 (s, 6H).

^{13}C NMR (101 MHz, $CDCl_3$) δ 161.86, 150.22, 139.39, 128.77, 127.67, 127.39, 91.87, 90.06, 55.26, 48.48.

Compound 3d:

***N*-benzyl-4-methylaniline**

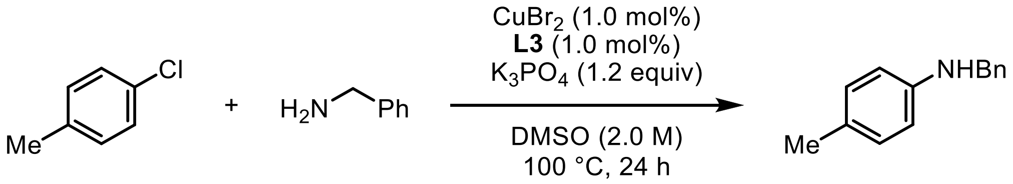

1d **2b** **3d**

Aniline **3d** was prepared according to **GP1** from aryl chloride **1d** (118 µL, 127 mg, 1.00 mmol, 1.00 equiv) and amine **2b** (164 µL, 161 mg, 1.50 mmol, 1.50 equiv) in DMSO. The crude product was purified via flash column chromatography (SiO_2, EtOAc/hexanes) to give **3d** as a yellow oil.

Yield: 199 mg, 1.01 mmol, quant.

^{1}H NMR (500 MHz, $CDCl_3$) δ 7.42 – 7.33 (m, 4H), 7.32 – 7.27 (m, 1H), 7.04 – 6.97 (m, 2H), 6.63 – 6.51 (m, 2H), 4.33 (s, 2H), 3.92 (s, 1H), 2.26 (s, 3H).

^{13}C NMR (101 MHz, $CDCl_3$) δ 146.04, 139.78, 129.84, 128.68, 127.58, 127.23, 126.79, 113.09, 48.71, 20.49.

Compound 3e:

***N*-benzylnaphthalen-2-amine**

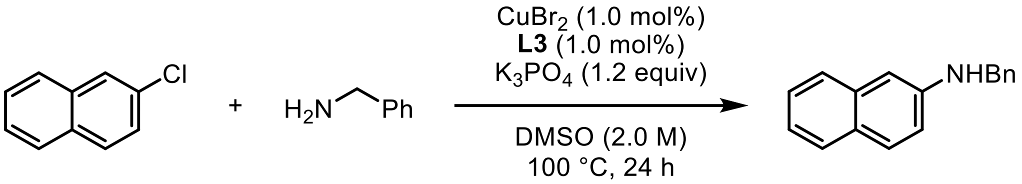

3e **2b** **3e**

Aniline **3e** was prepared according to **GP1** from aryl chloride **1e** (163 mg, 1.00 mmol, 1.00 equiv) and amine **2b** (164 µL, 161 mg, 1.50 mmol, 1.50 equiv) in DMSO. The crude product was purified via flash column chromatography (SiO_2, EtOAc/hexanes) to give **3e** as a yellow solid.

Yield: 237 mg, 1.02 mmol, quant.

^{1}H NMR (400 MHz, $CDCl_3$) δ 7.66 (dd, *J* = 13.5, 8.4 Hz, 2H), 7.60 (d, *J* = 8.3 Hz, 1H), 7.43 (d, *J* = 7.1 Hz, 2H), 7.36 (dtd, *J* = 8.2, 6.4, 1.2 Hz, 3H), 7.33 – 7.27 (m, 1H), 7.24 – 7.16 (m, 1H), 6.93 (dd, *J* = 8.8, 2.4 Hz, 1H), 6.85 (d, *J* = 2.3 Hz, 1H), 4.45 (s, 2H), 4.23 (s, 1H).

^{13}C NMR (101 MHz, $CDCl_3$) δ 146.22, 139.62, 135.62, 129.41, 129.14, 128.09, 128.06, 127.78, 126.77, 126.44, 122.50, 118.30, 105.10, 48.81.

Compound 3f:

***N*-benzyl-4-fluoroaniline**

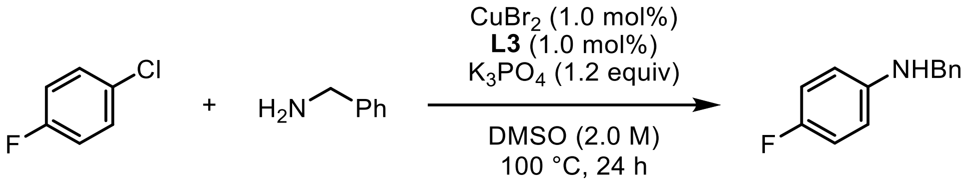

1f **2b** **3f**

Aniline **3f** was prepared according to **GP1** from aryl chloride **1f** (106 µL, 131 mg, 1.00 mmol, 1.00 equiv) and amine **2b** (164 µL, 161 mg, 1.50 mmol, 1.50 equiv) in DMSO. The crude product was purified via flash column chromatography (SiO_2, EtOAc/hexanes) to give **3f** as a yellow solid.

Yield: 184 mg, 0.915 mmol, 92%

^{1}H NMR (400 MHz, $CDCl_3$) δ 7.37 (s, 4H), 7.32 – 7.26 (m, 1H), 6.93 – 6.81 (m, 2H), 6.61 – 6.51 (m, 2H), 4.30 (s, 2H), 3.94 (s, 1H).

^{13}C NMR (101 MHz, $CDCl_3$) δ 156.00, 144.62, 139.37, 128.78, 127.60, 127.42, 115.77, 113.76, 49.04.

^{19}F NMR (376 MHz, $CDCl_3$) δ -127.80.

Compound 3g:

4-(benzylamino)benzonitrile

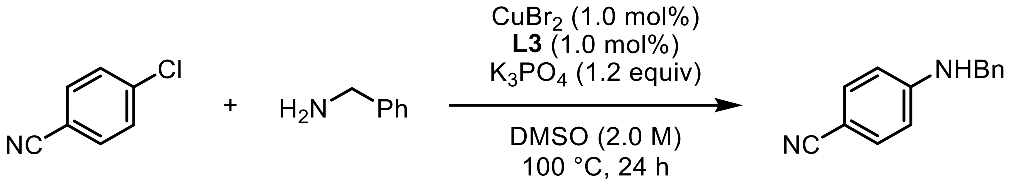

1g **2b** **3g**

Aniline **3g** was prepared according to **GP1** from aryl chloride **1g** (119 µL, 138 mg, 1.00 mmol, 1.00 equiv) and amine **2b** (164 µL, 161 mg, 1.50 mmol, 1.50 equiv) in DMSO. The crude product was purified via flash column chromatography (SiO_2, EtOAc/hexanes) to give **3g** as a yellow solid.

Yield: 204 mg, 0.978 mmol, 98%

^{1}H NMR (400 MHz, $CDCl_3$) δ 7.45 – 7.40 (m, 2H), 7.39 – 7.28 (m, 5H), 6.66 – 6.52 (m, 2H), 4.60 (s, 1H), 4.38 (d, *J* = 5.5 Hz, 2H).

^{13}C NMR (101 MHz, $CDCl_3$) δ 151.23, 137.93, 133.81, 128.96, 127.78, 127.40, 120.50, 112.51, 99.13, 47.57.

Compound 3h:

***tert*-butyl 4-(benzylamino)benzoate**

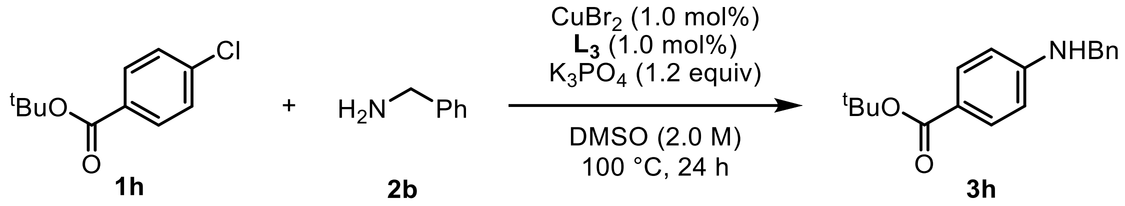

Aniline **3h** was prepared according to **GP1** from aryl chloride **1h** (213 mg, 1.00 mmol, 1.00 equiv) and amine **2b** (164 µL, 161 mg, 1.50 mmol, 1.50 equiv) in DMSO. The crude product was purified via flash column chromatography (SiO_2, EtOAc/hexanes) to give **3h** as a yellow solid.

Yield: 249 mg, 0.877 mmol, 88%

^{1}H NMR (500 MHz, $CDCl_3$) δ 7.85 – 7.78 (m, 2H), 7.34 (s, 4H), 7.29 (ddd, *J* = 8.8, 5.2, 3.6 Hz, 1H), 6.61 – 6.54 (m, 2H), 4.47 (s, 1H), 4.39 (s, 2H), 1.56 (s, 9H).

^{13}C NMR (101 MHz, $CDCl_3$) δ 166.23, 151.50, 138.65, 131.47, 128.89, 127.60, 127.51, 120.85, 111.70, 79.99, 47.85, 28.47.

Compound 3i:

4-(benzylamino)-*N*,*N*-diethylbenzamide

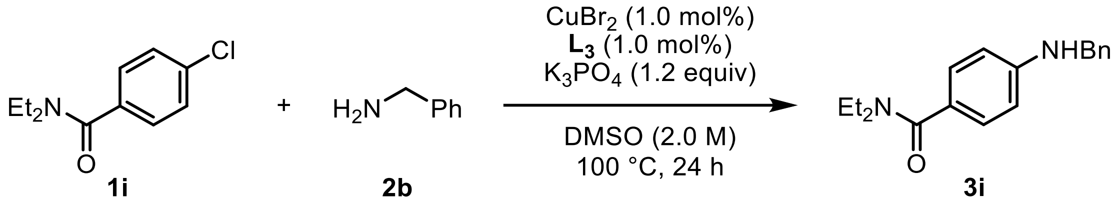

1i **2b** **3i**

Aniline **3i** was prepared according to **GP1** from aryl chloride **1i** (212 mg, 1.00 mmol, 1.00 equiv) and amine **2b** (164 µL, 161 mg, 1.50 mmol, 1.50 equiv) in DMSO. The crude product was purified via flash column chromatography (SiO_2, EtOAc/hexanes) to give **3i** as a yellow solid.

Yield: 292 mg, 1.03 mmol, quant.

^{1}H NMR (500 MHz, $CDCl_3$) δ 7.41 – 7.34 (m, 4H), 7.31 (td, *J* = 5.6, 2.7 Hz, 1H), 7.28 (d, *J* = 2.0 Hz, 1H), 7.27 (d, *J* = 2.0 Hz, 1H), 6.65 – 6.57 (m, 2H), 4.37 (s, 2H), 4.30 (s, 1H), 3.59 – 3.28 (m, 4H), 1.20 (t, *J* = 7.1 Hz, 6H).

^{13}C NMR (101 MHz, $CDCl_3$) δ 171.87, 149.14, 139.06, 128.82, 128.56, 127.56, 127.49, 125.98, 112.13, 48.13.

ESI-HR: calc'd for $C_{18}H_{23}N_2O^+$ ($[M+H]^+$) 283.1805, found 283.1813, residual 2.83 ppm.

Compound 3j:

3-(benzylamino)benzonitrile

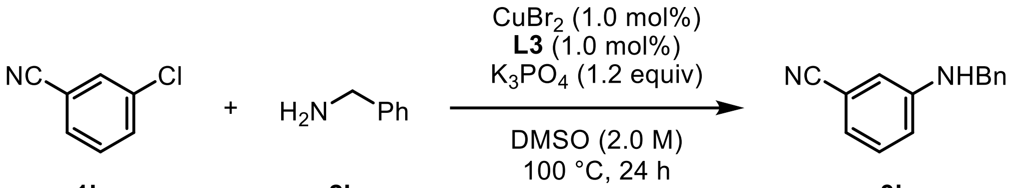

1j **2b** **3j**

Aniline **3j** was prepared according to **GP1** from aryl chloride **1j** (121 µL, 138 mg, 1.00 mmol, 1.00 equiv) and amine **2b** (164 µL, 161 mg, 1.50 mmol, 1.50 equiv) in DMSO. The crude product was purified via flash column chromatography (SiO_2, EtOAc/hexanes) to give **3j** as a yellow solid.

Yield: 208 mg, 1.00 mmol, quant.

^{1}H NMR (400 MHz, $CDCl_3$) δ 7.35 (s, 5H), 7.24 – 7.18 (m, 1H), 6.98 (dt, *J* = 7.6, 1.2 Hz, 1H), 6.82 (dq, *J* = 10.6, 1.5 Hz, 2H), 4.34 (s, 3H).

^{13}C NMR (101 MHz, $CDCl_3$) δ 148.35, 138.26, 130.03, 128.96, 127.74, 127.48, 121.09, 119.55, 117.37, 115.17, 113.09, 48.00.

Compound 3k:

***N*-benzyl-3-nitroaniline**

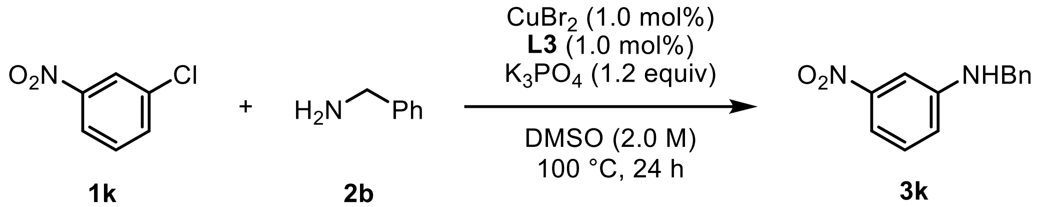

Aniline **3k** was prepared according to **GP1** from aryl chloride **1k** (103 µL, 158 mg, 1.00 mmol, 1.00 equiv) and amine **2b** (164 µL, 161 mg, 1.50 mmol, 1.50 equiv) in DMSO. The crude product was purified via flash column chromatography (SiO_2, EtOAc/hexanes) to give **3k** as a yellow solid.

Yield: 198 mg, 0.867 mmol, 87%

^{1}H NMR (400 MHz, $CDCl_3$) δ 7.52 (dd, *J* = 8.1, 2.2 Hz, 1H), 7.43 (t, *J* = 2.3 Hz, 1H), 7.36 (d, *J* = 4.4 Hz, 4H), 7.32 – 7.27 (m, 1H), 7.25 (s, 1H), 6.87 (dd, *J* = 8.2, 2.4 Hz, 1H), 4.38 (s, 3H).

^{13}C NMR (101 MHz, $CDCl_3$) δ 148.88, 138.16, 129.89, 129.00, 127.82, 127.60, 118.83, 112.30, 106.72, 48.21.

Compound 3l:

(4-(benzylamino)phenyl)(phenyl)methanone

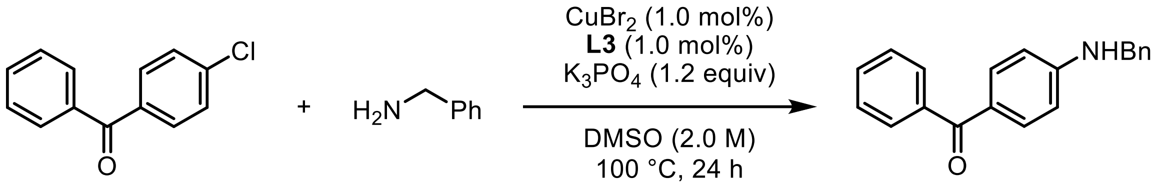

Aniline **3l** was prepared according to **GP1** from aryl chloride **1l** (217 mg, 1.00 mmol, 1.00 equiv) and amine **2b** (164 µL, 161 mg, 1.50 mmol, 1.50 equiv) in DMSO. The crude product was purified via flash column chromatography (SiO_2, EtOAc/hexanes) to give **3l** as a yellow solid.

Yield: 278 mg, 0.968 mmol, 97%

^{1}H NMR (500 MHz, $CDCl_3$) δ 7.73 (ddd, *J* = 15.5, 7.6, 1.8 Hz, 4H), 7.57 – 7.50 (m, 1H), 7.50 – 7.41 (m, 2H), 7.36 (s, 4H), 7.35 – 7.30 (m, 1H), 6.69 – 6.56 (m, 2H), 4.67 (s, 1H), 4.42 (d, *J* = 4.3 Hz, 2H).

^{13}C NMR (101 MHz, $CDCl_3$) δ 195.30, 151.98, 139.21, 138.37, 133.11, 131.36, 129.59, 128.95, 128.15, 127.71, 127.52, 126.60, 111.68, 47.76.

Compound 3m:

***N*-benzylbenzo[*d*][1,3]dioxol-5-amine**

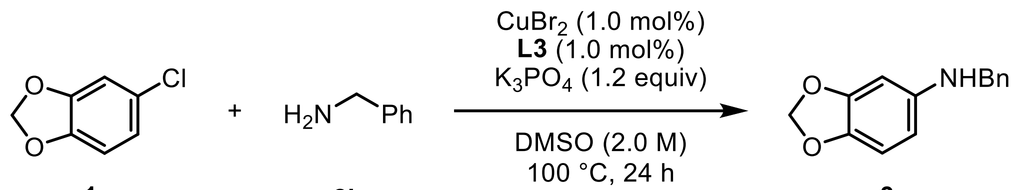

Aniline **3m** was prepared according to **GP1** from aryl chloride **1m** (118 µL, 157 mg, 1.00 mmol, 1.00 equiv) and amine **2b** (164 µL, 161 mg, 1.50 mmol, 1.50 equiv) in DMSO. The crude product was purified via flash column chromatography (SiO_2, EtOAc/hexanes) to give **3m** as a yellow solid.

Yield: 217 mg, 0.955 mmol, 96%

^{1}H NMR (400 MHz, $CDCl_3$) δ 7.40 – 7.32 (m, 4H), 7.27 (s, 1H), 6.65 (d, J = 8.3 Hz, 1H), 6.27 (d, J = 2.3 Hz, 1H), 6.08 (dd, J = 8.3, 2.4 Hz, 1H), 5.85 (s, 2H), 4.27 (s, 2H), 3.83 (s, 1H).

^{13}C NMR (101 MHz, $CDCl_3$) δ 148.4, 144.1, 139.8, 139.5, 128.7, 127.6, 127.3, 108.7, 104.5, 100.7, 96.1, 49.3.

Compound 3n:

***N*-benzylbenzo[*b*]thiophen-5-amine**

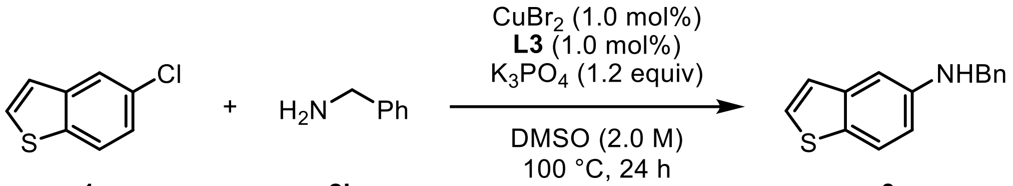

1n **2b** **3n**

Aniline **3n** was prepared according to **GP1** from aryl chloride **1n** (125 µL, 169 mg, 1.00 mmol, 1.00 equiv) and amine **2b** (164 µL, 161 mg, 1.50 mmol, 1.50 equiv) in DMSO. The crude product was purified via flash column chromatography (SiO_2, EtOAc/hexanes) to give **3n** as a yellow solid.

Yield: 230 mg, 0.963 mmol, 96%

^{1}H NMR (400 MHz, $CDCl_3$) δ 7.63 (d, *J* = 8.6 Hz, 1H), 7.44 – 7.39 (m, 2H), 7.39 – 7.32 (m, 3H), 7.32 – 7.27 (m, 1H), 7.15 (dd, *J* = 5.4, 0.8 Hz, 1H), 7.01 (d, *J* = 2.3 Hz, 1H), 6.77 (dd, *J* = 8.6, 2.3 Hz, 1H), 4.40 (s, 2H), 4.11 (s, 1H).

^{13}C NMR (101 MHz, $CDCl_3$) δ 145.81, 141.10, 139.52, 129.58, 128.77, 127.64, 127.36, 126.98, 123.49, 122.95, 114.03, 105.09, 48.86.

Compound 3o:

***N*-benzylpyridin-3-amine**

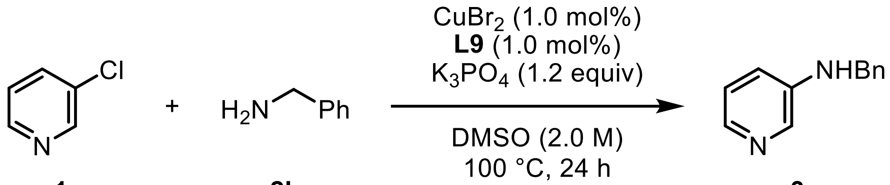

1o **2b** **3o**

Aniline **3o** was prepared according to **GP1** from aryl chloride **1o** (94.7 µL, 114 mg, 1.00 mmol, 1.00 equiv) and amine **2b** (164 µL, 161 mg, 1.50 mmol, 1.50 equiv) in DMSO. The crude product was purified via flash column chromatography (SiO_2, EtOAc/hexanes) to give **3o** as a yellow oil.

Yield: 167 mg, 0.906 mmol, 91%

^{1}H NMR (400 MHz, $CDCl_3$) δ 8.08 (d, *J* = 2.9 Hz, 1H), 7.97 (dd, *J* = 4.7, 1.4 Hz, 1H), 7.35 (s, 4H), 7.33 – 7.27 (m, 1H), 7.06 (dd, *J* = 8.3, 4.6 Hz, 1H), 6.87 (ddd, *J* = 8.3, 2.9, 1.3 Hz, 1H), 4.35 (d, *J* = 4.6 Hz, 2H), 4.26 – 4.05 (m, 1H).

^{13}C NMR (101 MHz, CHLOROFORM-*D*) δ 144.14, 139.11, 138.65, 136.34, 128.91, 127.65, 127.55, 123.83, 118.70, 48.03.

Compound 3p:

***N*-benzyl-5-(trifluoromethyl)pyridin-2-amine**

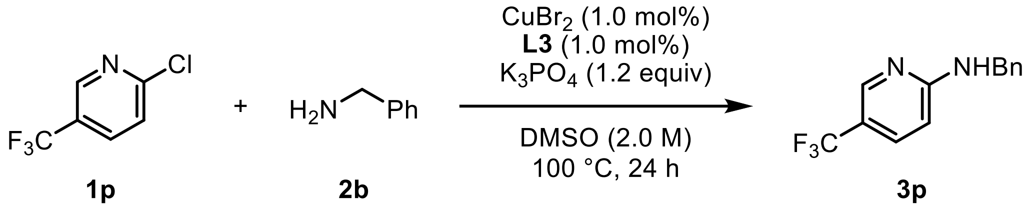

1p **2b** **3p**

Aniline **3p** was prepared according to **GP1** from aryl chloride **1p** (182 mg, 1.00 mmol, 1.00 equiv) and amine **2b** (164 µL, 161 mg, 1.50 mmol, 1.50 equiv) in DMSO. The crude product was purified via flash column chromatography (SiO_2, EtOAc/hexanes) to give **3p** as a yellow solid.

Yield: 237 mg, 0.941 mmol, 94%

^{1}H NMR (500 MHz, CD_3CN) δ 8.30 (dt, *J* = 2.3, 1.1 Hz, 1H), 7.60 (dd, *J* = 8.9, 2.5 Hz, 1H), 7.38 – 7.30 (m, 4H), 7.25 (ddt, *J* = 8.6, 5.9, 2.2 Hz, 1H), 6.55 (d, *J* = 8.8 Hz, 1H), 6.24 (s, 1H), 4.57 (d, *J* = 6.2 Hz, 2H).

Compound 3q:

***N*-benzyl-6-fluoropyridin-2-amine**

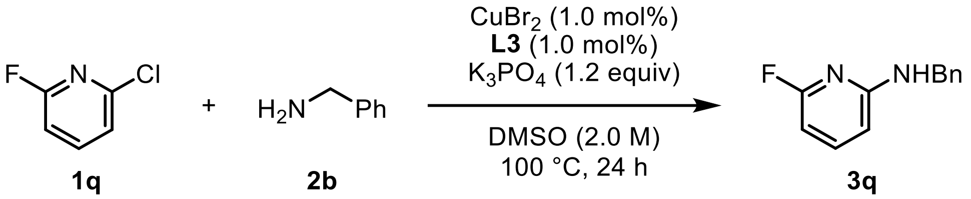

Aniline **3q** was prepared according to **GP1** from aryl chloride **1q** (132 mg, 1.00 mmol, 1.00 equiv) and amine **2b** (164 µL, 161 mg, 1.50 mmol, 1.50 equiv) in DMSO. The crude product was purified via flash column chromatography (SiO_2, EtOAc/hexanes) to give **3q** as a yellow solid.

Yield: 204 mg, 1.01 mmol, quant.

^{1}H NMR (400 MHz, $CDCl_3$) δ 7.34 (s, 5H), 7.32 – 7.27 (m, 1H), 6.60 (d, *J* = 7.5 Hz, 1H), 6.24 (d, *J* = 8.2 Hz, 1H), 5.08 (s, 1H), 4.48 (d, *J* = 5.6 Hz, 2H).

^{13}C NMR (101 MHz, $CDCl_3$) δ 158.77, 149.77, 139.92, 138.56, 128.86, 127.58, 127.51, 112.43, 104.49, 46.50.

^{19}F NMR (376 MHz, $CDCl_3$) δ -69.57.

Compound 3r:

***N*-benzyl-6-methylpyridin-2-amine**

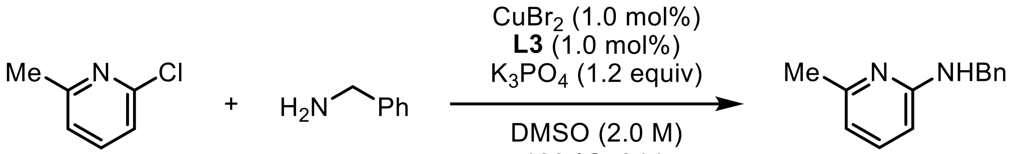

1r **2b** **3r**

Aniline **3r** was prepared according to **GP1** from aryl chloride **1r** (128 mg, 1.00 mmol, 1.00 equiv) and amine **2b** (164 µL, 161 mg, 1.50 mmol, 1.50 equiv) in DMSO. The crude product was purified via flash column chromatography (SiO_2, EtOAc/hexanes) to give **3r** as a yellow solid.

Yield: 195 mg, 0.984 mmol, 98%

^{1}H NMR (500 MHz, $CDCl_3$) δ 7.39 – 7.29 (m, 5H), 7.26 (s, 1H), 6.47 (d, *J* = 7.3 Hz, 1H), 6.17 (d, *J* = 8.2 Hz, 1H), 4.90 (s, 1H), 4.46 (d, *J* = 5.9 Hz, 2H), 2.39 (s, 3H).

^{13}C NMR (101 MHz, $CDCl_3$) δ 158.47, 157.12, 139.34, 138.04, 128.69, 127.45, 127.28, 112.64, 102.98, 46.67, 24.44.

Compound 3s:

***N*-benzylquinolin-7-amine**

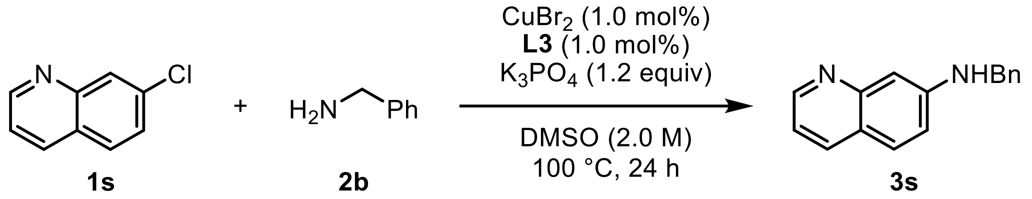

Aniline **3s** was prepared according to **GP1** from aryl chloride **1s** (164 mg, 1.00 mmol, 1.00 equiv) and amine **2b** (164 µL, 161 mg, 1.50 mmol, 1.50 equiv) in DMSO. The crude product was purified via flash column chromatography (SiO_2, EtOAc/hexanes) to give **3s** as a yellow solid.

Yield: 236 mg, 1.01 mmol, quant.

^{1}H NMR (400 MHz, $CDCl_3$) δ 8.72 (dd, *J* = 4.4, 1.8 Hz, 1H), 7.59 (d, *J* = 8.8 Hz, 1H), 7.46 – 7.39 (m, 2H), 7.39 – 7.33 (m, 2H), 7.33 – 7.27 (m, 1H), 7.16 – 7.07 (m, 2H), 6.96 (dd, *J* = 8.8, 2.4 Hz, 1H), 4.47 (s, 3H).

^{13}C NMR (101 MHz, $CDCl_3$) δ 150.63, 150.55, 149.01, 138.68, 135.71, 128.88, 128.73, 127.78, 127.60, 122.10, 118.58, 117.50, 105.57, 48.20.

ESI-HR: calc'd for $C_{16}H_{15}N_2^+$ ($[M+H]^+$) 235.1230, found 235.1219, residual 4.68 ppm.

Compound 3t:

***N*-benzylquinolin-6-amine**

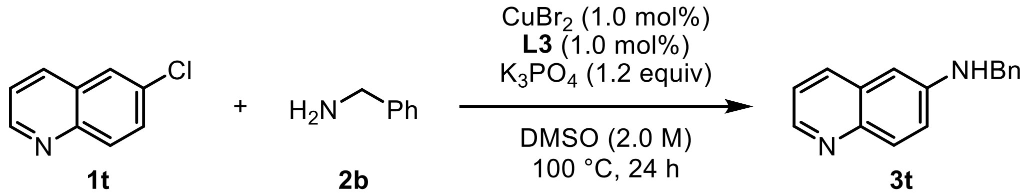

Aniline **3t** was prepared according to **GP1** from aryl chloride **1t** (164 mg, 1.00 mmol, 1.00 equiv) and amine **2b** (164 µL, 161 mg, 1.50 mmol, 1.50 equiv) in DMSO. The crude product was purified via flash column chromatography (SiO_2, EtOAc/hexanes) to give **3t** as a yellow solid.

Yield: 227 mg, 0.971 mmol, 97%

^{1}H NMR (400 MHz, $CDCl_3$) δ 8.62 (dd, *J* = 4.2, 1.7 Hz, 1H), 7.90 (dd, *J* = 8.5, 2.1 Hz, 2H), 7.44 – 7.35 (m, 4H), 7.35 – 7.28 (m, 1H), 7.26 – 7.23 (m, 1H), 7.15 (dd, *J* = 9.0, 2.6 Hz, 1H), 6.73 (d, *J* = 2.6 Hz, 1H), 4.45 (d, *J* = 5.3 Hz, 2H), 4.37 (d, *J* = 6.4 Hz, 1H).

^{13}C NMR (101 MHz, $CDCl_3$) δ 146.38, 146.09, 143.42, 138.81, 134.00, 130.44, 130.20, 128.88, 127.65, 127.58, 121.48, 121.41, 103.42, 48.40.

Compound 3u:

***N*-benzyl-6'-methyl-3-(4-(methylsulfonyl)phenyl)-[2,3'-bipyridin]-5-amine**

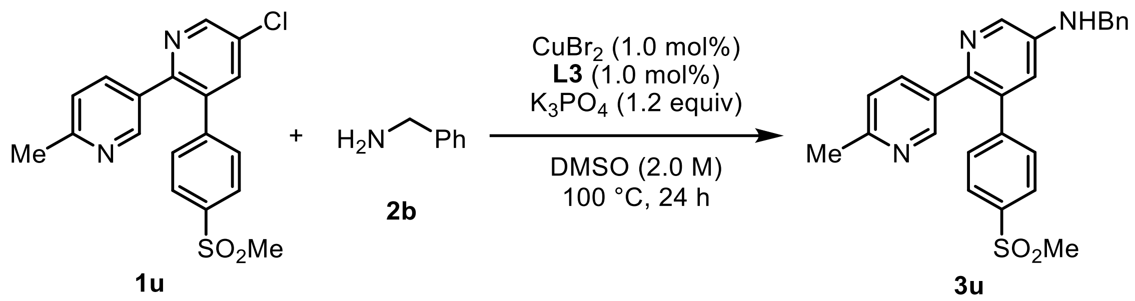

Aniline **3u** was prepared according to **GP1** from aryl chloride **1u** (359 mg, 1.00 mmol, 1.00 equiv) and amine **2b** (164 µL, 161 mg, 1.50 mmol, 1.50 equiv) in DMSO. The crude product was purified via flash column chromatography (SiO_2, EtOAc/hexanes) to give **3u** as a yellow solid.

Yield: 330 mg, 0.768 mmol, 77%

^{1}H NMR (500 MHz, $CDCl_3$) δ 8.31 (d, *J* = 2.3 Hz, 1H), 8.20 (d, *J* = 2.7 Hz, 1H), 7.86 – 7.80 (m, 2H), 7.50 (dd, *J* = 8.0, 2.3 Hz, 1H), 7.41 – 7.27 (m, 7H), 7.03 (d, *J* = 8.0 Hz, 1H), 6.86 (d, *J* = 2.7 Hz, 1H), 4.69 (t, *J* = 5.7 Hz, 1H), 4.42 (d, *J* = 5.5 Hz, 2H), 3.06 (s, 3H), 2.50 (s, 3H).

^{13}C NMR (126 MHz, $CDCl_3$) δ 156.85, 149.81, 145.94, 143.32, 142.82, 139.42, 138.09, 137.21, 136.01, 134.64, 132.52, 130.48, 128.93, 127.73, 127.62, 127.44, 122.67, 120.47, 47.77, 44.57, 24.15.

ESI-HR: calc'd for $C_{50}H_{47}N_6O_4S_2^+$ ($[2M+H]^+$) 859.3095, found 859.3105, residual 1.16 ppm

Compound 5a:

4-methoxy-*N*-(thiophen-2-ylmethyl)aniline

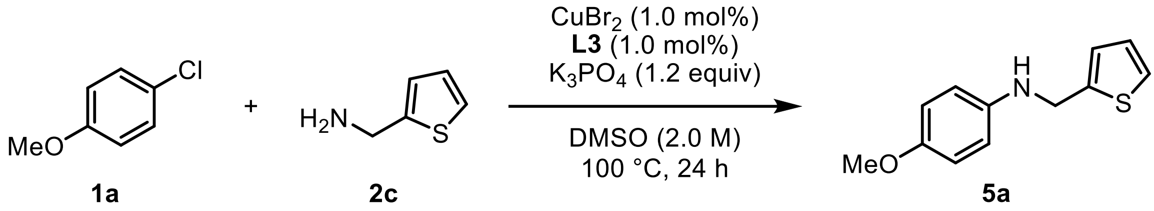

Aniline **5a** was prepared according to **GP1** from aryl chloride **1a** (122 µL, 143 mg, 1.00 mmol, 1.00 equiv) and amine **2c** (154 µL, 170 mg, 1.50 mmol, 1.50 equiv) in DMSO. The crude product was purified via flash column chromatography (SiO_2, EtOAc/hexanes) to give **5a** as a yellow solid.

Yield: 210 mg, 0.958 mmol, 96%

^{1}**H NMR** (400 MHz, $CDCl_3$) δ 7.23 – 7.18 (m, 1H), 7.00 (dq, *J* = 3.3, 1.0 Hz, 1H), 6.96 (dd, *J* = 5.0, 3.4 Hz, 1H), 6.83 – 6.77 (m, 2H), 6.69 – 6.62 (m, 2H), 4.47 (d, *J* = 1.0 Hz, 2H), 3.75 (s, 3H).

^{13}**C NMR** (101 MHz, $CDCl_3$) δ 152.68, 143.41, 141.94, 126.90, 125.00, 124.60, 114.98, 114.70, 55.86, 44.59.

Compound 5b:

***N*-(furan-2-ylmethyl)-4-methoxyaniline**

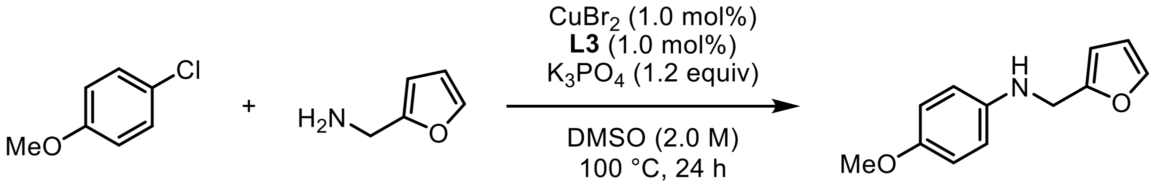

1a **2d** **5b**

Aniline **5b** was prepared according to **GP1** from aryl chloride **1a** (122 µL, 143 mg, 1.00 mmol, 1.00 equiv) and amine **2d** (135 µL, 146 mg, 1.00 mmol, 1.00 equiv) in DMSO. The crude product was purified via flash column chromatography (SiO_2, EtOAc/hexanes) to give **5b** as a yellow solid.

Yield: 205.2 mg, 1.01 mmol, quant.

^{1}H NMR (500 MHz, $CDCl_3$) δ 7.41 – 7.33 (m, 1H), 6.83 – 6.76 (m, 2H), 6.69 – 6.62 (m, 2H), 6.32 (dd, *J* = 3.2, 1.9 Hz, 1H), 6.22 (d, *J* = 3.2 Hz, 1H), 4.28 (s, 2H), 3.75 (s, 3H).

^{13}C NMR (101 MHz, $CDCl_3$) δ 153.15, 152.69, 141.97, 141.93, 114.95, 114.75, 110.42, 107.01, 55.86, 42.54.

Compound 5c:

4-((4-(trifluoromethyl)phenyl)amino)butan-1-ol

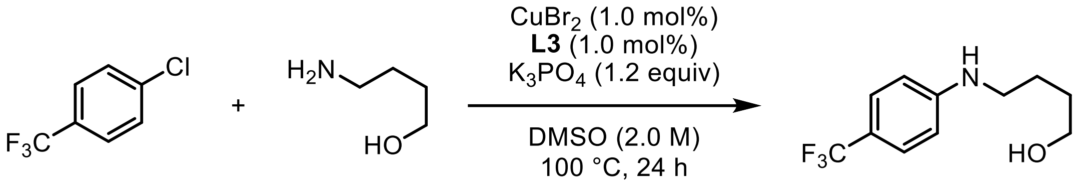

1w **2e** **5c**

Aniline **5c** was prepared according to **GP1** from aryl chloride **1w** (134 µL, 180.6 mg, 1.00 mmol, 1.00 equiv) and amine **2e** (139 µL, 134 mg, 1.00 mmol, 1.00 equiv) in DMSO. The crude product was purified via flash column chromatography (SiO_2, EtOAc/hexanes) to give **5c** as a yellow oil.

Yield: 211 mg, 0.906 mmol, 91%

^{1}H NMR (400 MHz, $CDCl_3$) δ 7.39 (d, *J* = 8.3 Hz, 2H), 6.59 (d, *J* = 8.5 Hz, 2H), 3.69 (t, *J* = 5.9 Hz, 2H), 3.18 (t, *J* = 6.5 Hz, 2H), 1.70 (ttd, *J* = 12.2, 6.2, 2.5 Hz, 4H).

^{13}C NMR (101 MHz, $CDCl_3$) δ 150.89, 126.70, 125.16, 118.65, 111.86, 62.57, 43.41, 30.19, 25.84.

Compound 5d:

***N*-(4-aminobenzyl)-4-(trifluoromethyl)aniline**

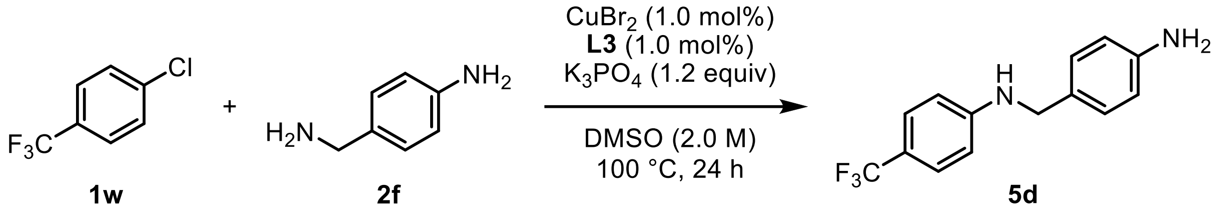

Aniline **5d** was prepared according to **GP1** from aryl chloride **1w** (134 µL, 181 mg, 1.00 mmol, 1.00 equiv) and amine **2f** (170 µL, 183 mg, 1.00 mmol, 1.00 equiv) in DMSO. The crude product was purified via flash column chromatography (SiO_2, EtOAc/hexanes) to give **5d** as a yellow solid.

Yield: 221 mg, 0.829 mmol, 83%

^{1}H NMR (400 MHz, $CDCl_3$) δ 7.39 (d, *J* = 8.5 Hz, 2H), 7.18 – 7.11 (m, 2H), 6.72 – 6.65 (m, 2H), 6.62 (d, *J* = 8.4 Hz, 2H), 4.22 (s, 3H), 3.71 (s, 1H).

^{13}C NMR (101 MHz, $CDCl_3$) δ 150.74, 146.01, 128.90, 128.27, 126.70, 125.15, 118.87, 115.41, 112.01, 47.60.

ESI-HR: calc'd for $C_{14}H_{14}F_3N_2^+$ ($[M+H]^+$) 267.1104, found 267.1108, residual 1.50 ppm

Compound 5e:

***N*-(4-phenylbutyl)pyridin-3-amine**

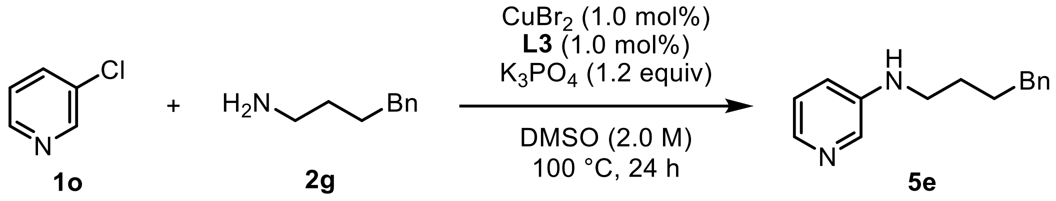

Aniline **5e** was prepared according to **GP1** from aryl chloride **1o** (94.7 µL, 114 mg, 1.00 mmol, 1.00 equiv) and amine **2g** (239 µL, 224 mg, 1.50 mmol, 1.00 equiv) in DMSO. The crude product was purified via flash column chromatography (SiO_2, EtOAc/hexanes) to give **5e** as a yellow solid.

Yield: 222 mg, 0.982 mmol, 98%

^{1}H NMR (500 MHz, $CDCl_3$) δ 8.00 (d, *J* = 2.9 Hz, 1H), 7.98 – 7.89 (m, 1H), 7.29 (dd, *J* = 8.6, 6.6 Hz, 2H), 7.24 – 7.16 (m, 3H), 7.06 (dd, *J* = 8.3, 4.6 Hz, 1H), 6.83 (ddd, *J* = 8.3, 2.9, 1.3 Hz, 1H), 3.72 (s, 1H), 3.12 (t, *J* = 6.9 Hz, 2H), 2.67 (t, *J* = 7.5 Hz, 2H), 1.75 (dddd, *J* = 14.4, 9.7, 7.0, 2.0 Hz, 2H), 1.72 – 1.62 (m, 2H).

^{13}C NMR (101 MHz, $CDCl_3$) δ 144.42, 142.09, 138.55, 136.03, 128.46, 125.97, 123.78, 118.38, 43.50, 35.67, 29.00, 28.87.

ESI-HR: calc'd for $C_{30}H_{34}N_4^+$ ($[2M+H]^+$) 453.3013, found 453.3013, residual 4.13 ppm

Compound 5f:

***N*-cyclopentylpyridin-3-amine**

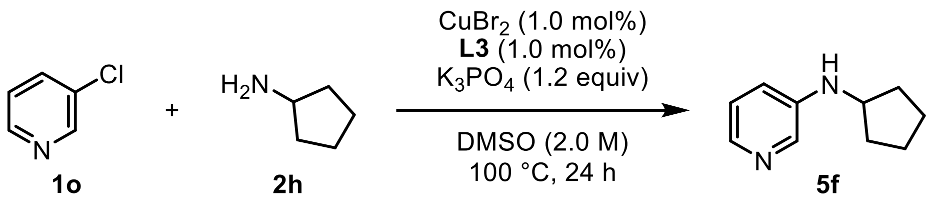

Aniline **5f** was prepared according to **GP1** from aryl chloride **1o** (94.7 µL, 114 mg, 1.00 mmol, 1.00 equiv) and amine **2h** (148 µL, 128 mg, 1.50 mmol, 1.50 equiv) in DMSO. The crude product was purified via flash column chromatography (SiO_2, EtOAc/hexanes) to give **5f** as a yellow solid.

Yield: 150 mg, 0.923 mmol, 92%

^{1}H NMR (500 MHz, $CDCl_3$) δ 8.12 – 7.96 (m, 1H), 7.93 (d, *J* = 4.7 Hz, 1H), 7.06 (dd, *J* = 8.3, 4.7 Hz, 1H), 6.86 (ddd, *J* = 8.3, 2.9, 1.3 Hz, 1H), 3.78 (h, *J* = 5.8 Hz, 1H), 3.74 – 3.64 (m, 1H), 2.03 (s, 2H), 1.81 – 1.58 (m, 4H), 1.55 – 1.41 (m, 2H).

^{13}C NMR (101 MHz, $CDCl_3$) δ 144.07, 138.24, 136.34, 123.72, 118.86, 54.40, 33.48, 24.10.

ESI-HR: calc'd for $C_{20}H_{29}N_4^+$ ($[2M+H]^+$) 325.2387, found 325.2391, residual 1.23 ppm.

Compound 5g:

***N*-propylquinolin-6-amine**

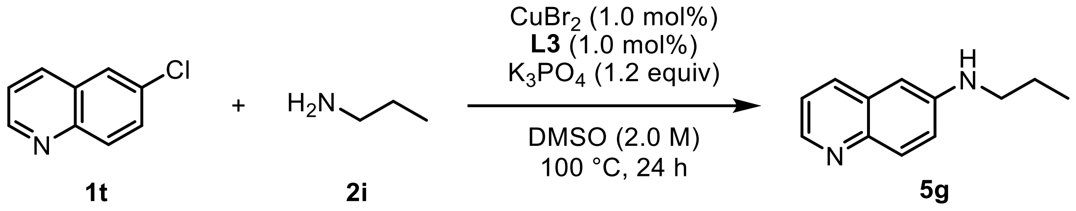

Aniline **5g** was prepared according to **GP1** from aryl chloride **1t** and amine **2i** in DMSO. The crude product was purified via flash column chromatography (SiO_2, EtOAc/hexanes) to give **5g** as a yellow solid.

Yield: 197 mg, 1.06 mmol, quant.

^{1}H NMR (400 MHz, $CDCl_3$) δ 8.60 (dd, *J* = 4.3, 1.7 Hz, 1H), 7.97 – 7.80 (m, 2H), 7.26 (s, 1H), 7.08 (dd, *J* = 9.0, 2.6 Hz, 1H), 6.68 (d, *J* = 2.6 Hz, 1H), 3.96 (s, 1H), 3.19 (td, *J* = 8.1, 4.8 Hz, 2H), 1.72 (h, *J* = 7.3 Hz, 2H), 1.05 (t, *J* = 7.4 Hz, 3H).

^{13}C NMR (101 MHz, CHLOROFORM-*D*) δ 146.46, 146.06, 143.25, 133.78, 130.32, 130.28, 121.47, 121.42, 102.78, 45.84, 22.58, 11.78.

Compound 5h:

***N*-(cyclopropylmethyl)quinolin-6-amine**

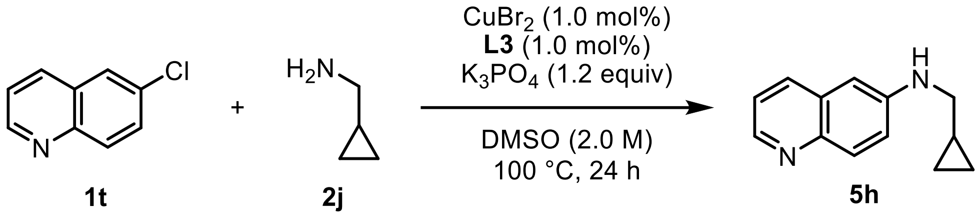

Aniline **5h** was prepared according to **GP1** from aryl chloride and amine **2j** in DMSO. The crude product was purified via flash column chromatography (SiO_2, EtOAc/hexanes) to give **5h** as a yellow solid.

Yield: 200 mg, 1.01 mmol, quant.

^{1}H NMR (400 MHz, $CDCl_3$) δ 8.60 (dd, *J* = 4.2, 1.7 Hz, 1H), 7.98 – 7.79 (m, 2H), 7.26 (s, 1H), 7.11 (dd, *J* = 9.0, 2.6 Hz, 1H), 6.67 (d, *J* = 2.6 Hz, 1H), 4.13 (s, 1H), 3.06 (dd, *J* = 7.3, 3.2 Hz, 2H), 1.23 – 1.08 (m, 1H), 0.70 – 0.53 (m, 2H), 0.30 (dt, *J* = 6.1, 4.5 Hz, 2H).

^{13}C NMR (101 MHz, $CDCl_3$) δ 146.42, 146.11, 143.29, 133.78, 130.28, 121.47, 121.41, 102.87, 49.05, 10.76, 3.64.

ESI-HR: calc'd for $C_{24}H_{25}N_4^+$ ($[2M+H]^+$) 397.2387, found 397.2399, residual 3.02 ppm

Compound 5i:

4-methoxy-*N*-(pyridin-3-ylmethyl)aniline

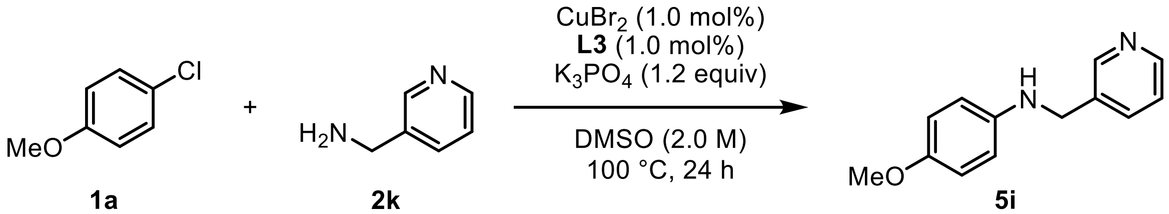

1a **2k** **5i**

Aniline **5i** was prepared according to **GP1** from aryl chloride **1a** and amine **2k** in DMSO. The crude product was purified via flash column chromatography (SiO_2, EtOAc/hexanes) to give **5i** as a yellow solid.

Yield: 176 mg, 0.822 mmol, 82%

^{1}H NMR (400 MHz, $CDCl_3$) δ 8.63 (d, *J* = 2.3 Hz, 1H), 8.53 (dd, *J* = 4.9, 1.7 Hz, 1H), 7.70 (dt, *J* = 7.9, 2.0 Hz, 1H), 7.27 (d, *J* = 5.3 Hz, 1H), 6.83 – 6.73 (m, 2H), 6.60 (d, *J* = 8.9 Hz, 2H), 4.32 (s, 2H), 3.74 (s, 3H).

^{13}C NMR (101 MHz, $CDCl_3$) δ 152.56, 149.30, 148.74, 141.93, 135.22, 123.59, 115.03, 114.36, 55.84, 46.78.

Compound 5j:

***N*-(2-morpholinoethyl)pyridin-3-amine**

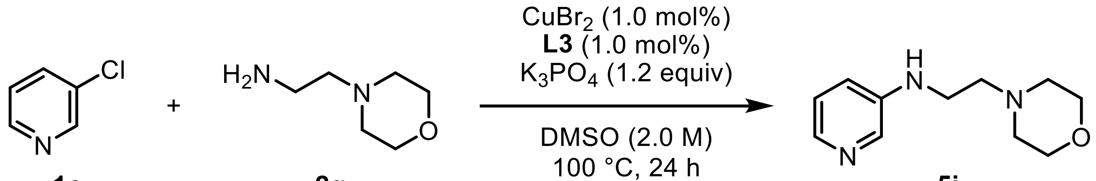

1o 2q 5j

Aniline **5o** was prepared according to **GP1** from aryl chloride **1s** (94.7 µL, 114 mg, 1.00 mmol, 1.00 equiv) and amine **2q** (196 µL, 195 mg, 1.50 mmol, 1.50 equiv) in DMSO. The crude product was purified via flash column chromatography (SiO_2, EtOAc/hexanes) to give **5j** as a yellow solid.

Yield: 183 mg, 0.884 mmol, 88%

^{1}H NMR (400 MHz, $CDCl_3$) δ 8.04 (s, 1H), 8.00 – 7.90 (m, 1H), 7.07 (dd, *J* = 8.3, 4.6 Hz, 1H), 6.87 (ddd, *J* = 8.3, 2.9, 1.3 Hz, 1H), 4.37 (s, 1H), 3.78 – 3.65 (m, 4H), 3.16 (q, *J* = 5.4 Hz, 2H), 2.64 (dd, *J* = 6.6, 5.1 Hz, 2H), 2.47 (dd, *J* = 5.7, 3.5 Hz, 4H).

^{13}C NMR (101 MHz, $CDCl_3$) δ 144.42, 138.76, 136.12, 123.78, 118.71, 67.00, 56.97, 53.39, 39.53.

ESI-HR: calc'd for $C_{22}H_{35}N_6O_2^+$ ($[2M+H]^+$) 415.2817, found 415.2834, residual 4.09 ppm

Compound 5k:

1-benzyl-*N*-(4-(trifluoromethyl)phenyl)piperidin-4-amine

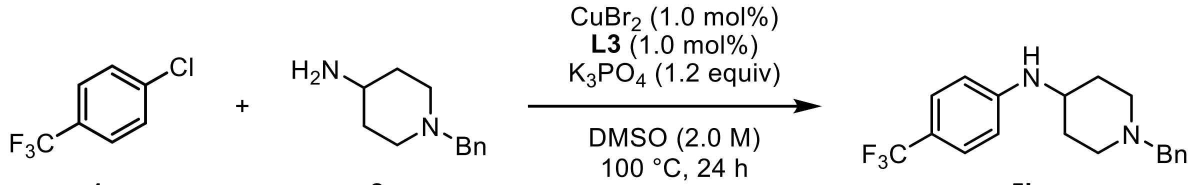

Aniline **5k** was prepared according to **GP1** from aryl chloride **1w** (134 µL, 181 mg, 1.00 mmol, 1.00 equiv) and amine **2m** (285 mg, 303 µL, 1.00 mmol, 1.00 equiv) in DMSO. The crude product was purified via flash column chromatography (SiO_2, EtOAc/hexanes) to give **5k** as a yellow solid.

Yield: 258 mg, 0.771 mmol, 77%

^{1}H NMR (500 MHz, $CDCl_3$) δ 7.38 (d, *J* = 8.5 Hz, 2H), 7.33 (s, 4H), 7.30 – 7.24 (m, 1H), 6.58 (d, *J* = 8.5 Hz, 2H), 3.88 (d, *J* = 7.9 Hz, 1H), 3.54 (s, 2H), 3.34 (dtd, *J* = 10.2, 7.8, 4.9 Hz, 1H), 2.86 (dd, *J* = 10.9, 4.6 Hz, 2H), 2.17 (td, *J* = 11.4, 2.6 Hz, 2H), 2.03 (ddq, *J* = 11.3, 4.8, 2.2 Hz, 2H), 1.51 (dtd, *J* = 13.9, 10.5, 3.7 Hz, 2H).

^{13}C NMR (101 MHz, $CDCl_3$) δ 149.73, 138.40, 129.26, 128.38, 127.22, 126.80, 125.12, 118.61, 112.22, 63.24, 52.30, 49.79, 32.43.

ESI-HR: calc'd for $C_{19}H_{22}F_3N_2^+$ ($[M+H]^+$) 335.1730, found 335.1741, residual 3.35 ppm

Compound 5l:

***N*-((5-methylfuran-2-yl)methyl)pyridin-3-amine**

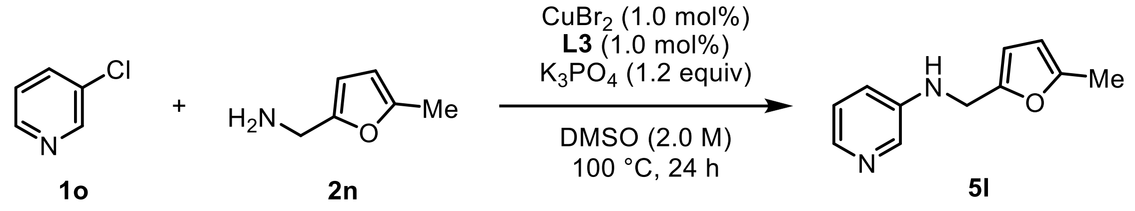

Aniline **5l** was prepared according to **GP1** from aryl chloride **1o** (114 mg, 94.7 µL, 1.00 mmol, 1.00 equiv) and amine **2n** (164 µL, 167 mg, 1.50 mmol, 1.50 equiv) in DMSO. The crude product was purified via flash column chromatography (SiO_2, EtOAc/hexanes) to give **5l** as a yellow oil.

Yield: 188 mg, 1.00 mmol, quant.

^{1}H NMR (500 MHz, $CDCl_3$) δ 8.08 (d, *J* = 2.9 Hz, 1H), 7.98 (dd, *J* = 4.7, 1.4 Hz, 1H), 7.08 (dd, *J* = 8.3, 4.7 Hz, 1H), 6.94 (ddd, *J* = 8.3, 2.9, 1.3 Hz, 1H), 6.11 (d, *J* = 3.1 Hz, 1H), 5.89 (dd, *J* = 2.9, 1.2 Hz, 1H), 4.25 (s, 2H), 4.12 (s, 1H), 2.26 (s, 3H).

^{13}C NMR (101 MHz, $CDCl_3$) δ 151.91, 149.94, 143.78, 139.19, 136.38, 123.69, 118.92, 108.29, 106.25, 41.03, 13.56.

ESI-HR: calc'd for $C_{22}H_{25}N_4O_2^+$ ($[2M+H]^+$) 377.1973, found 377.1986, residual 3.45 ppm

Compound 5m:

***N*-((1*S*,2*S*,4*R*)-bicyclo[2.2.1]heptan-2-yl)pyridin-3-amine**

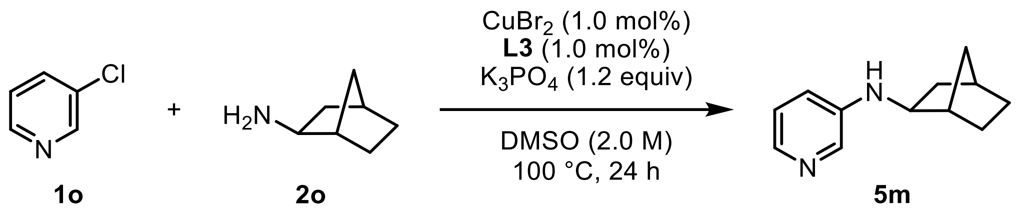

Aniline **5m** was prepared according to **GP1** from aryl chloride **1o** (94.7 µL, 114 mg, 1.00 mmol, 1.00 equiv) and amine **2o** (167 mg, 1.50 mmol, 1.50 equiv) in DMSO. The crude product was purified via flash column chromatography (SiO_2, EtOAc/hexanes) to give **5m** as a yellow solid.

Yield: 175 mg, 0.930 mmol, 93

^{1}H NMR (400 MHz, $CDCl_3$) δ 7.95 (dd, *J* = 26.9, 3.7 Hz, 2H), 7.06 (dd, *J* = 8.3, 4.7 Hz, 1H), 6.89 – 6.74 (m, 1H), 3.63 (s, 1H), 3.21 (p, *J* = 4.1 Hz, 1H), 2.48 – 2.19 (m, 2H), 1.84 (ddd, *J* = 12.9, 7.6, 2.5 Hz, 1H), 1.62 – 1.34 (m, 3H), 1.34 – 1.05 (m, 4H).

^{13}C NMR (101 MHz, $CDCl_3$) δ 143.63, 138.34, 136.40, 123.71, 118.83, 56.40, 41.18, 41.03, 35.72, 35.44, 28.46, 26.38.

ESI-HR: calc'd for $C_{24}H_{33}N_4^+$ ($[2M+H]^+$) 377.2699, found 377.2717, residual 4.77 ppm

Compound 5n:

***N*-(3-(vinyloxy)propyl)pyridin-3-amine**

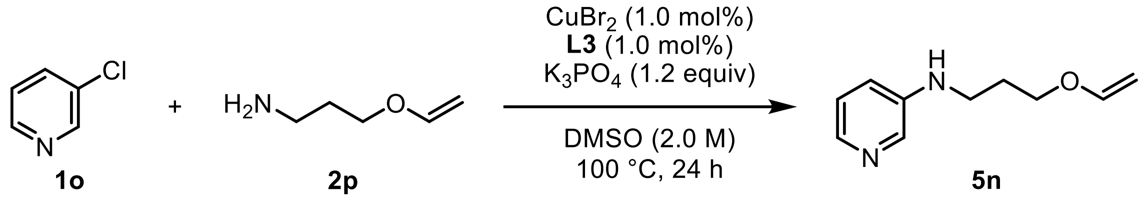

Aniline **5n** was prepared according to **GP1** from aryl chloride **1o** (94.7 µL, 114 mg, 1.00 mmol, 1.00 equiv) and amine **2p** (170 µL, 152 mg, 1.50 mmol, 1.50 equiv) in DMSO. The crude product was purified via flash column chromatography (SiO_2, EtOAc/hexanes) to give **5n** as a yellow solid.

Yield: 160 mg, 0.895 mmol, 90%

^{1}H NMR (400 MHz, $CDCl_3$) δ 8.10 – 7.87 (m, 2H), 7.07 (dd, *J* = 8.3, 4.7 Hz, 1H), 6.87 (ddd, *J* = 8.3, 2.9, 1.3 Hz, 1H), 6.48 (dd, *J* = 14.3, 6.8 Hz, 1H), 4.20 (dd, *J* = 14.3, 2.1 Hz, 1H), 4.03 (dd, *J* = 6.8, 2.1 Hz, 1H), 3.93 (s, 1H), 3.81 (t, *J* = 5.8 Hz, 2H), 3.28 (q, *J* = 4.6 Hz, 2H), 1.98 (p, *J* = 6.3 Hz, 2H).

^{13}C NMR (101 MHz, $CDCl_3$) δ 151.65, 144.33, 138.65, 136.09, 123.80, 118.41, 86.96, 65.92, 40.96, 28.68.

ESI-HR: calc'd for $C_{18}H_{25}N_4O_2^+$ ($[2M+H]^+$) 357.2285, found 357.2271, residual 3.92 ppm

11. High Turnover Number Reaction

Compound 3w:

***N*-benzyl-4-(trifluoromethyl)aniline**

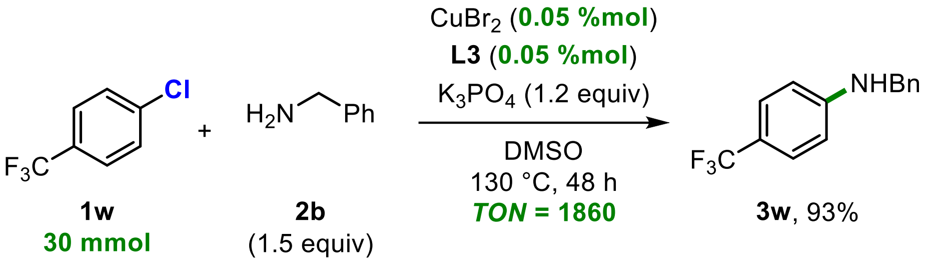

In an N_2-filled glovebox, **L3** (0.0150 mmol, 12.0 mg, 0.0500 mol%) and K_3PO_4 (36.0 mmol, 7.64 g, 1.20 equiv) were weighed into an oven-dried 50.0 mL round-bottom flask equipped with a stir bar. $CuBr_2$ (0.015 mmol, 3.4 mg, 0.050 mmol%) was then added, followed by 1-chloro-4-(trifluoromethyl)benzene (30.0 mmol, 5.42 mg, 1.00 equiv), benzylamine (45.0 mmol, 4.82 g, 1.5 equiv), and DMSO (15.0 mL). The flask was capped, removed from the glovebox, placed in an oil bath at 130 °C, and stirred for 48 h. Upon completion of the reaction, the mixture was cooled to room temperature, and 20 mL of water and 50 mL of ethyl acetate were added. The phases were separated, and the aqueous layer was further extracted with ethyl acetate (30 mL x 3). The combined organic phases were dried over Na_2SO_4, concentrated in vacuo, and the crude product was directly purified by silica gel column chromatography using EtOAc/hexane as the eluent to afford the corresponding product **3w** in 93% yield.

^{1}H NMR (500 MHz, $CDCl_3$) δ 7.40 (d, *J* = 8.5 Hz, 2H), 7.36 (s, 4H), 7.33 – 7.28 (m, 1H), 6.64 (d, *J* = 8.4 Hz, 2H), 4.43 (s, 1H), 4.38 (s, 2H).

^{13}C NMR (101 MHz, $CDCl_3$) δ 150.60, 138.59, 128.92, 127.66, 127.49, 126.76, 125.11, 119.17, 112.12, 47.93.

^{19}F NMR (376 MHz, $CDCl_3$) δ -60.84.

Compound 3t:

***N*-benzylpyridin-3-amine**

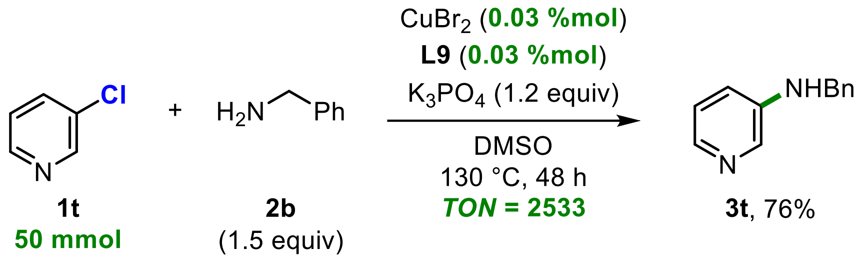

In an N_2-filled glovebox, **L9** (0.015 mmol, 12.0 mg, 0.05 mol%) and K_3PO_4 (60.0 mmol, 12.7 g, 1.2 equiv) were weighed into an oven-dried 50.0 mL round-bottom flask equipped with a stir bar. $CuBr_2$ (0.015 mmol, 3.4 mg, 0.05 mmol%) was then added, followed by 3-chloropyridine (50.0 mmol, 5677.0 mg, 1.0 equiv), benzylamine (75.0 mmol, 8037.0 mg, 1.5 equiv), and DMSO (15.0 mL). The flask was capped, removed from the glovebox, placed in an oil bath set at 130 °C, and stirred for 48 h. Upon completion of the reaction, the mixture was cooled to room temperature, and 30 mL of water and 60 mL of ethyl acetate were added. The phases were separated, and the aqueous layer was further extracted with ethyl acetate (40 mL × 3). The combined organic phases were dried over Na_2SO_4, concentrated in vacuo, and the crude product was directly purified by silica gel column chromatography using EtOAc/hexane as the eluent to afford the corresponding product 3t.

Compound 6c:

3,5-dimethoxyaniline

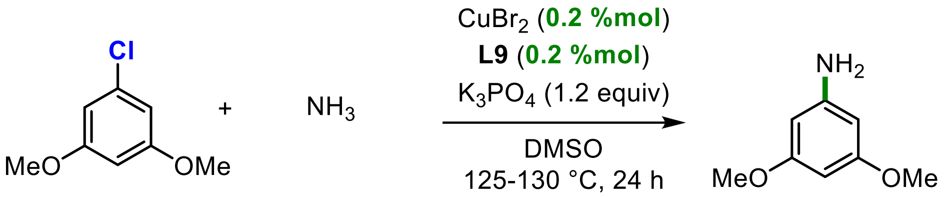

1m 50 mmol | **7** 1 atm | **6c**, 74%

In an N_2-filled glove-filled glovebox, **L9** (0.015 mmol, 12.0 mg, 0.05 mol%), $CuBr_2$ (0.015 mmol, 3.4 mg, 0.05 mmol%), 1-chloro-3,5-dimethoxybenzene(50.0 mmol, 8630.5 mg, 1.0 equiv) and K_3PO_4 (60.0 mmol, 12.7 g, 1.2 equiv) were weighed into an oven-dried 50.0 mL Schlenk flask equipped with a stir. The flask was capped, removed from the glovebox, and subjected to evacuation and backfilling with ammonia (1 atm, via balloon) for 4 cycles. DMSO (15.0 mL) was then added, and the flask was placed in an oil bath set at 130 °C and stirred for 24 h. Upon completion of the reaction, 1,3,5-trimethoxybenzene(30.0 mmol,5045.7 mg, 1H NMR internal) was added. Aliquots of the reaction mixtures were diluted in $CDCl_3$ and yields were determined by ^1H NMR.

12. Sequential Amination of Aryl dihalides

Compound 3v-2:

N[1]-benzyl-5-fluoro-*N*[3]-hexylbenzene-1,3-diamine

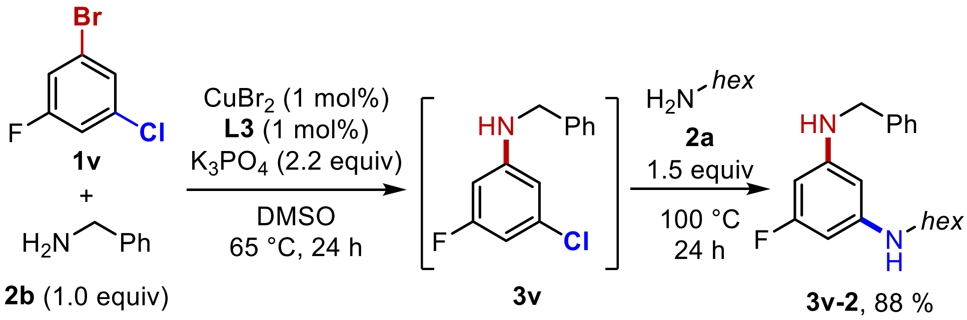

In an N_2-filled glovebox, **L3** (0.10 mmol, 80.0 mg, 1.0 mol%) and K_3PO_4 (22 mmol, 4.70 g, 2.2 equiv) were weighed into an oven-dried 20.0 mL round-bottom flask equipped with a stir bar. $CuBr_2$ (0.10 mmol, 22.3 mg, 1.0 mol%) was then added, followed by 1-bromo-3-chloro-5-fluorobenzene (2094.4 mg, 10.0 mmol, 1 equiv), benzylamine (1071.6 mg, 10.0 mmol, 1.0 equiv), and DMSO (10.0 mL). The flask was capped, removed from the glovebox, placed in an oil bath set at 65 °C, and stirred for 24 h. Hexylamine (15.0 mmol, 1518 mg, 1.5 equiv) was subsequently added, and the reaction was heated to 100 °C for another 24 h. Upon completion, the mixture was cooled to room temperature, and 20 mL of water and 50 mL of ethyl acetate were added. The phases were separated, and the aqueous layer was further extracted with ethyl acetate (30 mL × 3). The combined organic phases were dried over Na_2SO_4, concentrated in vacuo, and the crude product was directly purified by silica gel column chromatography using EtOAc/hexane as the eluent to afford the corresponding product **3v-2**.

Purification was conducted via flash chromatography (SiO2, EtOAc/hexanes) to give **3az** as a yellow oil in 88% yield (3.0g, 0.88 mmol)

^{1}H NMR (500 MHz, $CDCl_3$) δ 7.39 – 7.32 (m, 4H), 7.28 (ddt, J = 8.6, 5.6, 2.9 Hz, 1H), 5.82 – 5.69 (m, 2H), 5.63 (t, J = 2.0 Hz, 1H), 4.28 (s, 2H), 3.87 (s, 2H), 3.03 (t, J = 7.2 Hz, 2H), 1.63 – 1.51 (m, 2H), 1.45 – 1.25 (m, 6H), 0.91 (t, J = 6.8 Hz, 3H).

^{13}C NMR (101 MHz, $CDCl_3$) δ 165.34, 150.86, 150.48, 139.37, 128.78, 127.61, 127.41, 92.55, 89.80, 89.62, 48.39, 44.05, 31.75, 29.58, 26.94, 22.75, 14.17.

ESI-HR: calc'd for $C_{19}H_{25}FN_2^+$ ($[M+H]^+$) 301.2075, found 301.2063, residual 3.98 ppm

13. Synthesis of Cu(I) and Cu(II) Complexes

Cu(I) Complex Cu-B

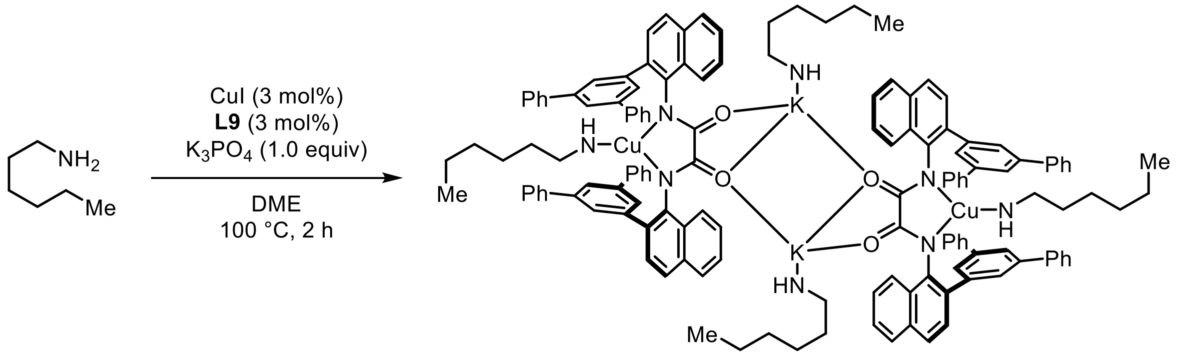

In an N_2-filled glovebox, a 4 mL scintillation vial containing a Teflon-coated stir bar was charged with CuI (23.9 mg, 125 µmol), **L3** (100 mg, 125 µmol), and K_3PO_4 (799 mg, 3.76 mmol). Then *n*-Hexylamine (151.8 mg, 1.5 mmol, 1.5 equiv) followed by 0.500 mL of DME were added. The vial was sealed with a Teflon-lined cap and electrical tape and the reaction was stirred at 100 °C for 2 h. After this time, the vial was returned to the glovebox, and the reaction was filtered through a frit while still warm. A red solid precipitated within one hour but the solution was left for slow evaporation in the glove box for an additional week to ensure crystal formation.

Synthesis of Cu(II) Complex Cu-A

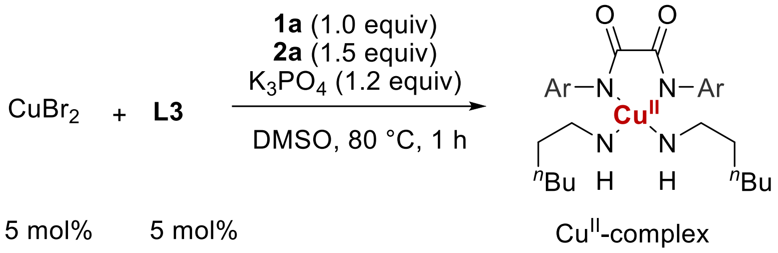

In an N_2-filled glovebox, a 4 mL scintillation vial containing a Teflon-coated stir bar was charged with $CuBr_2$ (11.2 mg, 0.05 mmol, 5.0 mol%), **L3** (39.8 mg, 0.05 mmol, 5 mol%), and K_3PO_4 (256.0 mg, 1.2 mmol, 1.2 equiv). 1-Hexanamine (151.8 mg, 1.5 mmol, 1.5 equiv) and 1-chloro-4-methoxybenzene (142.3 mg, 1.0 mmol, 1.0 equiv) were added, followed by 1.0 mL of DMSO. The vial was sealed with a Teflon-lined cap, and the reaction was stirred at 80 °C for 60 min. Upon completion, the vial was returned to the glovebox, cooled to room temperature, and filtered through a PTFE syringe filter (0.22 μm pore size) into an NMR tube. Dark red crystals precipitated after the NMR tube was left at room temperature for three days.

14. ^{1}H, ^{13}C, and ^{19}F NMR Spectra

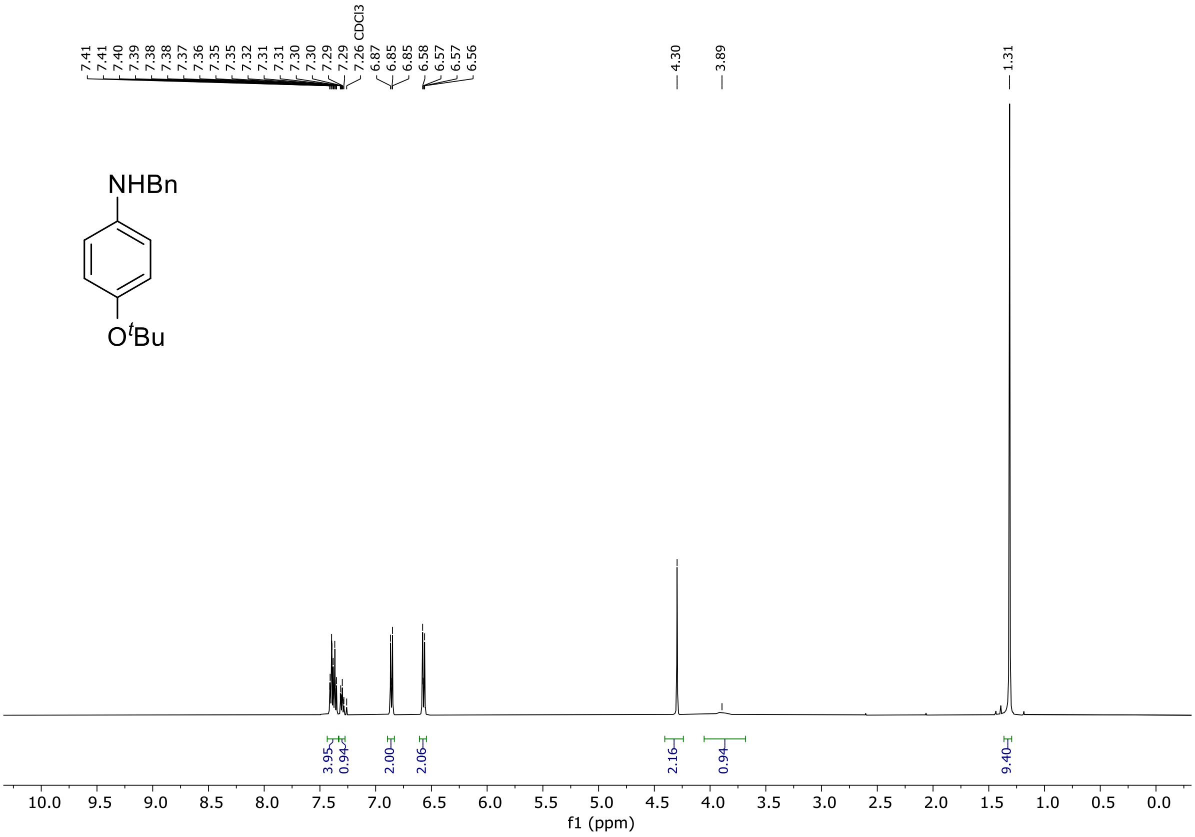

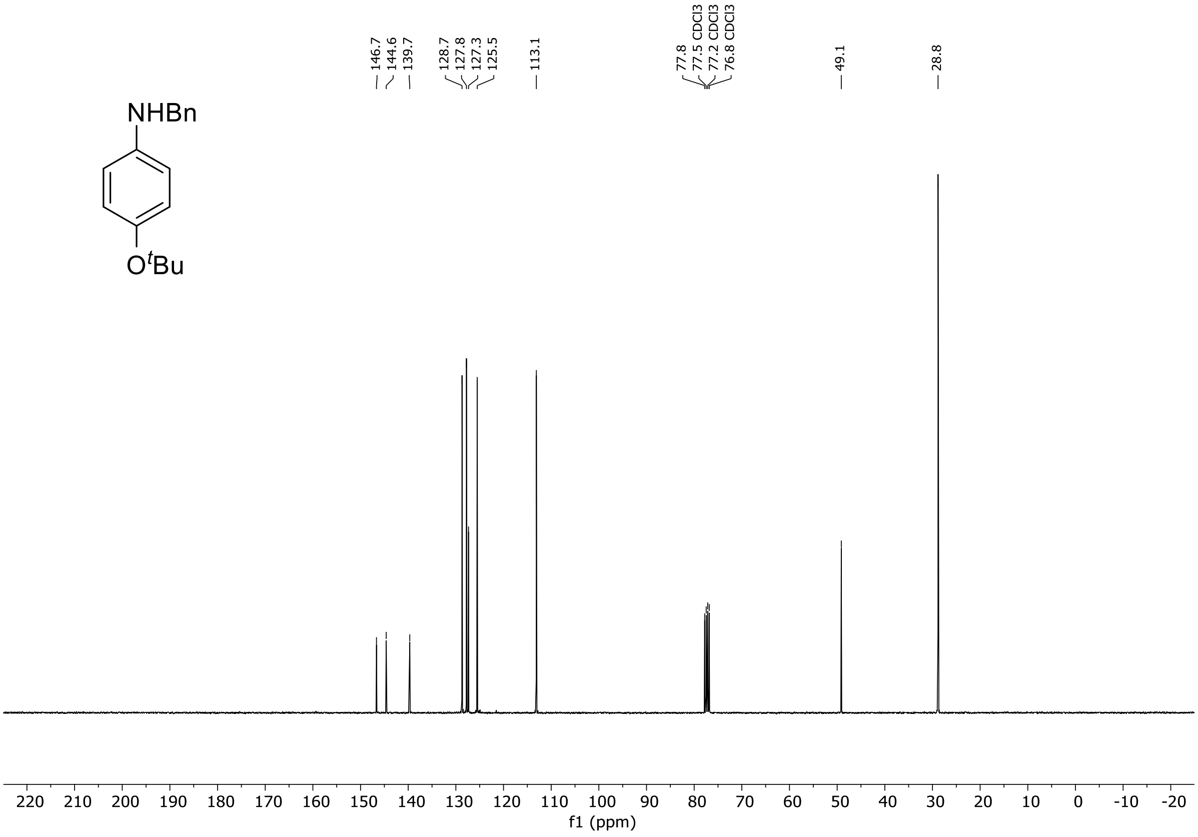

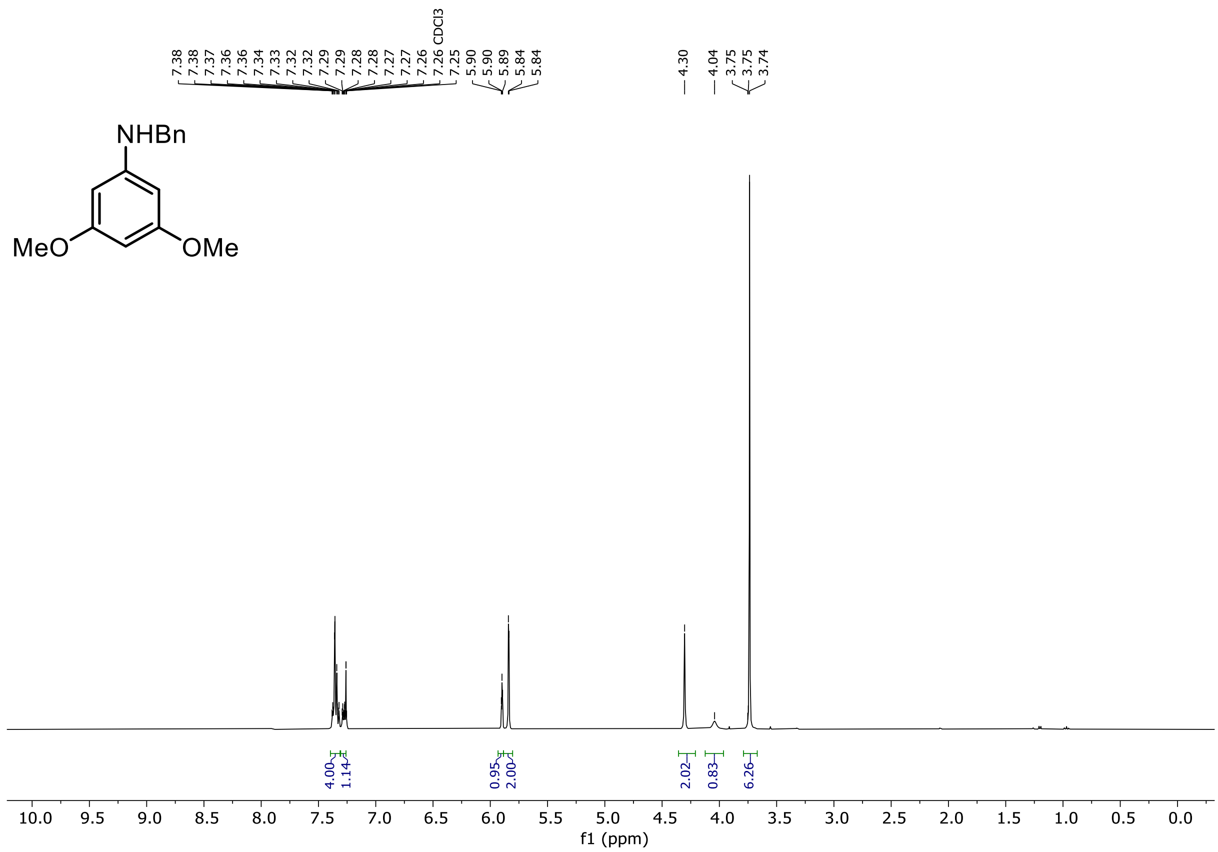

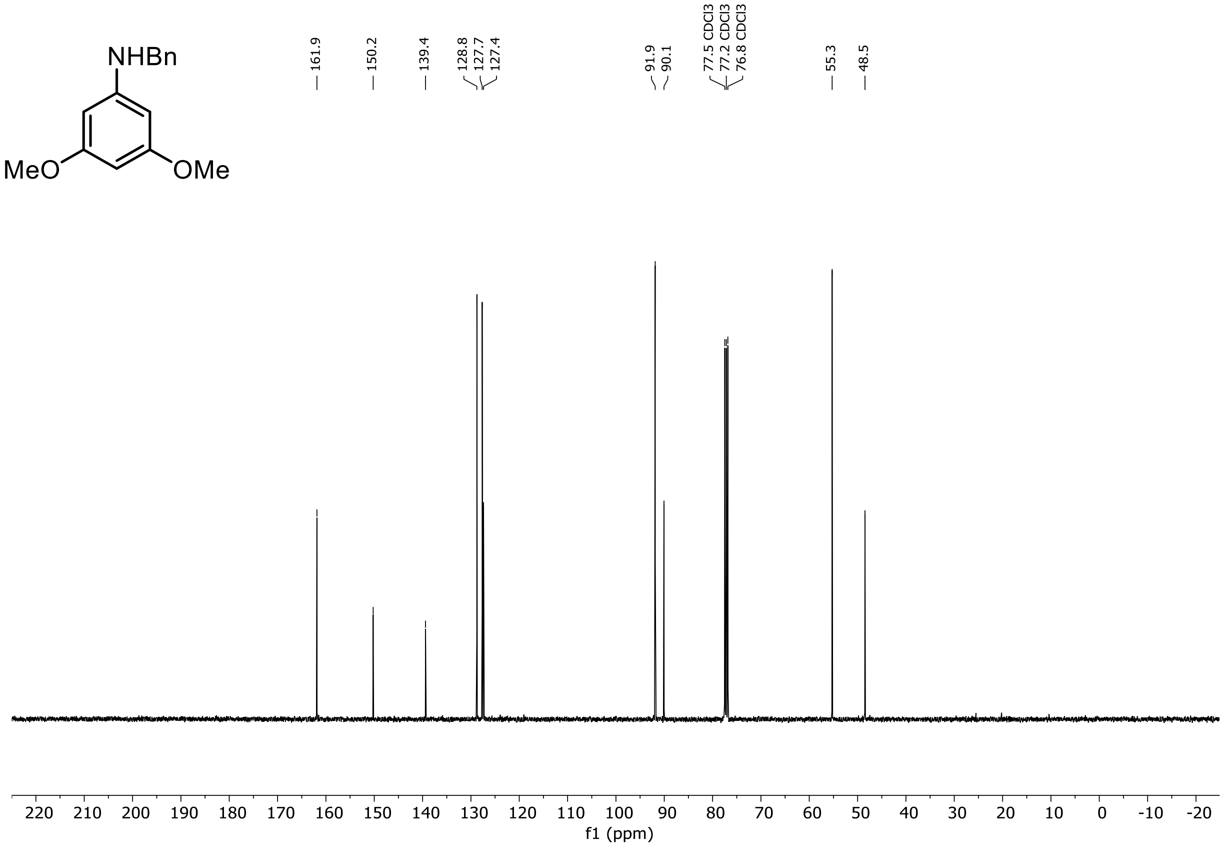

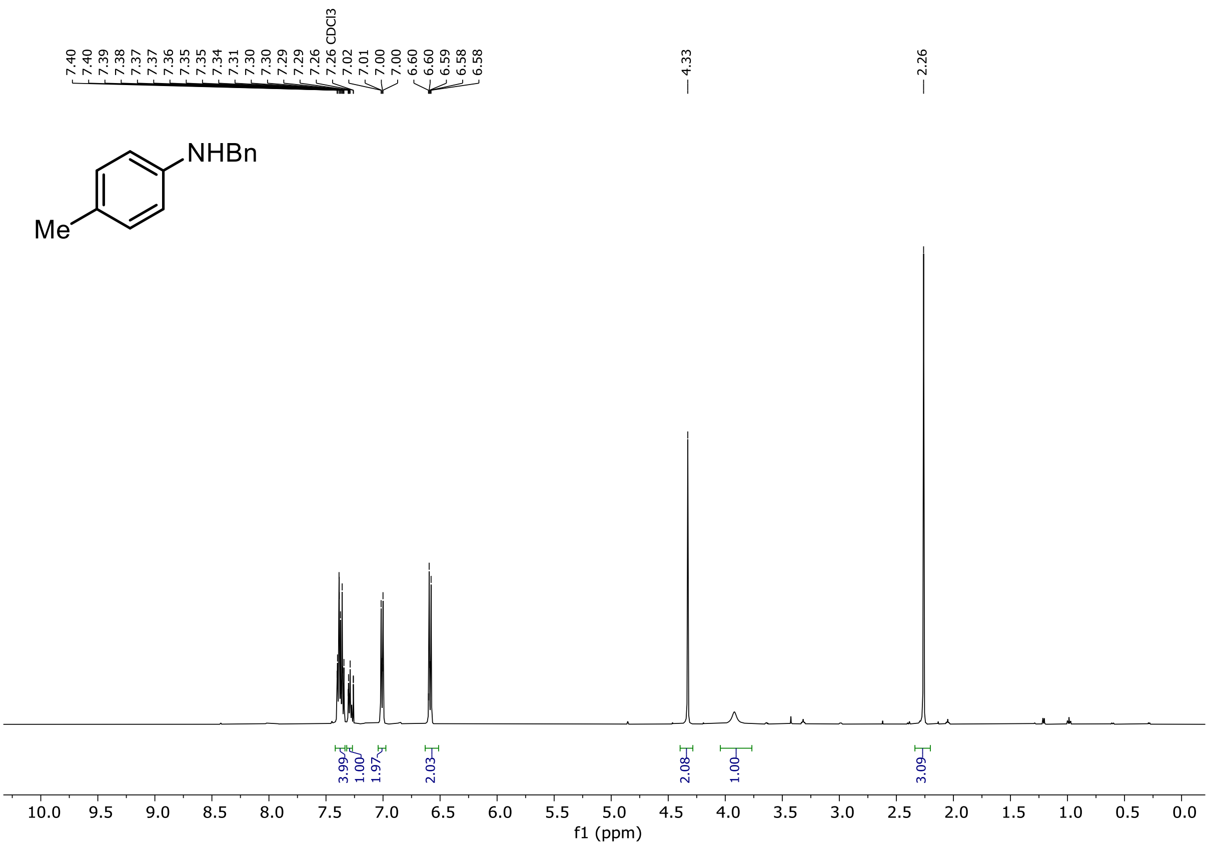
7.40
7.40
7.39
7.38
7.37
7.37
7.36
7.35
7.35
7.34
7.31
7.30
7.30
7.29
7.29
7.26
7.26 CDCl3
7.02
7.01
7.00
7.00
6.60
6.60
6.59
6.58
6.58
4.33
2.26
NHBn
Me
3.99
1.00
1.97
2.03
2.08
1.00
3.09
f1 (ppm)

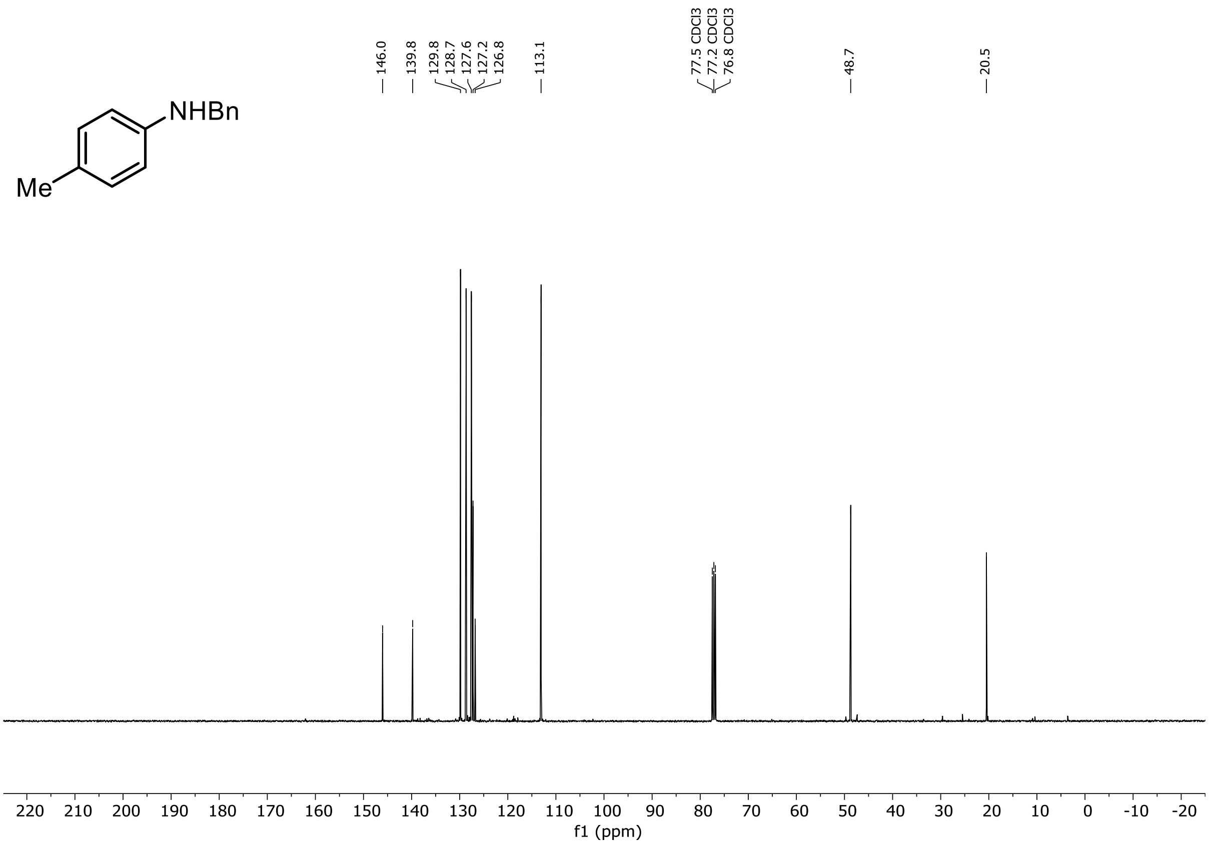
146.0
139.8
129.8
128.7
127.6
127.2
126.8
113.1
77.5 CDCl3
77.2 CDCl3
76.8 CDCl3
48.7
20.5
NHBn
Me
f1 (ppm)

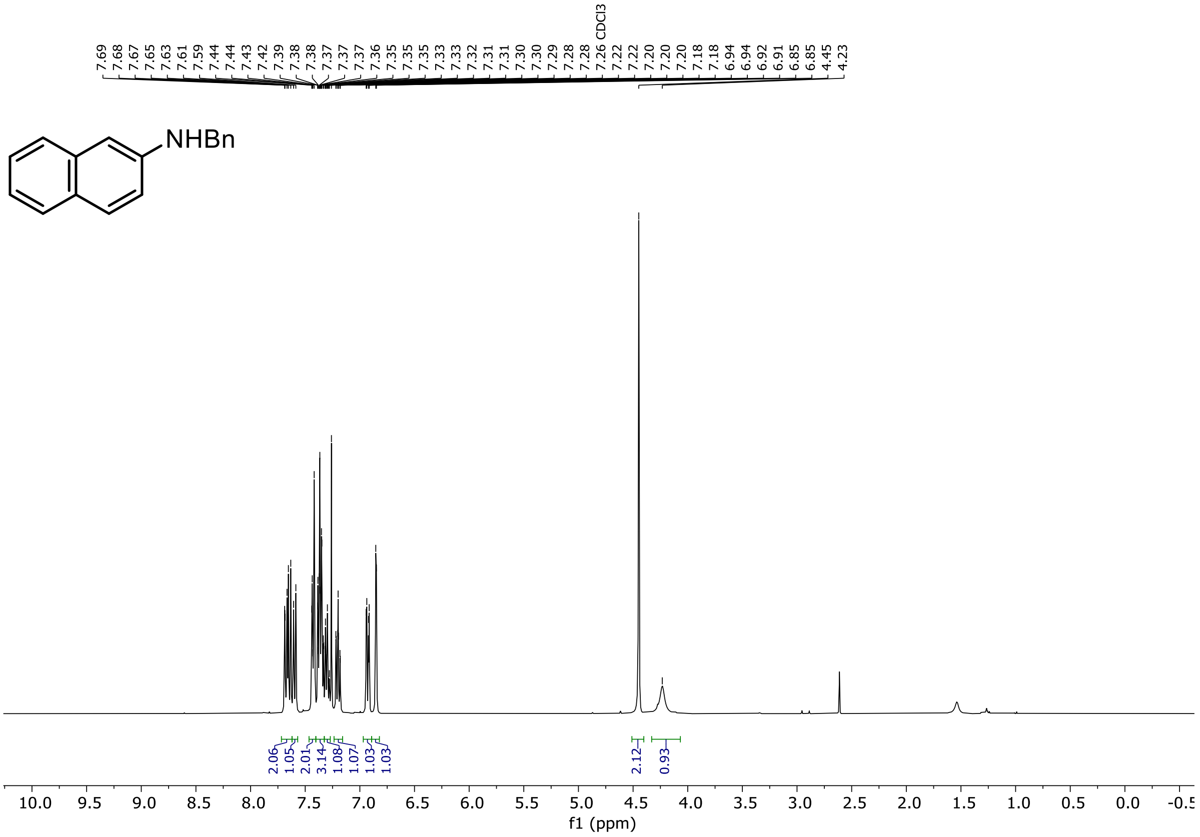
NHBn
7.69 7.68 7.67 7.65 7.63 7.61 7.59 7.44 7.44 7.43 7.42 7.39 7.38 7.38 7.37 7.37 7.37 7.36 7.35 7.35 7.35 7.33 7.33 7.32 7.31 7.31 7.30 7.30 7.29 7.28 7.28 7.26 CDCl3 7.22 7.22 7.22 7.20 7.20 7.20 7.18 7.18 6.94 6.94 6.92 6.91 6.85 6.85 4.45 4.23
2.06 1.05 2.01 3.14 1.08 1.07 1.03 1.03 2.12 0.93
f1 (ppm)

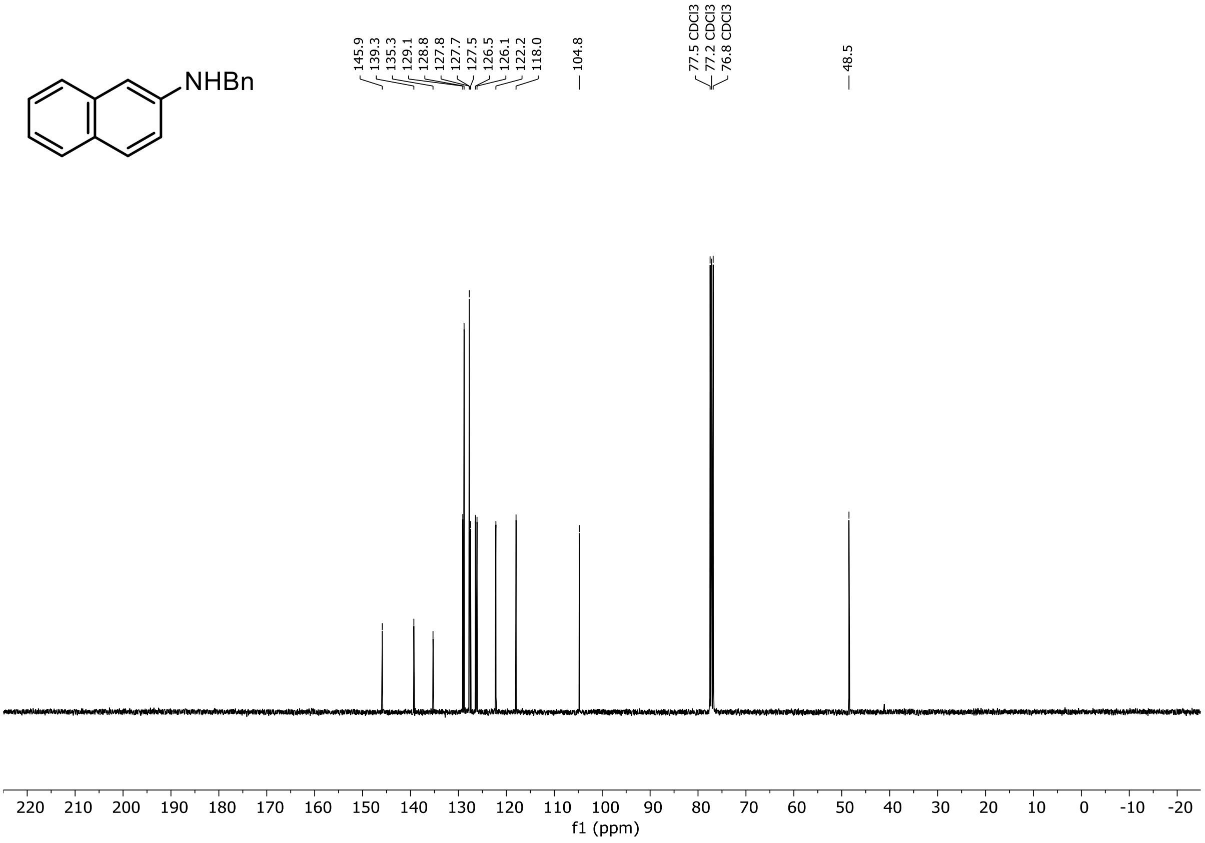
NHBn
145.9 139.3 135.3 129.1 128.8 127.8 127.7 127.5 126.5 126.1 122.2 118.0 104.8
77.5 CDCl3 77.2 CDCl3 76.8 CDCl3
48.5
f1 (ppm)

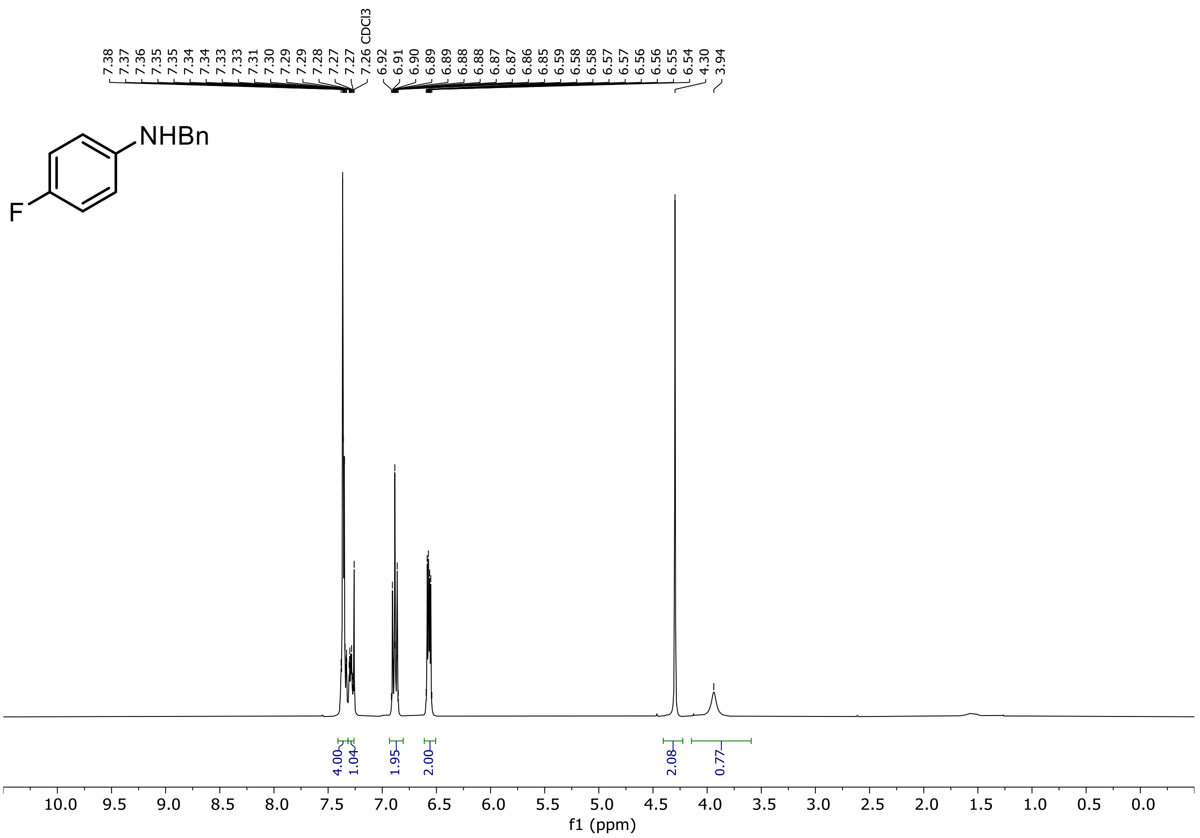
NHBn
F
7.38
7.37
7.36
7.35
7.35
7.34
7.34
7.33
7.33
7.31
7.30
7.29
7.29
7.28
7.27
7.27
7.26 CDCl3
6.92
6.91
6.90
6.89
6.89
6.88
6.88
6.87
6.87
6.86
6.85
6.59
6.58
6.58
6.57
6.57
6.56
6.56
6.55
6.54
4.30
3.94
4.00
1.04
1.95
2.00
2.08
0.77
f1 (ppm)

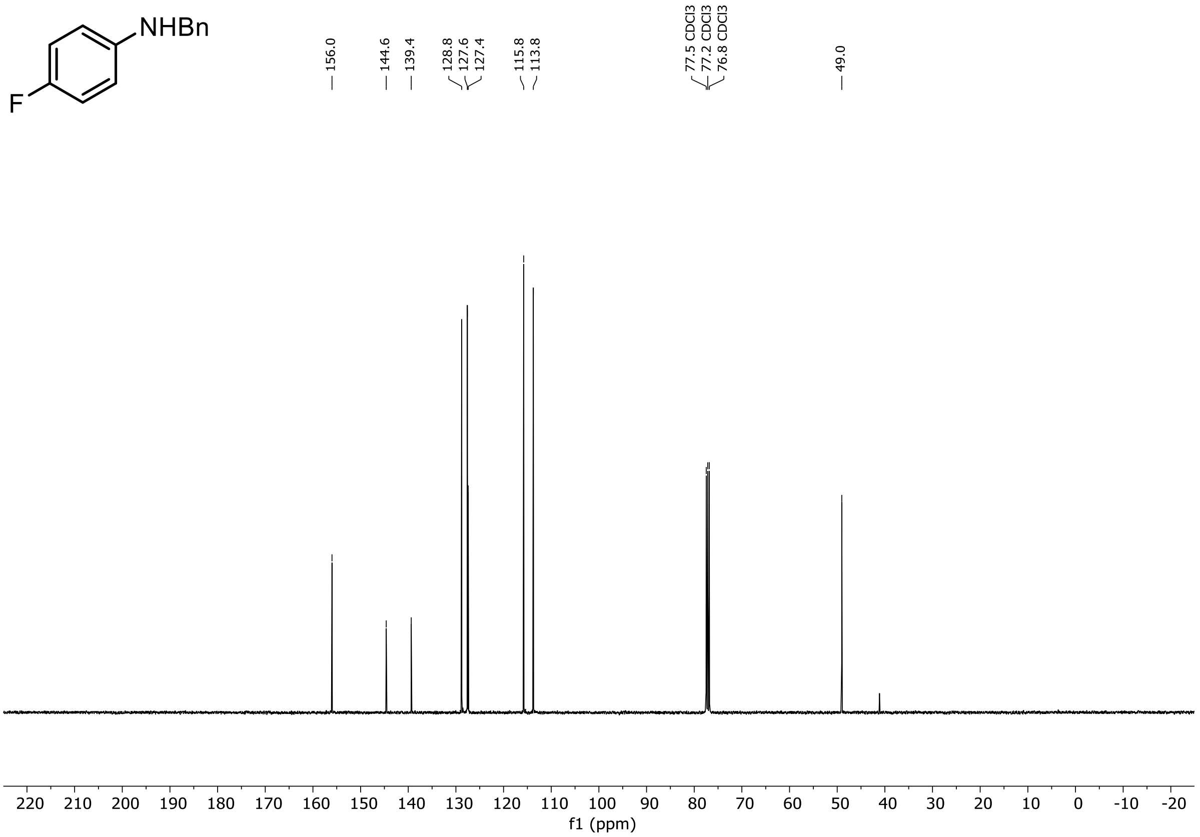
NHBn
F
156.0
144.6
139.4
128.8
127.6
127.4
115.8
113.8
77.5 CDCl3
77.2 CDCl3
76.8 CDCl3
49.0
f1 (ppm)

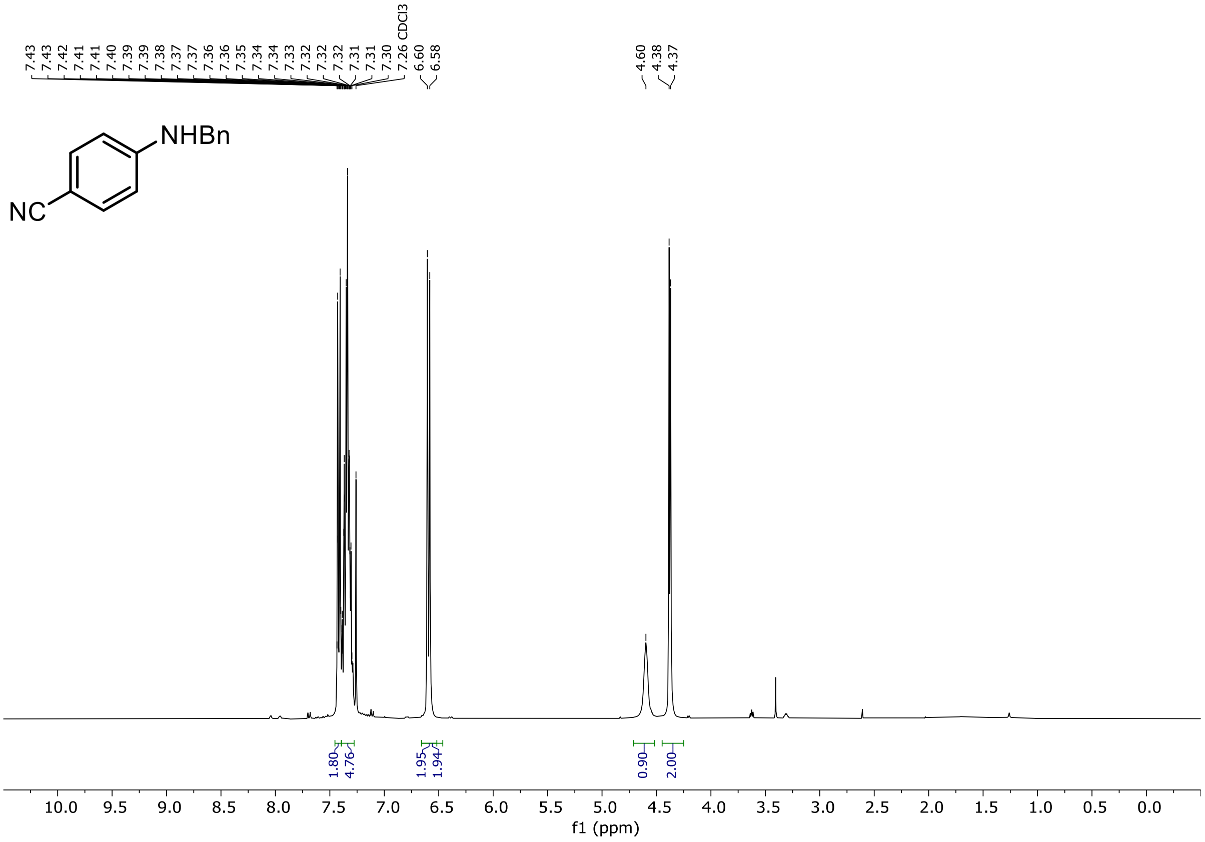
7.43
7.43
7.42
7.41
7.41
7.40
7.39
7.39
7.38
7.37
7.37
7.36
7.36
7.35
7.34
7.34
7.33
7.32
7.32
7.32
7.31
7.31
7.30
7.26 CDCl3
6.60
6.58
4.60
4.38
4.37
NHBn
NC
1.80
4.76
1.95
1.94
0.90
2.00
10.0 9.5 9.0 8.5 8.0 7.5 7.0 6.5 6.0 5.5 5.0 4.5 4.0 3.5 3.0 2.5 2.0 1.5 1.0 0.5 0.0
f1 (ppm)

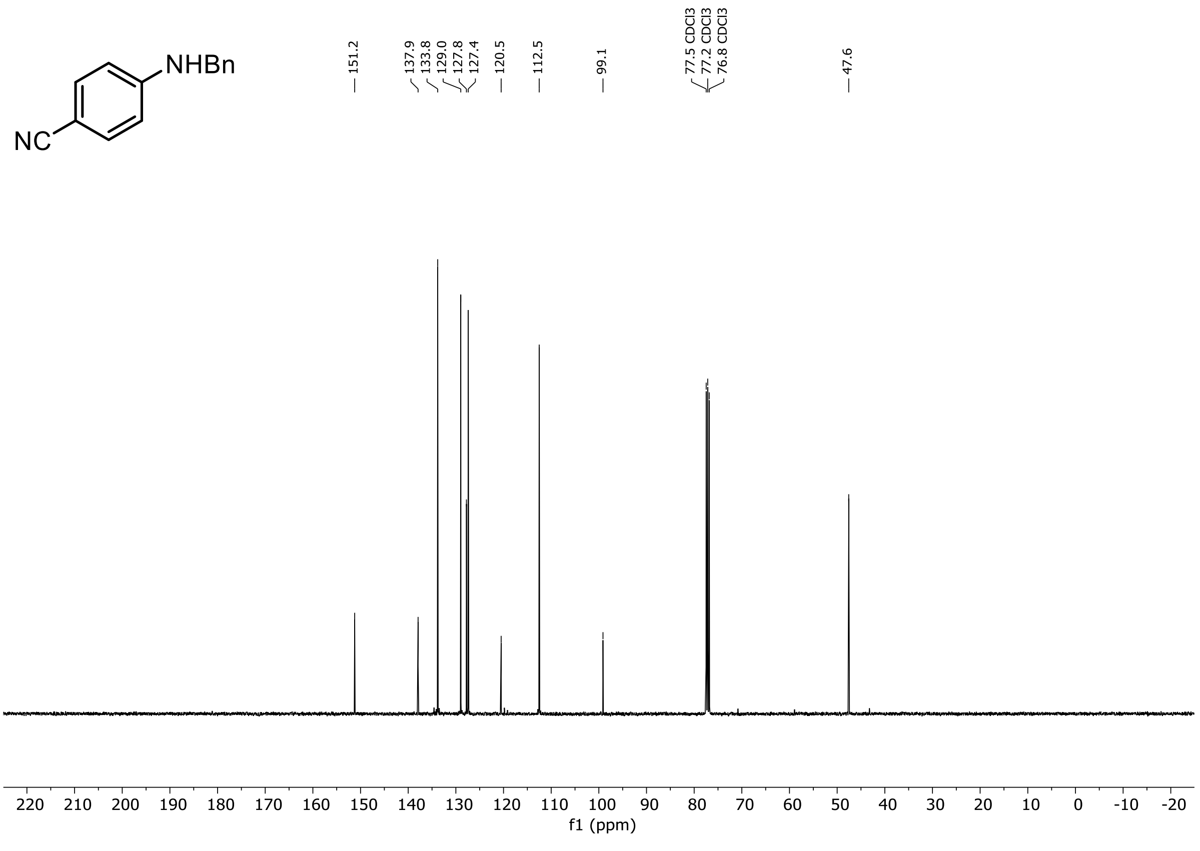
NHBn
NC
151.2
137.9
133.8
129.0
127.8
127.4
120.5
112.5
99.1
77.5 CDCl3
77.2 CDCl3
76.8 CDCl3
47.6
220 210 200 190 180 170 160 150 140 130 120 110 100 90 80 70 60 50 40 30 20 10 0 -10 -20
f1 (ppm)

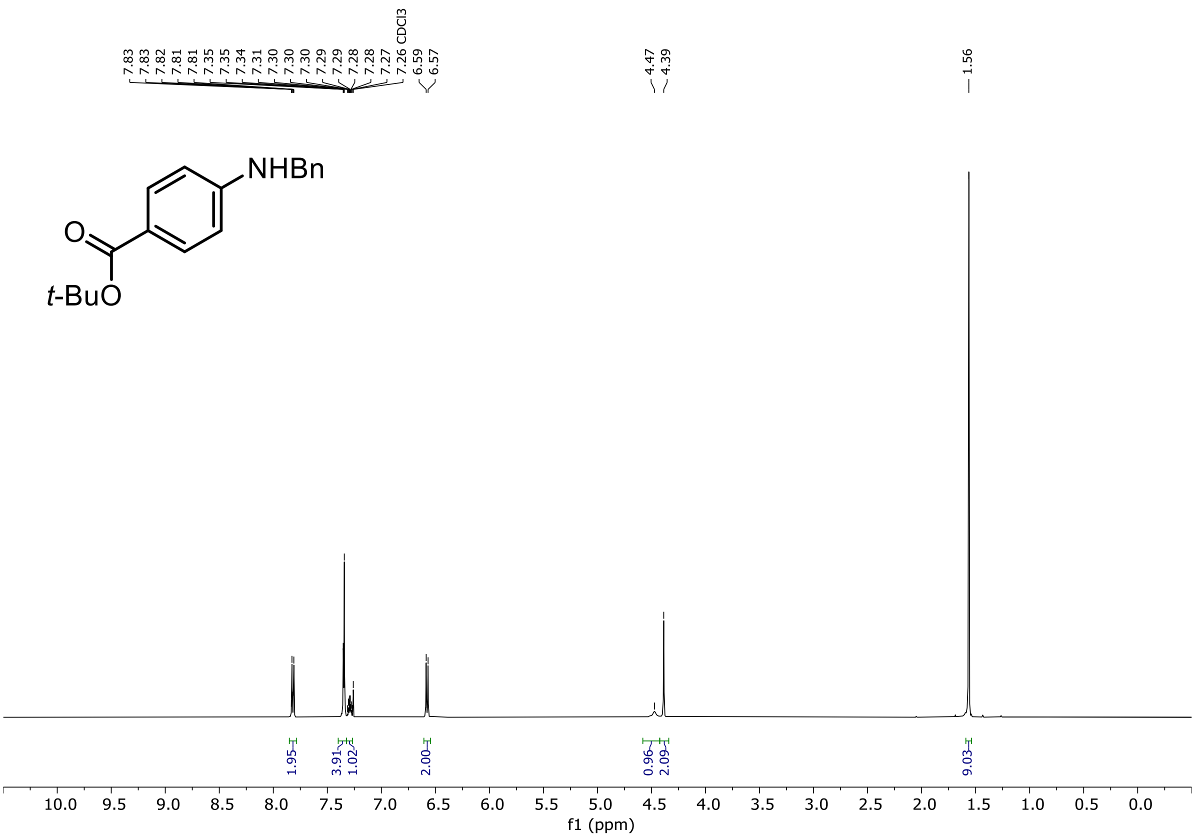
7.83
7.83
7.82
7.81
7.81
7.35
7.35
7.34
7.31
7.30
7.30
7.30
7.29
7.29
7.28
7.28
7.27
7.26 CDCl3
6.59
6.57
4.47
4.39
1.56
NHBn
O
t-BuO
1.95
3.91
1.02
2.00
0.96
2.09
9.03
10.0 9.5 9.0 8.5 8.0 7.5 7.0 6.5 6.0 5.5 5.0 4.5 4.0 3.5 3.0 2.5 2.0 1.5 1.0 0.5 0.0
f1 (ppm)

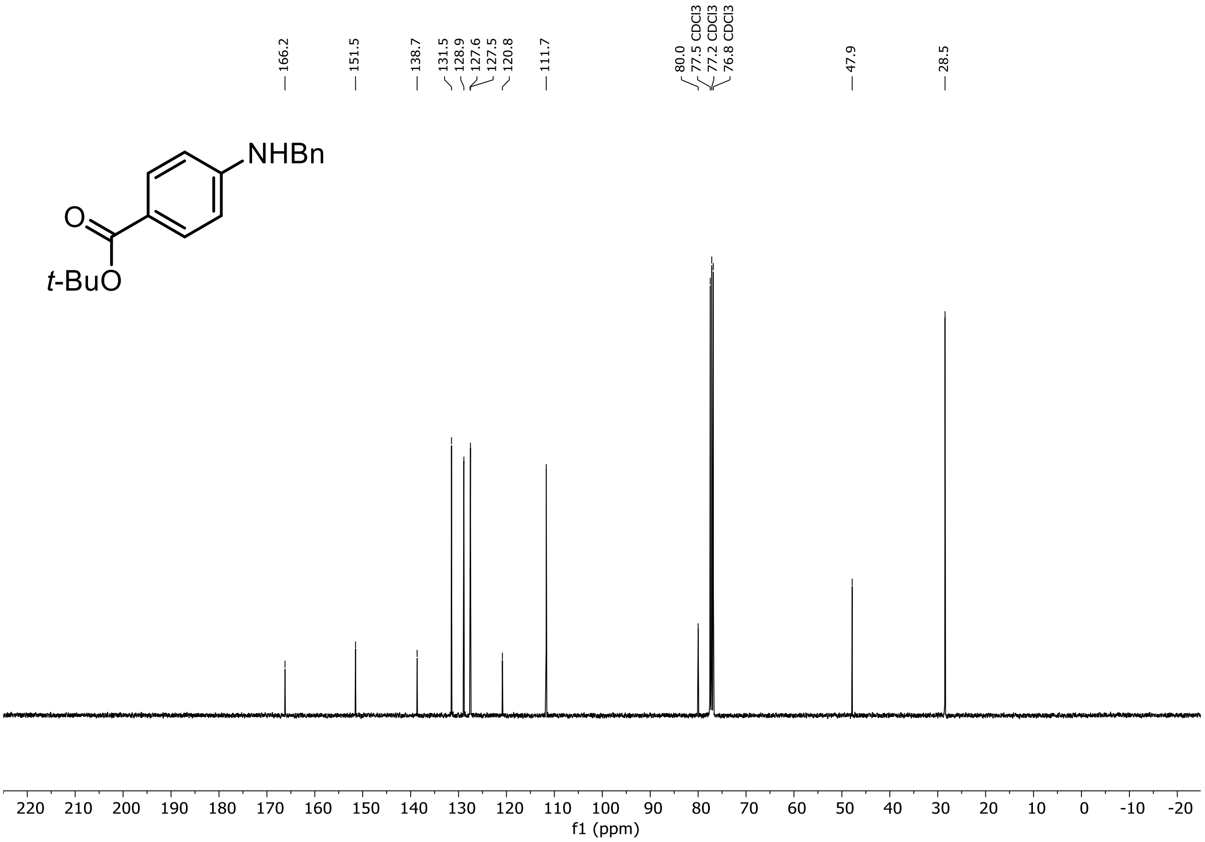
166.2
151.5
138.7
131.5
128.9
127.6
127.5
120.8
111.7
80.0
77.5 CDCl3
77.2 CDCl3
76.8 CDCl3
47.9
28.5
NHBn
O
t-BuO
220 210 200 190 180 170 160 150 140 130 120 110 100 90 80 70 60 50 40 30 20 10 0 -10 -20
f1 (ppm)

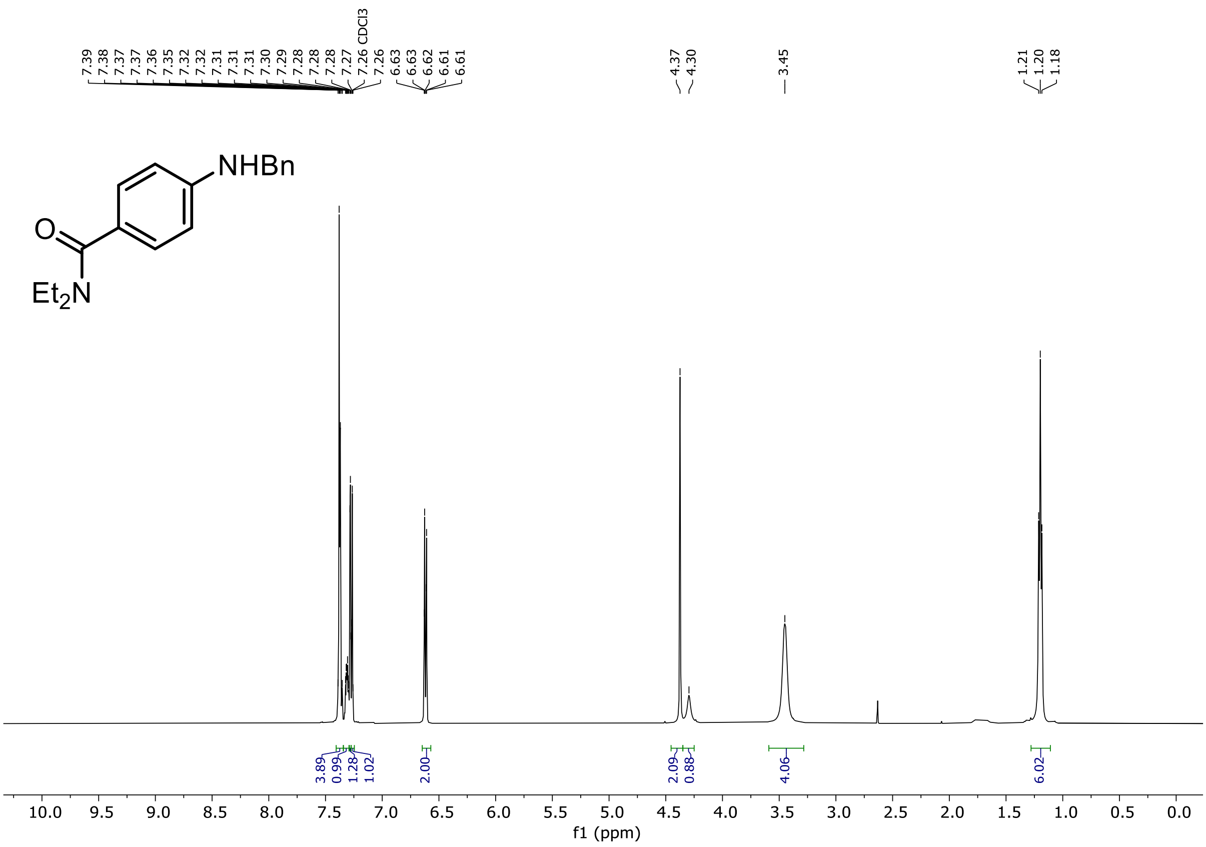
NHBn
O
Et_2N
7.39 7.38 7.37 7.37 7.36 7.35 7.32 7.32 7.31 7.31 7.31 7.30 7.29 7.28 7.28 7.28 7.27 7.26 CDCl3 7.26
6.63 6.63 6.62 6.61 6.61
4.37 4.30
3.45
1.21 1.20 1.18
3.89 0.99 1.28 1.02 2.00 2.09 0.88 4.06 6.02
10.0 9.5 9.0 8.5 8.0 7.5 7.0 6.5 6.0 5.5 5.0 4.5 4.0 3.5 3.0 2.5 2.0 1.5 1.0 0.5 0.0
f1 (ppm)

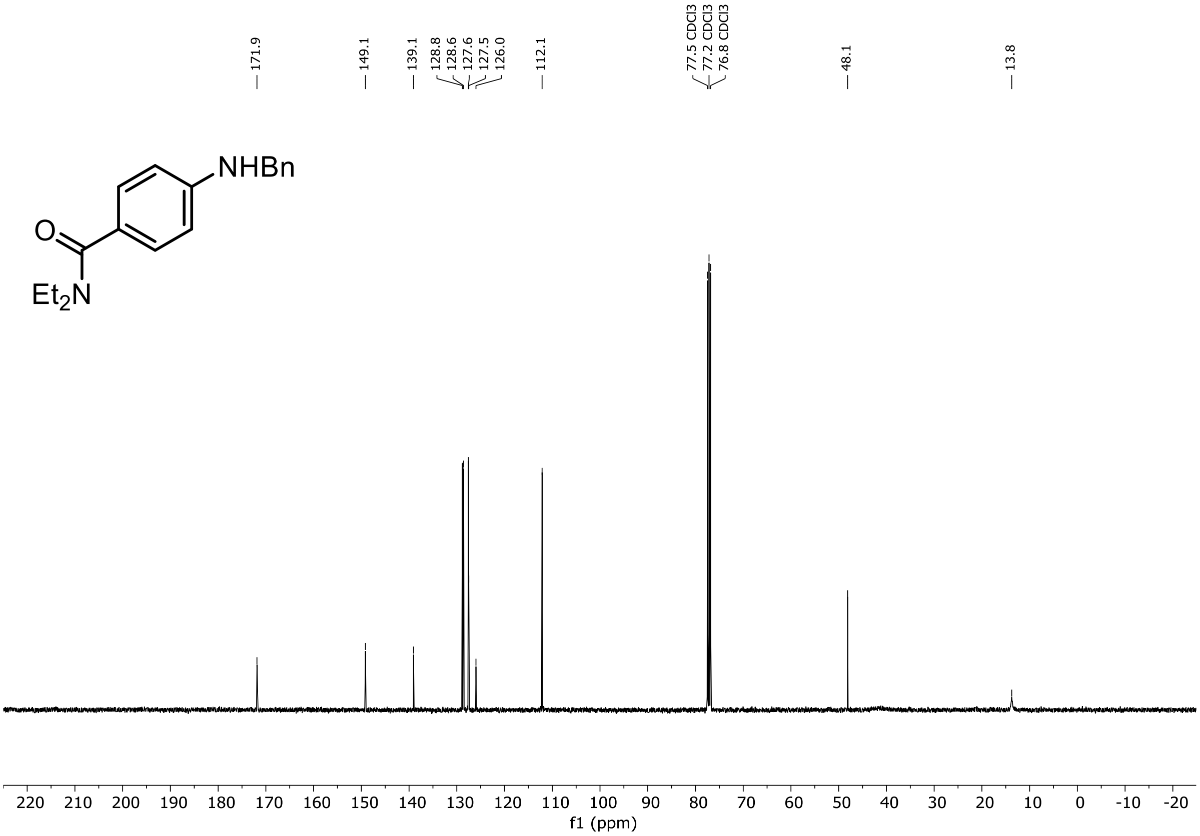
NHBn
O
Et_2N
171.9
149.1
139.1
128.8 128.6 127.6 127.5 126.0
112.1
77.5 CDCl3 77.2 CDCl3 76.8 CDCl3
48.1
13.8
220 210 200 190 180 170 160 150 140 130 120 110 100 90 80 70 60 50 40 30 20 10 0 -10 -20
f1 (ppm)

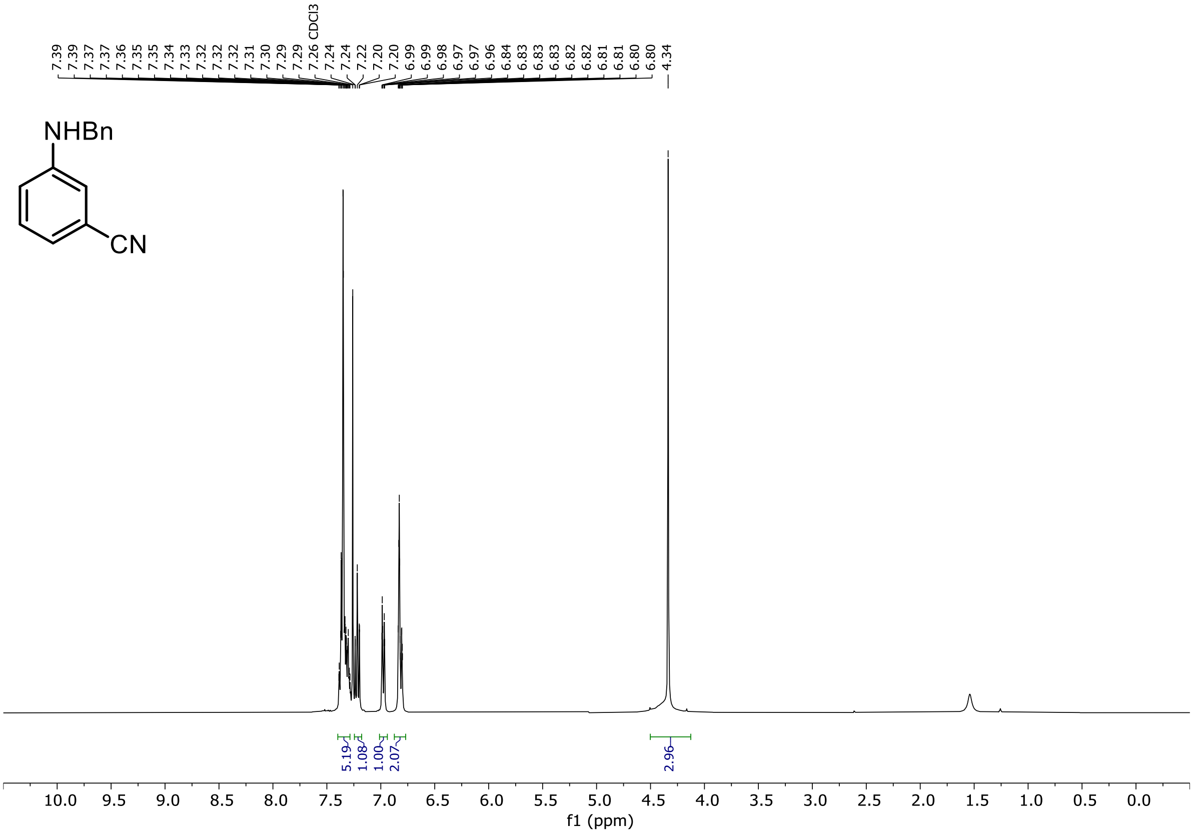
NHBn
CN
7.39
7.39
7.37
7.37
7.36
7.35
7.35
7.34
7.33
7.32
7.32
7.32
7.31
7.30
7.29
7.29
7.26 CDCl3
7.24
7.24
7.22
7.20
7.20
6.99
6.99
6.98
6.97
6.97
6.96
6.84
6.83
6.83
6.83
6.82
6.81
6.81
6.80
6.80
4.34
5.19
1.08
1.00
2.07
2.96
10.0 9.5 9.0 8.5 8.0 7.5 7.0 6.5 6.0 5.5 5.0 4.5 4.0 3.5 3.0 2.5 2.0 1.5 1.0 0.5 0.0
f1 (ppm)

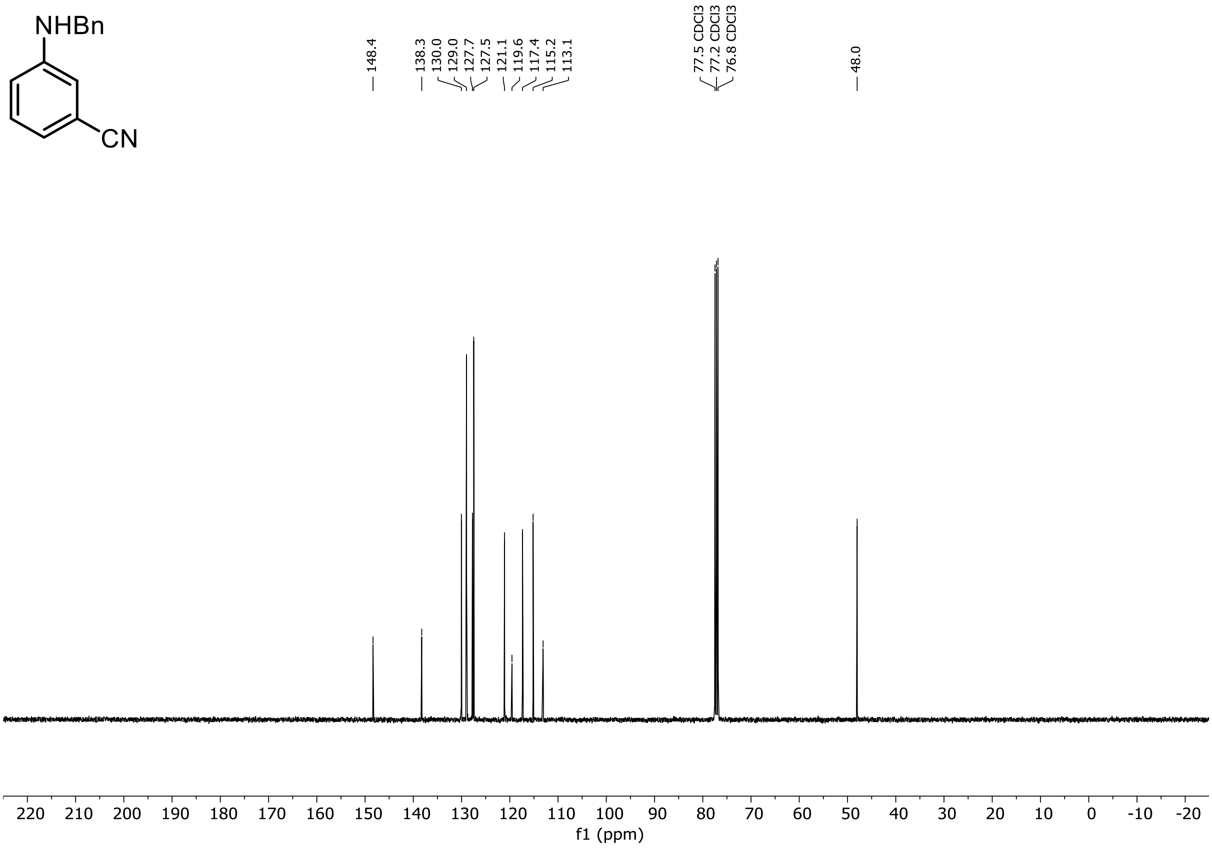
NHBn
CN
148.4
138.3
130.0
129.0
127.7
127.5
121.1
119.6
117.4
115.2
113.1
77.5 CDCl3
77.2 CDCl3
76.8 CDCl3
48.0
220 210 200 190 180 170 160 150 140 130 120 110 100 90 80 70 60 50 40 30 20 10 0 -10 -20
f1 (ppm)

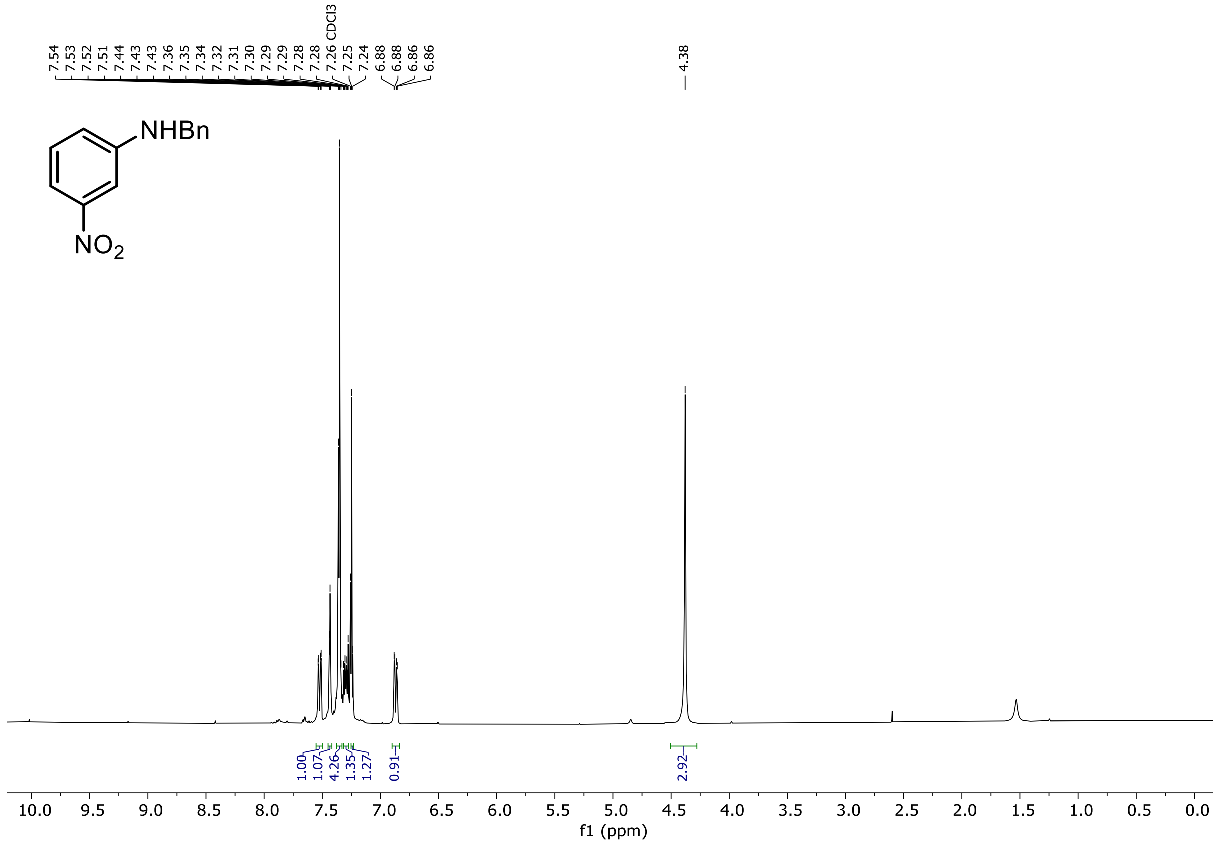
7.54
7.53
7.52
7.51
7.44
7.43
7.43
7.36
7.35
7.34
7.32
7.31
7.30
7.29
7.29
7.28
7.28
7.26 CDCl3
7.25
7.24
6.88
6.88
6.86
6.86
4.38
NHBn
NO2
1.00
1.07
4.26
1.35
1.27
0.91
2.92
10.0 9.5 9.0 8.5 8.0 7.5 7.0 6.5 6.0 5.5 5.0 4.5 4.0 3.5 3.0 2.5 2.0 1.5 1.0 0.5 0.0
f1 (ppm)

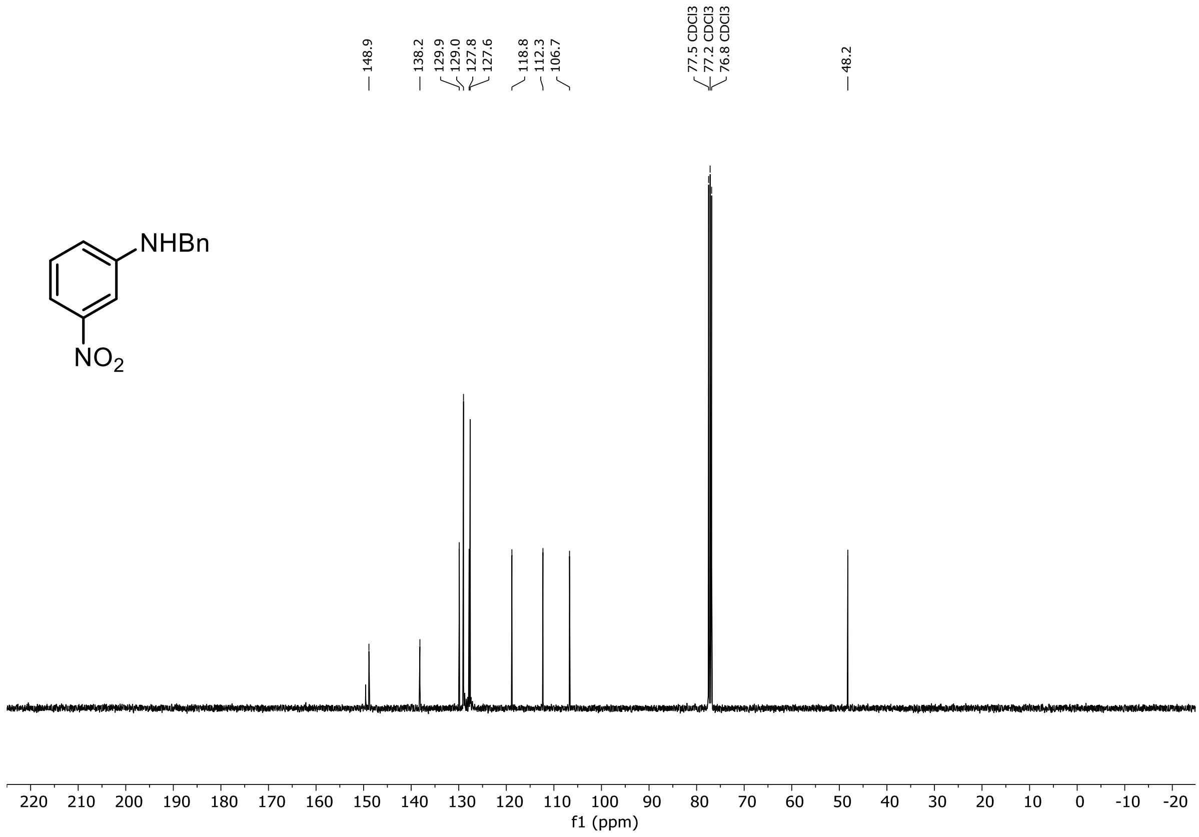
148.9
138.2
129.9
129.0
127.8
127.6
118.8
112.3
106.7
77.5 CDCl3
77.2 CDCl3
76.8 CDCl3
48.2
NHBn
NO2
220 210 200 190 180 170 160 150 140 130 120 110 100 90 80 70 60 50 40 30 20 10 0 -10 -20
f1 (ppm)

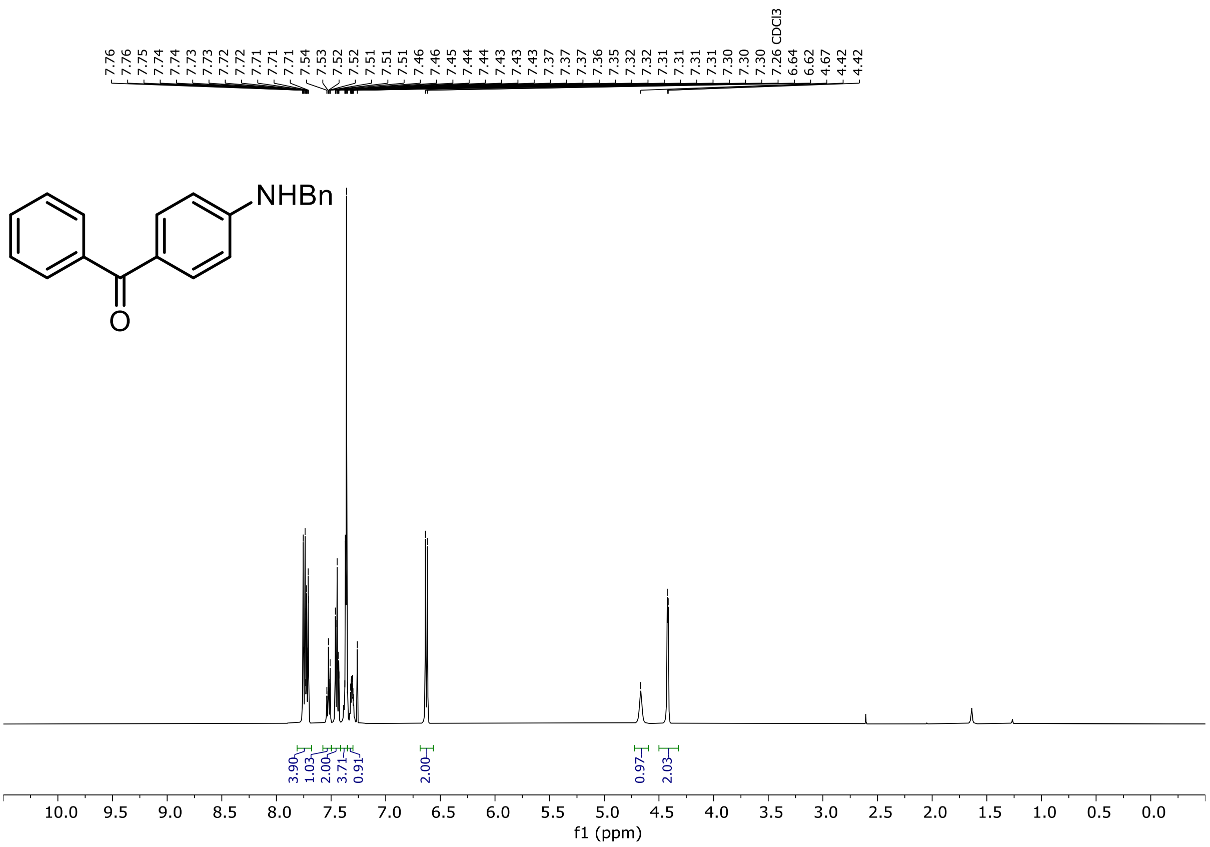

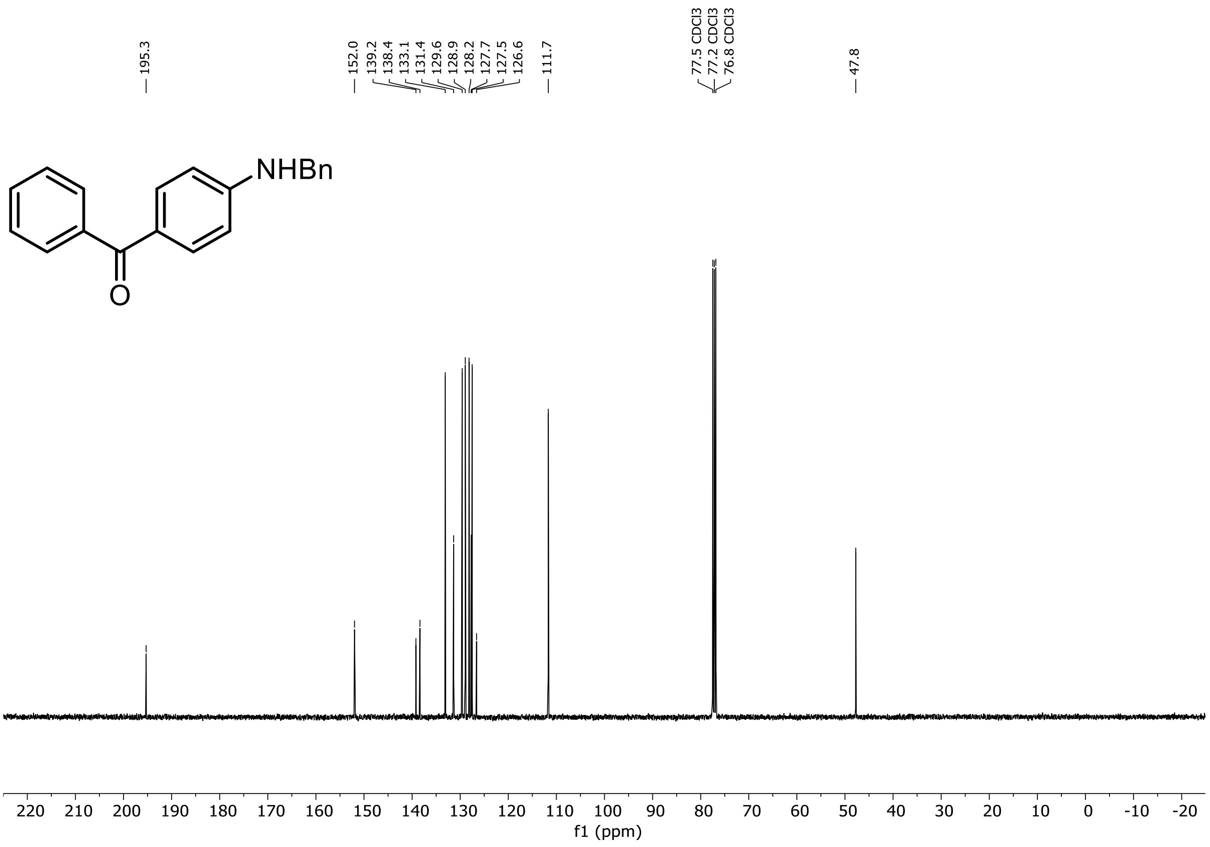

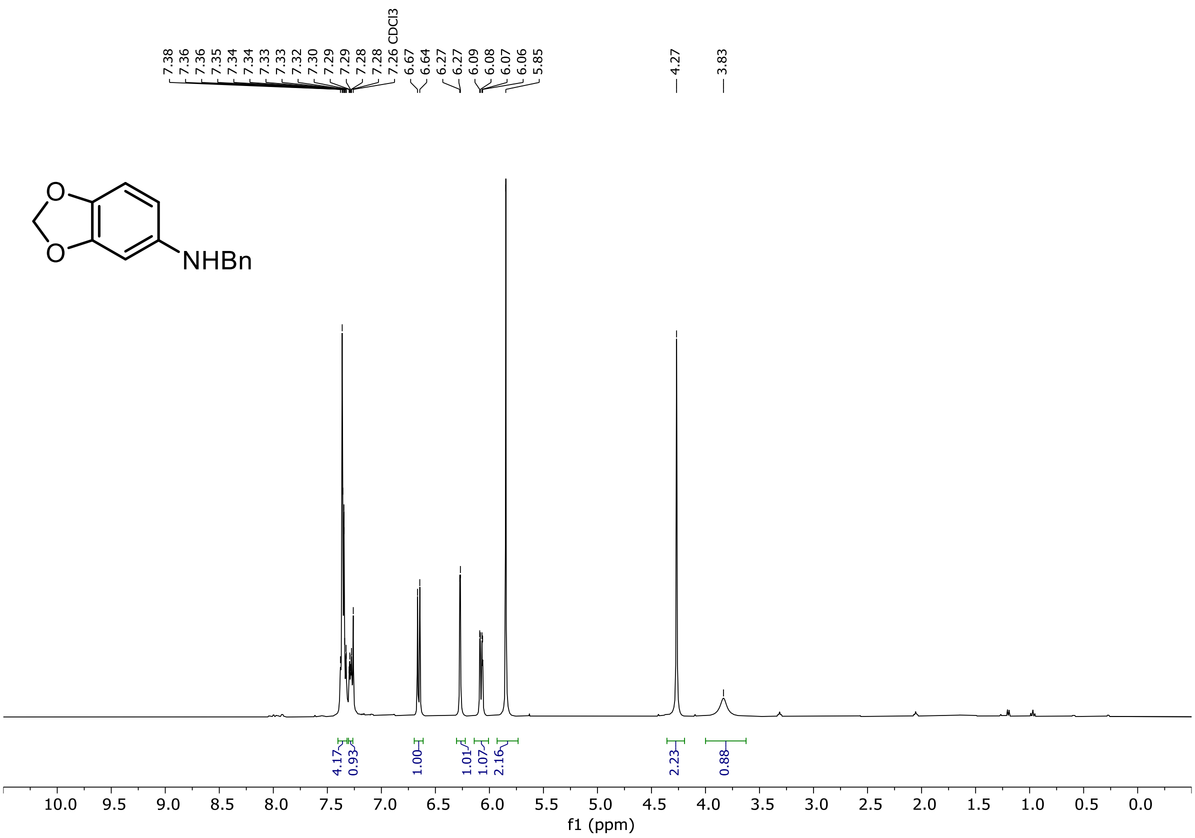
7.38
7.36
7.36
7.35
7.34
7.34
7.33
7.33
7.32
7.30
7.29
7.29
7.28
7.28
7.26 CDCl3
6.67
6.64
6.27
6.27
6.09
6.08
6.07
6.06
5.85
4.27
3.83
O
O
NHBn
4.17
0.93
1.00
1.01
1.07
2.16
2.23
0.88
10.0 9.5 9.0 8.5 8.0 7.5 7.0 6.5 6.0 5.5 5.0 4.5 4.0 3.5 3.0 2.5 2.0 1.5 1.0 0.5 0.0
f1 (ppm)

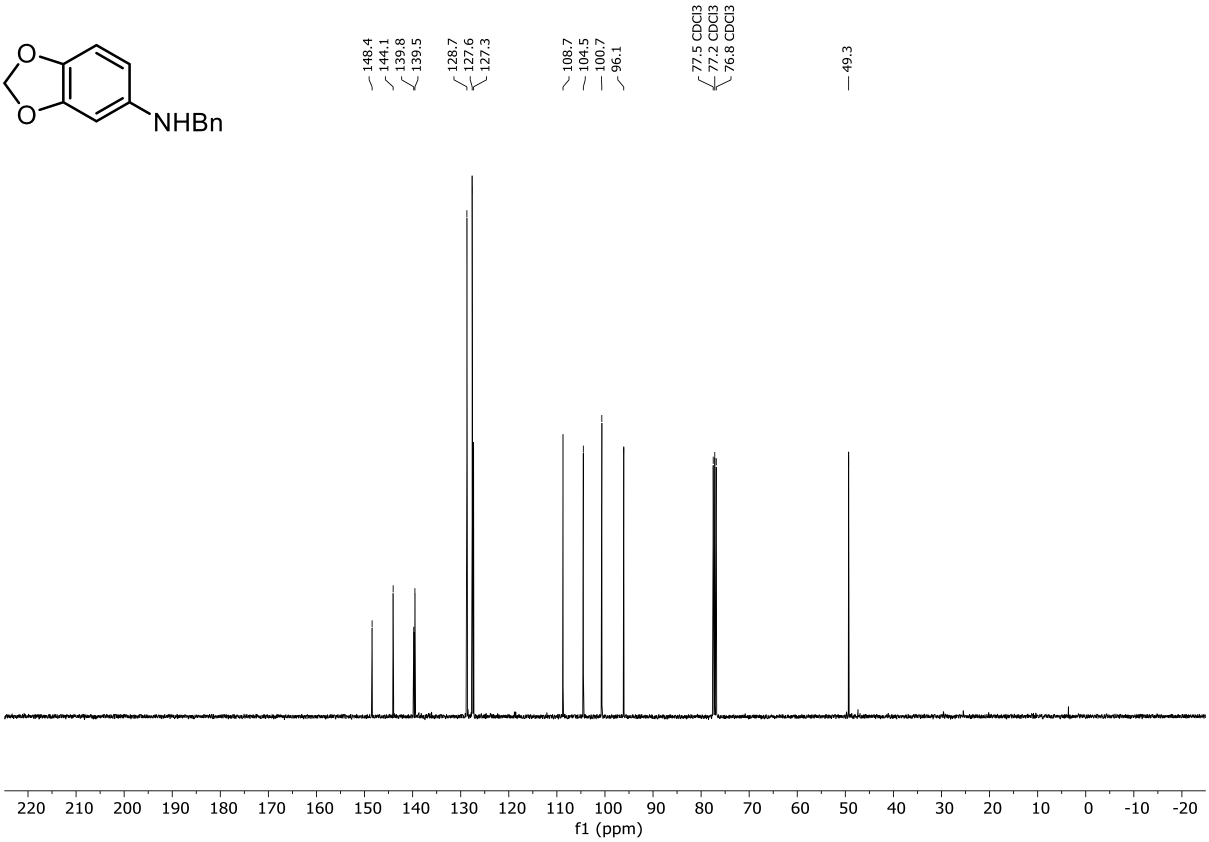
O
O
NHBn
148.4
144.1
139.8
139.5
128.7
127.6
127.3
108.7
104.5
100.7
96.1
77.5 CDCl3
77.2 CDCl3
76.8 CDCl3
49.3
220 210 200 190 180 170 160 150 140 130 120 110 100 90 80 70 60 50 40 30 20 10 0 -10 -20
f1 (ppm)

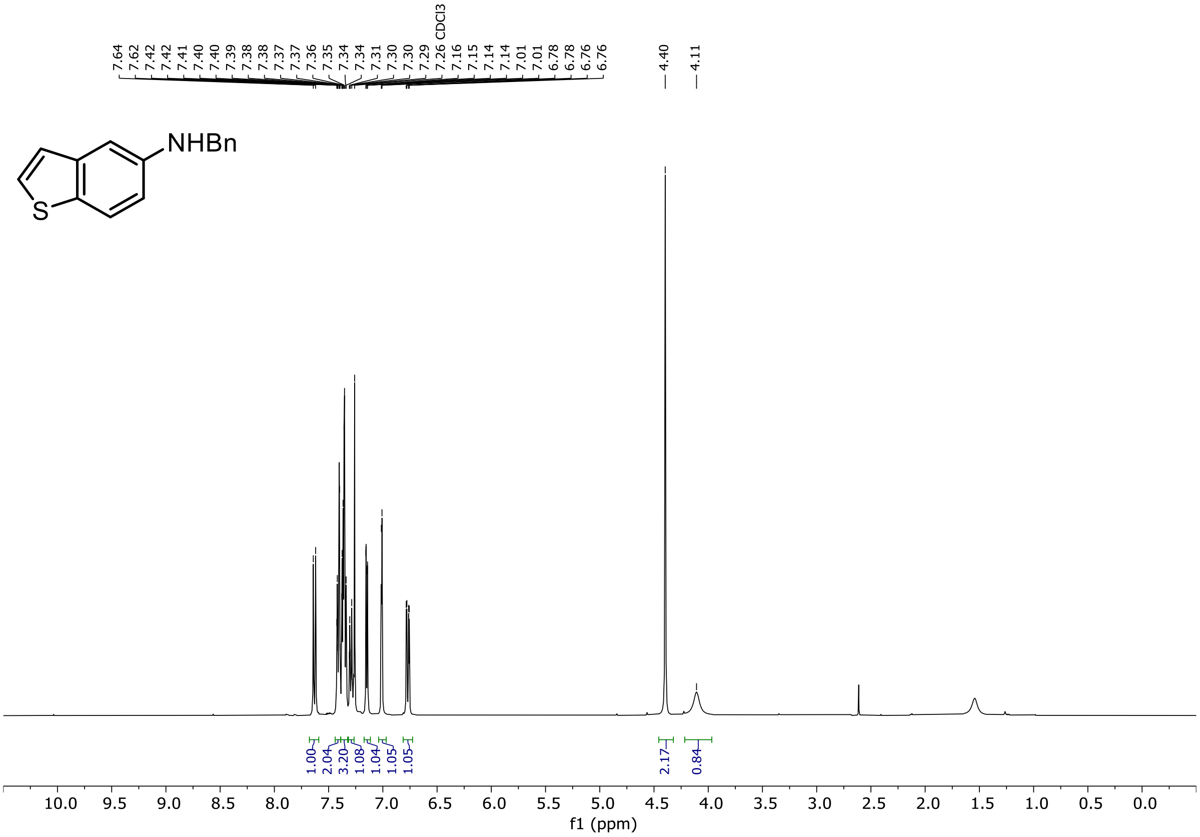
NHBn
S
7.64
7.62
7.42
7.42
7.41
7.40
7.39
7.38
7.38
7.37
7.37
7.36
7.35
7.34
7.34
7.31
7.30
7.30
7.29
7.26 CDCl3
7.16
7.15
7.14
7.14
7.01
7.01
6.78
6.78
6.76
6.76
4.40
4.11
1.00
2.04
3.20
1.08
1.04
1.05
1.05
2.17
0.84
10.0 9.5 9.0 8.5 8.0 7.5 7.0 6.5 6.0 5.5 5.0 4.5 4.0 3.5 3.0 2.5 2.0 1.5 1.0 0.5 0.0
f1 (ppm)

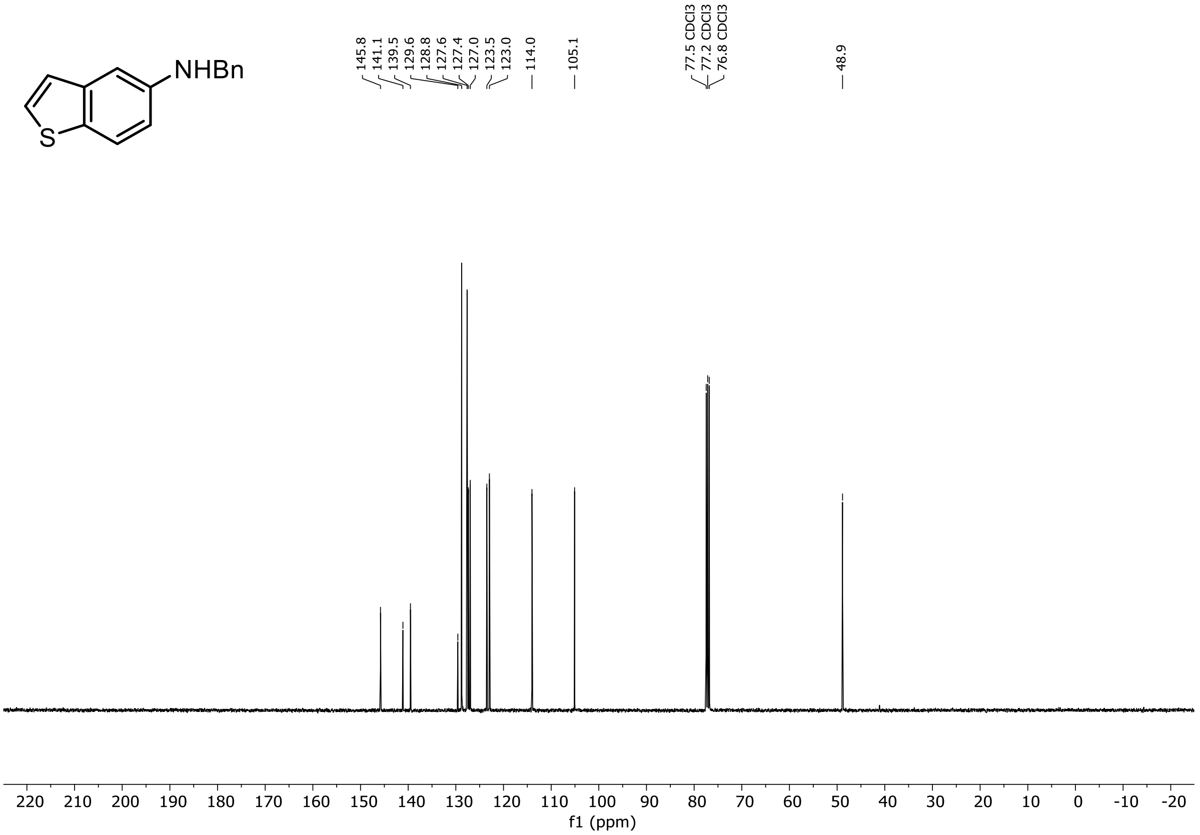
NHBn
S
145.8
141.1
139.5
129.6
128.8
127.6
127.4
127.0
123.5
123.0
114.0
105.1
77.5 CDCl3
77.2 CDCl3
76.8 CDCl3
48.9
220 210 200 190 180 170 160 150 140 130 120 110 100 90 80 70 60 50 40 30 20 10 0 -10 -20
f1 (ppm)

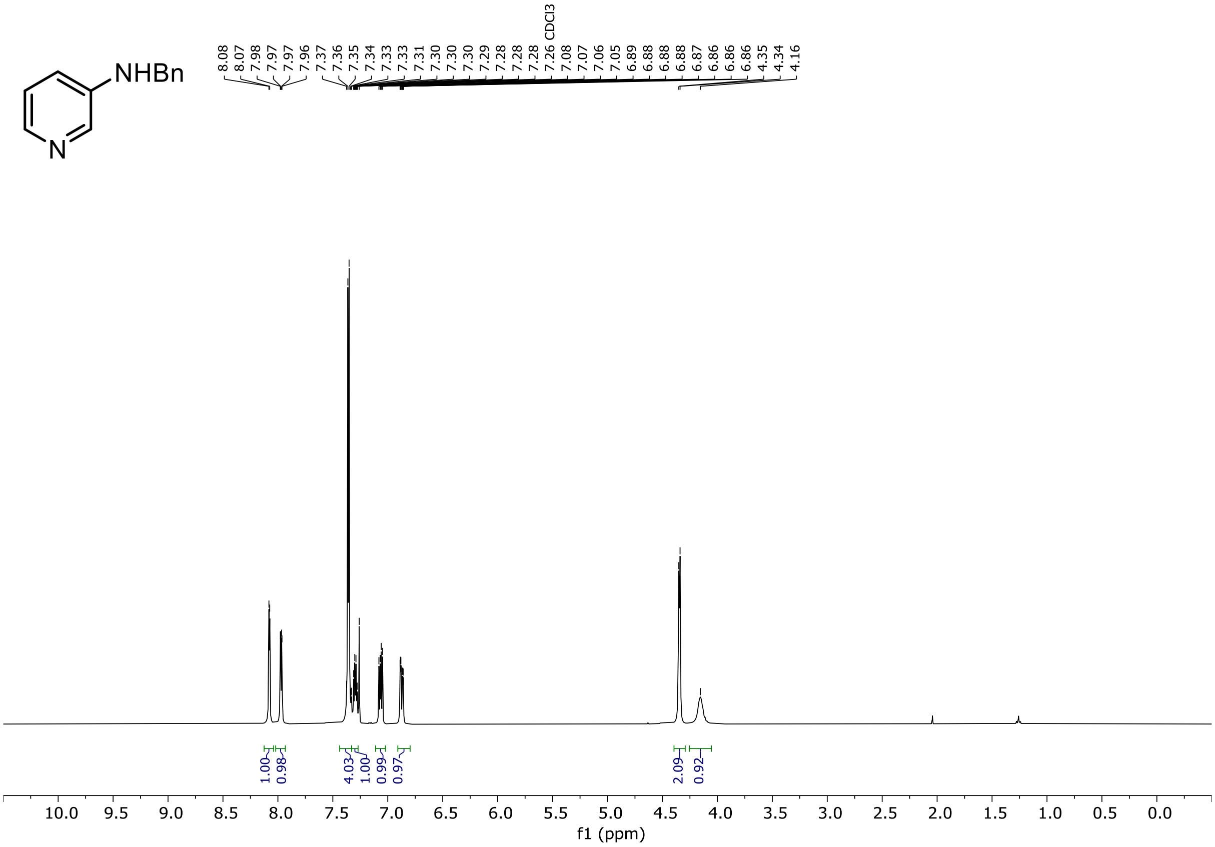
NHBn
N
8.08
8.07
7.98
7.97
7.97
7.96
7.37
7.36
7.35
7.34
7.33
7.33
7.31
7.30
7.30
7.30
7.29
7.28
7.28
7.28
7.26 CDCl3
7.08
7.07
7.06
7.05
6.89
6.88
6.88
6.88
6.87
6.86
6.86
6.86
4.35
4.34
4.16
1.00
0.98
4.03
1.00
0.99
0.97
2.09
0.92
10.0 9.5 9.0 8.5 8.0 7.5 7.0 6.5 6.0 5.5 5.0 4.5 4.0 3.5 3.0 2.5 2.0 1.5 1.0 0.5 0.0
f1 (ppm)

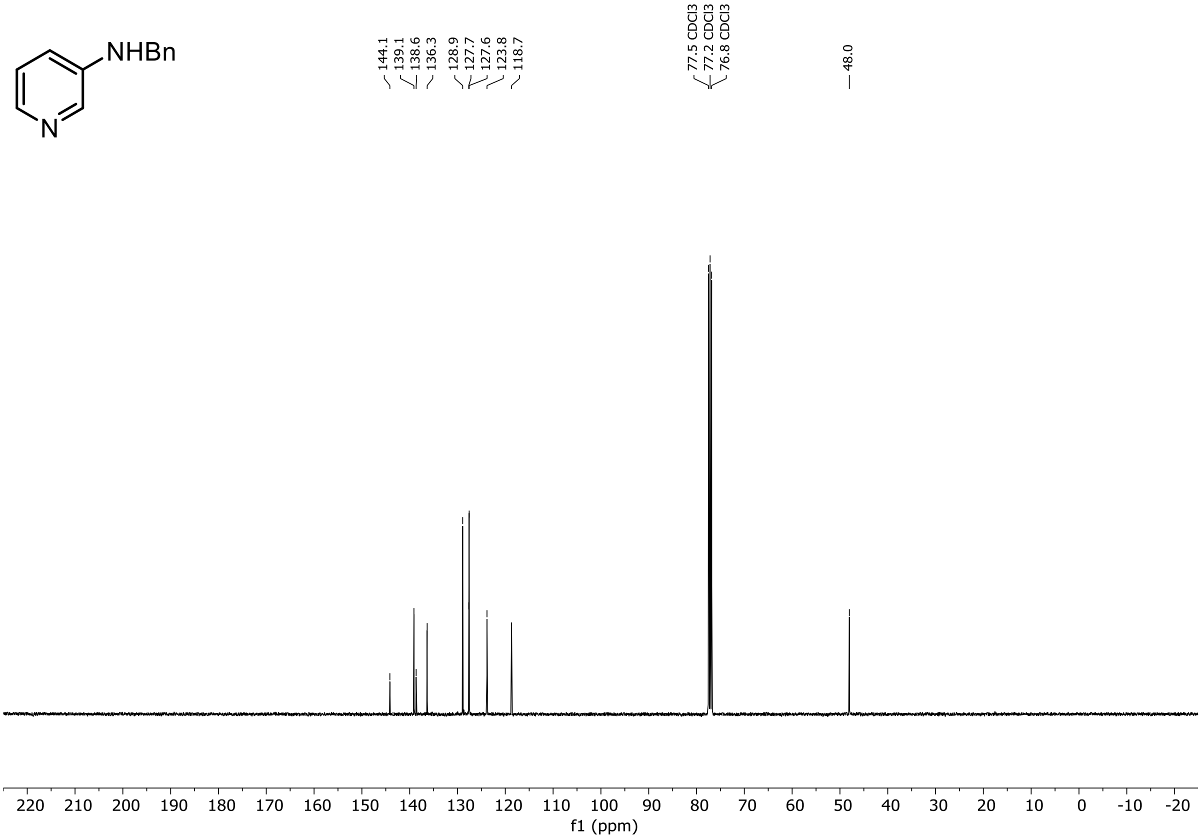
NHBn
N
144.1
139.1
138.6
136.3
128.9
127.7
127.6
123.8
118.7
77.5 CDCl3
77.2 CDCl3
76.8 CDCl3
48.0
220 210 200 190 180 170 160 150 140 130 120 110 100 90 80 70 60 50 40 30 20 10 0 -10 -20
f1 (ppm)

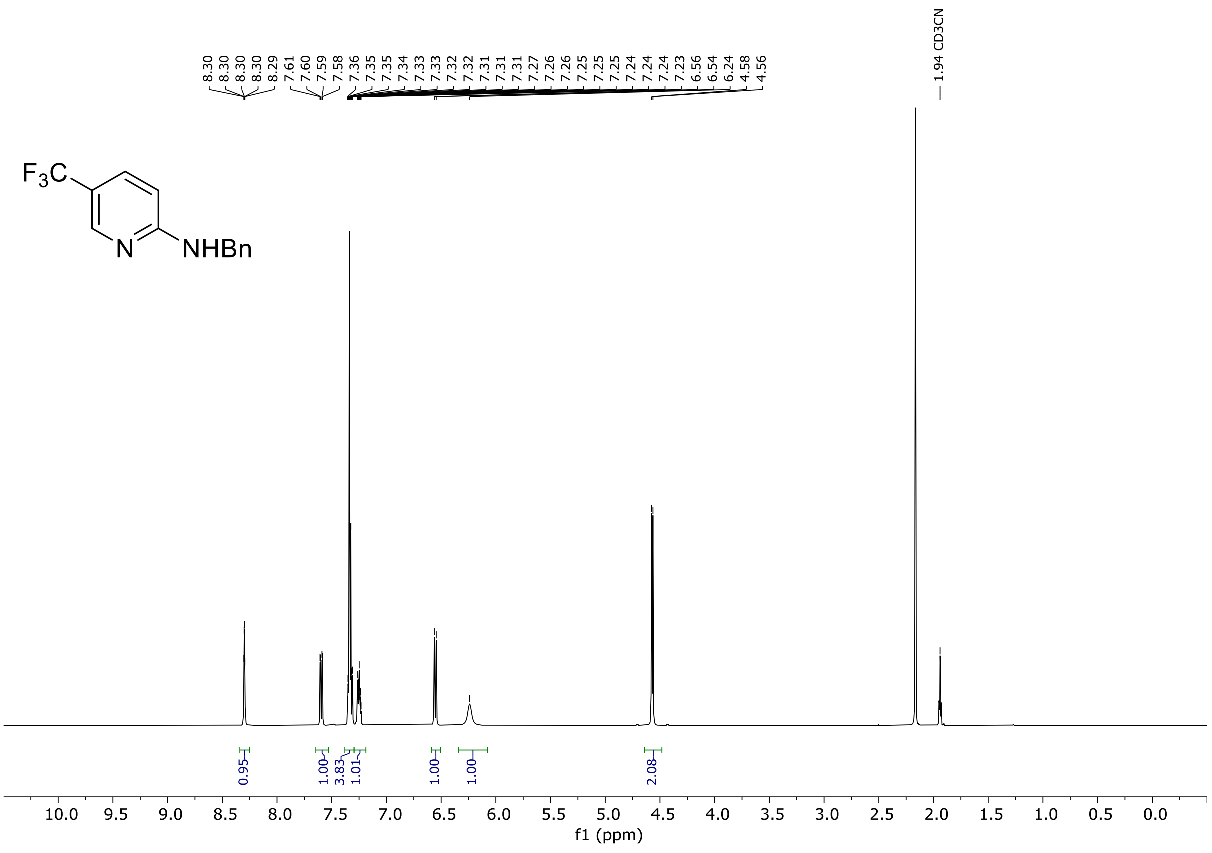
8.30
8.30
8.30
8.30
8.29
7.61
7.60
7.59
7.58
7.36
7.35
7.35
7.34
7.33
7.33
7.32
7.32
7.31
7.31
7.31
7.27
7.26
7.26
7.25
7.25
7.25
7.24
7.24
7.24
7.23
6.56
6.54
6.24
4.58
4.56
1.94 CD3CN
F3C
N
NHBn
0.95
1.00
3.83
1.01
1.00
1.00
2.08
10.0 9.5 9.0 8.5 8.0 7.5 7.0 6.5 6.0 5.5 5.0 4.5 4.0 3.5 3.0 2.5 2.0 1.5 1.0 0.5 0.0
f1 (ppm)

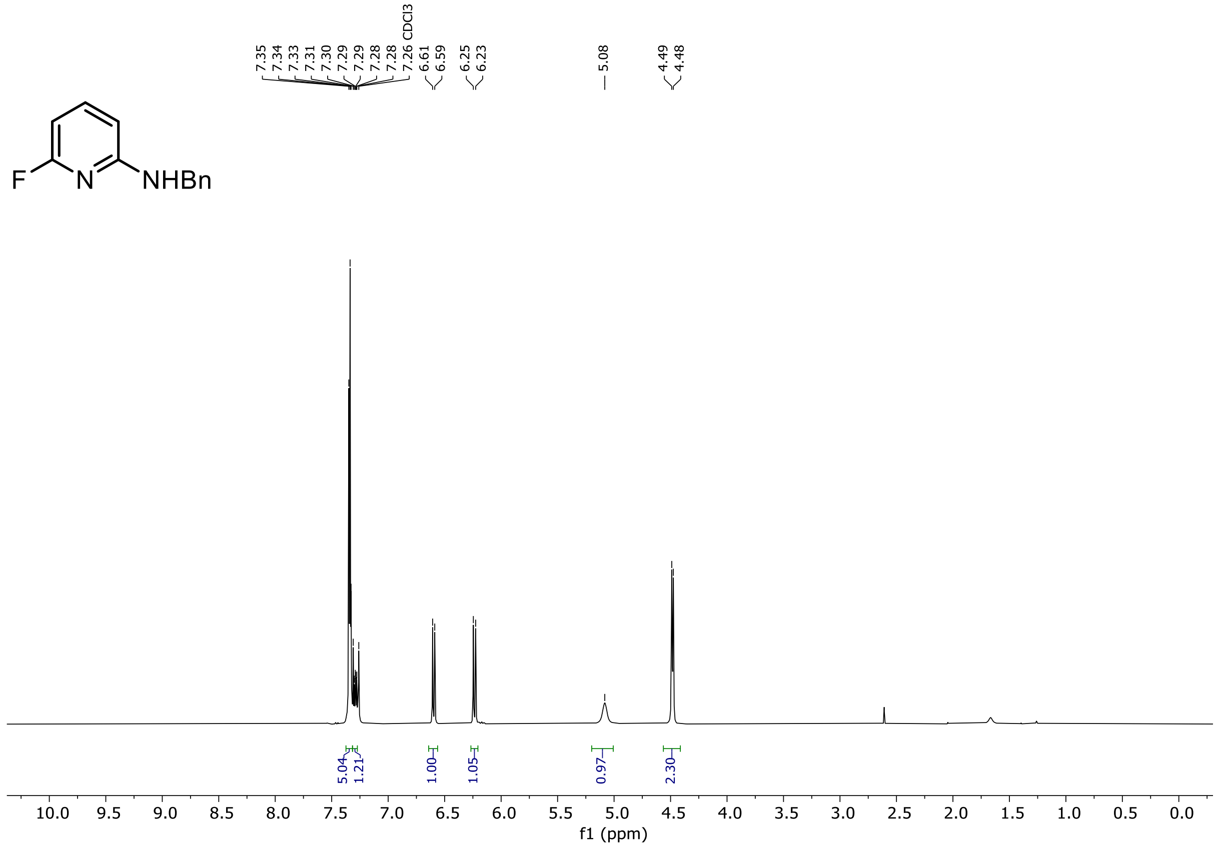
F
N
NHBn
7.35
7.34
7.33
7.31
7.30
7.29
7.29
7.28
7.28
7.26 CDCl3
6.61
6.59
6.25
6.23
5.08
4.49
4.48
5.04
1.21
1.00
1.05
0.97
2.30
10.0 9.5 9.0 8.5 8.0 7.5 7.0 6.5 6.0 5.5 5.0 4.5 4.0 3.5 3.0 2.5 2.0 1.5 1.0 0.5 0.0
f1 (ppm)

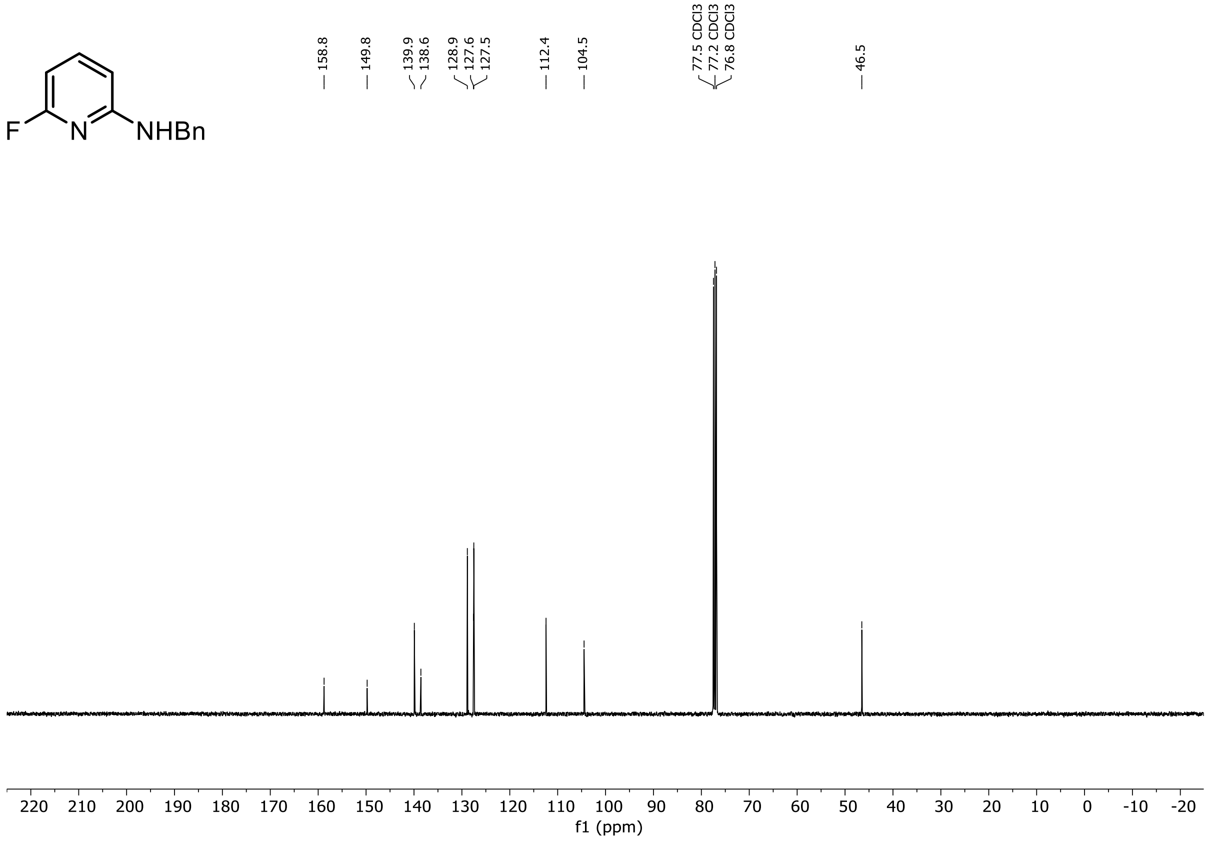
F
N
NHBn
158.8
149.8
139.9
138.6
128.9
127.6
127.5
112.4
104.5
77.5 CDCl3
77.2 CDCl3
76.8 CDCl3
46.5
220 210 200 190 180 170 160 150 140 130 120 110 100 90 80 70 60 50 40 30 20 10 0 -10 -20
f1 (ppm)

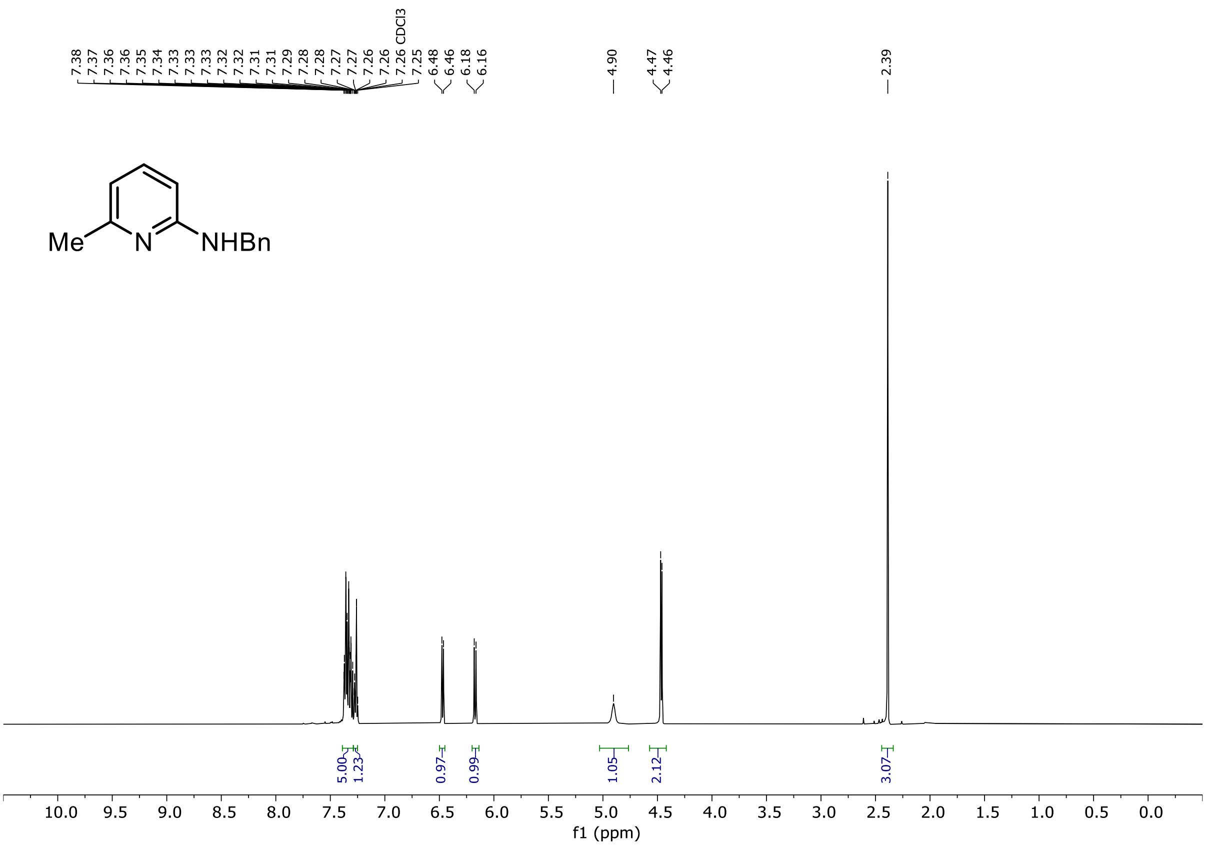
7.38
7.37
7.36
7.36
7.35
7.34
7.33
7.33
7.33
7.32
7.32
7.31
7.31
7.29
7.28
7.28
7.27
7.27
7.26
7.26
7.26 CDCl3
7.25
6.48
6.46
6.18
6.16
4.90
4.47
4.46
2.39
Me
N
NHBn
5.00
1.23
0.97
0.99
1.05
2.12
3.07
10.0 9.5 9.0 8.5 8.0 7.5 7.0 6.5 6.0 5.5 5.0 4.5 4.0 3.5 3.0 2.5 2.0 1.5 1.0 0.5 0.0
f1 (ppm)

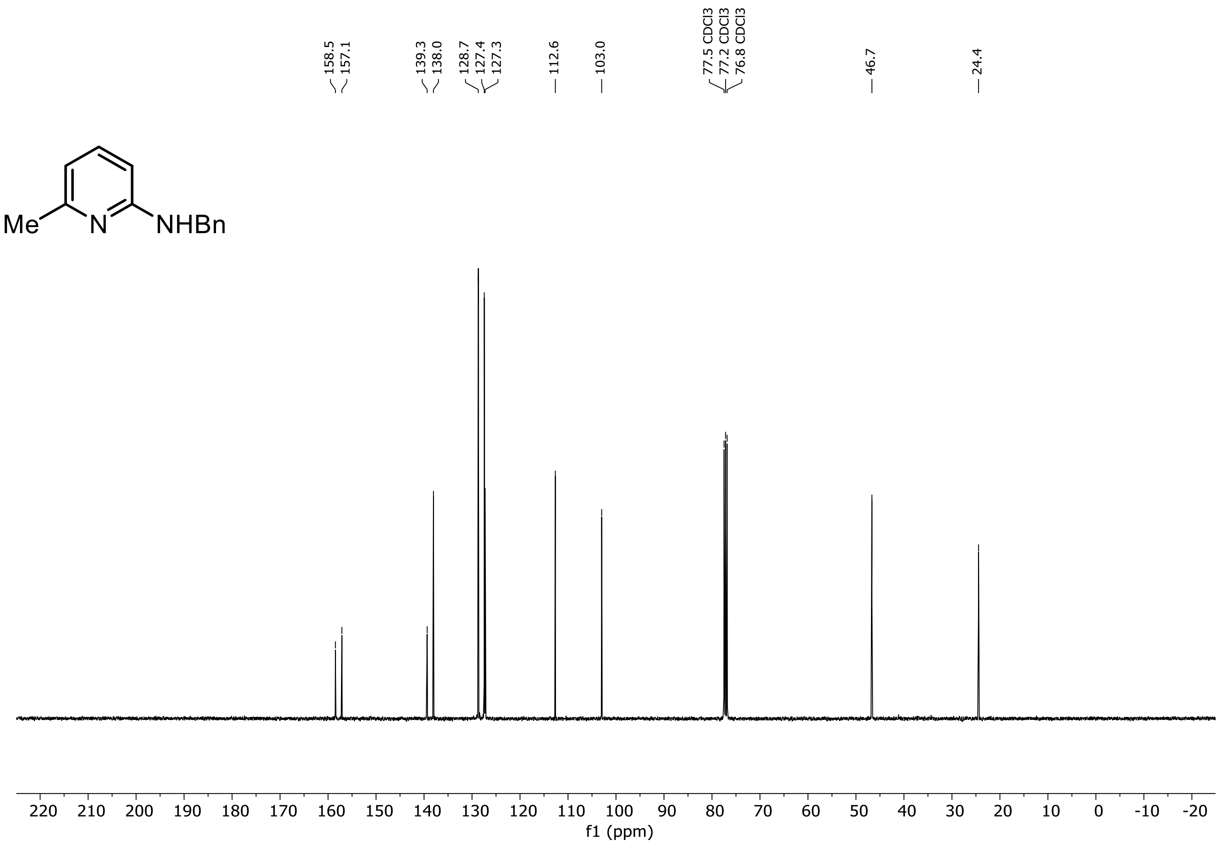
158.5
157.1
139.3
138.0
128.7
127.4
127.3
112.6
103.0
77.5 CDCl3
77.2 CDCl3
76.8 CDCl3
46.7
24.4
Me
N
NHBn
220 210 200 190 180 170 160 150 140 130 120 110 100 90 80 70 60 50 40 30 20 10 0 -10 -20
f1 (ppm)

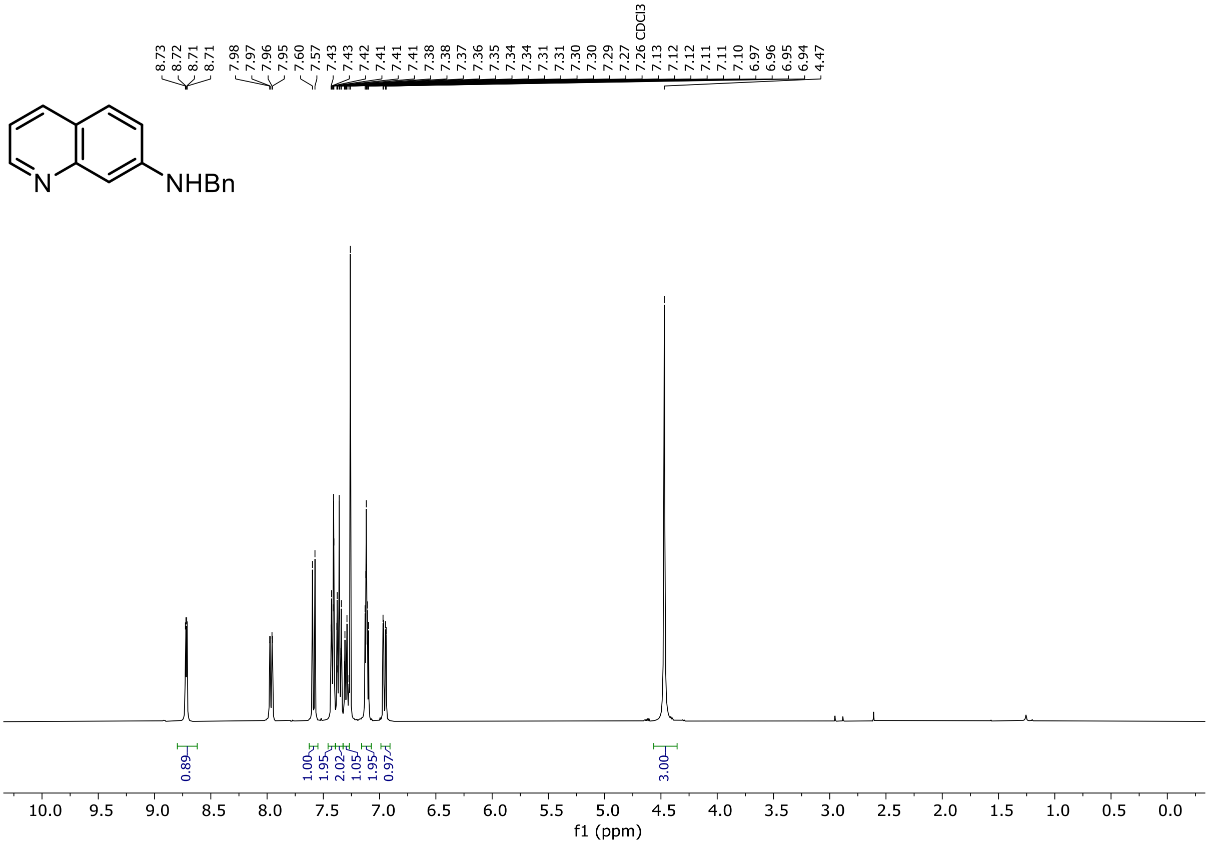
8.73
8.72
8.71
8.71
7.98
7.97
7.96
7.95
7.60
7.57
7.43
7.43
7.42
7.41
7.41
7.41
7.38
7.38
7.37
7.36
7.35
7.34
7.34
7.31
7.31
7.30
7.30
7.29
7.27
7.26 CDCl3
7.13
7.12
7.12
7.11
7.11
7.10
6.97
6.96
6.95
6.94
4.47
N
NHBn
0.89
1.00
1.95
2.02
1.05
1.95
0.97
3.00
10.0 9.5 9.0 8.5 8.0 7.5 7.0 6.5 6.0 5.5 5.0 4.5 4.0 3.5 3.0 2.5 2.0 1.5 1.0 0.5 0.0
f1 (ppm)

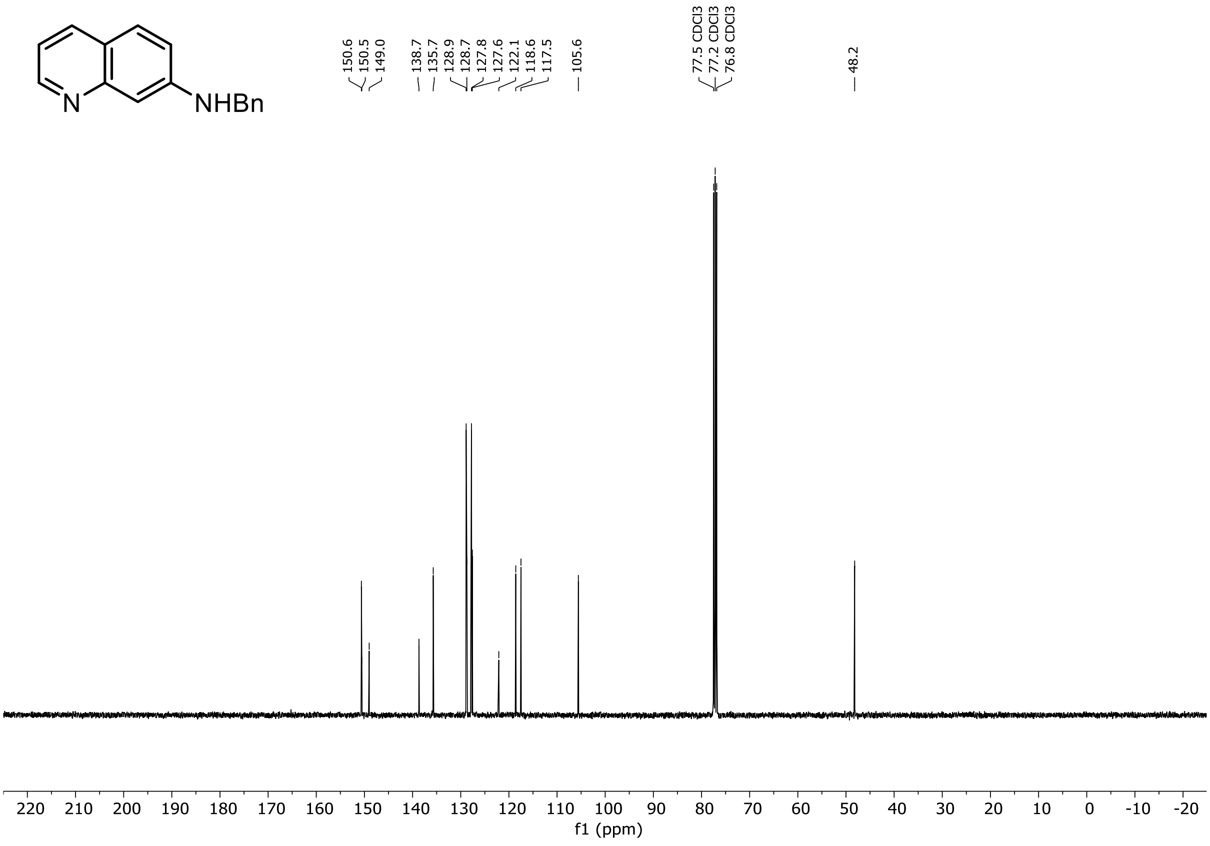
N
NHBn
150.6
150.5
149.0
138.7
135.7
128.9
128.7
127.8
127.6
122.1
118.6
117.5
105.6
77.5 CDCl3
77.2 CDCl3
76.8 CDCl3
48.2
220 210 200 190 180 170 160 150 140 130 120 110 100 90 80 70 60 50 40 30 20 10 0 -10 -20
f1 (ppm)

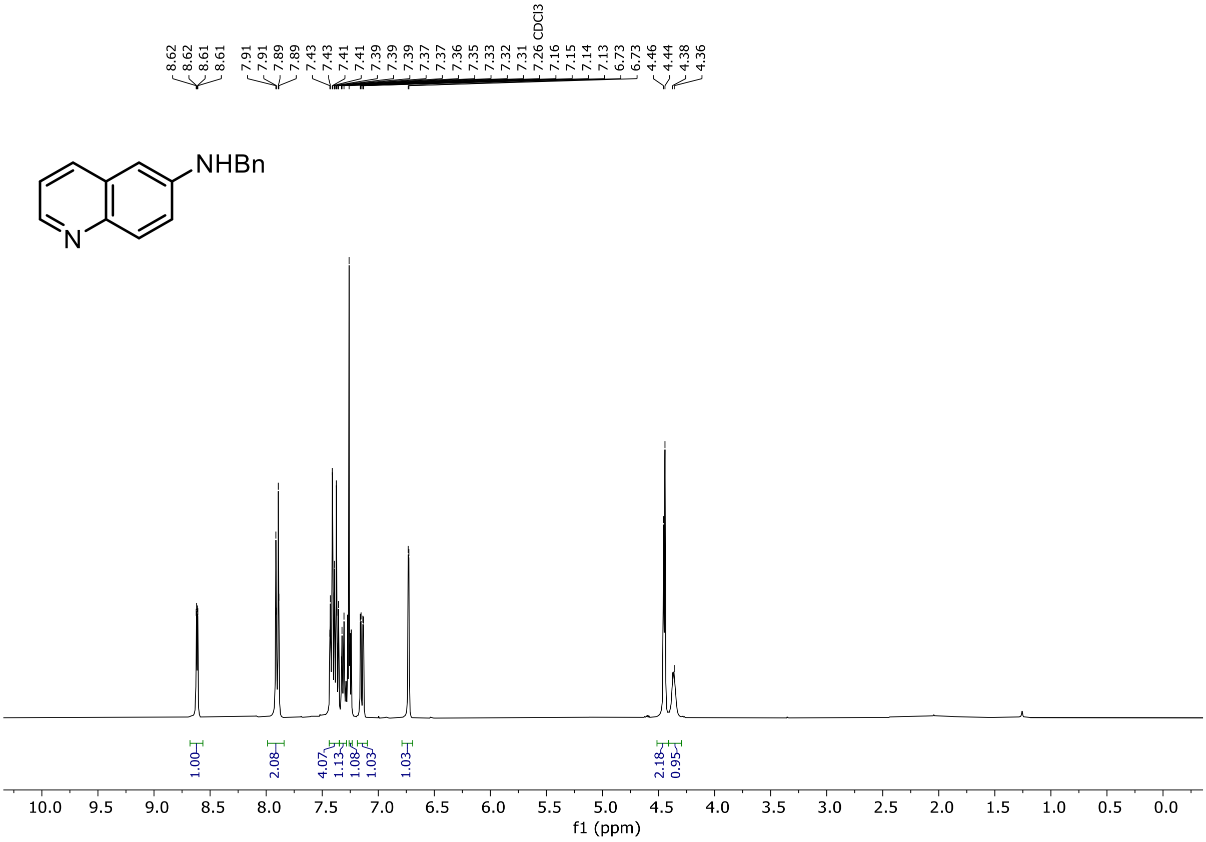
NHBn
N
8.62
8.62
8.61
8.61
7.91
7.91
7.89
7.89
7.43
7.43
7.41
7.41
7.39
7.39
7.39
7.37
7.37
7.36
7.35
7.33
7.32
7.31
7.26 CDCl3
7.16
7.15
7.14
7.13
6.73
6.73
4.46
4.44
4.38
4.36
1.00
2.08
4.07
1.13
1.08
1.03
1.03
2.18
0.95
10.0 9.5 9.0 8.5 8.0 7.5 7.0 6.5 6.0 5.5 5.0 4.5 4.0 3.5 3.0 2.5 2.0 1.5 1.0 0.5 0.0
f1 (ppm)

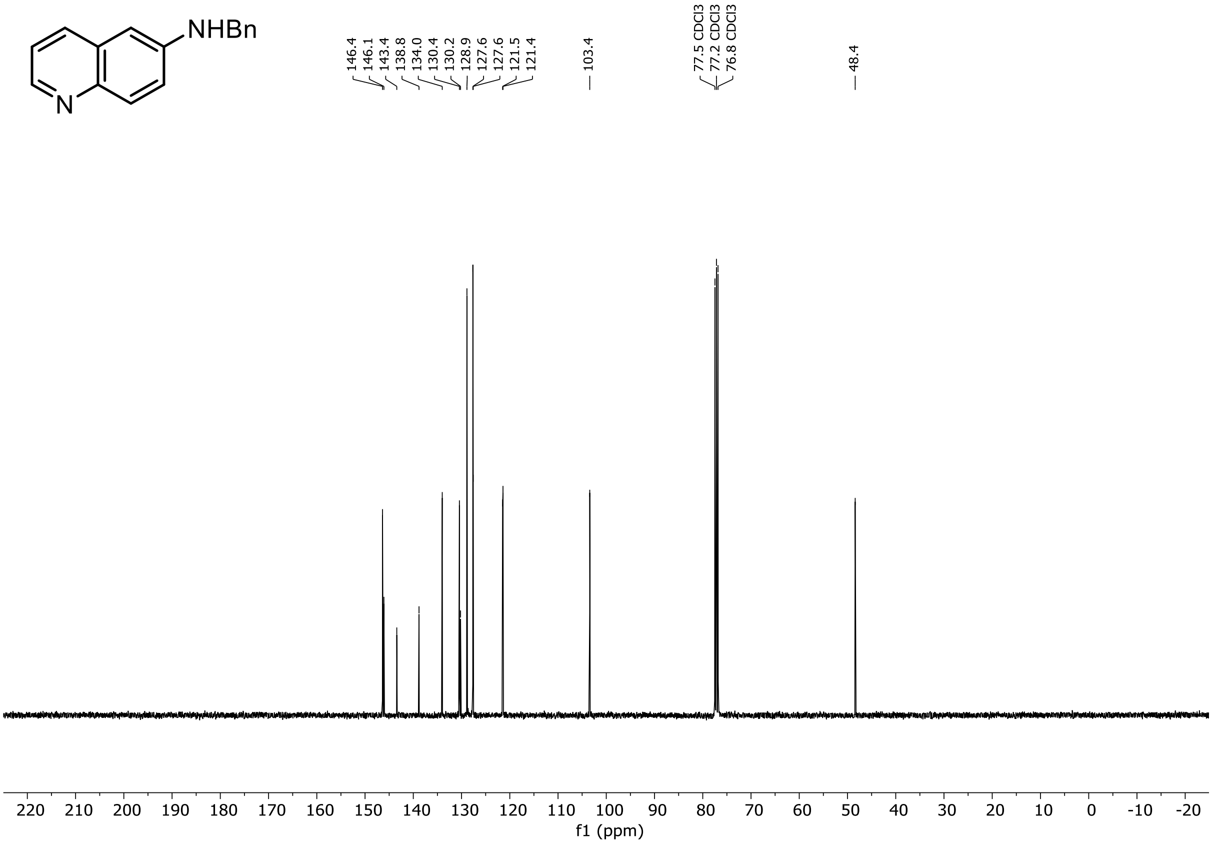
NHBn
N
146.4
146.1
143.4
138.8
134.0
130.4
130.2
128.9
127.6
127.6
121.5
121.4
103.4
77.5 CDCl3
77.2 CDCl3
76.8 CDCl3
48.4
220 210 200 190 180 170 160 150 140 130 120 110 100 90 80 70 60 50 40 30 20 10 0 -10 -20
f1 (ppm)

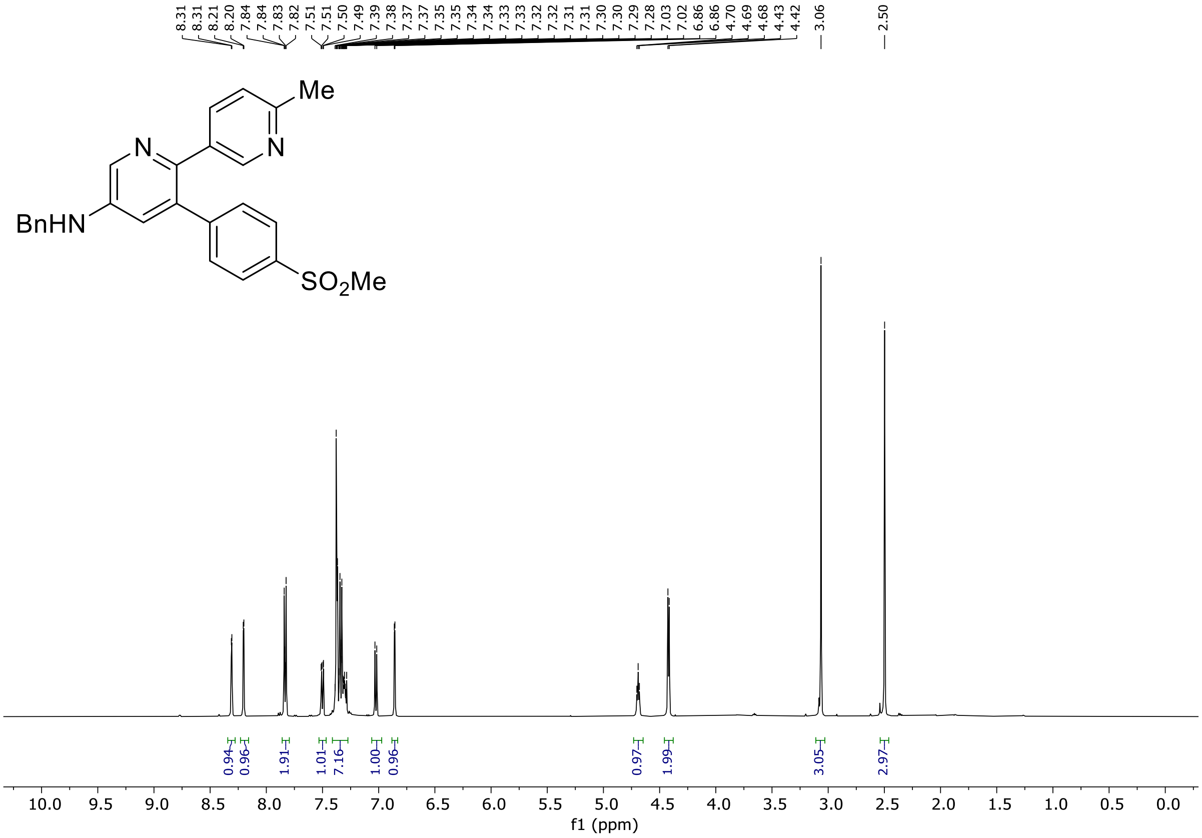
8.31
8.31
8.21
8.20
7.84
7.84
7.83
7.82
7.51
7.51
7.50
7.49
7.39
7.38
7.37
7.37
7.35
7.35
7.34
7.34
7.33
7.33
7.32
7.32
7.31
7.31
7.30
7.30
7.29
7.28
7.03
7.02
6.86
6.86
4.70
4.69
4.68
4.43
4.42
3.06
2.50
Me
N
N
BnHN
SO2Me
0.94
0.96
1.91
1.01
7.16
1.00
0.96
0.97
1.99
3.05
2.97
10.0 9.5 9.0 8.5 8.0 7.5 7.0 6.5 6.0 5.5 5.0 4.5 4.0 3.5 3.0 2.5 2.0 1.5 1.0 0.5 0.0
f1 (ppm)

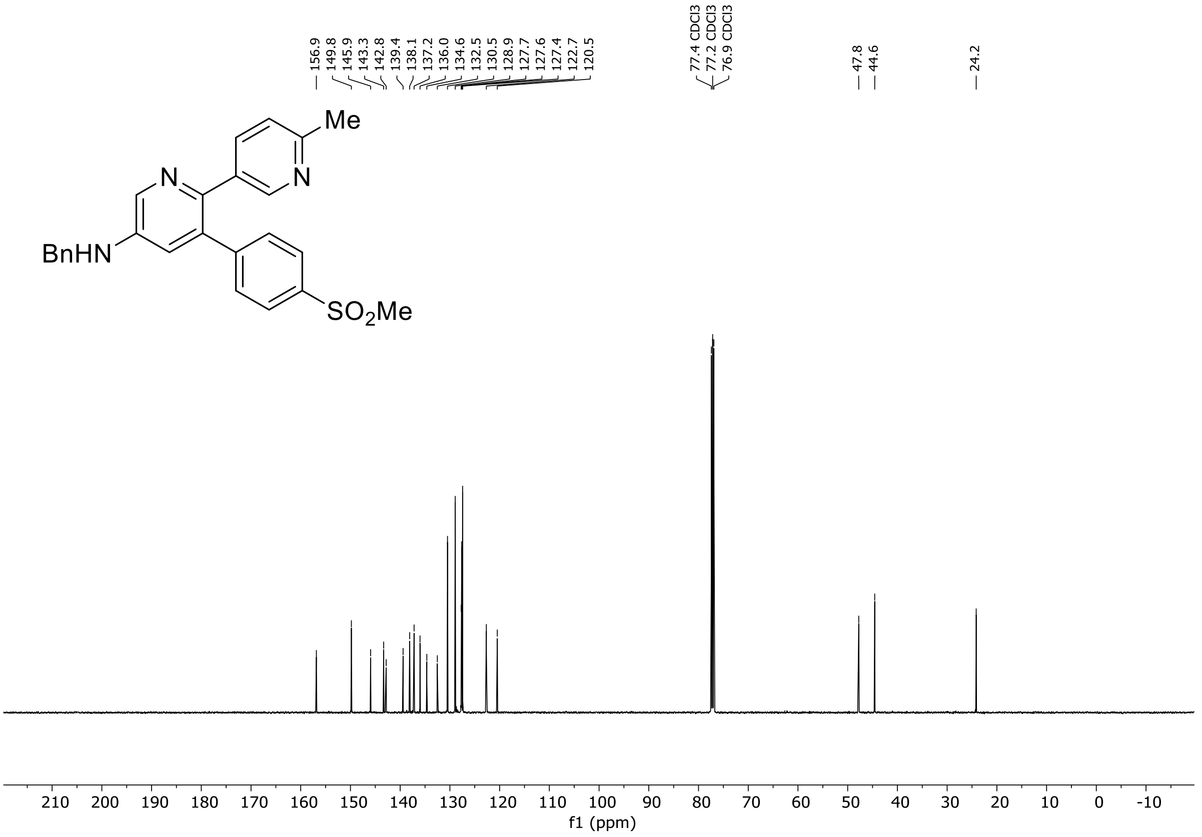
156.9
149.8
145.9
143.3
142.8
139.4
138.1
137.2
136.0
134.6
132.5
130.5
128.9
127.7
127.6
127.4
122.7
120.5
77.4 CDCl3
77.2 CDCl3
76.9 CDCl3
47.8
44.6
24.2
Me
N
N
BnHN
SO2Me
210 200 190 180 170 160 150 140 130 120 110 100 90 80 70 60 50 40 30 20 10 0 -10
f1 (ppm)

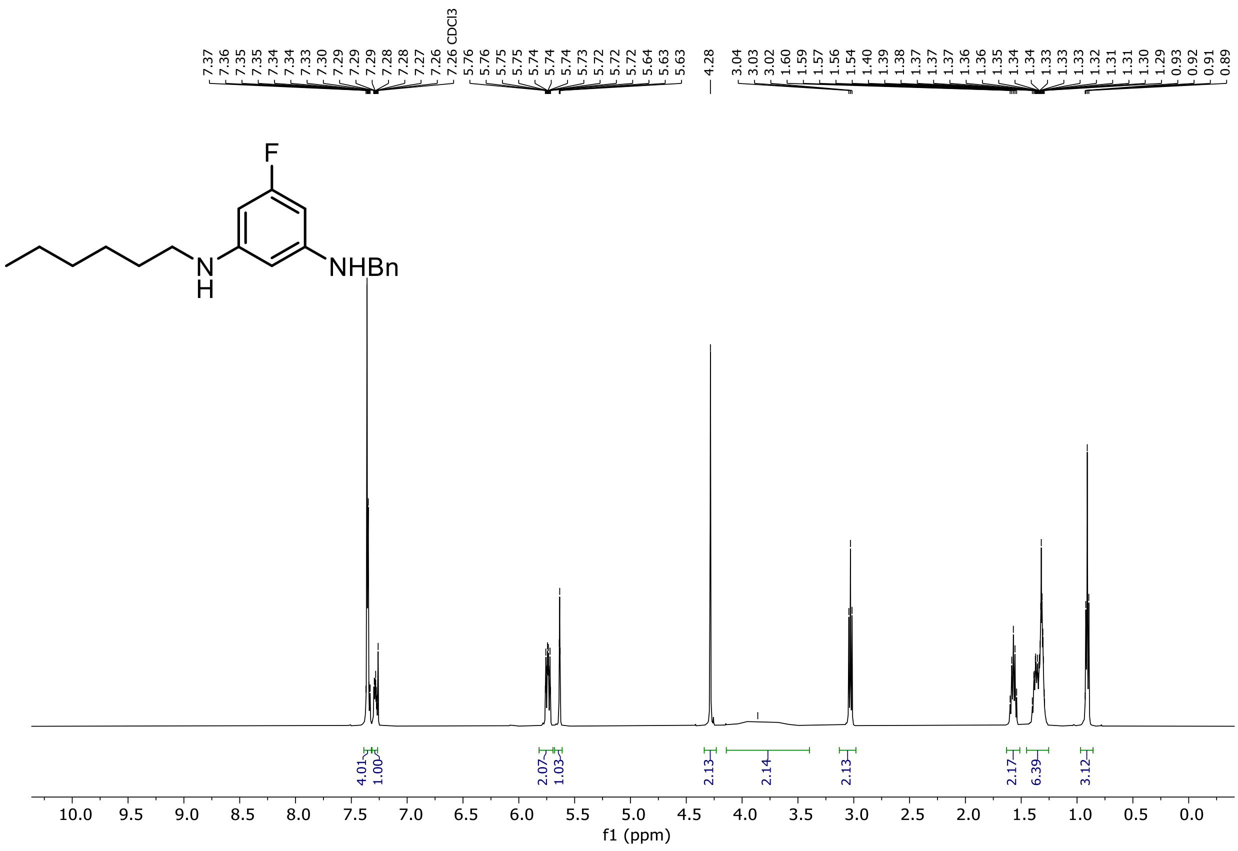
7.37 7.36 7.35 7.35 7.34 7.34 7.33 7.30 7.29 7.29 7.29 7.28 7.28 7.27 7.26 7.26 CDCl3 5.76 5.76 5.75 5.75 5.74 5.74 5.74 5.73 5.72 5.72 5.72 5.64 5.63 5.63
4.28
3.04 3.03 3.02 1.60 1.59 1.57 1.56 1.54 1.40 1.39 1.38 1.37 1.37 1.37 1.36 1.36 1.35 1.34 1.34 1.33 1.33 1.33 1.32 1.31 1.31 1.30 1.29 0.93 0.92 0.91 0.89
F
N
H
NHBn
4.01
1.00
2.07
1.03
2.13
2.14
2.13
2.17
6.39
3.12
10.0 9.5 9.0 8.5 8.0 7.5 7.0 6.5 6.0 5.5 5.0 4.5 4.0 3.5 3.0 2.5 2.0 1.5 1.0 0.5 0.0
f1 (ppm)

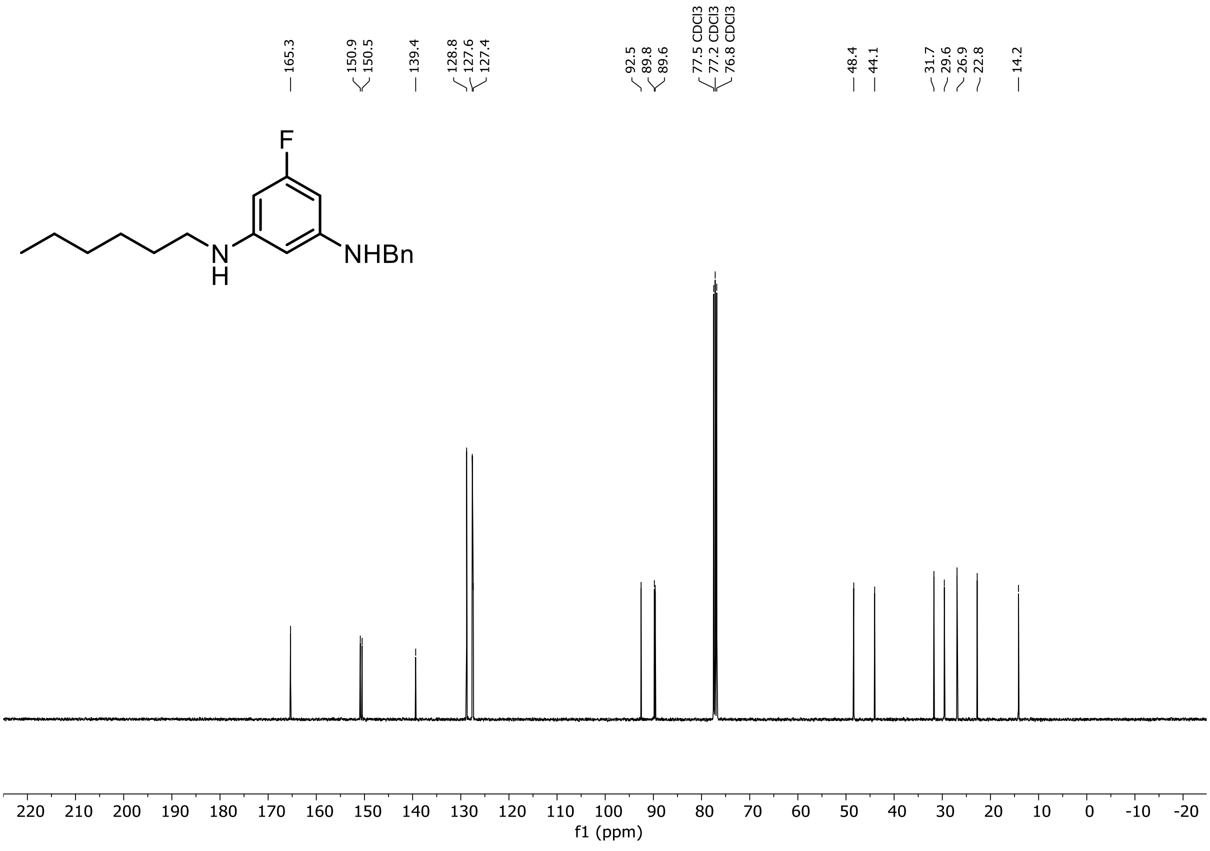
165.3
150.9 150.5
139.4
128.8 127.6 127.4
92.5 89.8 89.6
77.5 CDCl3 77.2 CDCl3 76.8 CDCl3
48.4
44.1
31.7 29.6 26.9 22.8
14.2
F
N
H
NHBn
220 210 200 190 180 170 160 150 140 130 120 110 100 90 80 70 60 50 40 30 20 10 0 -10 -20
f1 (ppm)

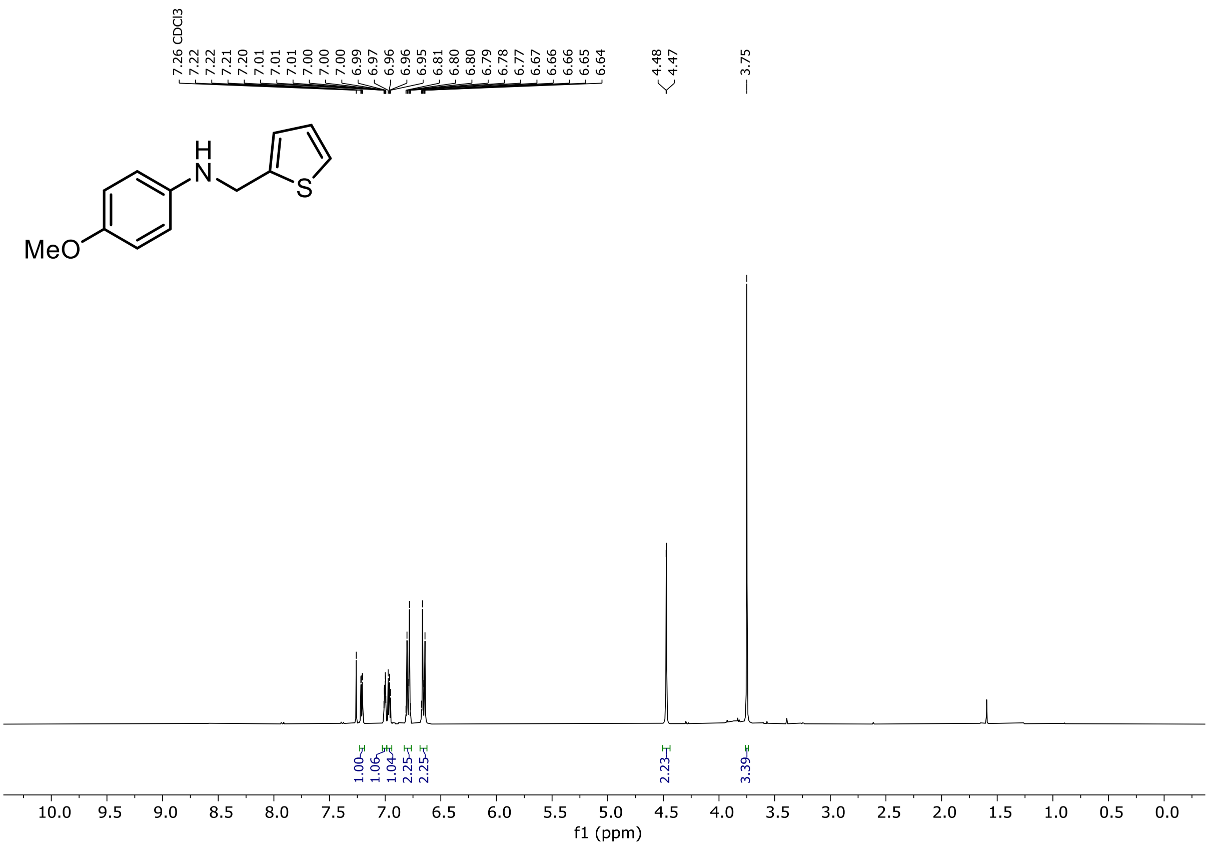
7.26 CDCl3
7.22
7.22
7.21
7.20
7.01
7.01
7.01
7.00
7.00
7.00
6.99
6.97
6.96
6.96
6.95
6.81
6.80
6.80
6.79
6.78
6.77
6.67
6.66
6.66
6.65
6.64
4.48
4.47
3.75
MeO
H
N
S
1.00
1.06
1.04
2.25
2.25
2.23
3.39
f1 (ppm)

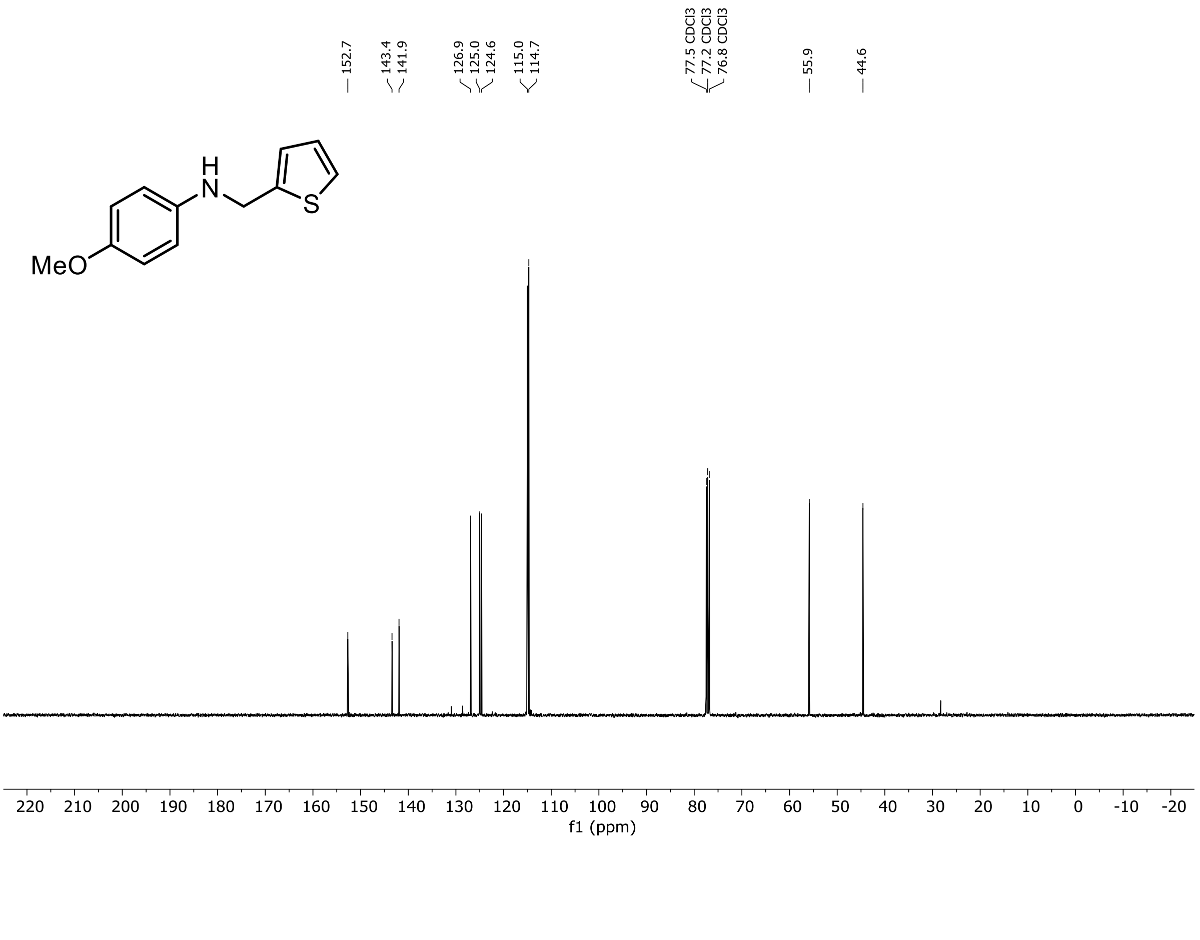
152.7
143.4
141.9
126.9
125.0
124.6
115.0
114.7
77.5 CDCl3
77.2 CDCl3
76.8 CDCl3
55.9
44.6
MeO
H
N
S
f1 (ppm)

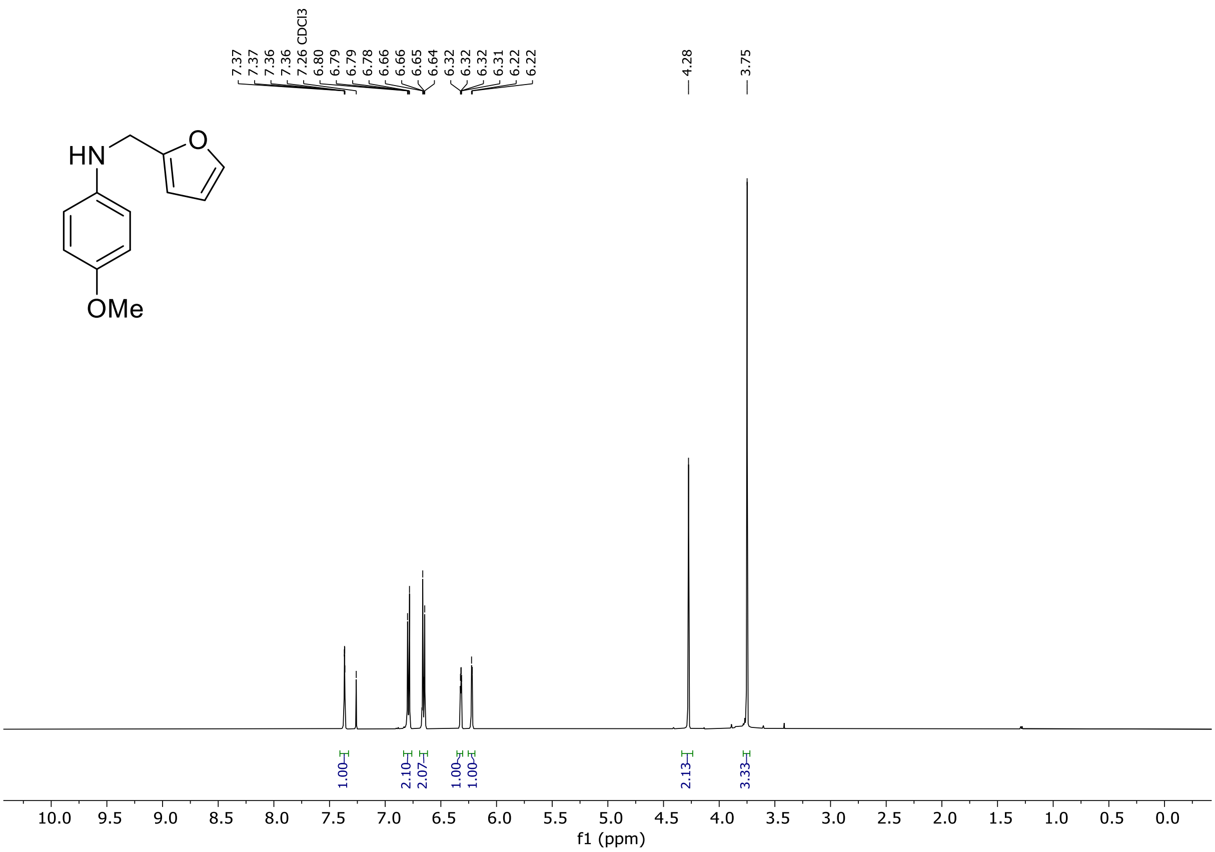
7.37
7.37
7.36
7.36
7.26 CDCl3
6.80
6.79
6.79
6.78
6.66
6.66
6.65
6.64
6.32
6.32
6.32
6.31
6.22
6.22
4.28
3.75
HN
O
OMe
1.00
2.10
2.07
1.00
1.00
2.13
3.33
10.0 9.5 9.0 8.5 8.0 7.5 7.0 6.5 6.0 5.5 5.0 4.5 4.0 3.5 3.0 2.5 2.0 1.5 1.0 0.5 0.0
f1 (ppm)

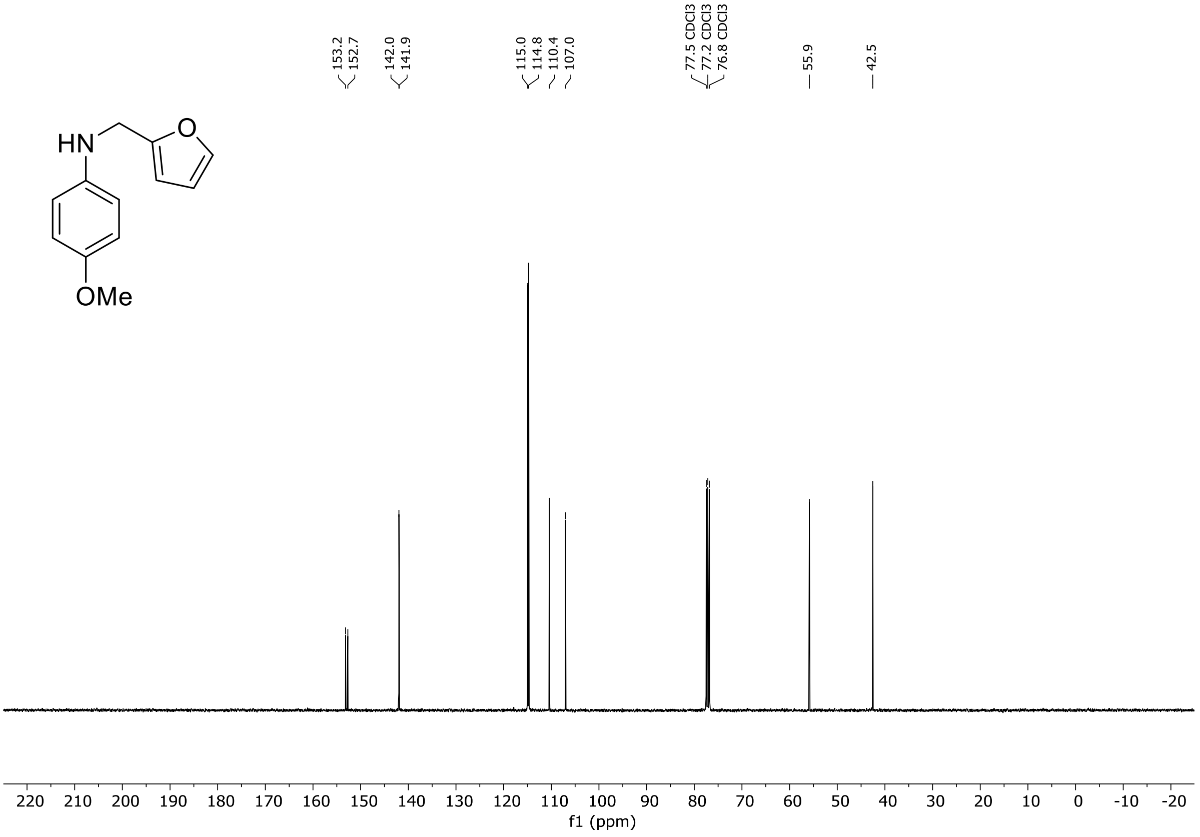
153.2
152.7
142.0
141.9
115.0
114.8
110.4
107.0
77.5 CDCl3
77.2 CDCl3
76.8 CDCl3
55.9
42.5
HN
O
OMe
220 210 200 190 180 170 160 150 140 130 120 110 100 90 80 70 60 50 40 30 20 10 0 -10 -20
f1 (ppm)

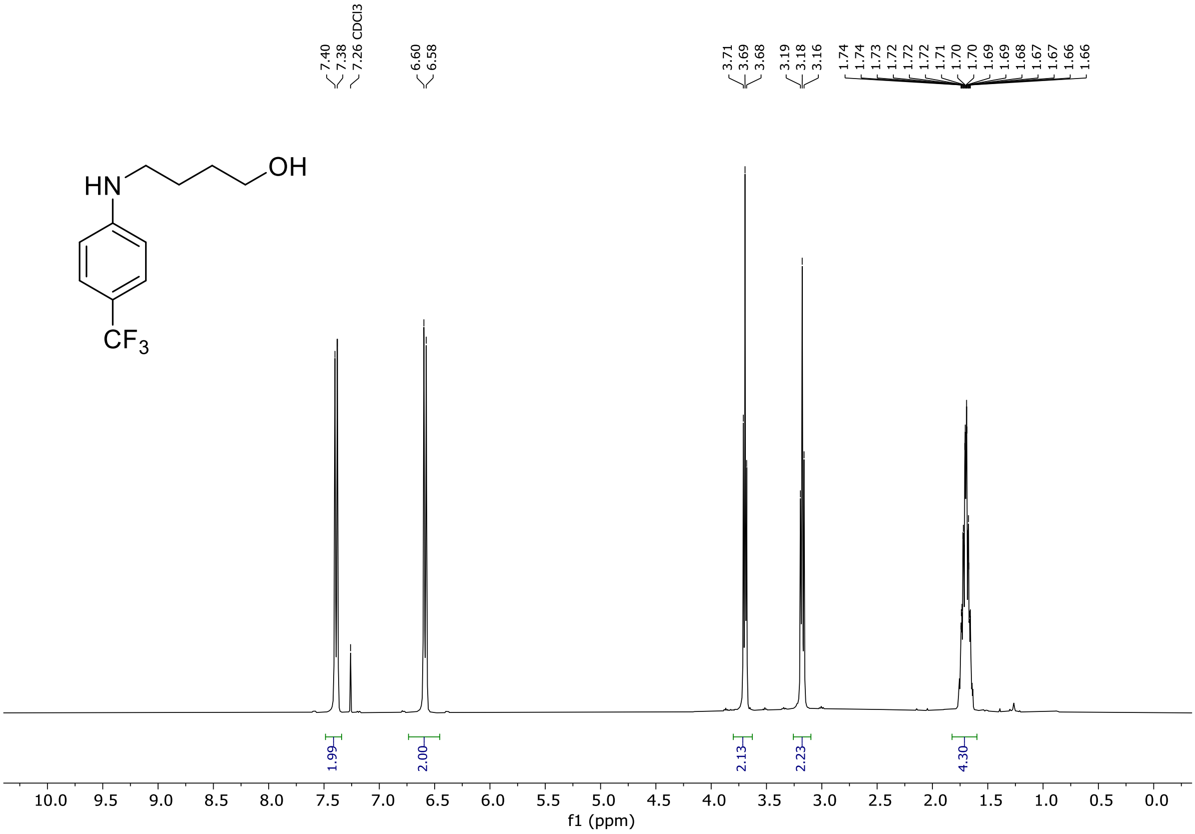
7.40
7.38
7.26 CDCl3
6.60
6.58
3.71
3.69
3.68
3.19
3.18
3.16
1.74
1.74
1.73
1.72
1.72
1.72
1.71
1.70
1.70
1.69
1.69
1.68
1.67
1.67
1.66
1.66
HN
OH
CF3
1.99
2.00
2.13
2.23
4.30
10.0 9.5 9.0 8.5 8.0 7.5 7.0 6.5 6.0 5.5 5.0 4.5 4.0 3.5 3.0 2.5 2.0 1.5 1.0 0.5 0.0
f1 (ppm)

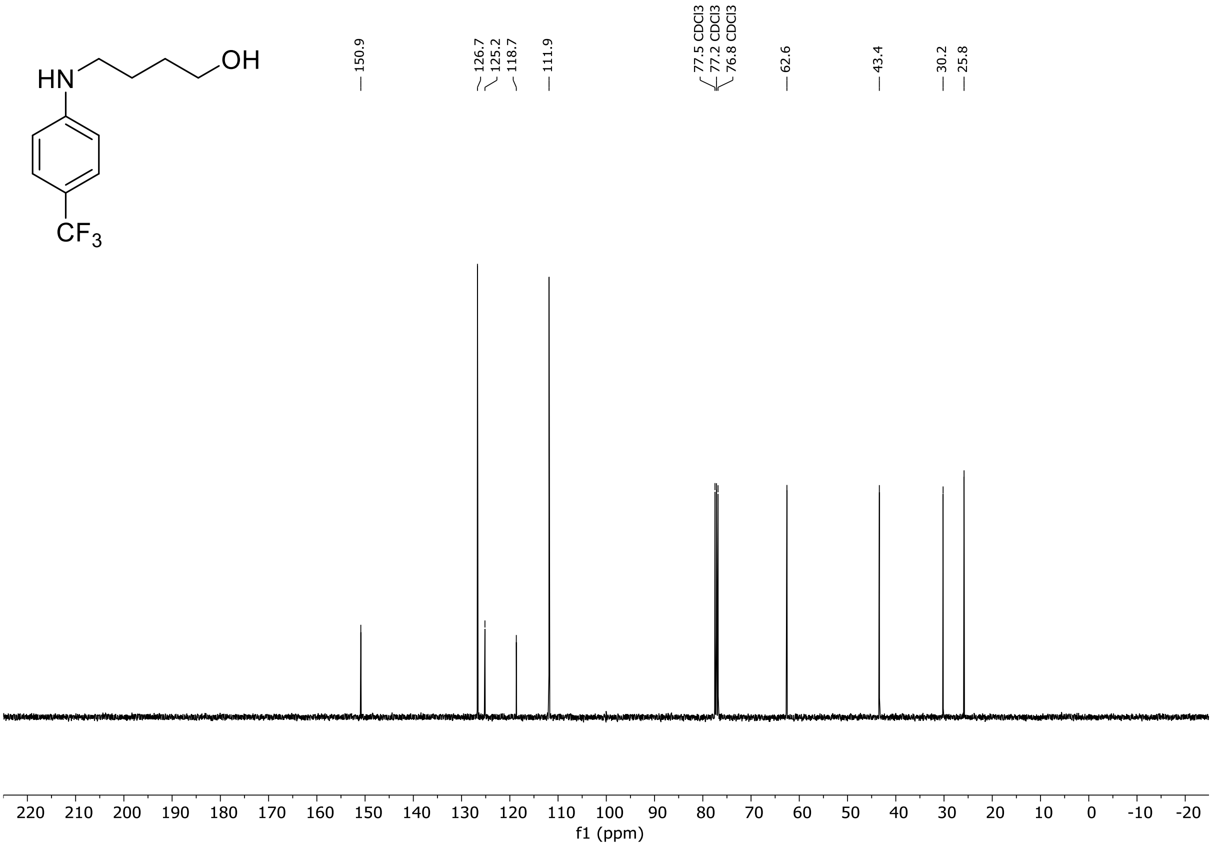
150.9
126.7
125.2
118.7
111.9
77.5 CDCl3
77.2 CDCl3
76.8 CDCl3
62.6
43.4
30.2
25.8
HN
OH
CF3
220 210 200 190 180 170 160 150 140 130 120 110 100 90 80 70 60 50 40 30 20 10 0 -10 -20
f1 (ppm)

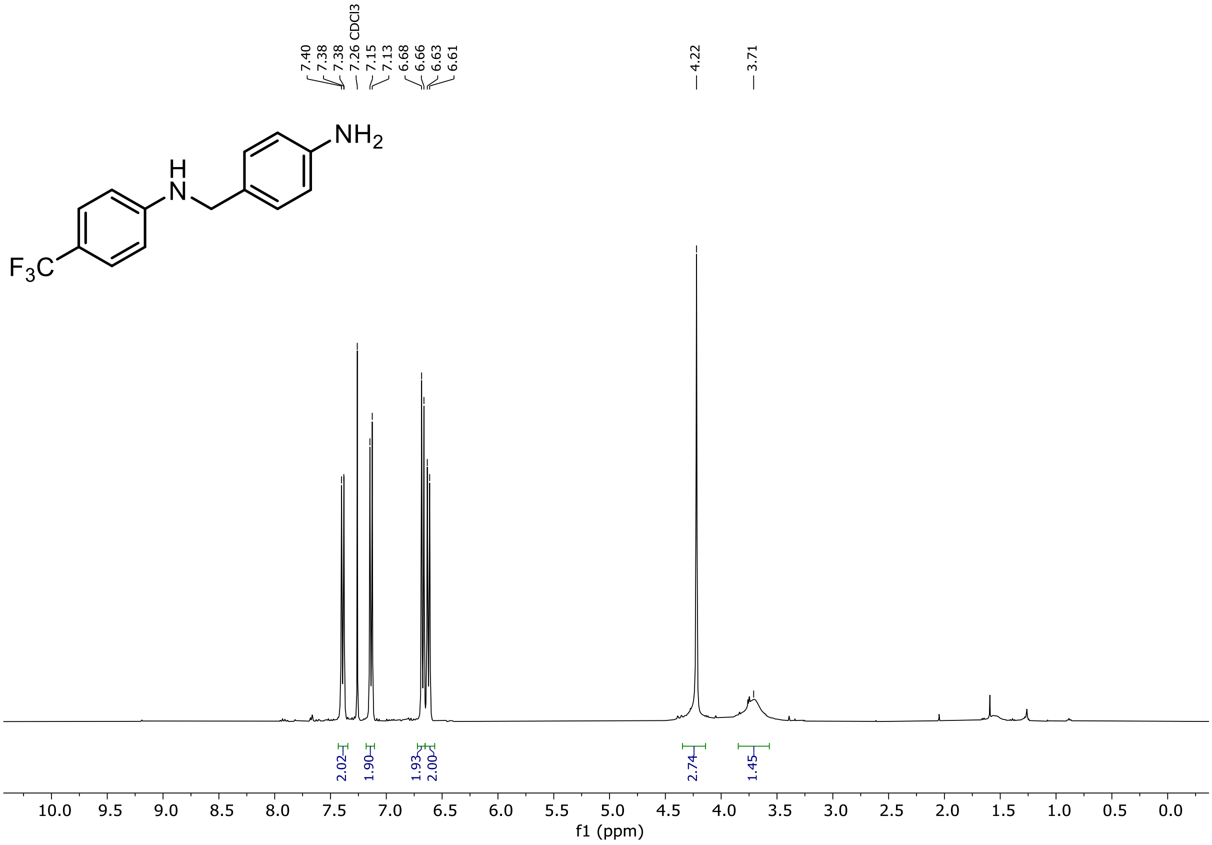
7.40
7.38
7.38
7.26 CDCl3
7.15
7.13
6.68
6.66
6.63
6.61
4.22
3.71
NH2
H
N
F3C
2.02
1.90
1.93
2.00
2.74
1.45
10.0 9.5 9.0 8.5 8.0 7.5 7.0 6.5 6.0 5.5 5.0 4.5 4.0 3.5 3.0 2.5 2.0 1.5 1.0 0.5 0.0
f1 (ppm)

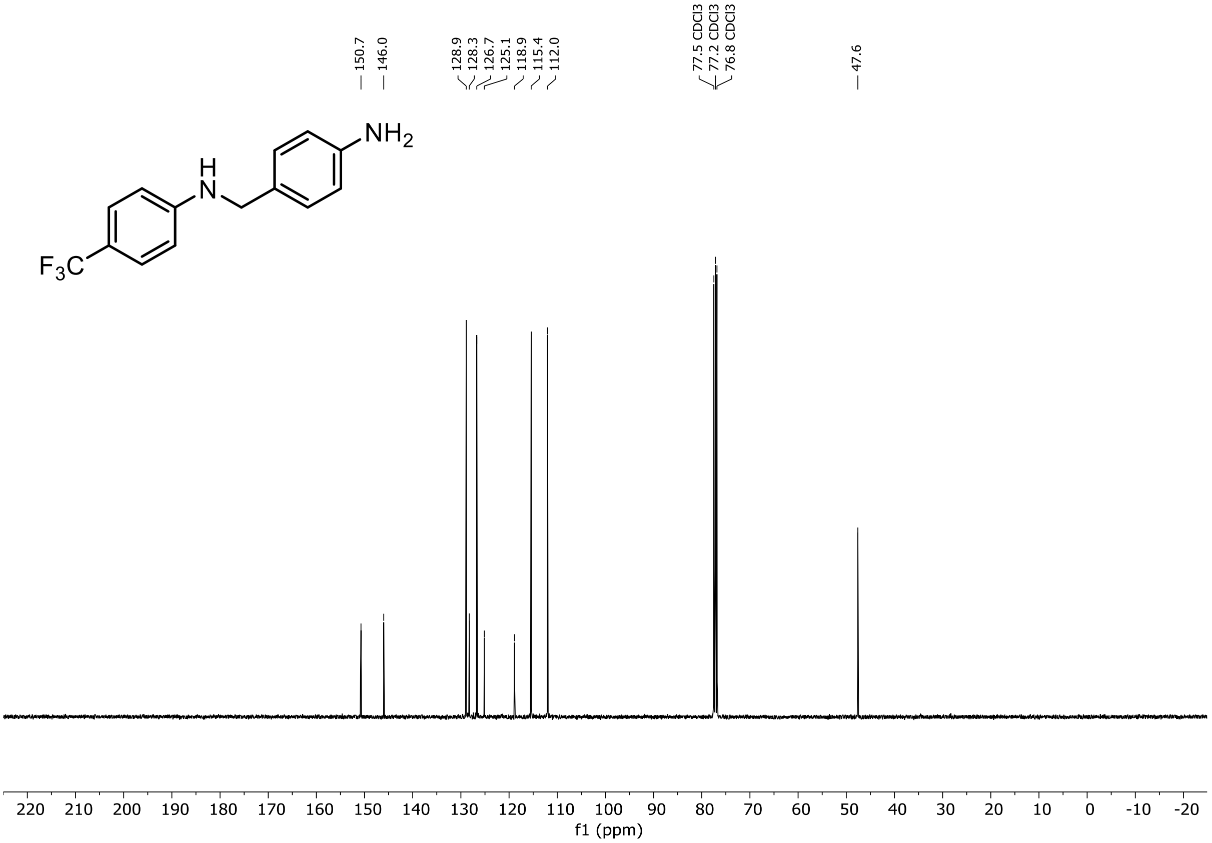
150.7
146.0
128.9
128.3
126.7
125.1
118.9
115.4
112.0
77.5 CDCl3
77.2 CDCl3
76.8 CDCl3
47.6
NH2
H
N
F3C
220 210 200 190 180 170 160 150 140 130 120 110 100 90 80 70 60 50 40 30 20 10 0 -10 -20
f1 (ppm)

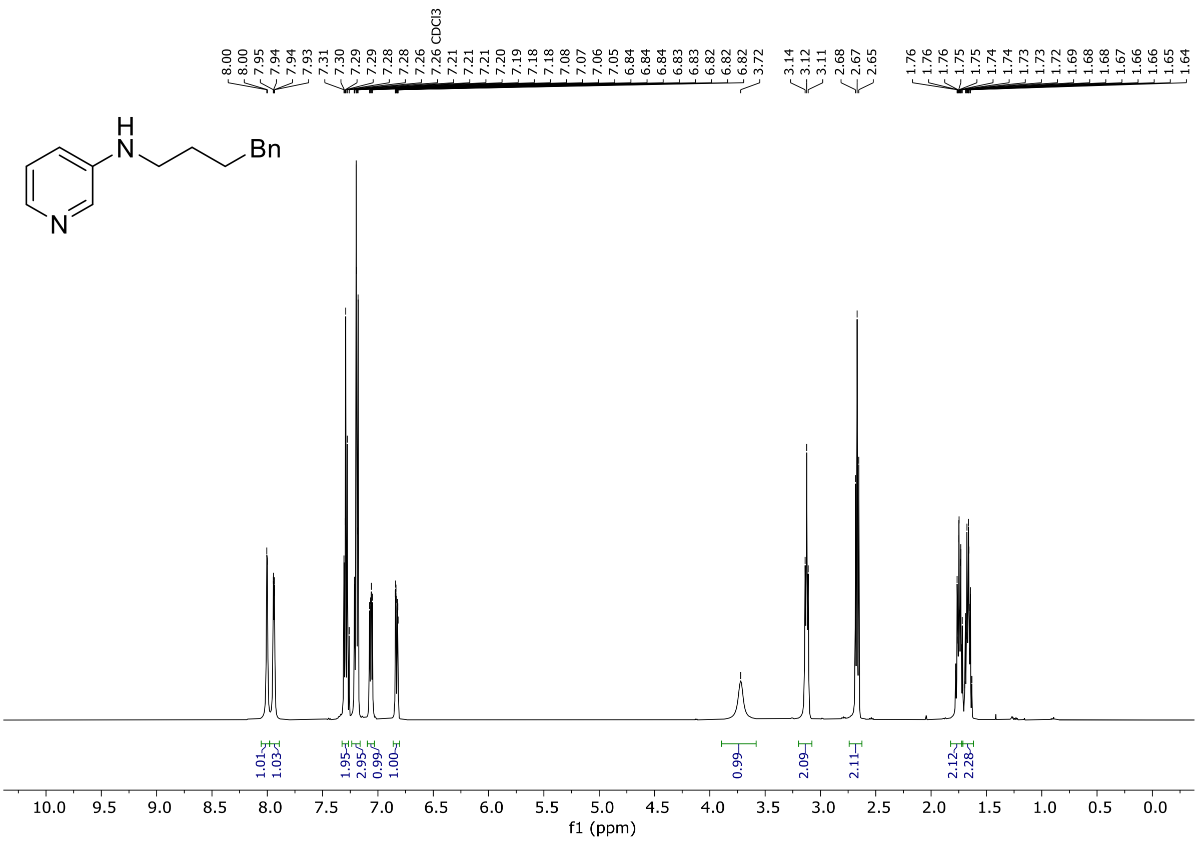

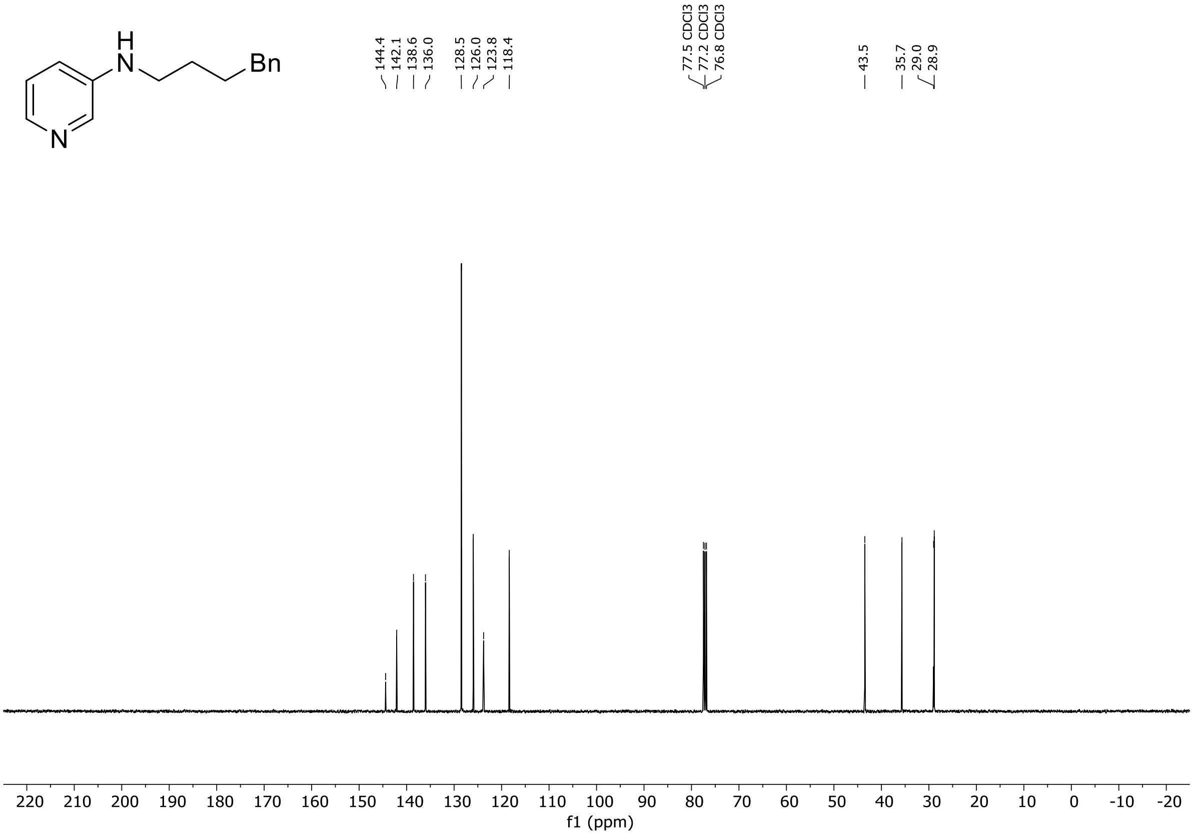

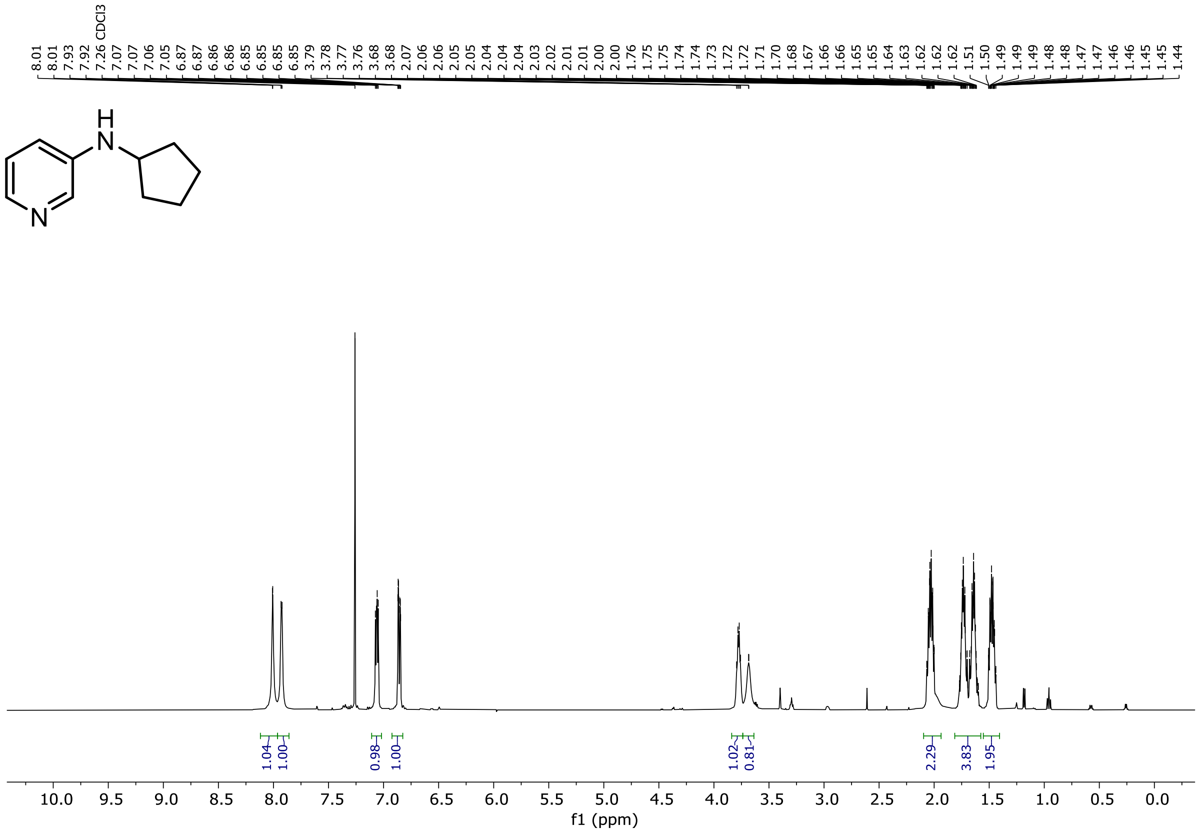
H
N
N
1.04
1.00
0.98
1.00
1.02
0.81
2.29
3.83
1.95
f1 (ppm)

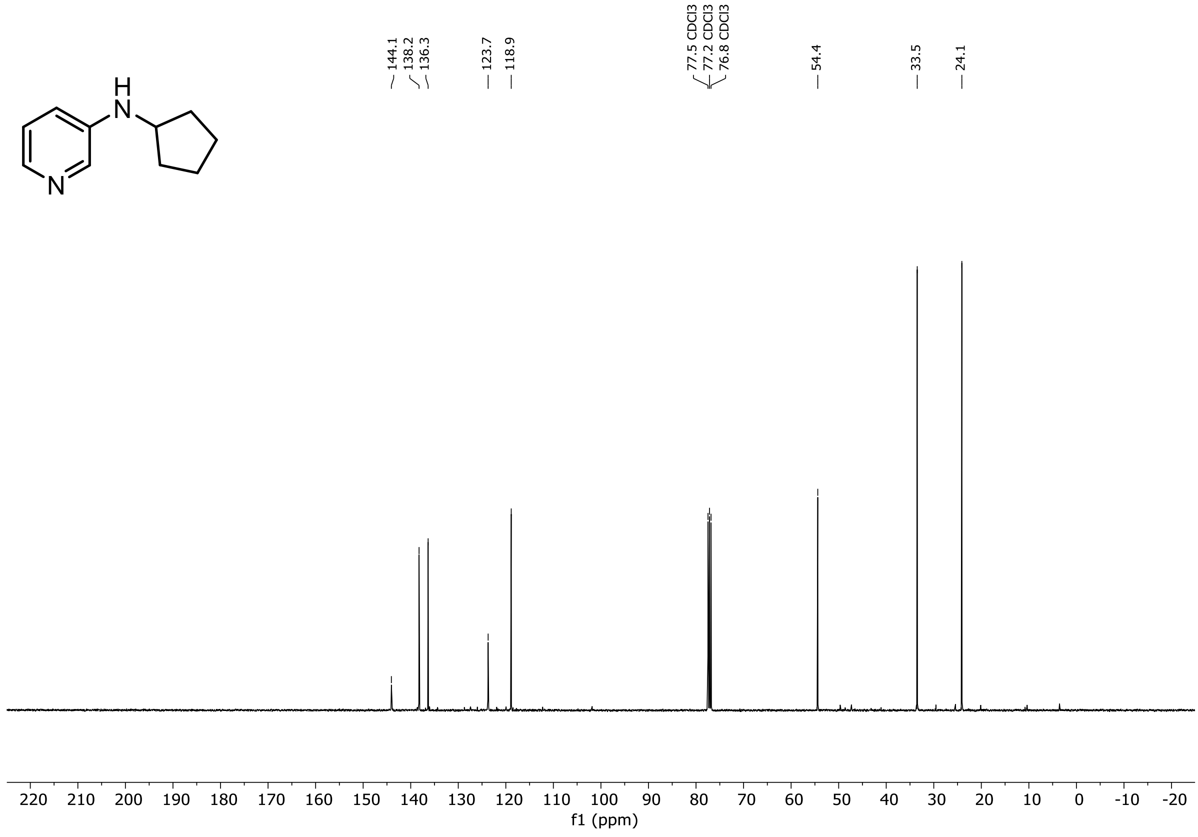
H
N
N
144.1
138.2
136.3
123.7
118.9
77.5 CDCl3
77.2 CDCl3
76.8 CDCl3
54.4
33.5
24.1
f1 (ppm)

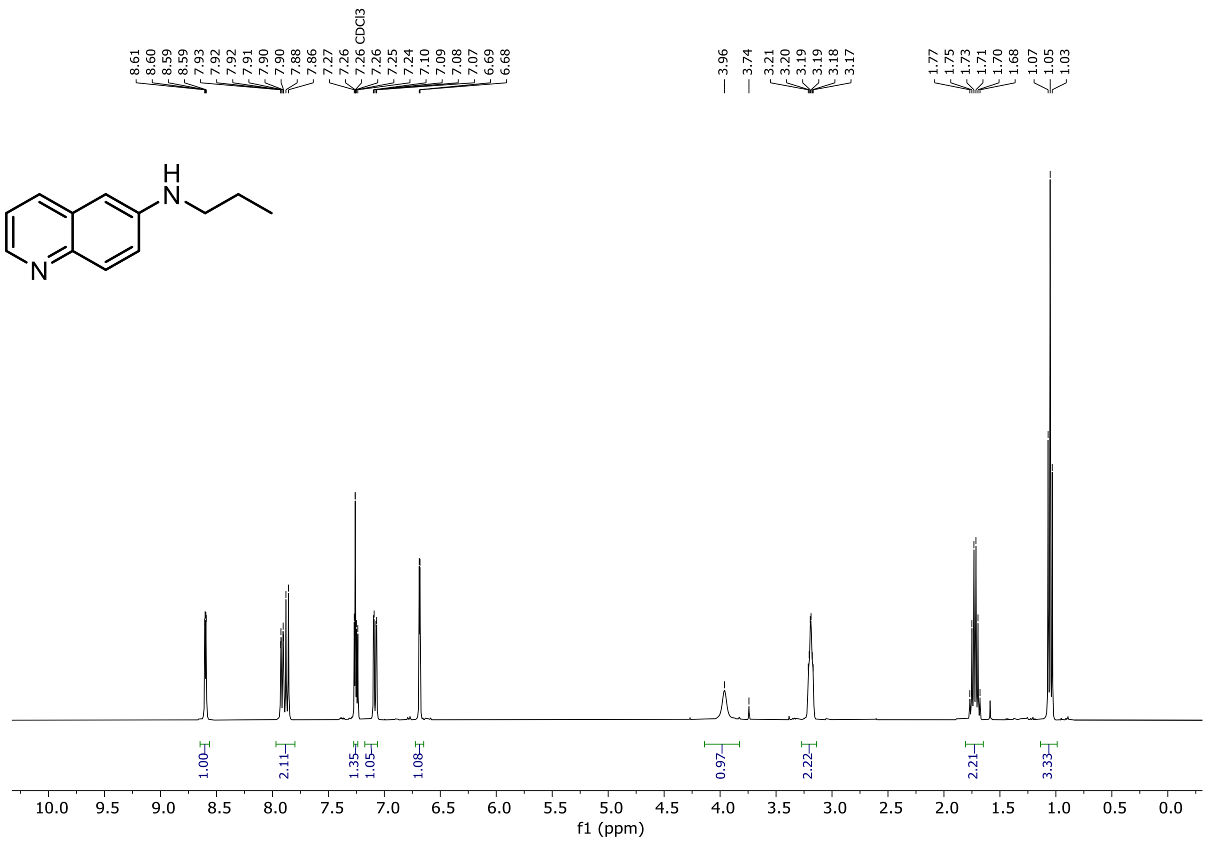

8.61
8.60
8.59
8.59
7.93
7.92
7.91
7.90
7.90
7.88
7.86
7.27
7.26
7.26 CDCl3
7.26
7.25
7.24
7.10
7.09
7.08
7.07
6.69
6.68
3.96
3.74
3.21
3.20
3.19
3.19
3.18
3.17
1.77
1.75
1.73
1.71
1.70
1.68
1.07
1.05
1.03
1.00
2.11
1.35
1.05
1.08
0.97
2.22
2.21
3.33
f1 (ppm)

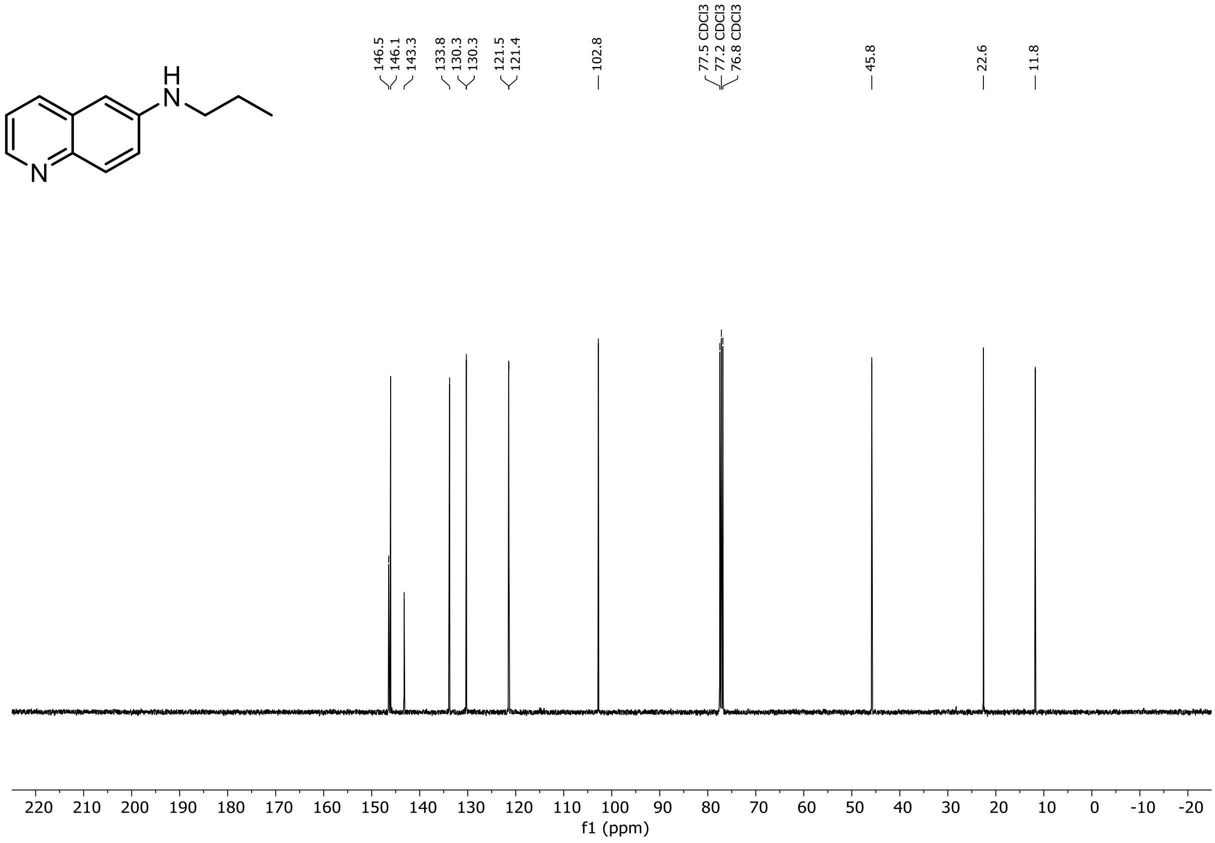

146.5
146.1
143.3
133.8
130.3
130.3
121.5
121.4
102.8
77.5 CDCl3
77.2 CDCl3
76.8 CDCl3
45.8
22.6
11.8
f1 (ppm)

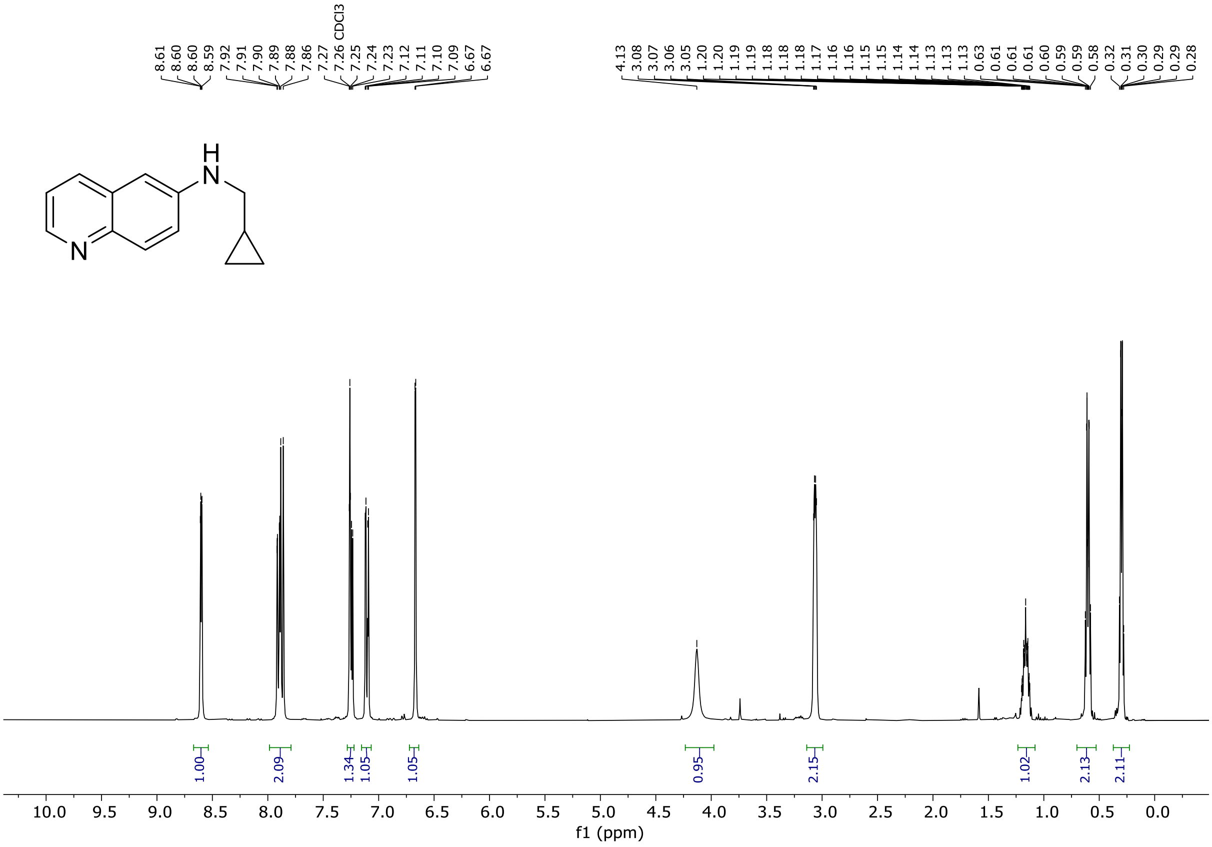
8.61
8.60
8.60
8.59
7.92
7.91
7.90
7.89
7.88
7.86
7.27
7.26 CDCl3
7.25
7.24
7.23
7.12
7.11
7.10
7.09
6.67
6.67
4.13
3.08
3.07
3.06
3.05
1.20
1.20
1.19
1.19
1.18
1.18
1.18
1.17
1.16
1.16
1.15
1.15
1.14
1.14
1.13
1.13
1.13
0.63
0.61
0.61
0.61
0.60
0.59
0.59
0.58
0.32
0.31
0.30
0.29
0.29
0.28
H
N
N
1.00
2.09
1.34
1.05
1.05
0.95
2.15
1.02
2.13
2.11
10.0 9.5 9.0 8.5 8.0 7.5 7.0 6.5 6.0 5.5 5.0 4.5 4.0 3.5 3.0 2.5 2.0 1.5 1.0 0.5 0.0
f1 (ppm)

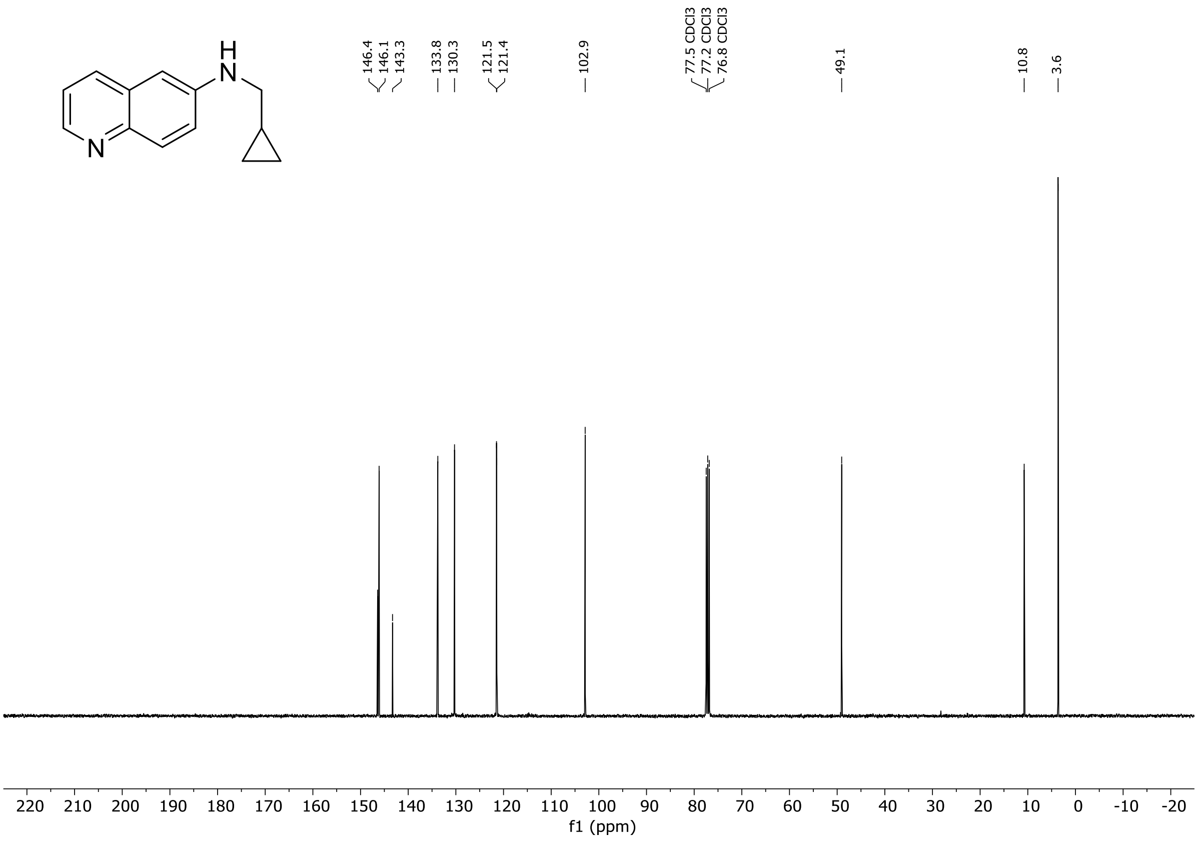
H
N
N
146.4
146.1
143.3
133.8
130.3
121.5
121.4
102.9
77.5 CDCl3
77.2 CDCl3
76.8 CDCl3
49.1
10.8
3.6
220 210 200 190 180 170 160 150 140 130 120 110 100 90 80 70 60 50 40 30 20 10 0 -10 -20
f1 (ppm)

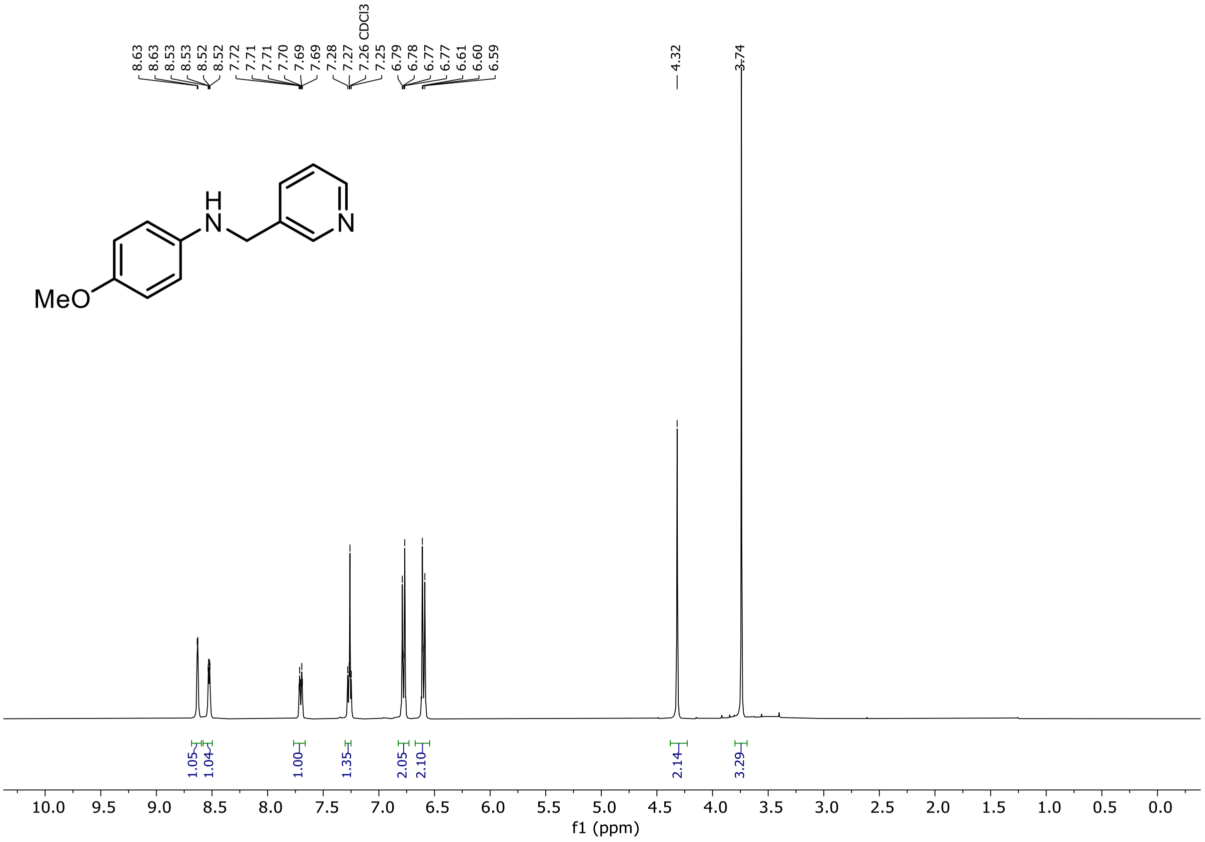
8.63
8.63
8.53
8.53
8.52
8.52
7.72
7.71
7.71
7.70
7.69
7.69
7.28
7.27
7.26 CDCl3
7.25
6.79
6.78
6.77
6.77
6.61
6.60
6.59
4.32
3.74
H
N
N
MeO
1.05
1.04
1.00
1.35
2.05
2.10
2.14
3.29
10.0 9.5 9.0 8.5 8.0 7.5 7.0 6.5 6.0 5.5 5.0 4.5 4.0 3.5 3.0 2.5 2.0 1.5 1.0 0.5 0.0
f1 (ppm)

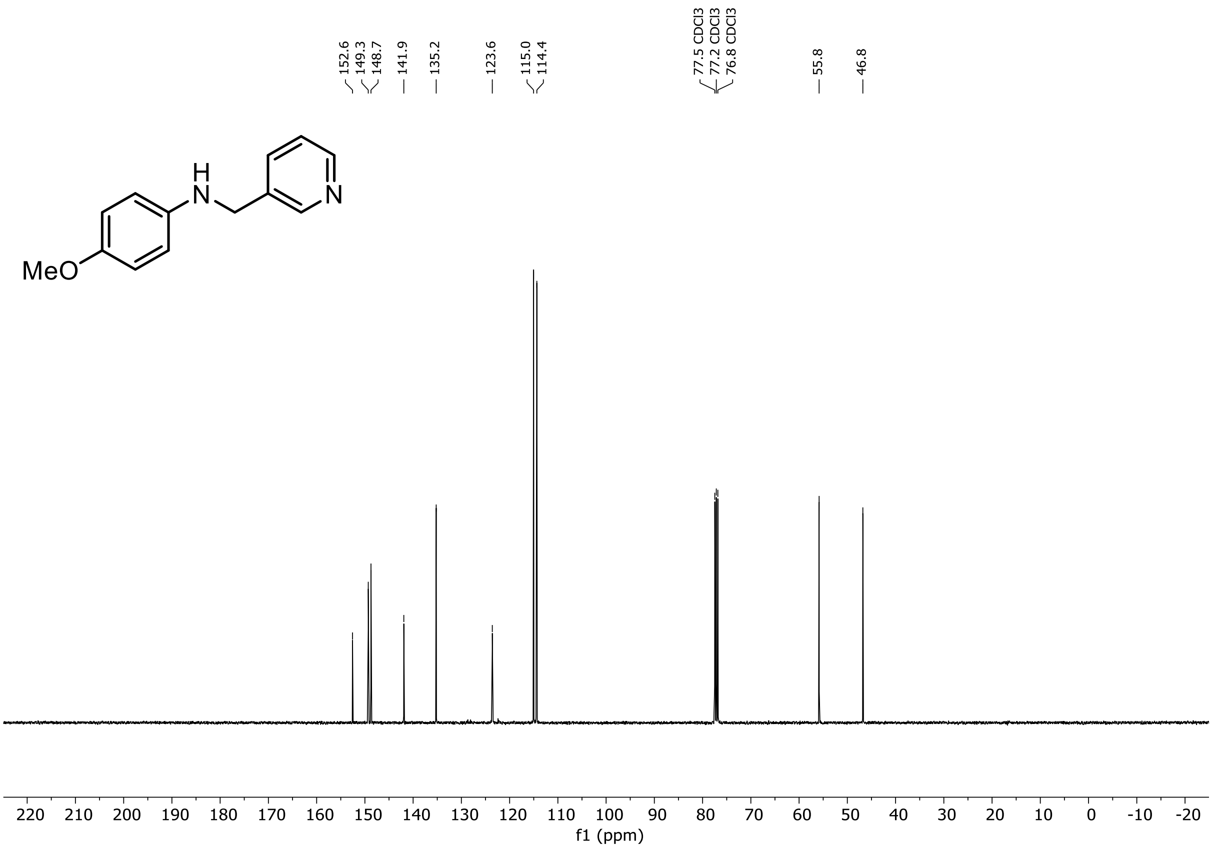
152.6
149.3
148.7
141.9
135.2
123.6
115.0
114.4
77.5 CDCl3
77.2 CDCl3
76.8 CDCl3
55.8
46.8
H
N
N
MeO
220 210 200 190 180 170 160 150 140 130 120 110 100 90 80 70 60 50 40 30 20 10 0 -10 -20
f1 (ppm)

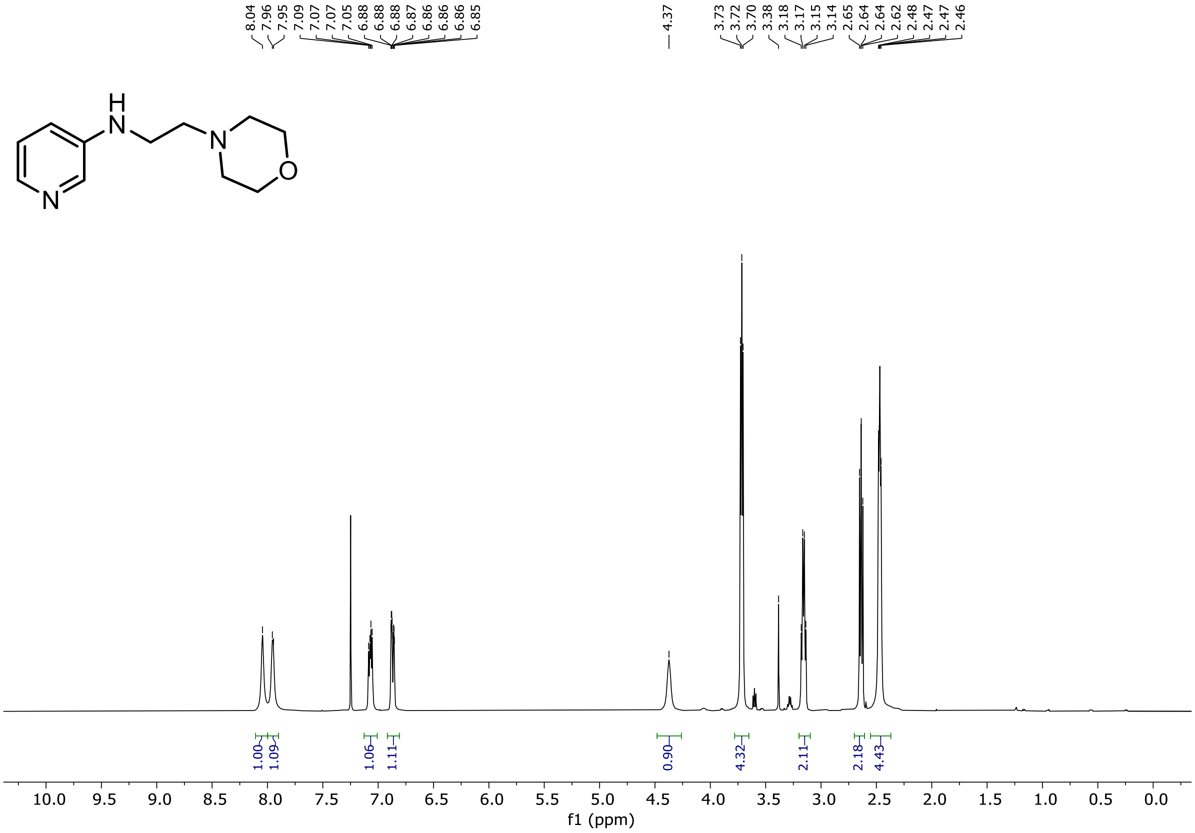
8.04
7.96
7.95
7.09
7.07
7.07
7.05
6.88
6.88
6.88
6.87
6.86
6.86
6.86
6.85
4.37
3.73
3.72
3.70
3.38
3.18
3.17
3.15
3.14
2.65
2.64
2.64
2.62
2.48
2.47
2.46
H
N
N
O
N
1.00
1.09
1.06
1.11
0.90
4.32
2.11
2.18
4.43
10.0 9.5 9.0 8.5 8.0 7.5 7.0 6.5 6.0 5.5 5.0 4.5 4.0 3.5 3.0 2.5 2.0 1.5 1.0 0.5 0.0
f1 (ppm)

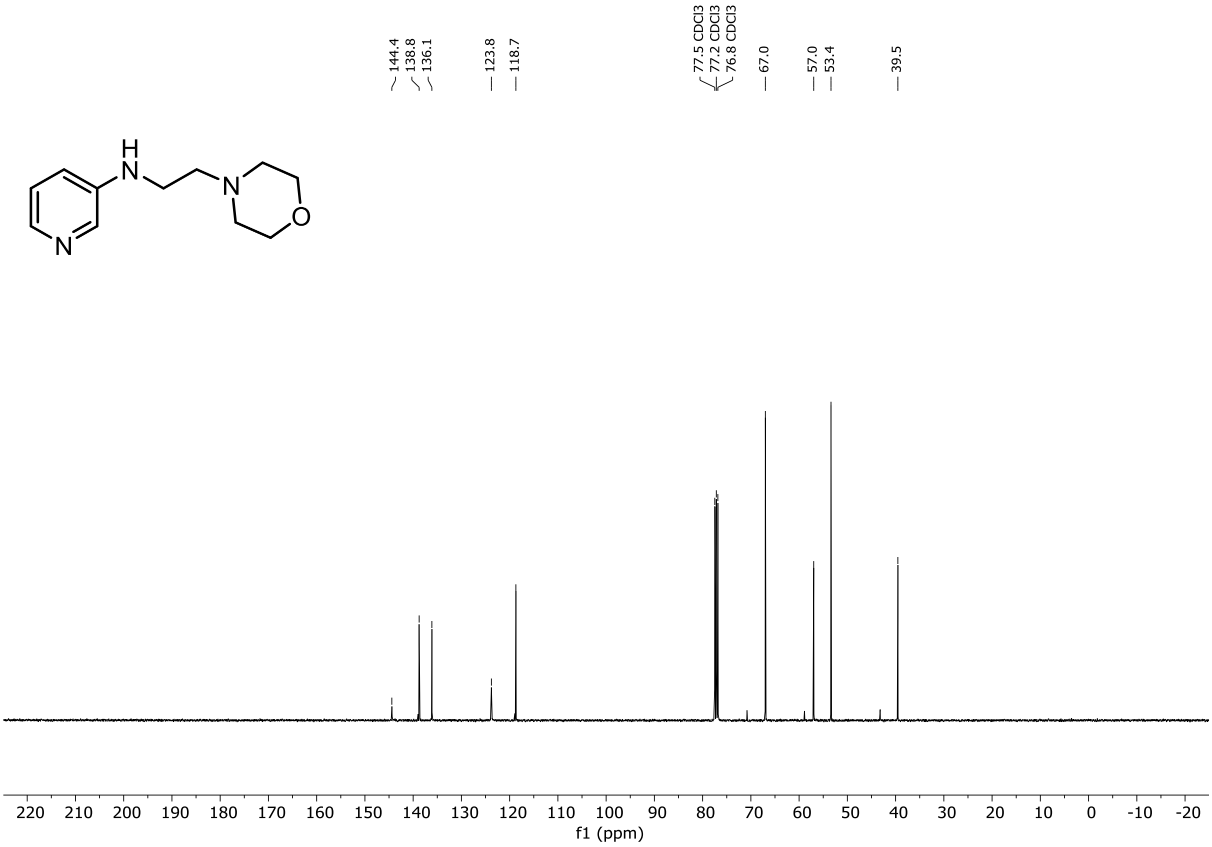
144.4
138.8
136.1
123.8
118.7
77.5 CDCl3
77.2 CDCl3
76.8 CDCl3
67.0
57.0
53.4
39.5
H
N
N
O
N
220 210 200 190 180 170 160 150 140 130 120 110 100 90 80 70 60 50 40 30 20 10 0 -10 -20
f1 (ppm)

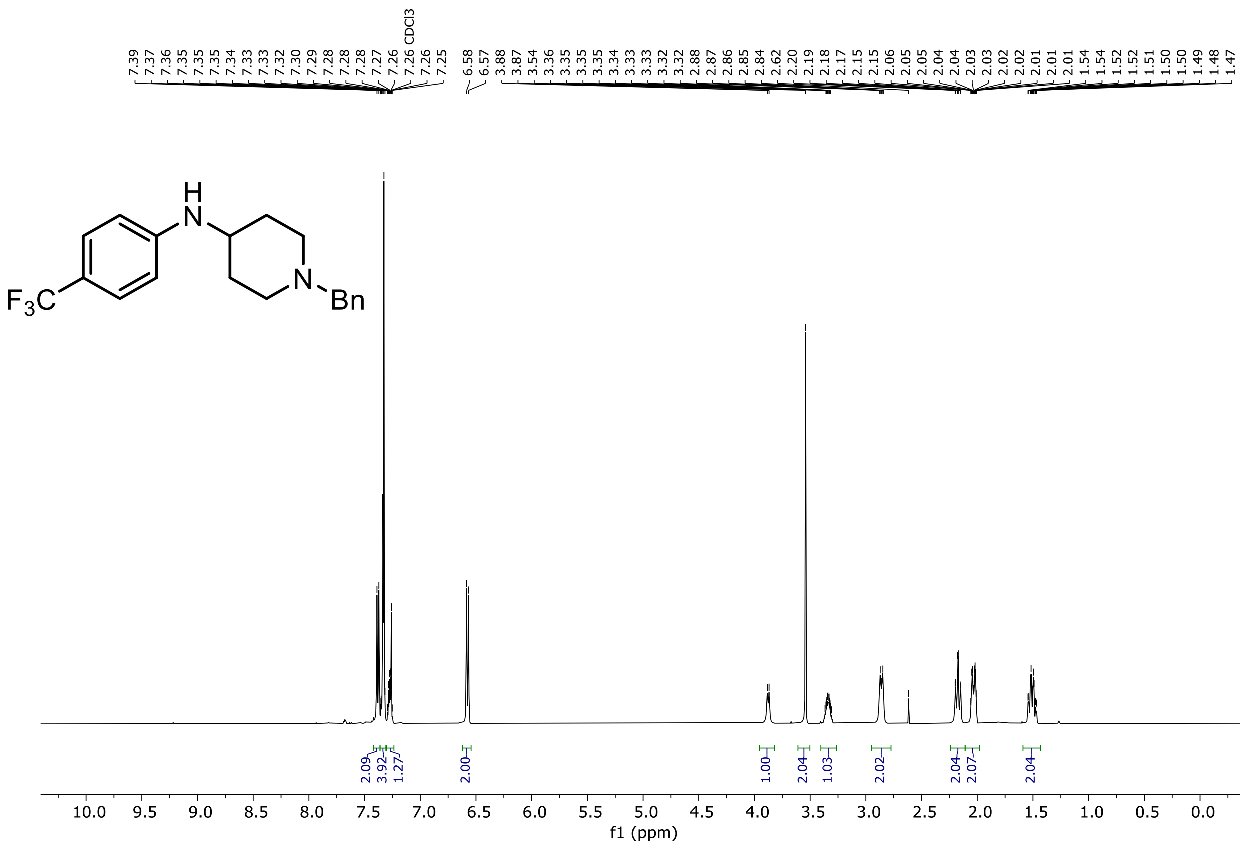
7.39 7.37 7.36 7.35 7.35 7.34 7.33 7.33 7.32 7.30 7.29 7.28 7.28 7.28 7.27 7.26 CDCl3 7.26 7.25
6.58 6.57
3.88 3.87 3.54 3.36 3.35 3.35 3.35 3.34 3.33 3.33 3.33 3.32 3.32 2.88 2.87 2.86 2.85 2.84 2.62 2.20 2.19 2.18 2.17 2.15 2.15 2.06 2.05 2.05 2.04 2.04 2.03 2.03 2.02 2.02 2.01 2.01 1.54 1.54 1.52 1.51 1.50 1.50 1.49 1.48 1.47
H
N
F3C
N
Bn
2.09 3.92 1.27 2.00 1.00 2.04 1.03 2.02 2.04 2.07 2.04
10.0 9.5 9.0 8.5 8.0 7.5 7.0 6.5 6.0 5.5 5.0 4.5 4.0 3.5 3.0 2.5 2.0 1.5 1.0 0.5 0.0
f1 (ppm)

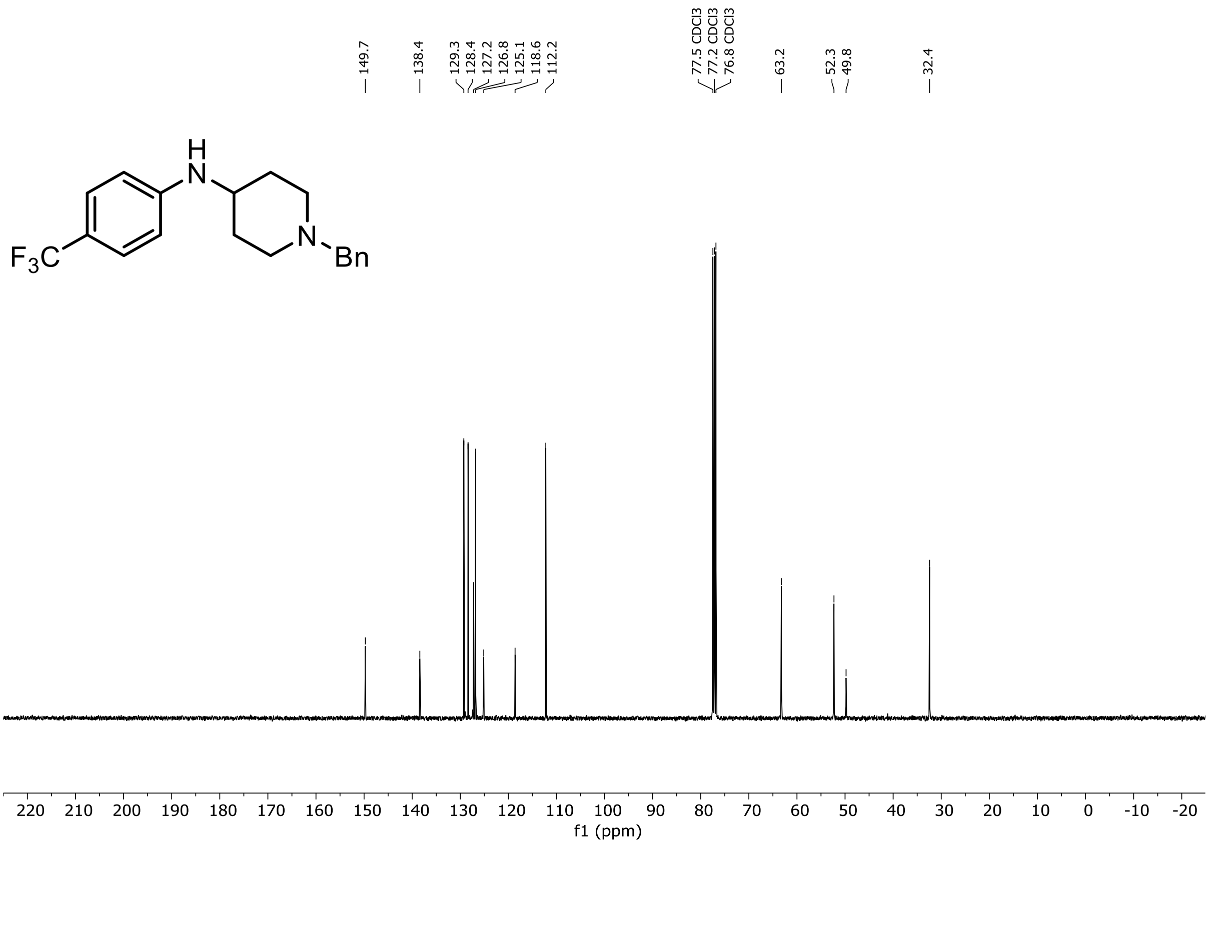
149.7
138.4
129.3 128.4 127.2 126.8 125.1 118.6 112.2
77.5 CDCl3 77.2 CDCl3 76.8 CDCl3
63.2
52.3 49.8
32.4
H
N
F3C
N
Bn
220 210 200 190 180 170 160 150 140 130 120 110 100 90 80 70 60 50 40 30 20 10 0 -10 -20
f1 (ppm)

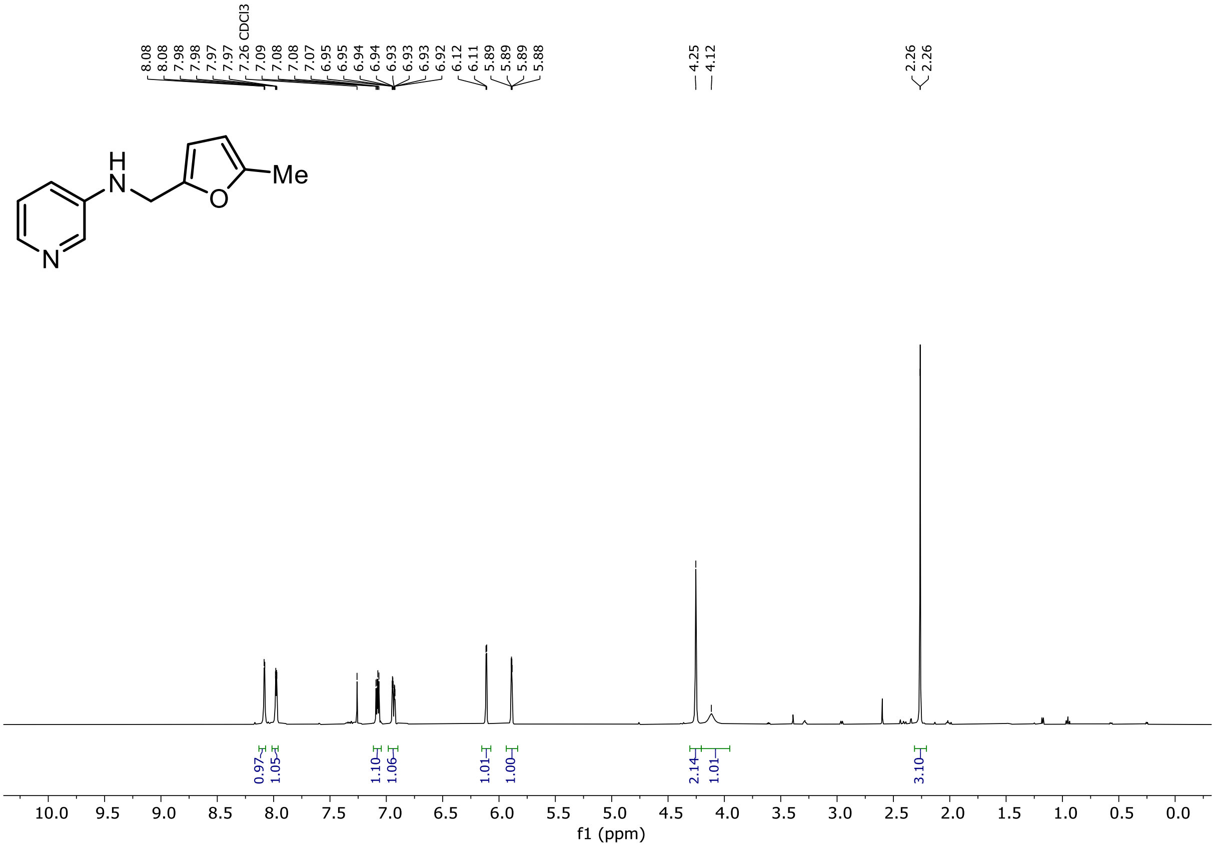
8.08
8.08
7.98
7.98
7.97
7.97
7.26 CDCl3
7.09
7.08
7.08
7.07
6.95
6.95
6.94
6.94
6.93
6.93
6.93
6.92
6.12
6.11
5.89
5.89
5.89
5.88
4.25
4.12
2.26
2.26
Me
N
H
O
N
0.97
1.05
1.10
1.06
1.01
1.00
2.14
1.01
3.10
10.0 9.5 9.0 8.5 8.0 7.5 7.0 6.5 6.0 5.5 5.0 4.5 4.0 3.5 3.0 2.5 2.0 1.5 1.0 0.5 0.0
f1 (ppm)

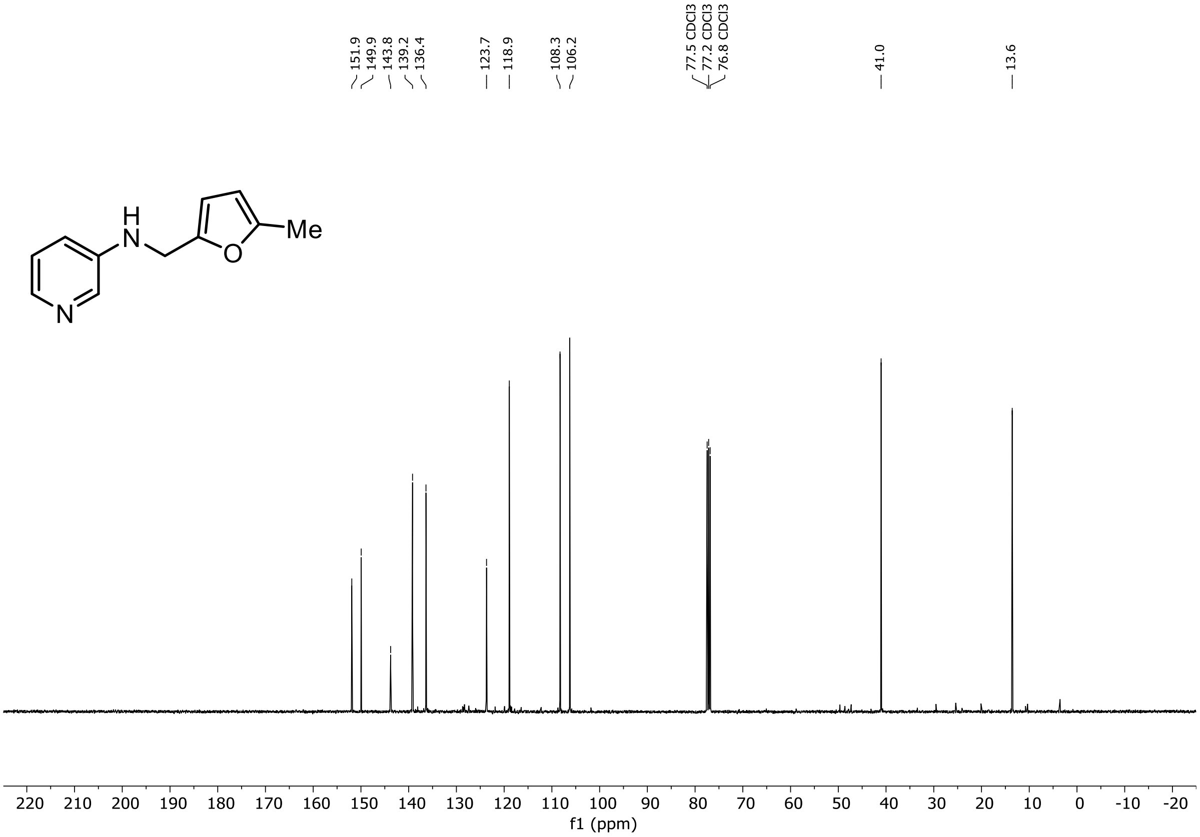
151.9
149.9
143.8
139.2
136.4
123.7
118.9
108.3
106.2
77.5 CDCl3
77.2 CDCl3
76.8 CDCl3
41.0
13.6
Me
N
H
O
N
220 210 200 190 180 170 160 150 140 130 120 110 100 90 80 70 60 50 40 30 20 10 0 -10 -20
f1 (ppm)

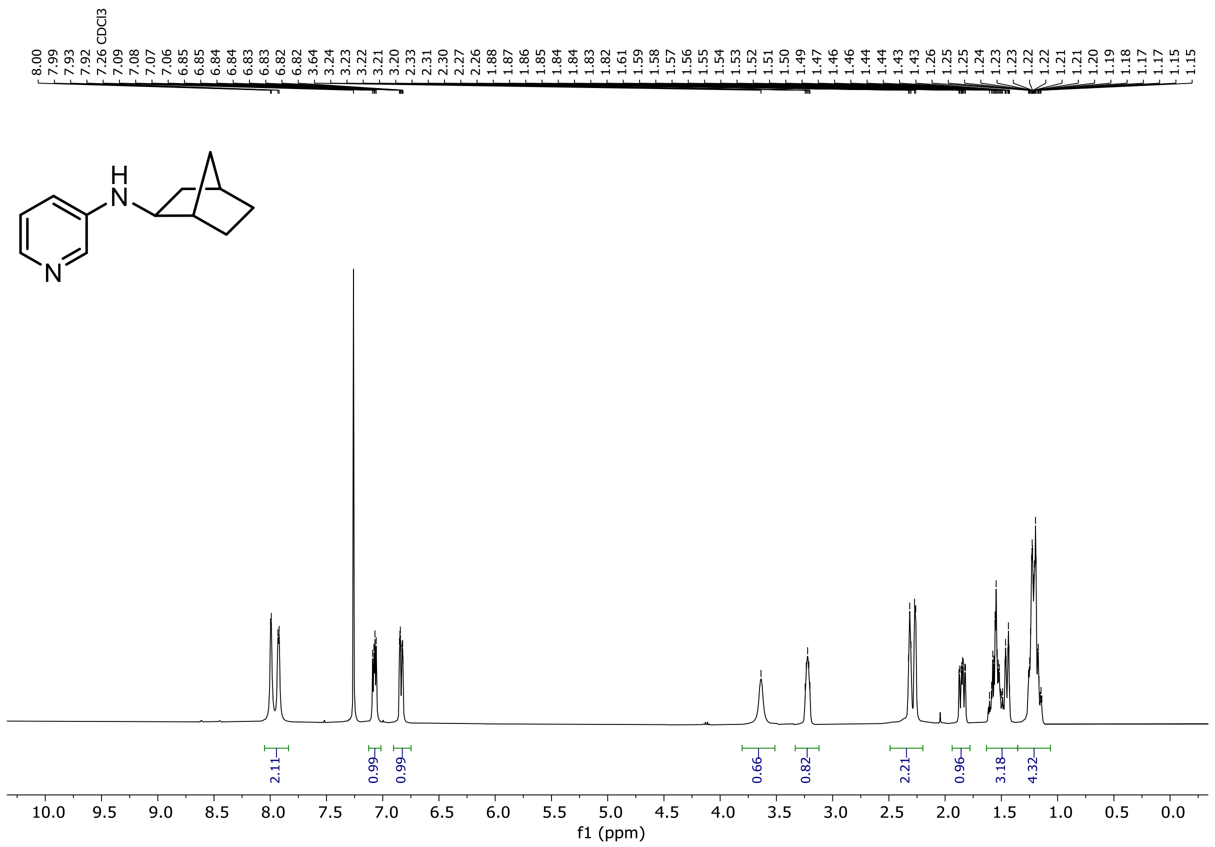

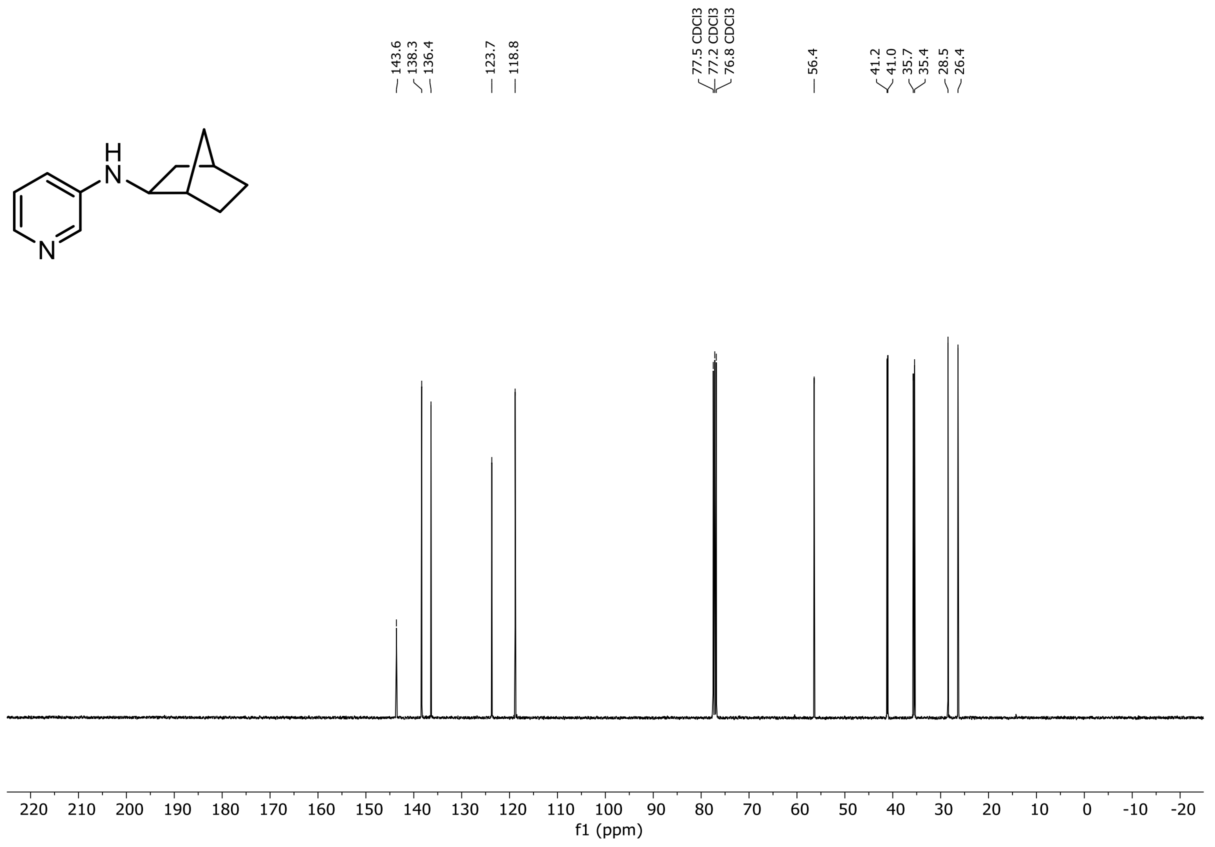

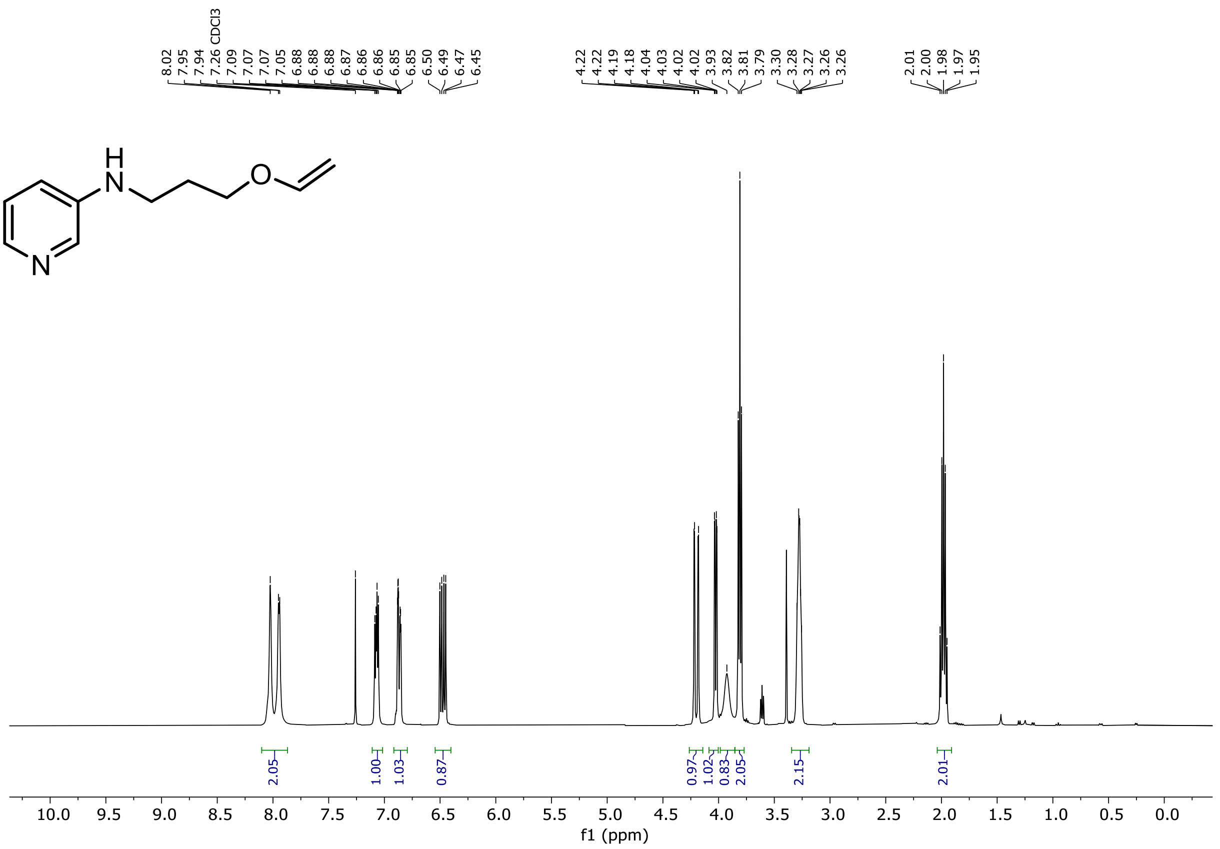
8.02
7.95
7.94
7.26 CDCl3
7.09
7.07
7.07
7.05
6.88
6.88
6.88
6.87
6.86
6.86
6.85
6.85
6.50
6.49
6.47
6.45
4.22
4.22
4.19
4.18
4.04
4.03
4.02
4.02
3.93
3.82
3.81
3.79
3.30
3.28
3.27
3.26
3.26
2.01
2.00
1.98
1.97
1.95
N
H
O
2.05
1.00
1.03
0.87
0.97
1.02
0.83
2.05
2.15
2.01
10.0 9.5 9.0 8.5 8.0 7.5 7.0 6.5 6.0 5.5 5.0 4.5 4.0 3.5 3.0 2.5 2.0 1.5 1.0 0.5 0.0
f1 (ppm)

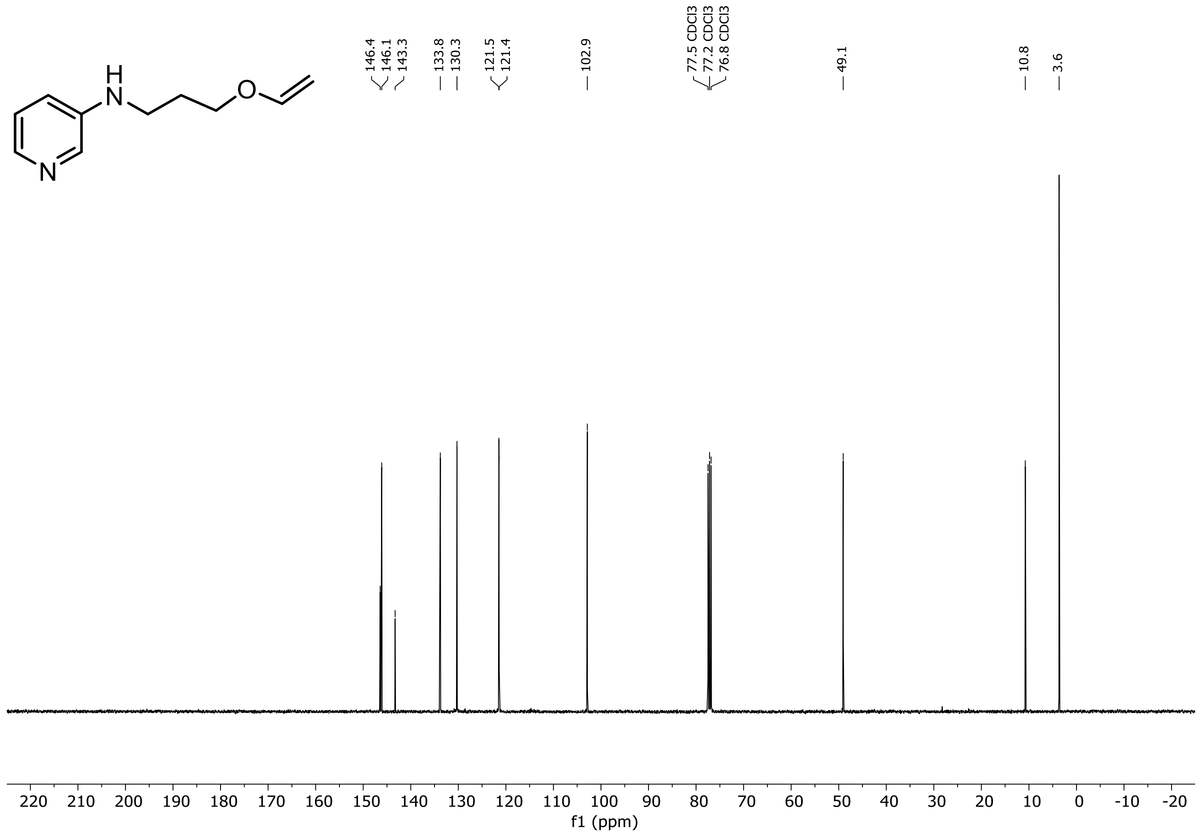
146.4
146.1
143.3
133.8
130.3
121.5
121.4
102.9
77.5 CDCl3
77.2 CDCl3
76.8 CDCl3
49.1
10.8
3.6
N
H
O
220 210 200 190 180 170 160 150 140 130 120 110 100 90 80 70 60 50 40 30 20 10 0 -10 -20
f1 (ppm)

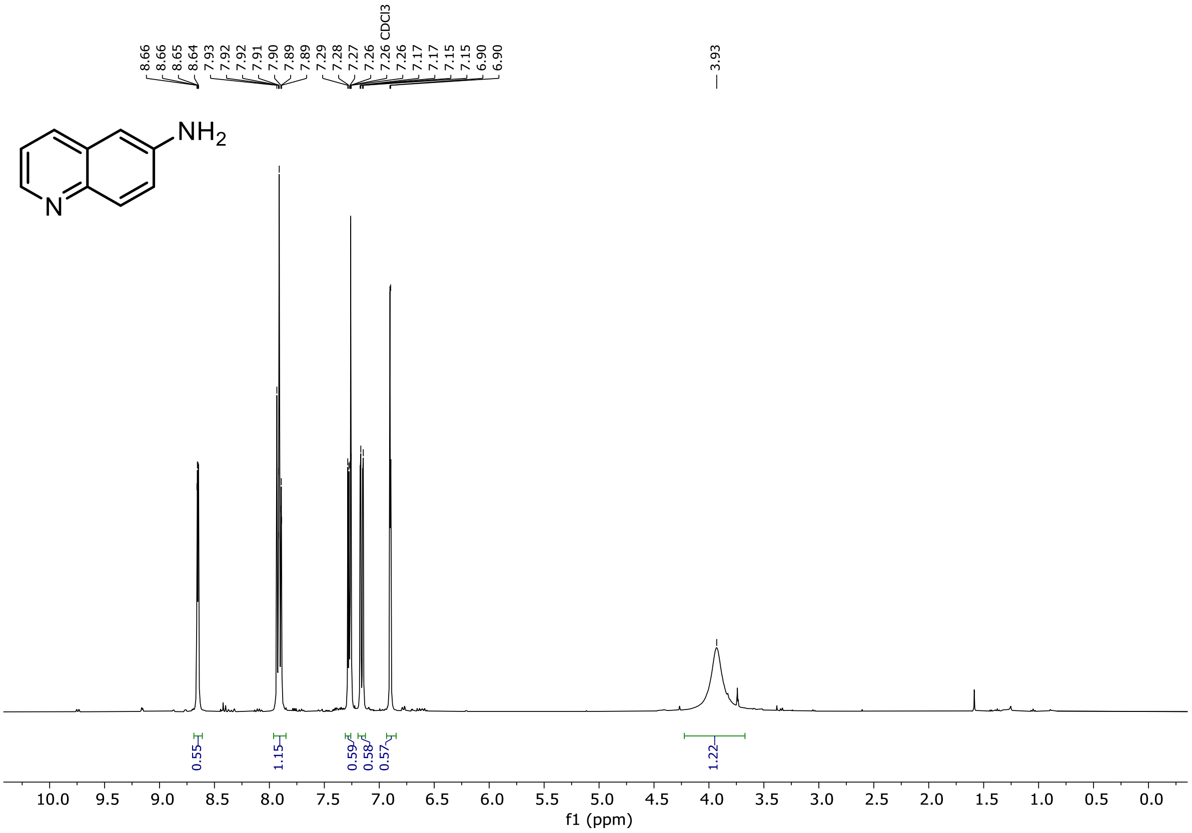
NH2
N
8.66
8.66
8.65
8.64
7.93
7.92
7.92
7.91
7.90
7.89
7.89
7.29
7.28
7.27
7.26
7.26 CDCl3
7.26
7.17
7.17
7.15
7.15
6.90
6.90
3.93
0.55
1.15
0.59
0.58
0.57
1.22
10.0 9.5 9.0 8.5 8.0 7.5 7.0 6.5 6.0 5.5 5.0 4.5 4.0 3.5 3.0 2.5 2.0 1.5 1.0 0.5 0.0
f1 (ppm)

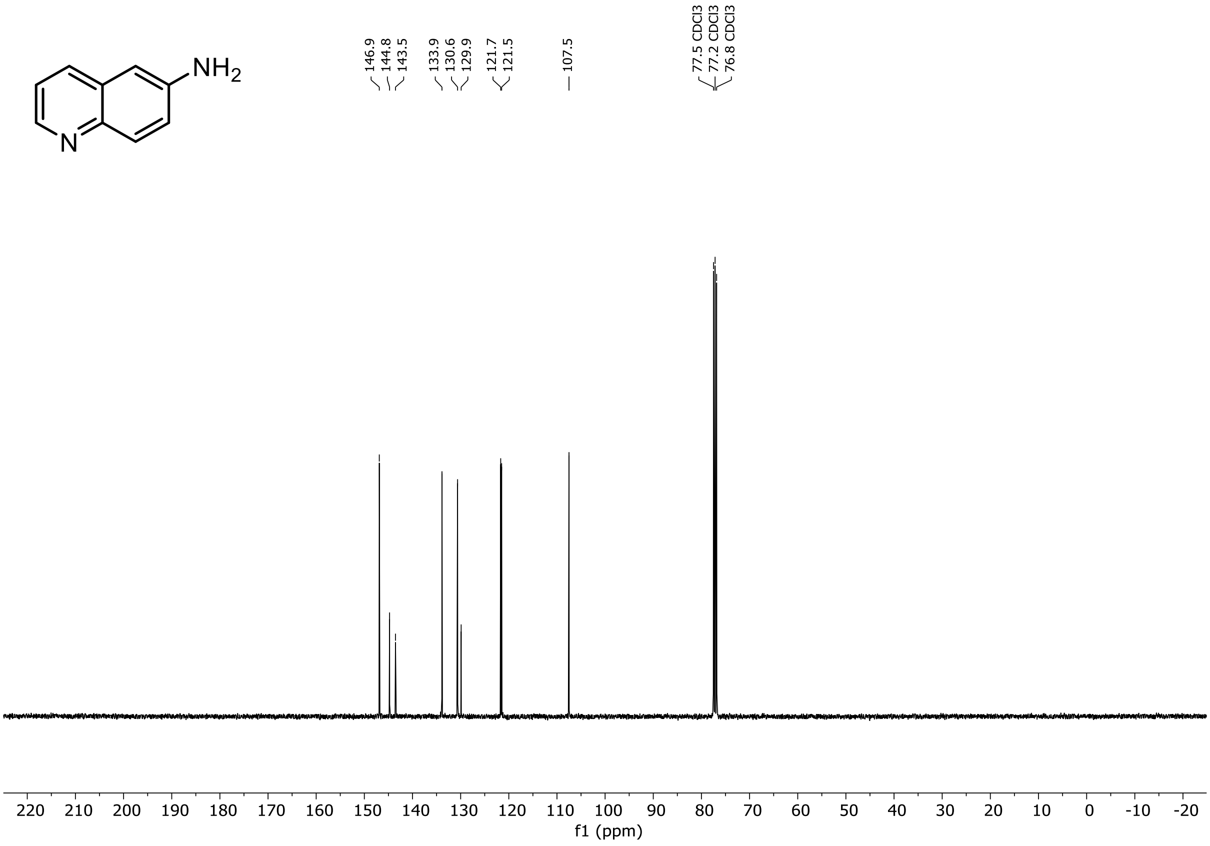
NH2
N
146.9
144.8
143.5
133.9
130.6
129.9
121.7
121.5
107.5
77.5 CDCl3
77.2 CDCl3
76.8 CDCl3
220 210 200 190 180 170 160 150 140 130 120 110 100 90 80 70 60 50 40 30 20 10 0 -10 -20
f1 (ppm)

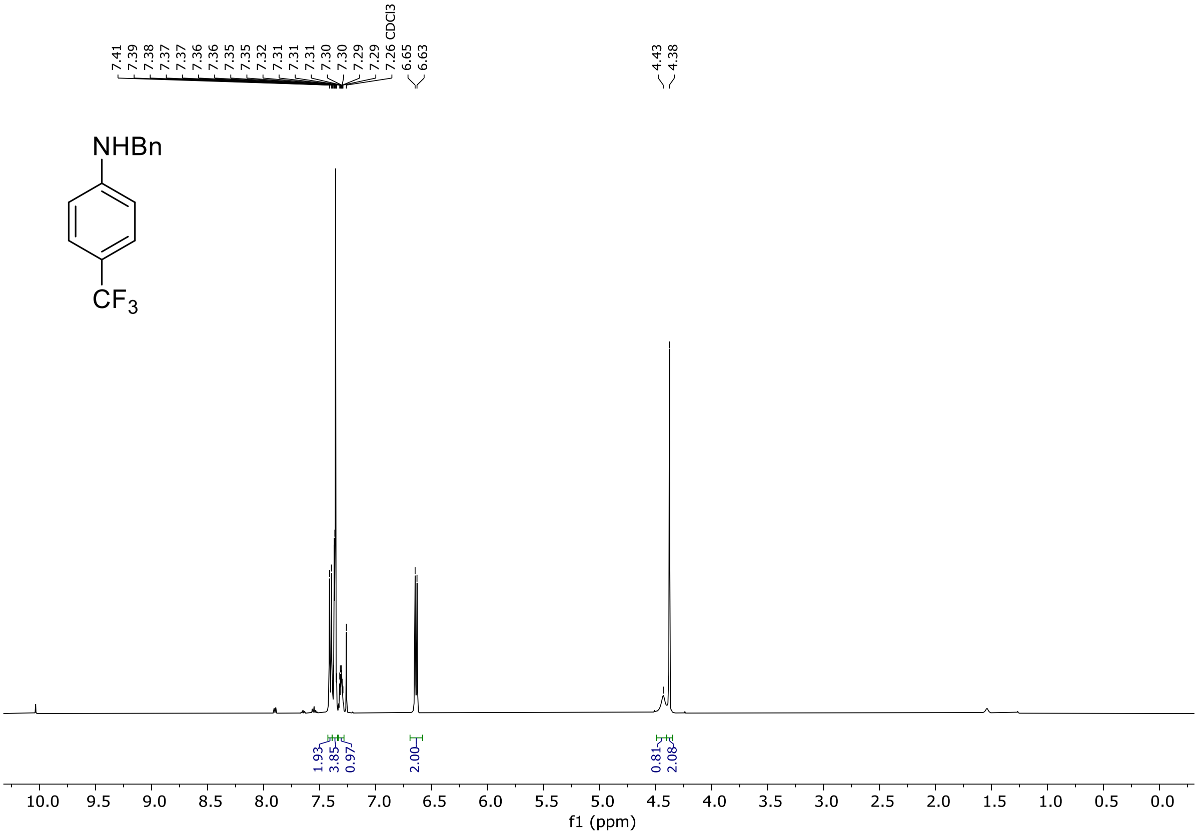
7.41
7.39
7.38
7.37
7.37
7.36
7.36
7.35
7.35
7.32
7.31
7.31
7.31
7.30
7.30
7.29
7.29
7.26 CDCl3
6.65
6.63
4.43
4.38
NHBn
CF3
1.93
3.85
0.97
2.00
0.81
2.08
f1 (ppm)

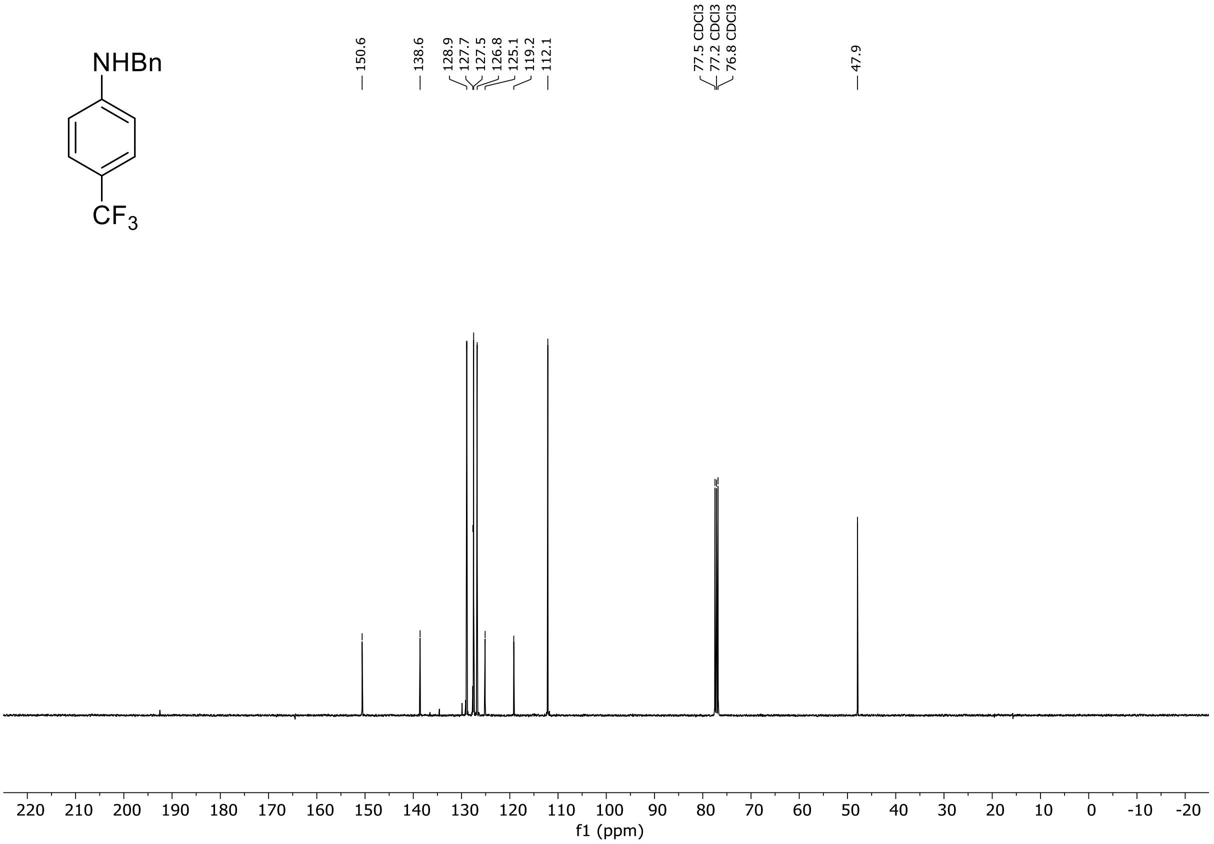
NHBn
CF3
150.6
138.6
128.9
127.7
127.5
126.8
125.1
119.2
112.1
77.5 CDCl3
77.2 CDCl3
76.8 CDCl3
47.9
f1 (ppm)

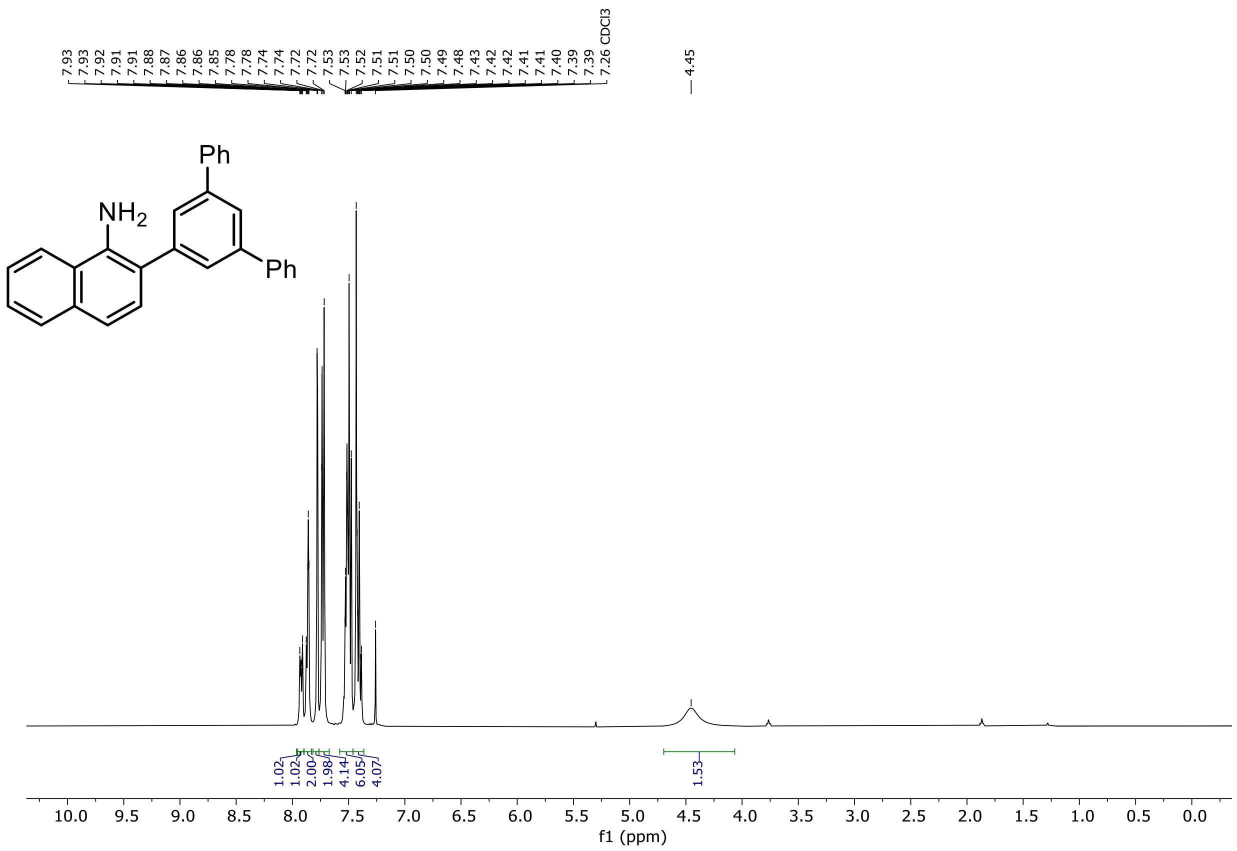
7.93 7.93 7.92 7.91 7.91 7.88 7.87 7.86 7.86 7.85 7.78 7.78 7.74 7.74 7.72 7.72 7.53 7.53 7.52 7.51 7.51 7.50 7.50 7.49 7.48 7.43 7.42 7.42 7.41 7.41 7.40 7.39 7.39 7.26 CDCl3
4.45
Ph
NH2
Ph
1.02 1.02 2.00 1.98 4.14 6.05 4.07
1.53
10.0 9.5 9.0 8.5 8.0 7.5 7.0 6.5 6.0 5.5 5.0 4.5 4.0 3.5 3.0 2.5 2.0 1.5 1.0 0.5 0.0
f1 (ppm)

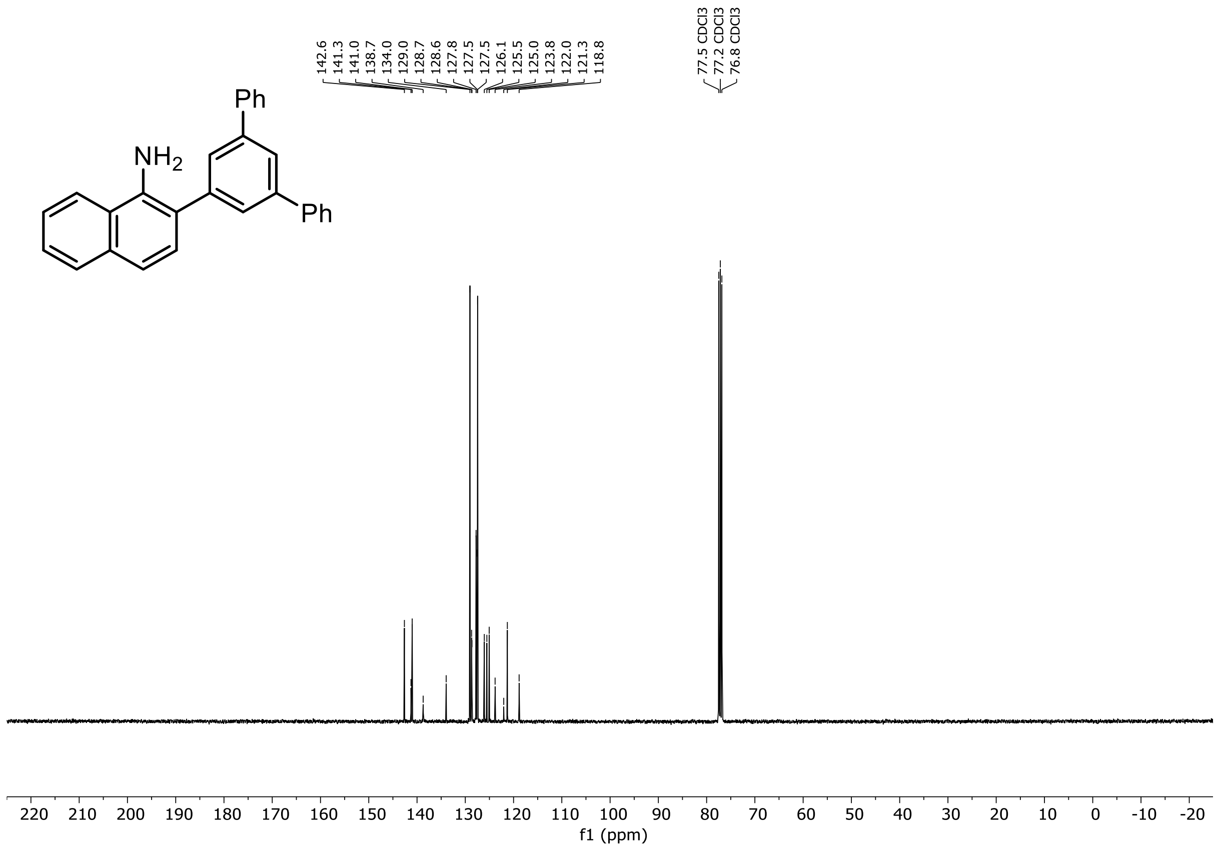
142.6 141.3 141.0 138.7 134.0 129.0 128.7 128.6 127.8 127.5 127.5 126.1 125.5 125.0 123.8 122.0 121.3 118.8
77.5 CDCl3 77.2 CDCl3 76.8 CDCl3
Ph
NH2
Ph
220 210 200 190 180 170 160 150 140 130 120 110 100 90 80 70 60 50 40 30 20 10 0 -10 -20
f1 (ppm)

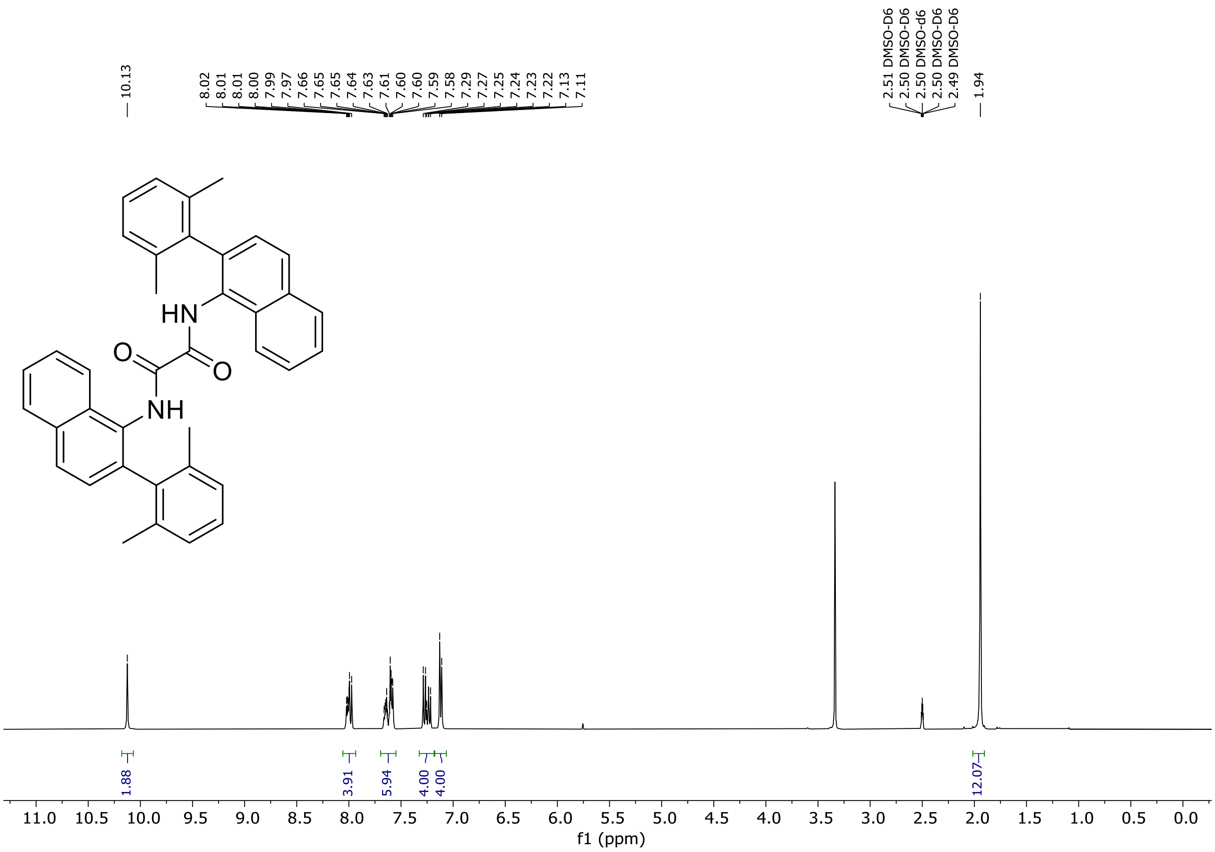
10.13
8.02
8.01
8.01
8.00
7.99
7.97
7.66
7.65
7.65
7.64
7.63
7.61
7.60
7.60
7.59
7.58
7.29
7.27
7.25
7.24
7.23
7.22
7.13
7.11
2.51 DMSO-D6
2.50 DMSO-D6
2.50 DMSO-d6
2.50 DMSO-D6
2.49 DMSO-D6
1.94
HN
O
O
NH
1.88
3.91
5.94
4.00
4.00
12.07
f1 (ppm)

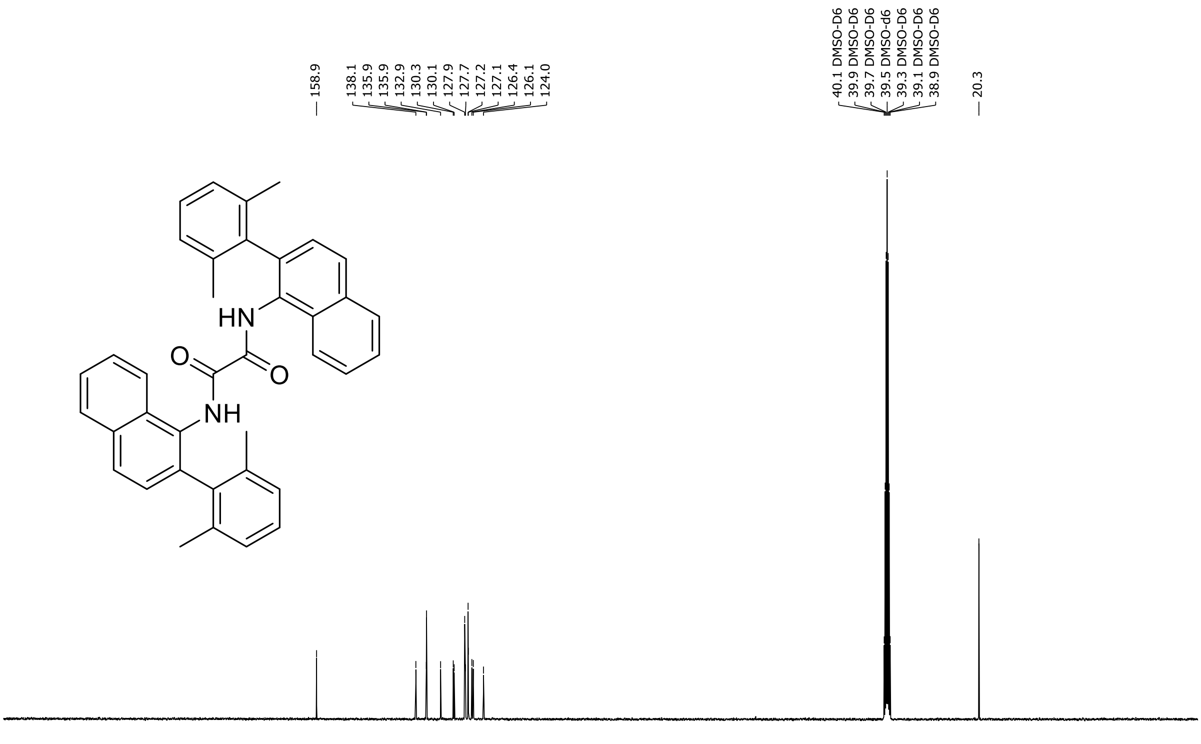
158.9
138.1
135.9
135.9
132.9
130.3
130.1
127.9
127.7
127.2
127.1
126.4
126.1
124.0
40.1 DMSO-D6
39.9 DMSO-D6
39.7 DMSO-D6
39.5 DMSO-d6
39.3 DMSO-D6
39.1 DMSO-D6
38.9 DMSO-D6
20.3
HN
O
O
NH
f1 (ppm)

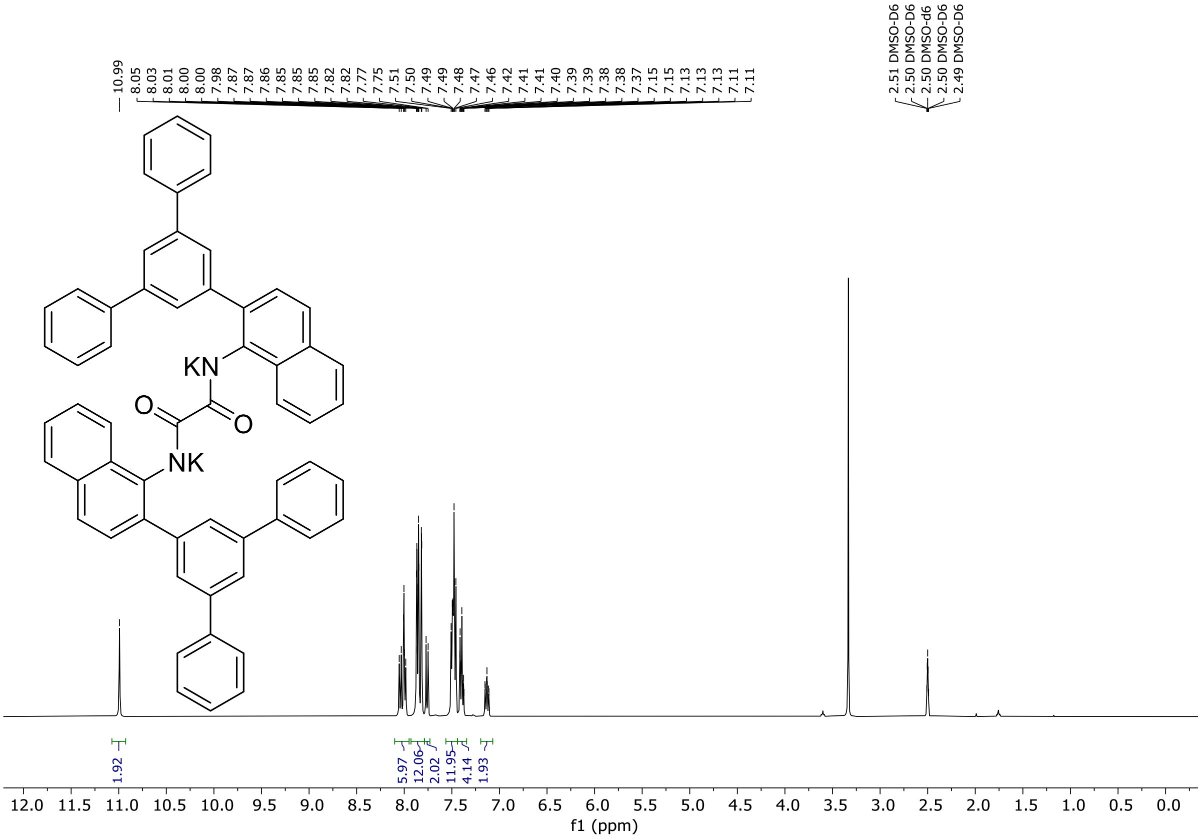
10.99
8.05 8.03 8.01 8.00 8.00 7.98 7.87 7.87 7.86 7.85 7.85 7.85 7.82 7.82 7.77 7.75 7.51 7.50 7.49 7.49 7.48 7.47 7.46 7.42 7.41 7.41 7.40 7.39 7.39 7.38 7.38 7.37 7.15 7.15 7.13 7.13 7.13 7.11 7.11
2.51 DMSO-D6 2.50 DMSO-D6 2.50 DMSO-d6 2.50 DMSO-D6 2.49 DMSO-D6
KN
O
O
NK
1.92
5.97
12.06
2.02
11.95
4.14
1.93
12.0 11.5 11.0 10.5 10.0 9.5 9.0 8.5 8.0 7.5 7.0 6.5 6.0 5.5 5.0 4.5 4.0 3.5 3.0 2.5 2.0 1.5 1.0 0.5 0.0
f1 (ppm)

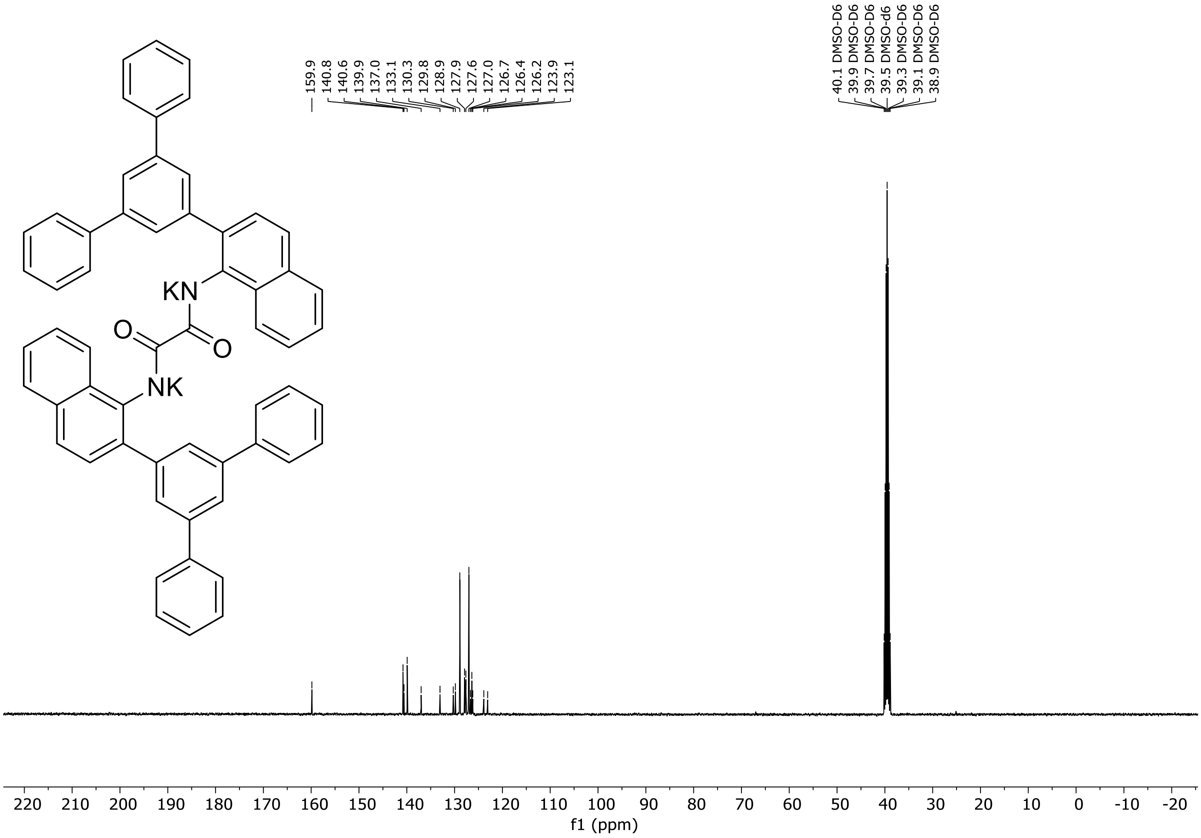
159.9
140.8 140.6 139.9 137.0 133.1 130.3 129.8 128.9 127.9 127.6 127.0 126.7 126.4 126.2 123.9 123.1
40.1 DMSO-D6 39.9 DMSO-D6 39.7 DMSO-D6 39.5 DMSO-d6 39.3 DMSO-D6 39.1 DMSO-D6 38.9 DMSO-D6
KN
O
O
NK
220 210 200 190 180 170 160 150 140 130 120 110 100 90 80 70 60 50 40 30 20 10 0 -10 -20
f1 (ppm)

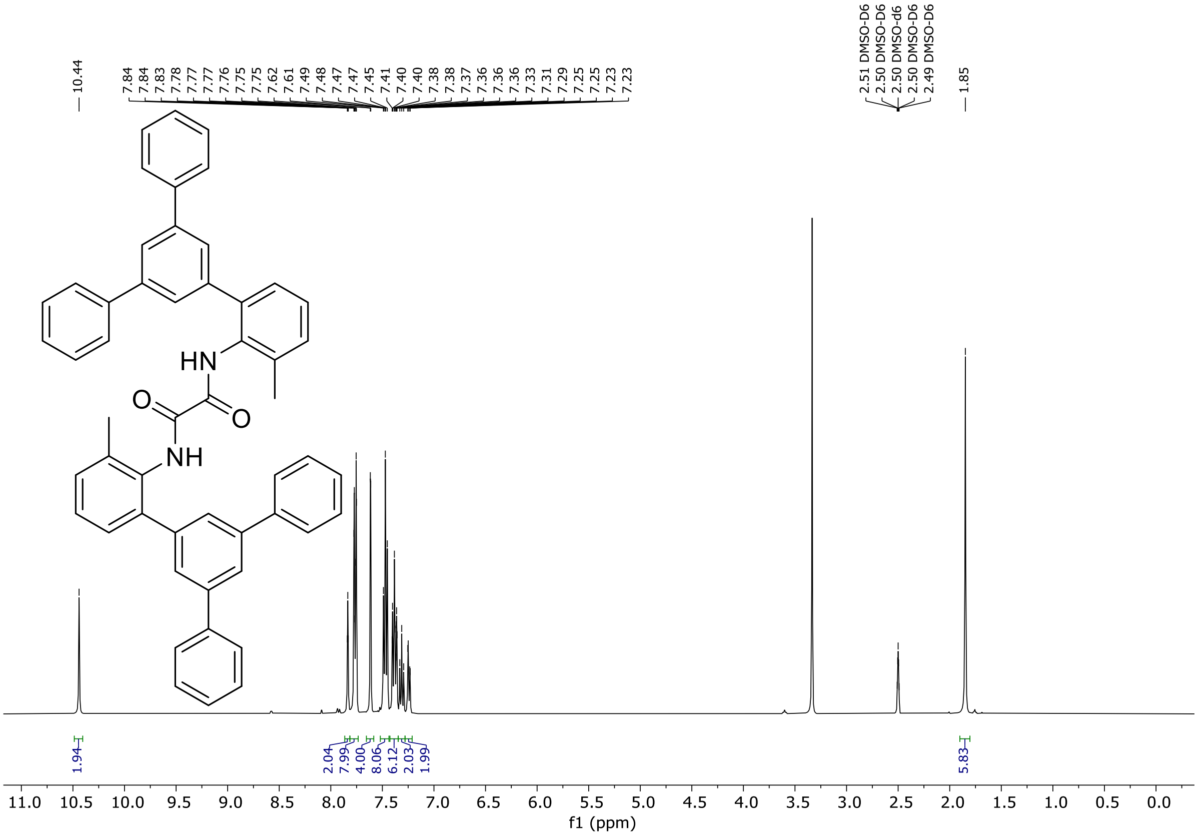
10.44
7.84 7.84 7.83 7.78 7.77 7.77 7.76 7.75 7.75 7.62 7.61 7.49 7.48 7.47 7.47 7.45 7.41 7.40 7.40 7.38 7.38 7.37 7.36 7.36 7.36 7.33 7.31 7.29 7.25 7.25 7.23 7.23
2.51 DMSO-D6
2.50 DMSO-D6
2.50 DMSO-d6
2.50 DMSO-D6
2.49 DMSO-D6
1.85
HN
O
O
NH
1.94
2.04 7.99 4.00 8.06 6.12 2.03 1.99
5.83
11.0 10.5 10.0 9.5 9.0 8.5 8.0 7.5 7.0 6.5 6.0 5.5 5.0 4.5 4.0 3.5 3.0 2.5 2.0 1.5 1.0 0.5 0.0
f1 (ppm)

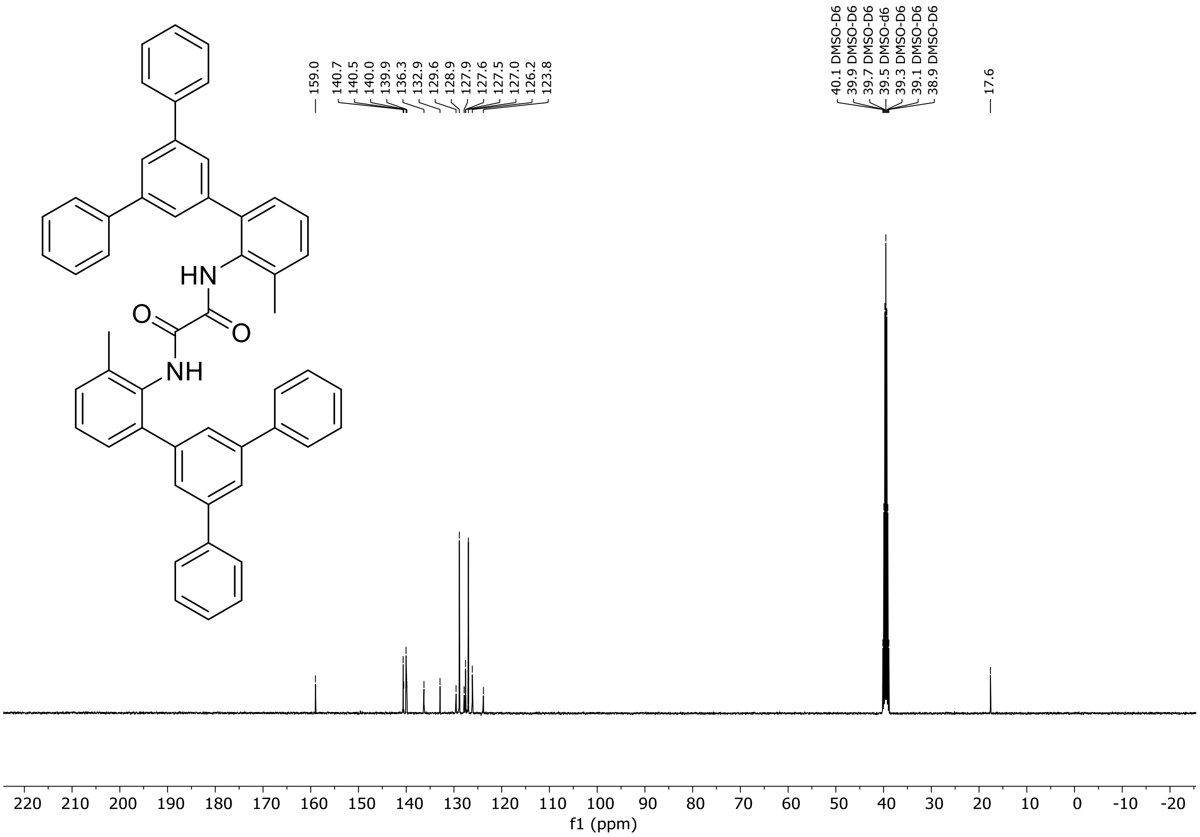
159.0
140.7 140.5 140.0 139.9 136.3 132.9 129.6 128.9 127.9 127.6 127.5 127.0 126.2 123.8
40.1 DMSO-D6
39.9 DMSO-D6
39.7 DMSO-D6
39.5 DMSO-d6
39.3 DMSO-D6
39.1 DMSO-D6
38.9 DMSO-D6
17.6
HN
O
O
NH
220 210 200 190 180 170 160 150 140 130 120 110 100 90 80 70 60 50 40 30 20 10 0 -10 -20
f1 (ppm)

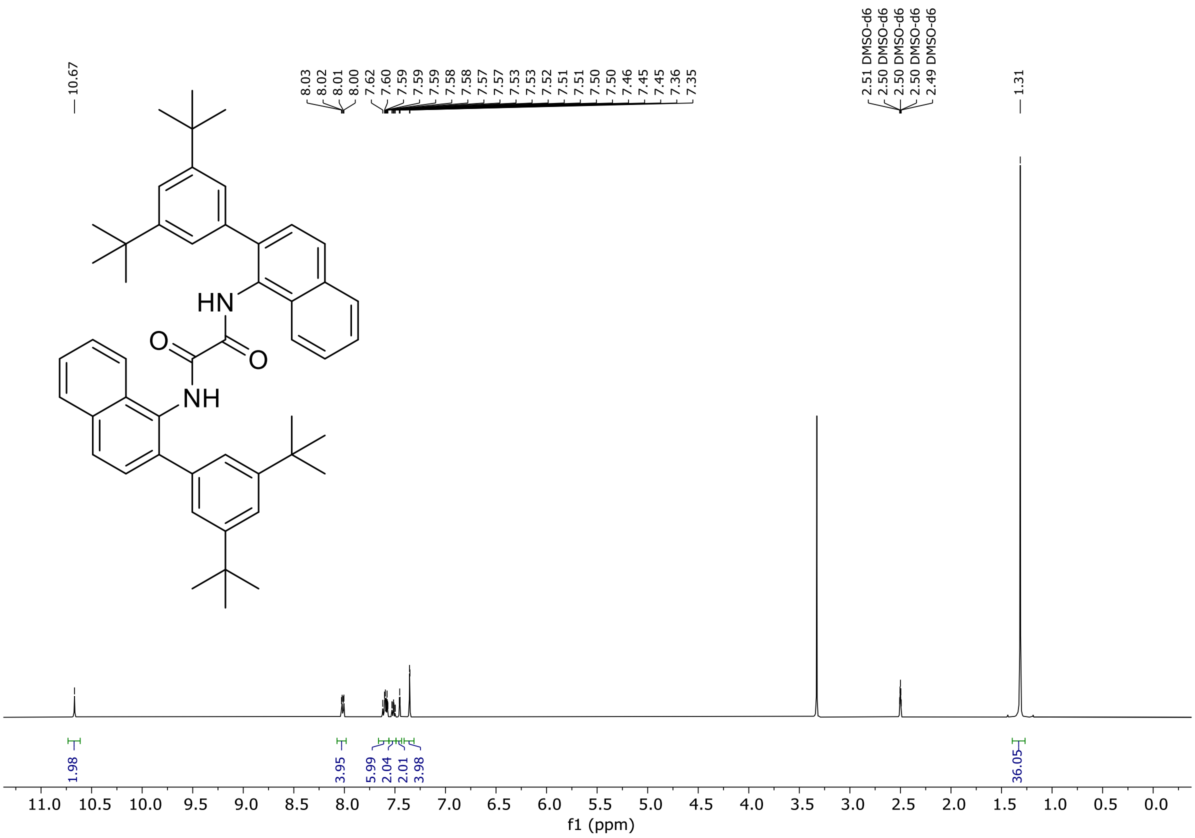
10.67
8.03
8.02
8.01
8.00
7.62
7.60
7.59
7.59
7.59
7.58
7.58
7.57
7.57
7.53
7.53
7.52
7.51
7.51
7.50
7.50
7.46
7.45
7.45
7.36
7.35
2.51 DMSO-d6
2.50 DMSO-d6
2.50 DMSO-d6
2.50 DMSO-d6
2.49 DMSO-d6
1.31
HN
O
O
NH
1.98
3.95
5.99
2.04
2.01
3.98
36.05
11.0 10.5 10.0 9.5 9.0 8.5 8.0 7.5 7.0 6.5 6.0 5.5 5.0 4.5 4.0 3.5 3.0 2.5 2.0 1.5 1.0 0.5 0.0
f1 (ppm)

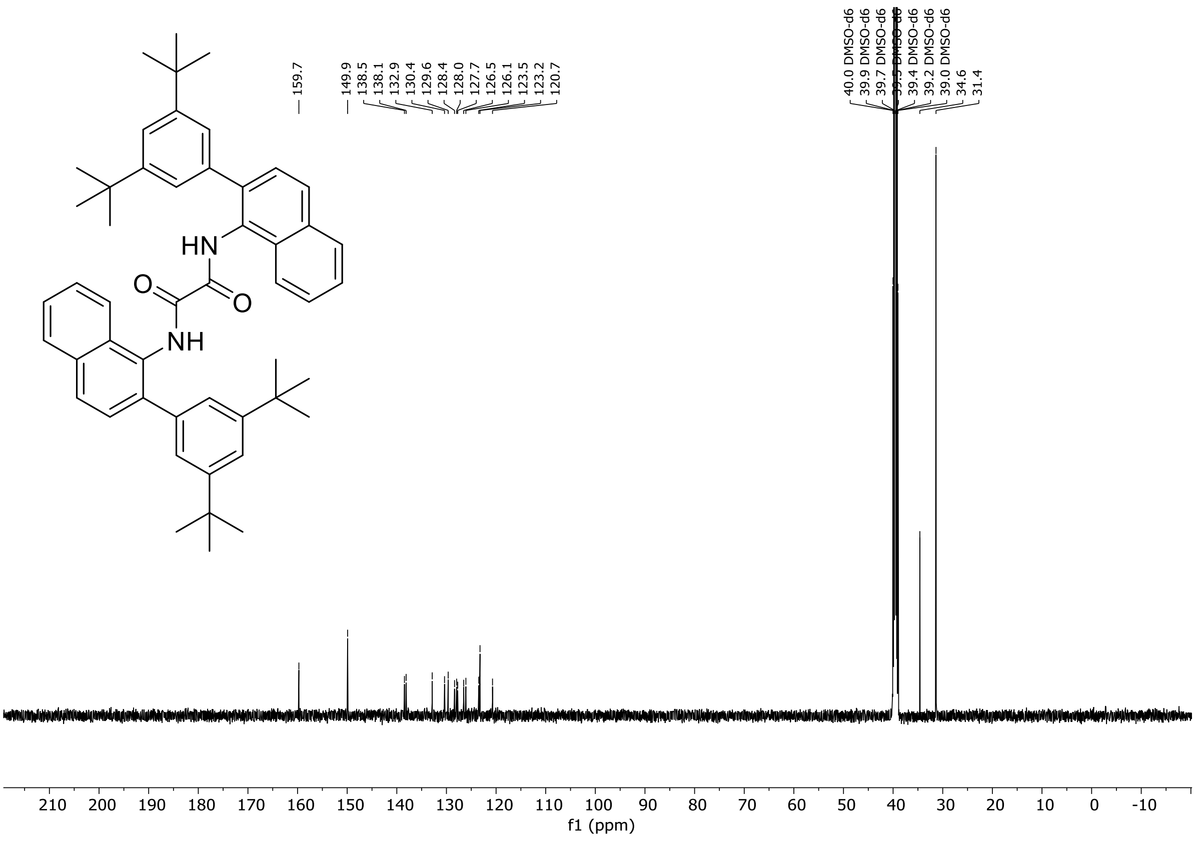
159.7
149.9
138.5
138.1
132.9
130.4
129.6
128.4
128.0
127.7
126.5
126.1
123.5
123.2
120.7
40.0 DMSO-d6
39.9 DMSO-d6
39.7 DMSO-d6
39.5 DMSO-d6
39.4 DMSO-d6
39.2 DMSO-d6
39.0 DMSO-d6
34.6
31.4
HN
O
O
NH
210 200 190 180 170 160 150 140 130 120 110 100 90 80 70 60 50 40 30 20 10 0 -10
f1 (ppm)

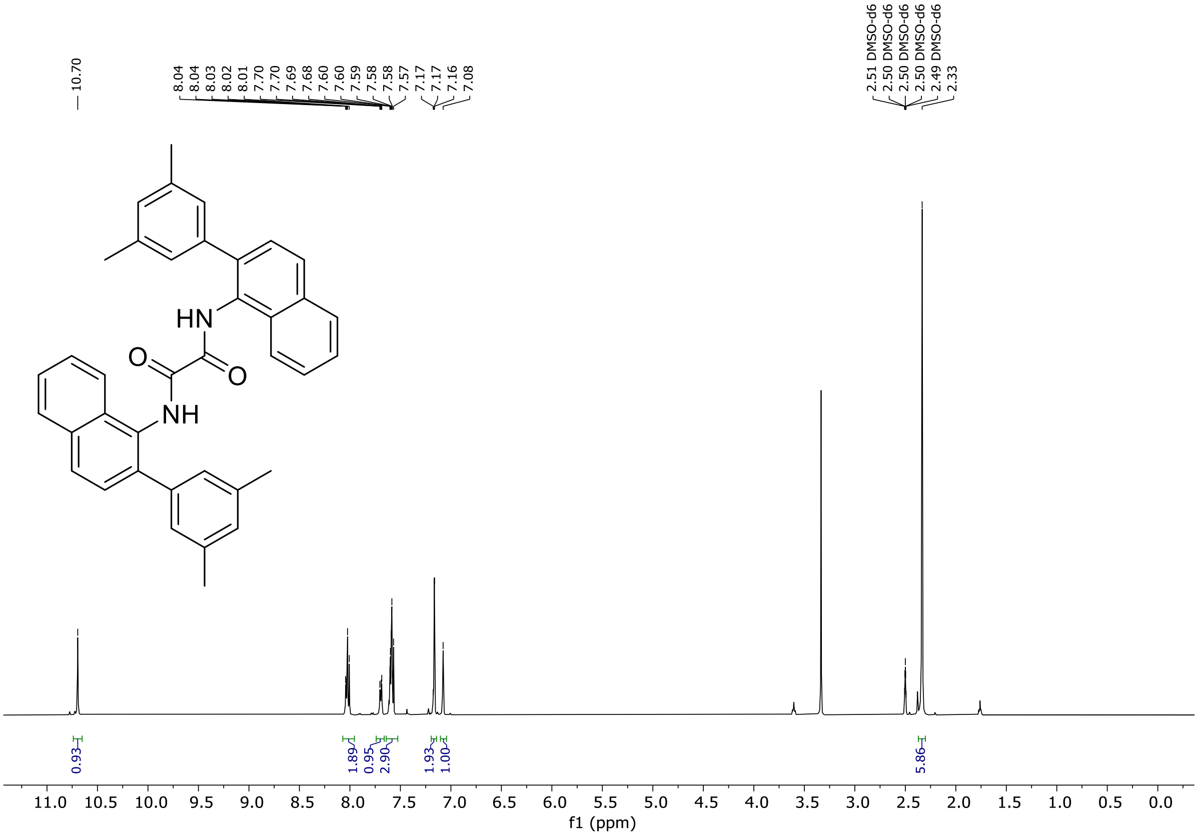
10.70
8.04
8.04
8.03
8.02
8.01
7.70
7.70
7.69
7.68
7.60
7.60
7.59
7.58
7.58
7.57
7.17
7.17
7.16
7.08
2.51 DMSO-d6
2.50 DMSO-d6
2.50 DMSO-d6
2.50 DMSO-d6
2.49 DMSO-d6
2.33
HN
O
O
NH
0.93
1.89
0.95
2.90
1.93
1.00
5.86
11.0 10.5 10.0 9.5 9.0 8.5 8.0 7.5 7.0 6.5 6.0 5.5 5.0 4.5 4.0 3.5 3.0 2.5 2.0 1.5 1.0 0.5 0.0
f1 (ppm)

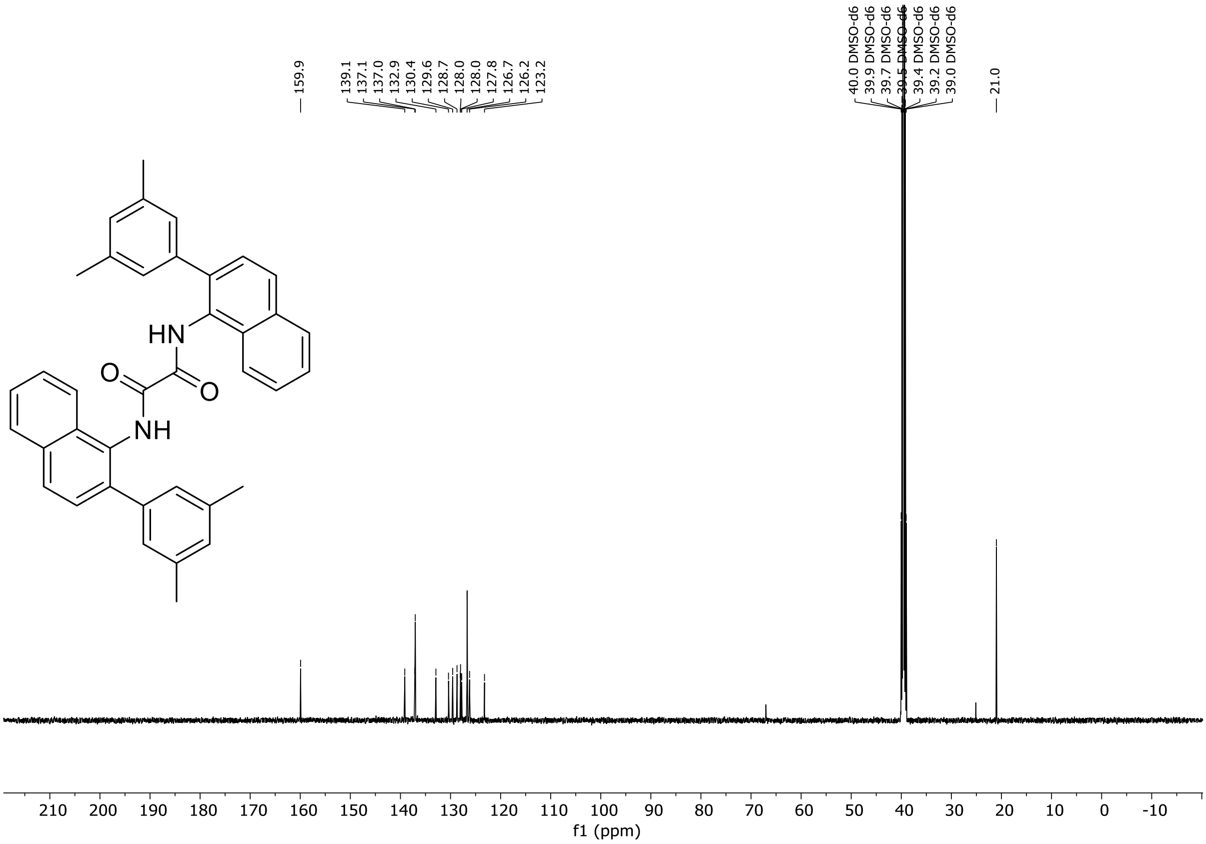
159.9
139.1
137.1
137.0
132.9
130.4
129.6
128.7
128.0
128.0
127.8
126.7
126.2
123.2
40.0 DMSO-d6
39.9 DMSO-d6
39.7 DMSO-d6
39.5 DMSO-d6
39.4 DMSO-d6
39.2 DMSO-d6
39.0 DMSO-d6
21.0
HN
O
O
NH
210 200 190 180 170 160 150 140 130 120 110 100 90 80 70 60 50 40 30 20 10 0 -10
f1 (ppm)

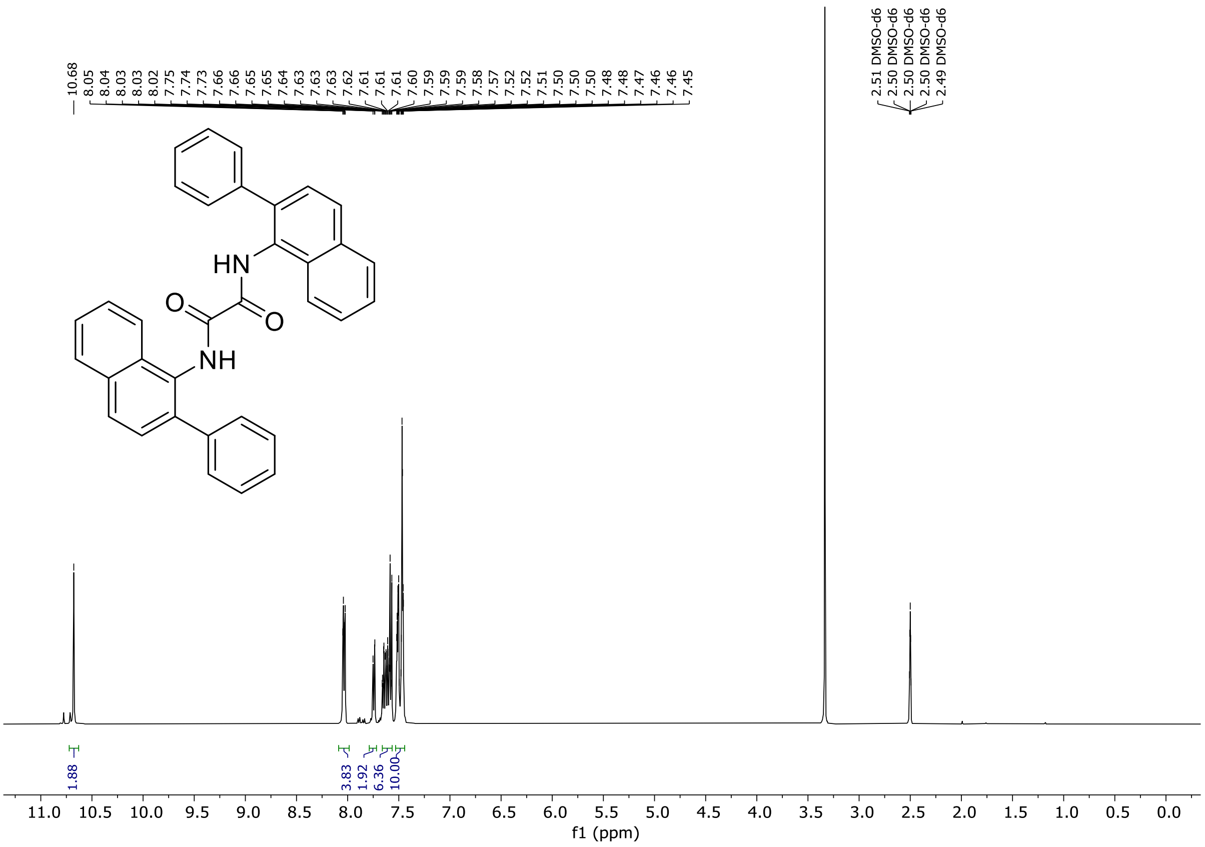

10.68
8.05
8.04
8.03
8.03
8.02
7.75
7.74
7.73
7.66
7.66
7.65
7.65
7.64
7.63
7.63
7.63
7.62
7.61
7.61
7.61
7.60
7.59
7.59
7.59
7.58
7.57
7.52
7.52
7.51
7.50
7.50
7.50
7.48
7.47
7.46
7.46
7.45
2.51 DMSO-d6
2.50 DMSO-d6
2.50 DMSO-d6
2.50 DMSO-d6
2.49 DMSO-d6
HN
O
O
NH
1.88
3.83
1.92
6.36
10.00
11.0 10.5 10.0 9.5 9.0 8.5 8.0 7.5 7.0 6.5 6.0 5.5 5.0 4.5 4.0 3.5 3.0 2.5 2.0 1.5 1.0 0.5 0.0
f1 (ppm)

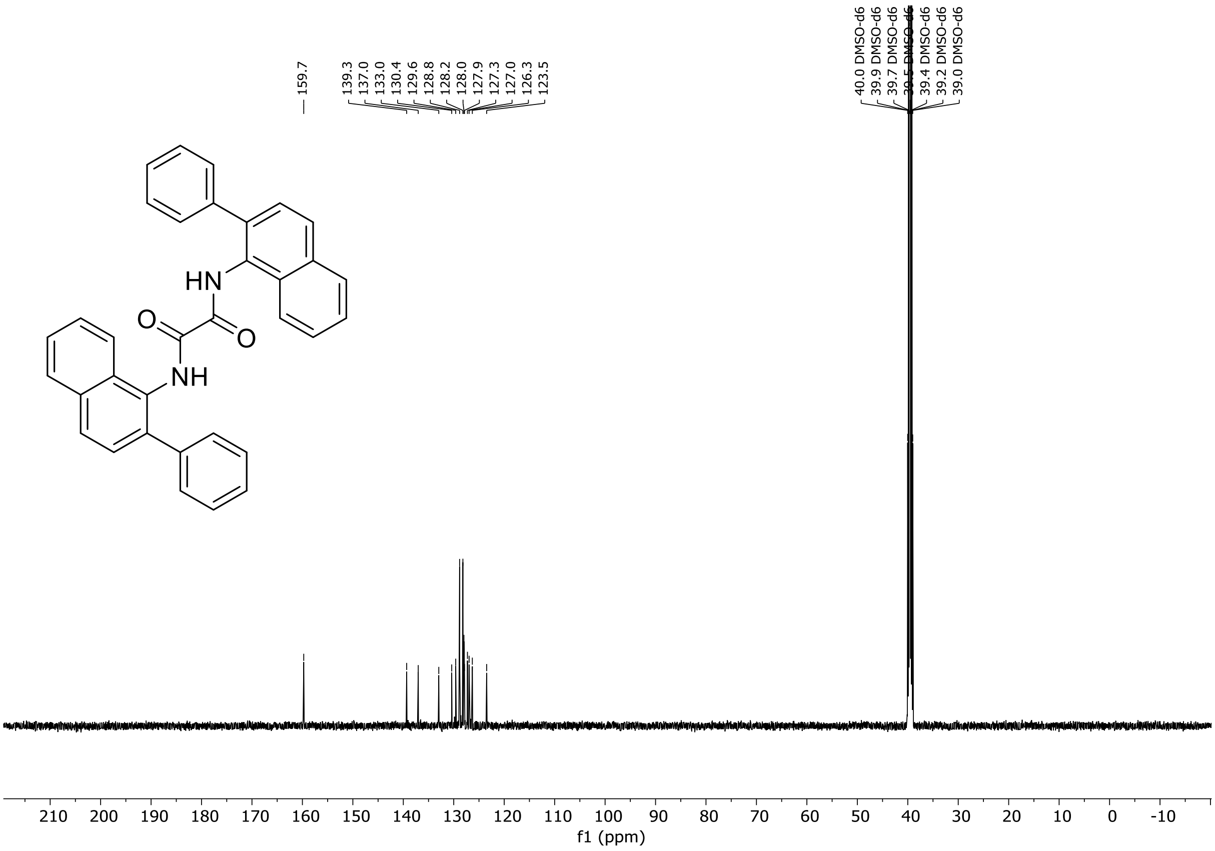

159.7
139.3
137.0
133.0
130.4
129.6
128.8
128.2
128.0
127.9
127.3
127.0
126.3
123.5
40.0 DMSO-d6
39.9 DMSO-d6
39.7 DMSO-d6
39.5 DMSO-d6
39.4 DMSO-d6
39.2 DMSO-d6
39.0 DMSO-d6
HN
O
O
NH
210 200 190 180 170 160 150 140 130 120 110 100 90 80 70 60 50 40 30 20 10 0 -10
f1 (ppm)

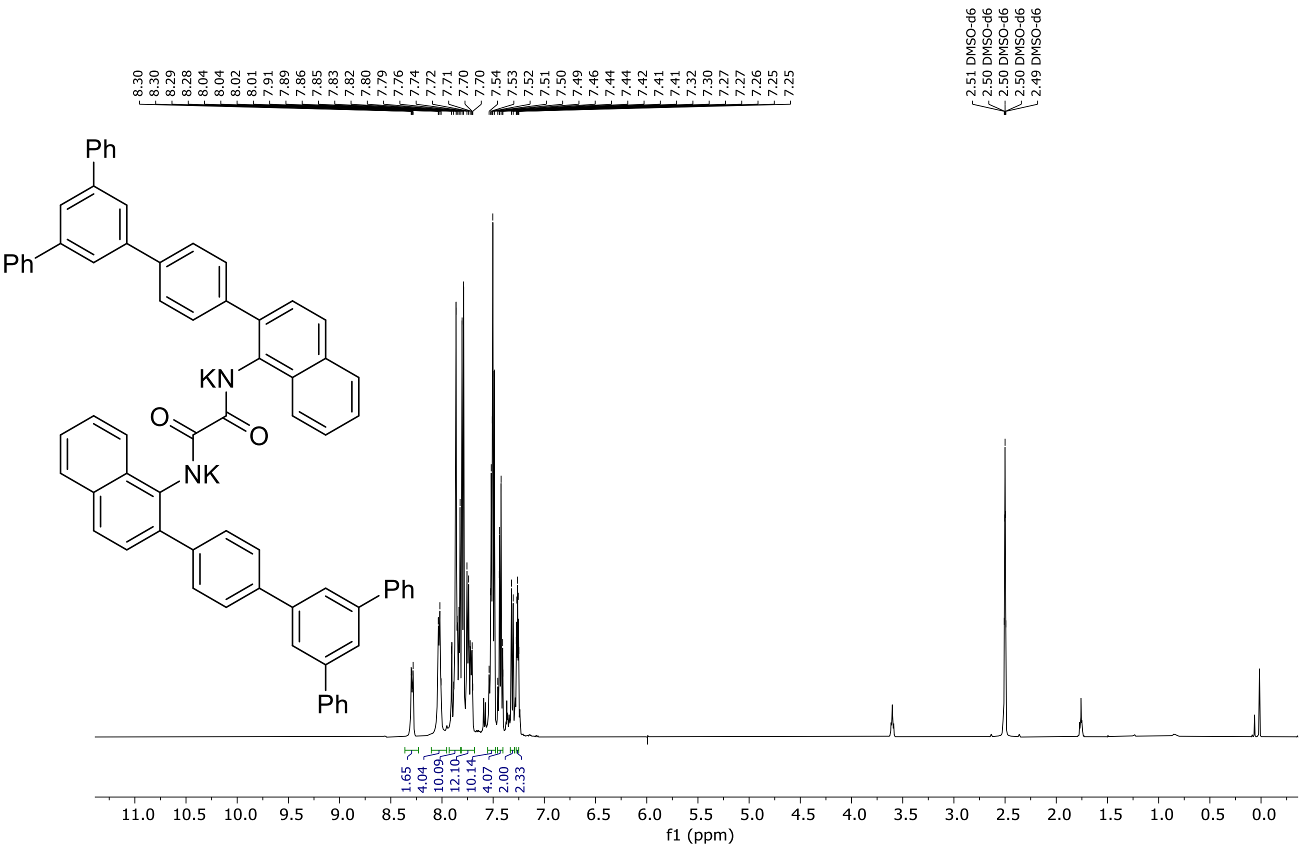

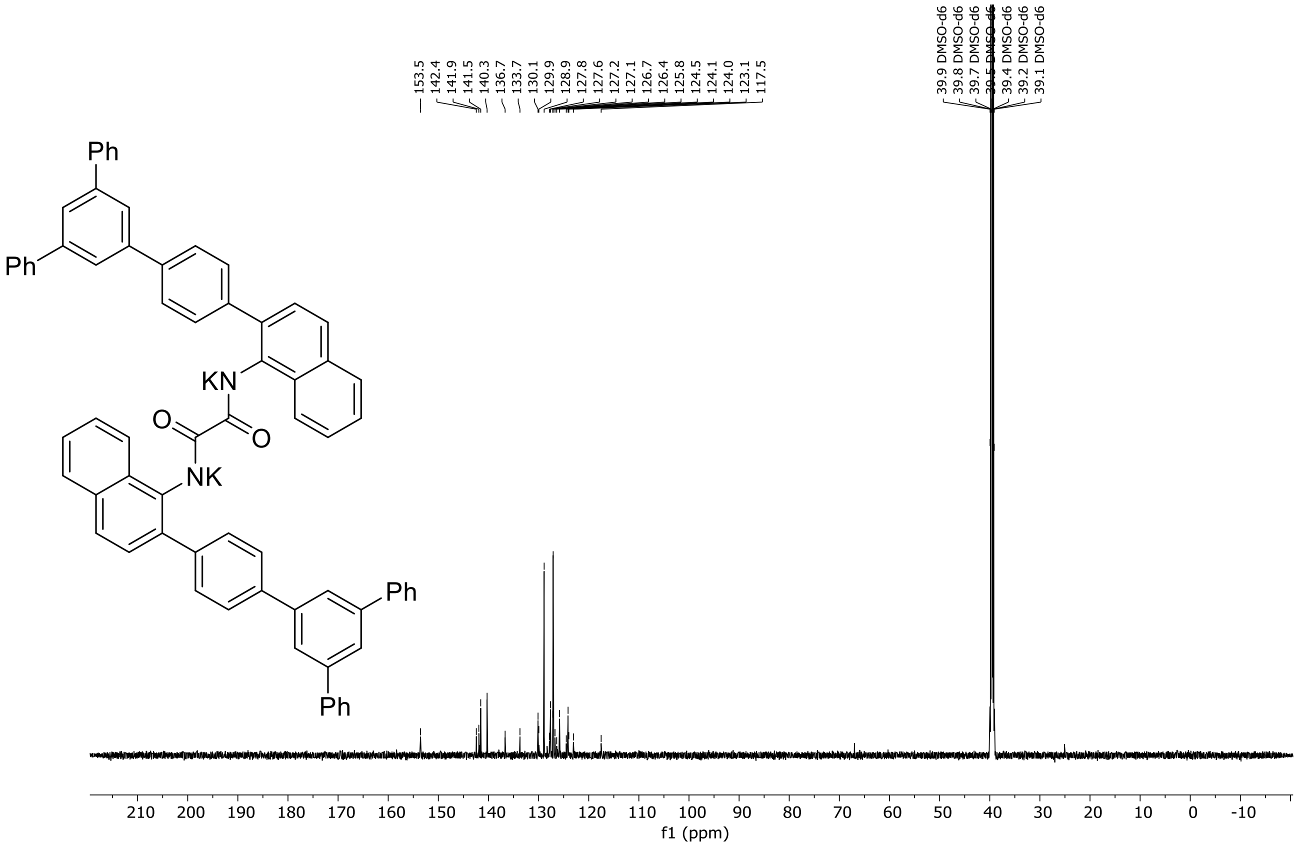

15. X-Ray Crystallographic Data

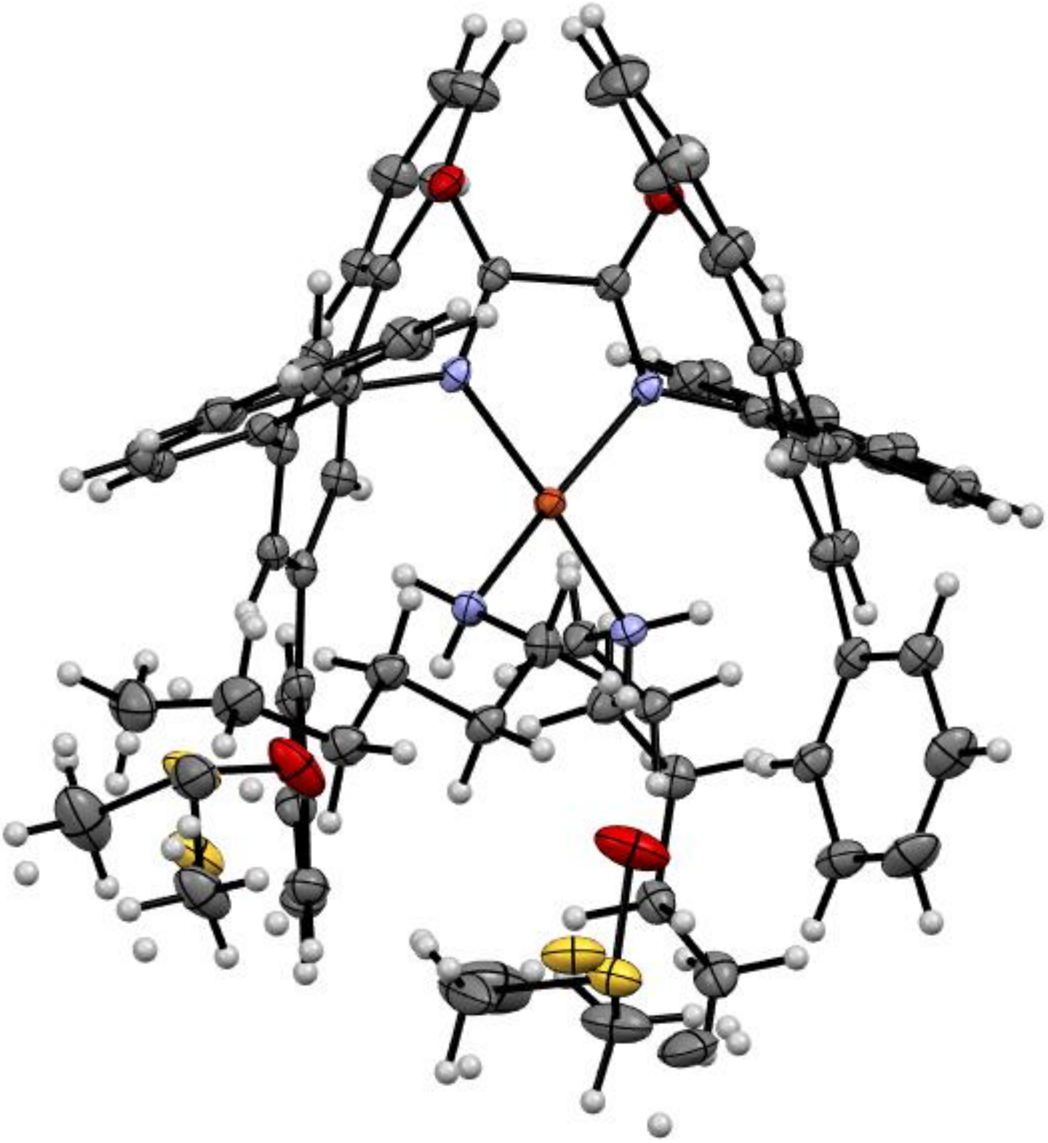

Figure 13: X-Ray structure of Cu-A showing 50% probability displacement ellipsoids. CCDC: 2499046

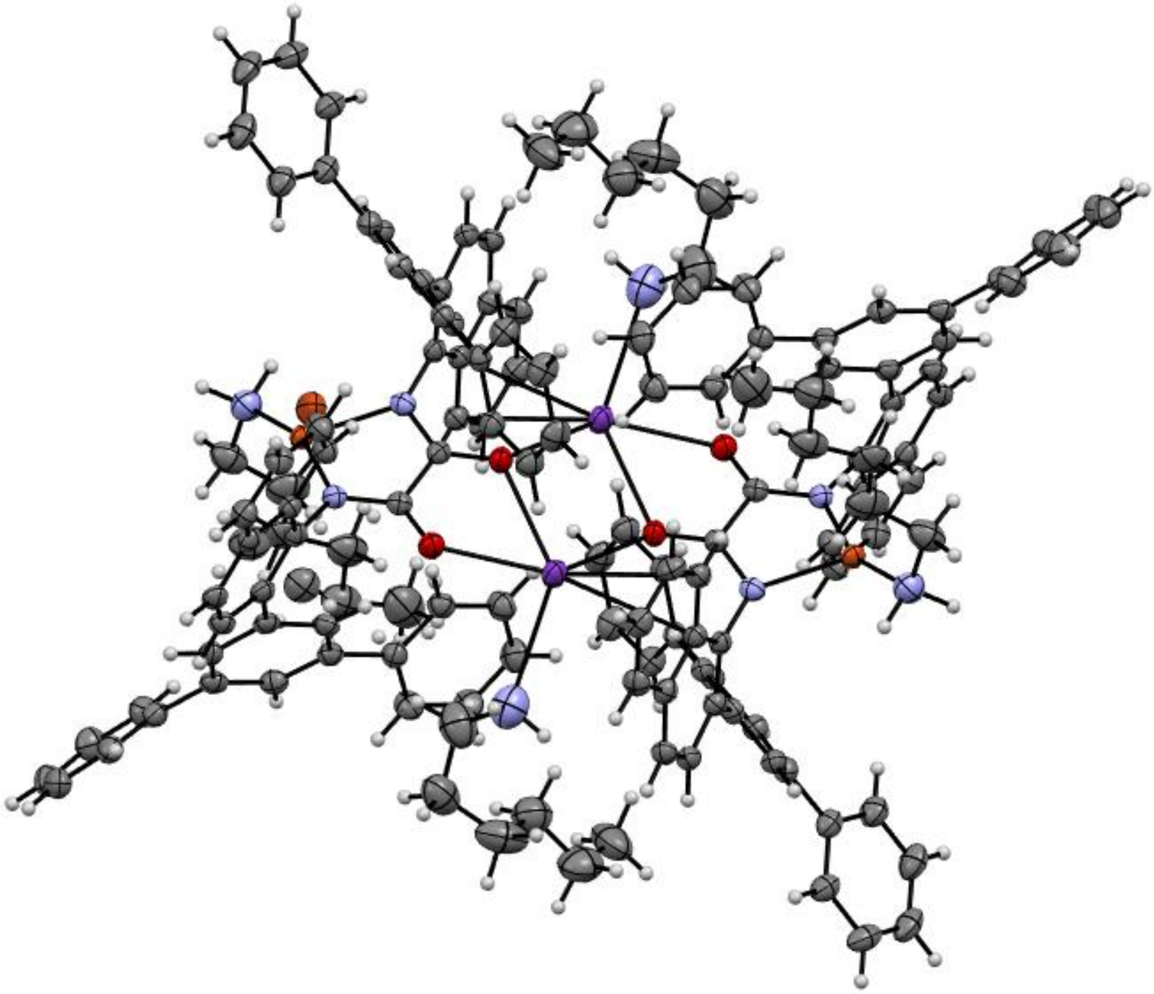

Figure 14: X-Ray structure of Cu-A showing 50% probability displacement ellipsoids. CCDC: 2503981

16. References

1. Stoll, S.; Schweiger, A., EasySpin, a comprehensive software package for spectral simulation and analysis in EPR. *Journal of Magnetic Resonance* **2006,** *178*, 42-55.
2. Neese, F., The ORCA program system. *WIREs Computational Molecular Science* **2012,** *2*, 73-78.
3. (a) Staroverov, V. N.; Scuseria, G. E.; Tao, J.; Perdew, J. P., Comparative assessment of a new nonempirical density functional: Molecules and hydrogen-bonded complexes. *The Journal of Chemical Physics* **2003,** *119*, 12129-12137; (b) Neese, F.; Wennmohs, F.; Hansen, A.; Becker, U., Efficient, approximate and parallel Hartree–Fock and hybrid DFT calculations. A 'chain-of-spheres' algorithm for the Hartree–Fock exchange. *Chem. Phys.* **2009,** *356*, 98-109.
4. Orio, M.; Pantazis, D. A.; Neese, F., Density functional theory. *Photosynth. Res.* **2009,** *102*, 443-453.
5. Weigend, F., Accurate Coulomb-fitting basis sets for H to Rn. *Physical Chemistry Chemical Physics* **2006,** *8*, 1057-1065.
6. (a) Pantazis, D. A.; Neese, F., All-Electron Scalar Relativistic Basis Sets for the Lanthanides. *Journal of Chemical Theory and Computation* **2009,** *5*, 2229-2238; (b) Pantazis, D. A.; Chen, X.-Y.; Landis, C. R.; Neese, F., All-Electron Scalar Relativistic Basis Sets for Third-Row Transition Metal Atoms. *Journal of Chemical Theory and Computation* **2008,** *4*, 908-919.
7. (a) Barone, V.; Cossi, M., Quantum Calculation of Molecular Energies and Energy Gradients in Solution by a Conductor Solvent Model. *The Journal of Physical Chemistry A* **1998,** *102*, 1995-2001; (b) Grimme, S.; Ehrlich, S.; Goerigk, L., Effect of the damping function in dispersion corrected density functional theory. *J. Comput. Chem.* **2011,** *32*, 1456-1465.
8. (a) Sharma, M.; Leadbeater, N. E., Preparation of Symmetric and Nonsymmetric Imines from Primary Benzyl Amines by Means of an Oxidative Functionalization Reaction Using an Oxoammonium Salt Bearing the Nitrate Anion. *Synlett* **2025,** *36*, 1231-1236; (b) Schroeder, C. M.; Gasior, A.; Leadbeater, N. E., Oxidative Functionalization of Amines Using an Electrocatalytic Aminoxyl-Mediated

Reaction: Preparation of N-Benzylidenebenzylamines from Benzylamines. *Eur. J. Org. Chem.* **2025,** *28*, e202500720.
